# Diastereoselective Umpolung cyclisation of ketones promoted by hypervalent iodine†

**DOI:** 10.1039/d5sc01085c

**Published:** 2025-05-13

**Authors:** Giulia Iannelli, Philipp Spieß, Ricardo Meyrelles, Daniel Kaiser, Boris Maryasin, Leticia González, Nuno Maulide

**Affiliations:** a Institute of Organic Chemistry, University of Vienna Währinger Straße 38 1090 Vienna Austria nuno.maulide@univie.ac.at; b Institute of Theoretical Chemistry, University of Vienna Währinger Straße 17 1090 Vienna Austria

## Abstract

Umpolung reactivity facilitated by hypervalent iodine has emerged as an appealing method for the efficient α-functionalization of ketones. However, skeletal reorganisation or migration reactions remain comparatively underexplored, primarily due to the challenging taming of transient carbocationic intermediates. In this study, we introduce a method for the functionalisation of ketones, employing a 6*-endo-trig* cyclisation initiated by Umpolung of silyl enol ethers, resulting in the diastereoselective formation of *cis*-substituted cyclohexanes. Additional investigations, both experimental and computational, give insight into the mechanistic intricacies of this process, and shed light on an unconventional iodine(iii)-reactivation mechanism.

## Abstract 2

Ketone α-Umpolung using an underexplored class of hypervalent iodine reagents enables 6-*endo-trig* cyclisations on unbiased systems to yield *cis*-configured 1,3-disubstituted cyclohexanols.

## Graphical abstract

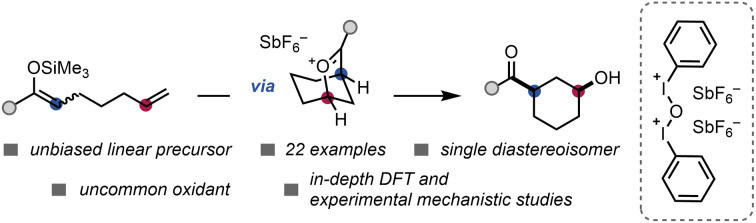

## Introduction

**Scheme 1:**
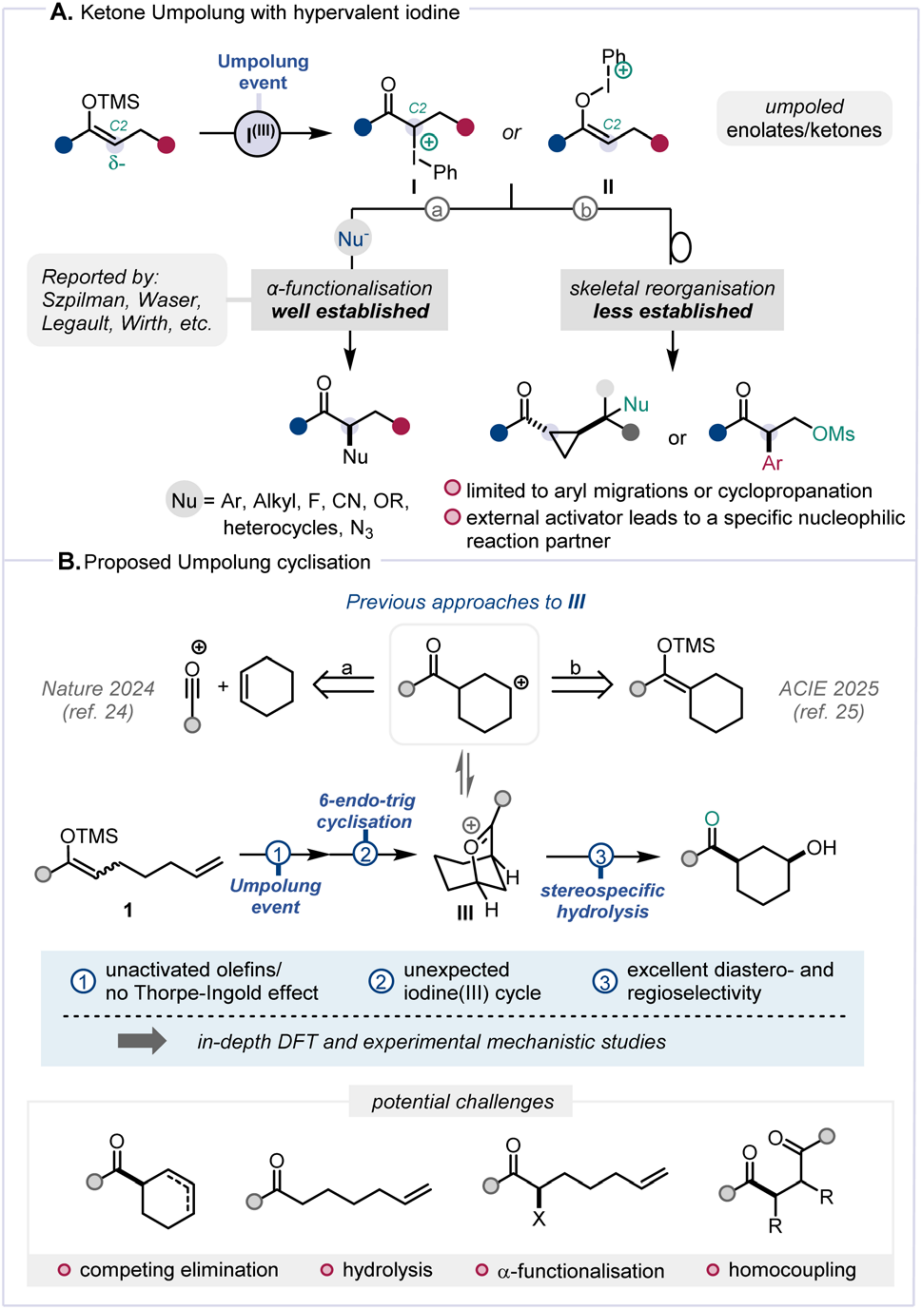
(A) Ketone Umpolung with iodine(iii). (B) Proposed transformation and potential pitfalls.

Umpolung, a term referring to the reversal of inherent polarity, has become an established and effective strategy in organic synthesis.^1^ In recent years, the so-called “α-position Umpolung” of ketone derivatives has garnered substantial interest and, among various other methods,^2–5^ hypervalent iodine sources show considerable promise (Scheme 1A).^6,7^ In particular, the interaction between hypervalent iodine reagents and silyl enol ethers triggers the generation of α-carbonyl carbocationic synthons (I/II),^8,9^ whose capture by nucleophilic species enables a broad range of α-functionalisation reactions (Scheme 1A, path a).^4,8,10–19^ A range of hypervalent iodine reagents, including Koser's reagent,^10^ (diacetoxyiodo)benzene (DIB),^15^ benziodoxolone (BX),^20^ and *p*-iodotoluene difluoride,^21^ have proven effective to elicit such Umpolung reactivity. Less often, α-carbocationic synthons have also been shown to readily engage in skeletal reorganisation and migration reactions (Scheme 1A, path b),^22,23^ painting a landscape where ketone functionalisation through Umpolung has been largely limited to intermolecular regimes.^11,16,17^

Given our ongoing interest in the interaction between π-systems and umpoled synthons (*cf.*I/II), we were intrigued by the dearth of Umpolung-mediated cyclisations and were drawn to explore the latent reactivity of substrates such as 1 (Scheme 1B). Building on our previous research involving the formation of oxocarbenium species,^24,25^ we enquired whether a 6-*endo-trig* cyclisation of the alkene onto an umpoled ketone could mediate the formation of an intermediate akin to III (Scheme 1B).

Notwithstanding favourable precedent for formation of species related to III,^24,25^ a number of pitfalls were anticipated. For instance, the absence of typical accelerating factors such as the Thorpe–Ingold effect or electronic olefin activation was deemed challenging.^26,27^ Competing processes in the proposed transformation, including known reactions such as elimination, hydrolysis, α-functionalisation, and homocoupling (Scheme 1B, bottom)^8,22^ rendered the ultimately successful development of this Umpolung cyclisation a challenging endeavour.

Herein we report a study on Umpolung π-cyclisation processes whereby the deployment of an unconventional iodonium reagent was key to mediate stereo- and regioselective formation of *cis-*configured 1,3-disubstituted cyclohexanols in the absence of biasing elements. Furthermore, we offer a deeper understanding of the underlying mechanistic intricacies of this Umpolung-triggered cyclisation sequence through a combination of computational and experimental studies.

## Results and discussion

**Scheme 2:**
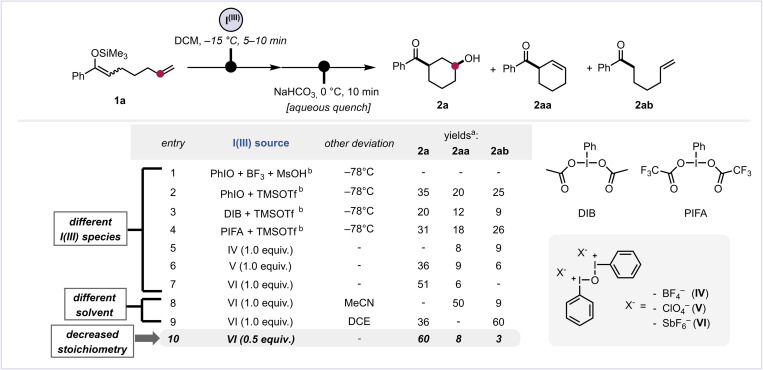
Optimisation studies of the Umpolung cyclisation. All reactions were carried out on a 0.1 mmol scale with 1a (1.0 equiv.). For more reaction details, see the ESI.† (a) NMR yield determined using mesitylene as the internal standard. (b) 1.2 equiv. were used for both iodine reagent and activator. MsOH = methanesulfonic acid. TMSOTf = trimethylsilyl trifluoromethanesulfonate. DIB = (diacetoxyiodo)benzene. PIFA = (bis(trifluoroacetoxy)iodo)benzene. DCE = 1,2-dichloroethane.

We began our investigation with substrate 1a (Scheme 2). Surprisingly, under the conditions previously optimised for the cyclopropanation of silyl enol ethers (Scheme 1A, path b),^22,23^ utilising BF_3_ and MsOH (entry 1), no trace of cyclised products was detected, and only a complex mixture was observed. Encouragingly, the use of iodosobenzene (PhIO) activated by TMSOTf (entry 2) afforded small quantities of the hydroxylated product 2a as a single diastereomer, exhibiting a *cis*-relationship between the acyl and hydroxyl groups. However, significant amounts of side products were also detected, including elimination (2aa) and hydrolysis of the silylated starting material (2ab), necessitating further optimisation of the reaction conditions. Subsequent alterations to the iodine oxidation source failed to result in significant improvement (entries 3 and 4), underscoring the substantial challenge of mitigating undesired side reactions with conventional hypervalent iodine sources. Consequently, we considered the use of an underexplored class of stable iodine sources, derived from iodosobenzene through condensation in the presence of an acid, as developed by Zefirov, Caple and co-workers: dicationic μ-oxo-bis[(phenyl)iodine] reagents IV–VI.^28,29^ These hypervalent iodine species lack a nucleophilic ligand and demonstrate pronounced electrophilicity, obviating the need for external activators—otherwise invariably a source of a nucleophilic or basic counteranion. Despite these intriguing chemical properties, their full potential in more complex synthetic contexts—beyond silyl enolate homocoupling^29^—remained virtually unexplored until recently, when our group investigated their use in the remote oxygenation of inert cycloalkane C–H bonds,^25^ which showed promise for overcoming similar difficulties. When investigating different counteranion variants of this iodine reagent (entries 5–7), specifically BF_4_^−^, ClO_4_^−^ and SbF_6_^−^, we were pleased to observe a successful suppression of significant side reactions in all cases, although this was accompanied by a diminished mass balance. Particularly noteworthy was the achievement of a satisfactory reaction yield of 51% with the hexafluoroantimonate salt (entry 7). Continued investigations involving alternative solvents led to either the near-exclusive formation of the elimination product (2aa) or the desired product 2a accompanied by large amounts of hydrolysed starting material 2ab (entries 8–9), highlighting the pronounced sensitivity of the reaction to even slight changes to the conditions. The key insight in our optimisation came when we hypothesised that both iodine(iii) atoms in VI could exert oxidising properties. Thus, the effective presence of two equivalents of the oxidant, as encountered when VI is applied in a ratio of 1 : 1 (oxidant:substate), might provoke competing deleterious reaction pathways. Indeed, the application of only 0.5 equiv. of VI—amounting to a full equivalent of iodine(iii) owing to its dimeric nature—gave the desired product in an improved yield of 60% and with only negligible levels of side-product formation (entry 10).^30^

**Scheme 3:**
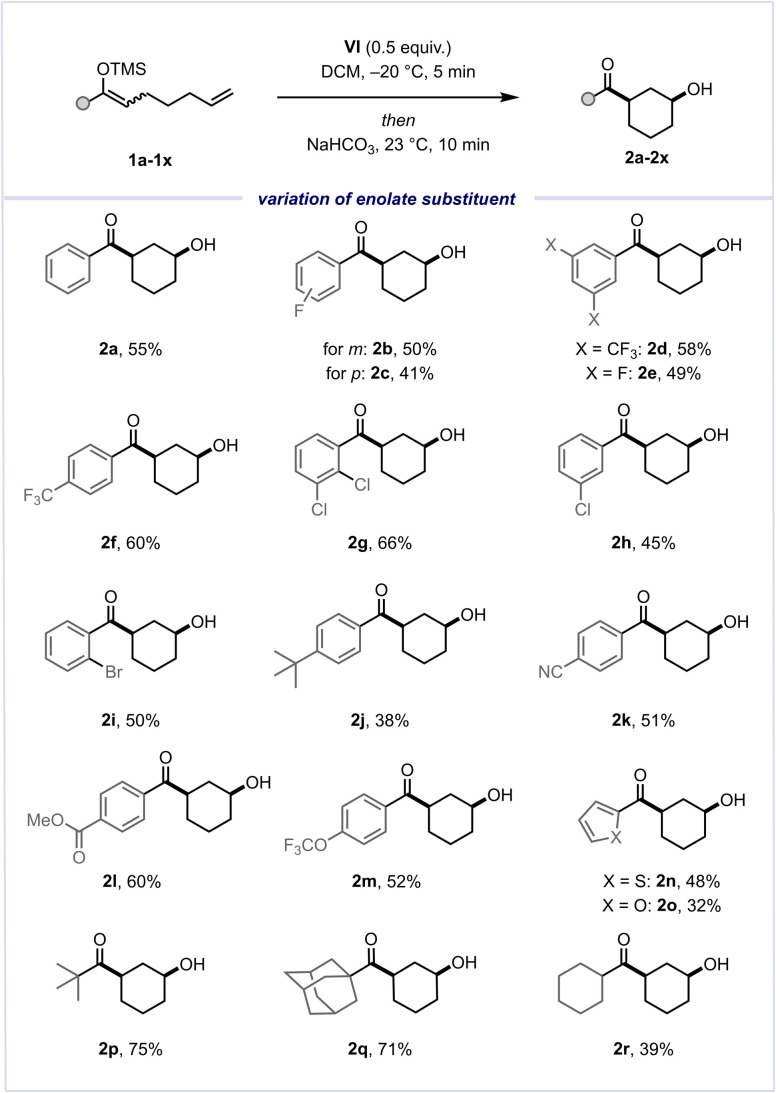
Reaction scope varying the silyl enol ether substituent.

**Scheme 4:**
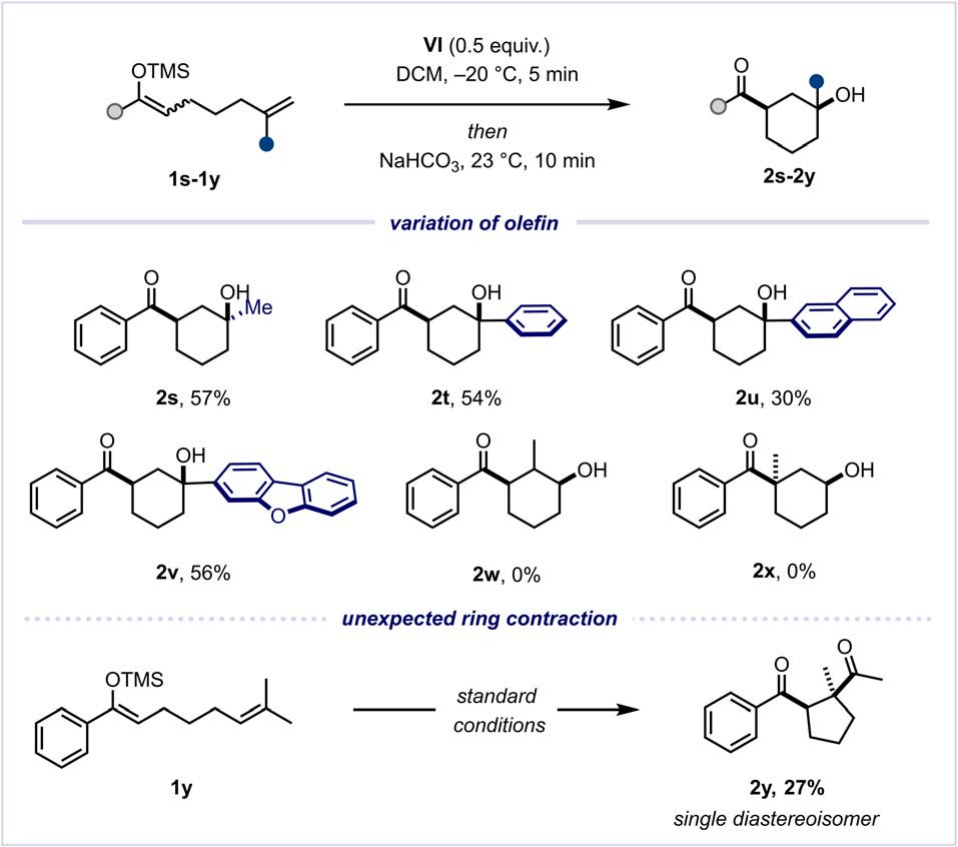
Reaction scope of different olefins.

With optimised conditions in hand, we initially explored the effects of varying substitution on the aryl substituent of the silyl enol ether (Scheme 3). Encouragingly, we observed excellent tolerance towards diverse electron-withdrawing substituents, encompassing fluorine atoms and CF_3_ groups at different positions (2b–2f). Furthermore, substrates containing other halogen atoms, such as Cl or Br (2g–2i), and a *t*-Bu group (2j), also showed similarly favourable outcomes. Gratifyingly, the reaction exhibited broad functional group tolerance, encompassing nitrile (2k), ester (2l), and ether (2m) moieties, as well as heterocycles such as thiophene (2n) and furan (2o). Fully alkyl-substituted silyl enol ethers performed slightly better than the majority of aryl-substituted substrates, with yields of 75% (2p) and 71% (2q) for the desired *cis*-configured alcohol products, as a result of an observed reduced tendency to undergo hydrolysis of the starting material. Additionally, an alkyl-substituted silyl enol ether bearing an enolisable position was evaluated, delivering the product in 39% yield (2r). These promising findings motivated us to explore the olefinic component of this cyclisation reaction (Scheme 4).

We were intrigued to discover that 1,1-disubstituted olefins yielded the desired tertiary alcohol products efficiently, once again furnishing the desired *cis*-stereoisomers (2s–2v) exclusively. However, upon investigating 1,2-disubstituted olefins and α-substituted silyl enol ethers, we did not observe the desired alcohol products (2w, 2x).^31^ To our surprise, upon scrutiny of a terminal gem-dimethyl-substituted olefin (1y), instead of the anticipated alcohol, we observed the formation of a single diastereomer of 1,4-diketone 2y. This outcome likely stems from a complex domino sequence of Umpolung-cyclisation, 1,2-methyl shift, elimination and ultimate ring contraction (see the ESI† for a proposed mechanism with a further experimental study).

**Scheme 5:**
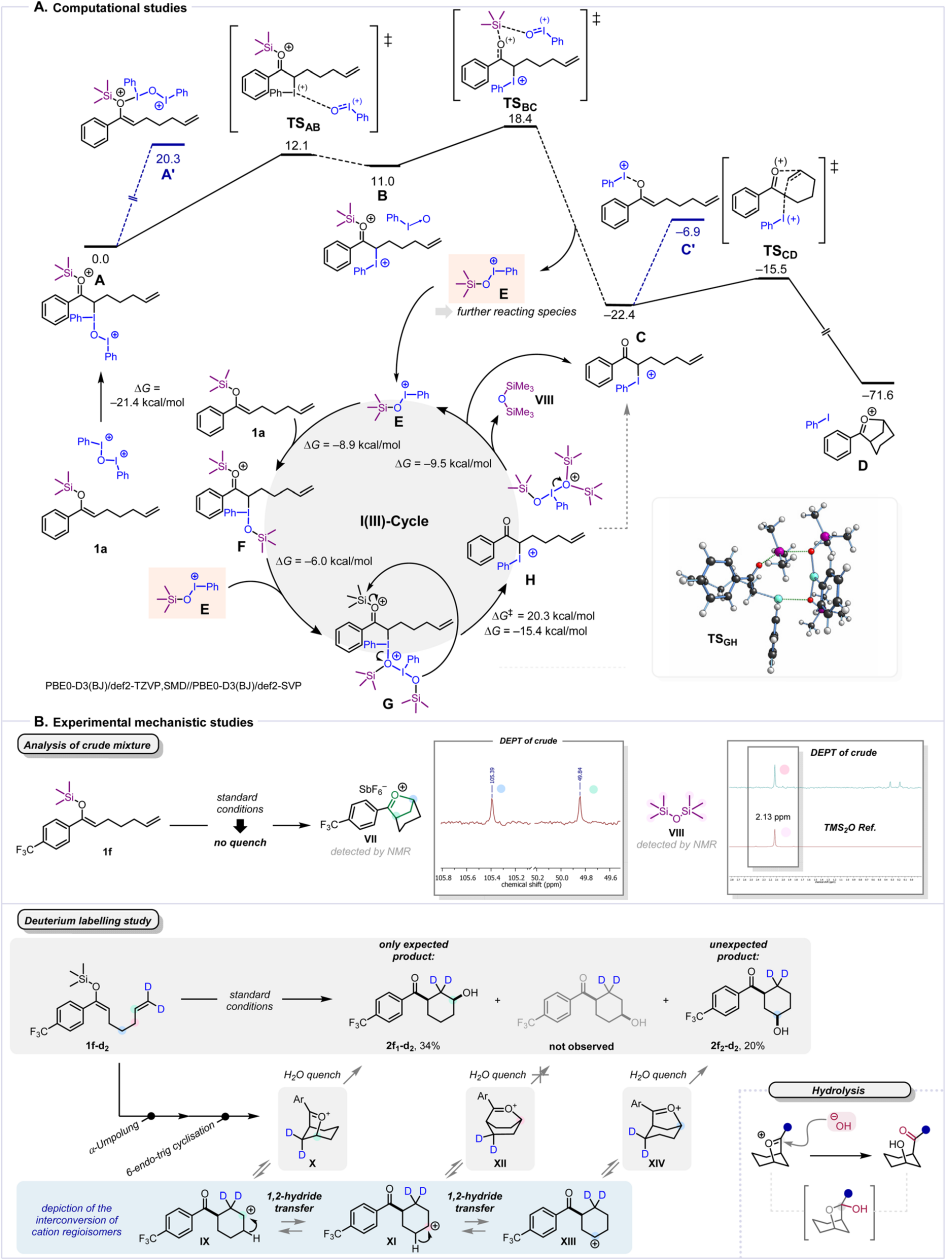
Mechanistic Studies. (A) Computed Gibbs free energy profiles for the iodine-mediated formation of the key oxocarbenium intermediate. Relative Gibbs free energies are presented in kcal mol^−1^ (298 K). The reactant complex A is used as the reference (0.0 kcal mol^−1^). (B) Experimental studies based on crude material and deuterium labelling.

Next, we set out to explore the reaction mechanism in more detail. To this end, a computational investigation at the PBE0-D3(BJ)/def2-TZVP,SMD(DCM)//PBE0-D3(BJ)/def2-SVP^32–38^ level of theory was conducted to shed light on the role of hypervalent iodine, with a focus on understanding how, following initial reaction with a silyl enol ether, the second equivalent of iodine(iii) contained within VI—essentially iodosobenzene, initially used as a leaving group—is able to engage in product formation despite not being activated for nucleophilic attack (Scheme 5A). The interaction of O(IPh)_2_^2+^, a truncated version of VI, from which the counteranion was removed due to negligible influence, with the double bond of 1a was found to initially lead to the adduct A, with I–C bond formation. An alternative interaction of O(IPh)_2_^2+^ with the oxygen of the 1a, leading to the adduct intermediate A′, as has been described in previous work,^8^ was also investigated. However, the high thermodynamic instability of this species (Δ*G*(A→A′) = 20.3 kcal mol^−1^) strongly suggests that I–O interactions are not favourable, particularly prior to loss of the TMS group. Adduct A subsequently evolves to B through dissociation of the I_IPh_–O bond, releasing an iodosobenzene fragment (with a low activation barrier (Δ*G*^‡^(A → B) = 12.1 kcal mol^−1^). Although this step is endergonic (Δ*G*(A → B) = 11.0 kcal mol^−1^), the formed iodosobenzene fragment is able to promote a highly exergonic TMS^+^ abstraction (Δ*G*(B → C) = −33.4 kcal mol^−1^), yielding intermediate C and the silylated iodine species E.

It is worth mentioning that, in intermediate A, the iodine presents a T-shape geometry,^7^ in which the silyl enol ether 1a and the OIPh^+^ fragment reside *anti* to each other.

Due to this geometric constraint, no direct pathway for intramolecular TMS^+^ abstraction from A was found, justifying the presented two-step deprotection process (steps A → B → C), which occurs with an overall barrier of 18.4 kcal mol^−1^, obtained from A to TS_BC_. With the abstraction of TMS^+^ complete, the possibility to form an adduct of the IPh^+^ fragment with the oxygen atom, potentially leading to intermediate C′, was investigated. Once more, however, calculations showed the I_Ph_–O interaction to be thermodynamically unfavourable (Δ*G*(C → C′) = 15.5 kcal mol^−1^). Finally, intermediate C was found to readily undergo a cyclisation step (Δ*G*^‡^(C → D) = 6.9 kcal mol^−1^) which takes place in S_N_2-type fashion, where C–C bond formation and the cleavage of the C–I_Ph_ bond occur concertedly. This step is highly exergonic (Δ*G*(C → D) = −49.2 kcal mol^−1^), yielding structure D, composed of iodobenzene and, notably, a 5-membered oxocarbenium intermediate, which is hydrolysed to the observed product 2a during workup. The high thermodynamic stability of D reflects the driving force exerted by the reduction of the hypervalent iodine reagent to iodobenzene. With the reaction found capable of using both equivalents of iodine(iii) contained in IV, additional considerations with regard to complementary processes were warranted.

Both the calculations detailed above and general synthetic logic suggest that each linear sequence of steps (A → B → C → D) requires the consumption one equivalent of the OIPh_2_^2+^ reagent. In this linear sequence, however, only one of the two iodine(iii) atoms of O(IPh)_2_^2+^ is converted into iodobenzene, while the other is released as TMSOIPh^+^ (E). We found that this species is capable of itself interacting with another molecule of the substrate, generating an intermediate (F) of increased stability (Δ*G*(E → F) = −8.9 kcal mol^−1^), as a result of C–I bond formation. Once more, due to the spatial constraint of the T-shape geometry of the iodine centre, intramolecular abstraction of the TMS group cannot occur. As a result, F interacts with a second equivalent of E to form dicationic intermediate G (Δ*G*(F → G) = −6.0 kcal mol^−1^). The thus added rotational degrees of freedom allow this intermediate to then undergo an intramolecular TMS abstraction step through a rearrangement in which the I–O_IPhOTMS_ and O–Si bonds cleave and a new O–Si bond is formed. The activation barrier of this step is slightly higher than the TMS abstraction in the linear sequence (Δ*G*^‡^(G → H) = 20.3 kcal mol^−1^, compared to Δ*G*^‡^(A → C) = 18.4 kcal mol^−1^), but the formation of intermediate H is exergonic (Δ*G*(G → H) = −15.4 kcal mol^−1^). This intermediate compound group (H) consists of a ketone adduct equivalent to intermediate C, and a hypervalent iodine adduct of TMSOIPh^+^ with (TMS)_2_O. An exergonic I–O bond cleavage from the iodine species (Δ*G*(H → E) = −9.5 kcal mol^−1^) regenerates the iodine species E and releases (TMS)_2_O, which was additionally detected experimentally (*vide infra*, Scheme 5B). It is worth mentioning that each iodine(iii) cycle requires the participation of two iodine species E to convert one equivalent substrate into the intermediate C, regenerating only one equivalent of E. Therefore, although the system is not catalytic, this secondary mechanism for the formation of the product (steps 1a + E → F → G → H → C → D) nicely accounts for the unusual stoichiometry of VI (0.5 equivalents) used in the reaction. Calculations for this process are therefore in line with the experimental observation, which shows that species E, when formed *in situ*, can promote the Umpolung-cyclisation (Scheme 2, entry 2).^39^

As computational analysis of the described process postulated the formation of an oxocarbenium ion (D), we further aimed to validate the presence of such a species in the reaction mixture prior to hydrolytic quench. Pleasingly (Scheme 5B), analysis of the ^13^C NMR (DEPT) of the crude reaction mixture allowed us to identify the oxocarbenium species VII, distinguished by the chemical shifts of two characteristic carbon atoms.^40^ Upon closer inspection of the ^13^C NMR spectrum of the crude reaction mixture, we also detected the presence of (TMS)_2_O (VIII), further corroborating our computational results.

The evidence for intermediate formation of a discrete cation D, and our experience with related species,^24^ prompted us to investigate the potential equilibration of this species through hydride shifts (Scheme 5B). Thus, we prepared the silyl enol ether 1f-d_2_, bearing deuterium atoms on the terminal position of the alkene. Following analysis of the crude reaction mixture, we did not exclusively observe the anticipated product 2f_1_-d_2_, but also the regioisomeric *cis*-configured cyclohexanol 2f_2_-d_2_, in which the deuterium atoms and the alcohol reside in a 1,4-relationship to one another. This observation indeed indicates the occurrence of a sequence of hydride shifts, specifically two sequential hydride shift events, along the cyclohexane ring. Notably, while the initial hydride shift must inevitably form carbocation XI, transient stabilisation of this species is only possible when the cyclohexane adopts a boat conformation (XII). Thus, this less stable intermediate appears to rapidly convert to a more favoured oxocarbenium ion allowing a chair conformation (XIV). As, starting from XI, the necessary hydride shift can—with equal probability—occur in either direction (XI → IX or XI → XIII)—formation of 2f_2_-d_2_ can be readily explained.

## Conclusions

In conclusion, we have described a study on 6-*endo-trig* cyclisations on unbiased systems initiated by ketone Umpolung. Key to the success of these reactions was the identification of a previously underexplored class of hypervalent iodine reagents (VI), which provided access to the desired *cis*-configured 1,3-disubstituted cyclohexanols with excellent diastereo- and regioselectivity and accommodating a wide variety of substrates. The unexpected activation of the second equivalent of iodine(iii) contained in VI was explained through in-depth computational and experimental mechanistic analysis. We believe that the employment of reagent VI in a new context, combined with the involvement of an oxocarbenium species, will advance the development of new synthetic methods leveraging ketone Umpolung.

## Author contributions

N. M. conceptualised the work. G. I. and P. S. conducted experiments. R. M. and B. M. performed DFT calculations. The manuscript was written through contributions of all authors. D. K., B. M. and N. M. were involved in manuscript editing, finalising and overall supervision of the project. N. M. and L. G. secured funding and supervised the work.

## Conflicts of interest

There are no conflicts to declare.

## Supplementary Material

SC-016-D5SC01085C-s001

## Data Availability

The data that support the findings of this study are available in the ESI† of this article.
