# Supplementary material for: Diastereoselective Umpolung cyclisation of ketones promoted by hypervalent iodine
Source: Chem Sci. 2025 May 13;16(24):10944–50. doi: 10.1039/d5sc01085c (PMC12086710; doi:10.1039/d5sc01085c)
Supplement: SC-016-D5SC01085C-s001 [file SC-016-D5SC01085C-s001.pdf]

Supplementary Information (SI) for Chemical Science.
This journal is © The Royal Society of Chemistry 2025

Diastereoselective Umpolung cyclisation of ketones promoted by hypervalent iodine

Giulia Iannelli,[a,†] Philipp Spieß,[a,†] Ricardo Meyrelles,[a,b] Daniel Kaiser,[a] Boris Maryasin,[a,b] Leticia González[b] and Nuno Maulide*[a]

[a]Institute of Organic Chemistry, University of Vienna, Währinger Straße 38, 1090 Vienna, Austria

[b]Institute of Theoretical Chemistry, University of Vienna, Währinger Straße 17, 1090 Vienna, Austria

*Corresponding author; nuno.maulide@univie.ac.at

Contents

1. GENERAL INFORMATION ... 3

2. OPTIMIZATION OF THE REACTION CONDITIONS ... 4

3. EXPERIMENTAL ... 6

3.1. PREPARATION OF IODONIUM REAGENTS ... 6

3.2. GENERAL PROCEDURES ... 10

3.2.1. General procedure 1 (GP1): Preparation of Weinreb amides ... 10

3.2.2. General procedure 2 (GP2): Weinreb amides for the preparation of 1,1-disubstituted olefins ... 10

3.2.3. General procedure 3 (GP3): Preparation of ketones from Weinreb amides ... 11

3.2.4. General procedure 4 (GP4): Formation of silyl enol ethers from ketones ... 12

3.2.5. General procedure 5 (GP5): Diasteroselective Umpolung cyclization ... 12

3.3. CHARACTERIZATION OF STARTING MATERIALS ... 13

3.3.1. Characterization of Weinreb Amides ... 13

3.3.2. Characterization of Ketones ... 20

3.3.3. Characterization of Silyl Enol Ethers ... 30

3.4. CHARACTERIZATION OF CYCLIZED KETONES ... 42

3.5. FORMATION OF AN UNEXPECTED RING-CONTRACTION PRODUCT ... 52

3.5.1 Proposed Mechanism and Experimental Hints ... 52

3.6. UNSUCCESSFUL EXAMPLES OF THE UMPOLUNG CYCLIZATION ... 54

4. EXPERIMENTAL MECHANISTIC STUDIES ... 55

4.1. DETECTION OF OXOCARBENIUM **VII** AND TMS_2O (**VIII**) ... 55

4.2. UMPOLUNG CYCLIZATION WITH **1F-D$_2$** ... 57

5. DFT CALCULATIONS ... 63

5.1. THERMODYNAMIC COMPARISON OF I–C AND I–O INTERACTIONS ... 63

5.2. THE INFLUENCE OF THE COUNTERION ON THE REACTION MECHANISM ... 64

5.3. XYZ STRUCTURES ... 65

6. NMR SPECTRA ... 90

7 REFERENCES ... 187

1. General Information

Unless otherwise stated, all glassware was flame-dried before use and all reactions were performed under an atmosphere of argon. All solvents were distilled from appropriate drying agents prior to use. All reagents were used as received from commercial suppliers unless otherwise stated. Reaction progress was monitored by thin layer chromatography (TLC) performed on aluminum plates coated with silica gel F254 with 0.2 mm thickness. Chromatograms were visualized by fluorescence quenching with UV light at 254 nm or by staining using potassium permanganate. Flash column chromatography was performed using silica gel 60 (230-400 mesh, Merck and co.). Neat infrared spectra were recorded using a Perkin-Elmer Spectrum 100 FT-IR spectrometer. Wavenumbers (ν_{max}) are reported in cm^{-1}. Mass spectra were obtained using a Bruker maXis UHR-TOF (QQ-TOF) spectrometer, using electrospray ionization (ESI) or an Agilent 7200B GC/Q-TOF spectrometer, using electron ionization (EI). All ^{1}H NMR and ^{13}C NMR spectra were recorded using a Bruker AV-400 or AV-600 spectrometer at 300 K. Chemical shifts are given in parts per million (ppm, δ), referenced to the solvent peak of $CDCl_3$ defined at δ = 7.26 ppm (^{1}H NMR) and δ = 77.16 (^{13}C NMR). Coupling constants are quoted in Hz (*J*). ^{1}H NMR splitting patterns are designated as singlet (s), doublet (d), triplet (t), quartet (q), heptet (hept), as they appeared in the spectrum. If the appearance of a signal differs from the expected splitting pattern, the observed pattern is designated as apparent (app). Splitting patterns that could not be interpreted or easily visualized are designated as multiplet (m) or broad (br).

2. Optimization of the Reaction Conditions

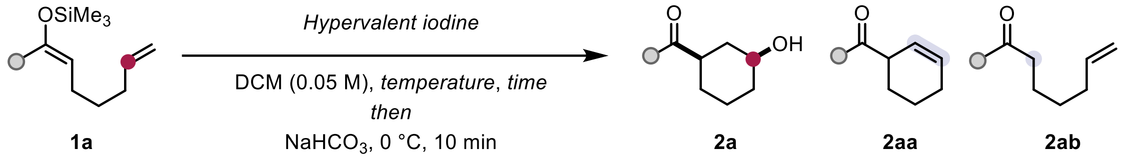

Entry	Temperature [°C]	Iodine	Solvent	Stoichiometry	Time	NMR yield %[a]		
						2a	2aa	2ab
1	-78	$PhIO/BF_3Et_2O$ MsOH	DCM	1.2 eq.	5 min	n.d.	n.d.	n.d.
2	-78	DIB/TMSOTf	DCM	1.2 eq.	60 min	12	15	<10
3	-78	DIB/TMSOTf	DCM	1.2 eq.	5 min	20	12	<10
4	-78	PIFA/TMSOTf	DCM	1.2 eq.	5 min	31	18	26
5	-15	$(PhI)_2O(BF_4)_2$	DCM	1.0 eq.	10 min	n.d.	<10	<10
6	-15	$(PhI)_2O(ClO_4)_2$	DCM	1.0 eq.	10 min	36	<10	<10
7	-15	$(PhI)_2O(SbF_6)_2$	DCM	1.0 eq.	10 min	51	<10	-
8	-78	PhIO/TMSOTf	DCM	1.2 eq.	10 min	35	20	25
9	-78	Koser's reagent/ BF_3Et_2O	DCM	1.2 eq.	10 min	20	35	n.d.
10	-78	$DIB/HSbF_6$	DCM	1.2 eq.	5 min	35	33	13
11	-78	$PhIO/HSbF_6$	DCM	1.2 eq.	5 min	25	25	12
12	-40	$(PhI)_2O(SbF_6)_2$	DCM	1.0 eq.	10 min	45	<10	<10
13	0	$(PhI)_2O(SbF_6)_2$	DCM	1.0 eq.	10 min	40	<10	<10
14	25	$(PhI)_2O(SbF_6)_2$	DCM	1.0 eq.	10 min	27	<10	10
15	-15	$(PhI)_2O(SbF_6)_2$	DCE	1.0 eq.	10 min	36	n.d.	60
16	-15	$(PhI)_2O(SbF_6)_2$	ACN	1.0 eq.	10 min	n.d.	50	<10
17	-15	$(PhI)_2O(SbF_6)_2$	Difluorobenzene	1.0 eq.	10 min	26	20	45
18	-15	$(PhI)_2O(SbF_6)_2$	Cyclohexane	1.0 eq.	10 min	11	<10	32
19	-15	$(PhI)_2O(SbF_6)_2$	THF	1.0 eq.	10 min	n.d.	n.d.	99
20	-15	$(PhI)_2O(SbF_6)_2$	DCM	0.25 eq.	10 min	35	22	35
**21**	**-15**	**$(PhI)_2O(SbF_6)_2$**	**DCM**	**0.5 eq.**	**10 min**	**60**	**<10**	**<10**
22	-15	$(PhI)_2O(SbF_6)_2$	DCM	0.6 eq.	10 min	65	<10	<10
23	-15	$(PhI)_2O(SbF_6)_2$	DCM	2.0 eq.	10 min	50	<10	<10

24	-15	$(PhI)_2O(SbF_6)_2$	DCM	0.5 eq.	2 min	44	<10	<10
25	-15	$(PhI)_2O(SbF_6)_2$	DCM	0.5 eq.	30 min	41	25	16
26	-15	$(PhI)_2O(SbF_6)_2$	DCM	0.5 eq.	90 min	33	21	15

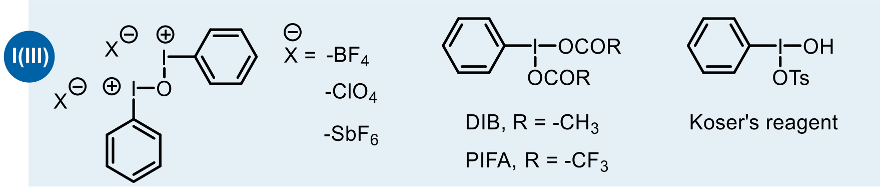

Reactions were performed on a 0.1 mmol scale using the SEE **1a** (1.0 eq.), hypervalent iodine in CH_2Cl_2 at the given temperature. [a]Determined using mesitylene as internal standard.

3. Experimental

3.1. Preparation of iodonium reagents

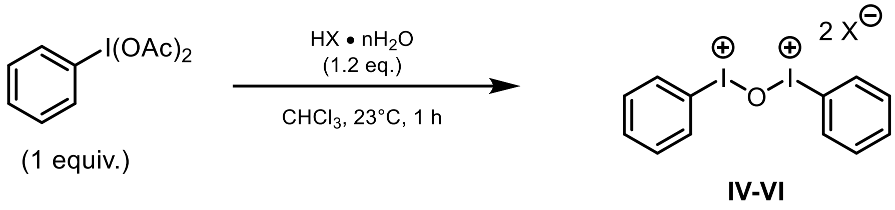

Compounds were prepared using a modified version of a reported procedure.[1]

To a cooled solution (0 °C) of the corresponding diacetoxyiodoarene (1.0 eq.) in chloroform ($CHCl_3$, 2 M), the acid (1.2 eq.) was slowly added, and the resulting mixture was allowed to warm to 23 °C. After 1 h, water (1.0 mL for 1.00 mmol of starting diacetoxyiodoarene) was added, and the mixture was cooled to 0 °C. After 3 h at 0 °C, a precipitate had formed and the mixture was filtered. The precipitate was washed with $CHCl_3$, water and pentane. The obtained solid was crushed to a powder and the washing steps were repeated in the same order twice. The resulting solid was dried under high vacuum (10^{-2}–10^{-3} mbar) for at least 5 h to afford the dicationic iodonium reagent as a bright-yellow powder.

$(PhI)_2O(BF_4)_2$ – Oxybis(phenyliodonium) bis[tetrafluoroborate(V)] - IV

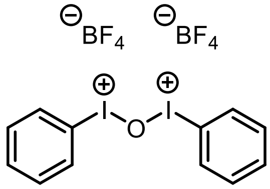

Following the general procedure (10.0 mmol scale), diacetoxyiodobenzene (3.3 g, 10.0 mmol, 1.0 eq.) and HBF_4 (2.09 mL, 16.0 mmol, 1.6 eq. 48 w% in H_2O) afforded **IV** (845 mg, 1.41 mmol, 28%) as a bright-yellow powder.

All NMR data were found to be in accordance with the literature.[1]

Carbon spectrum could not be recorded as product decomposes readily in the presence of DMSO.

^{1}H NMR (400 MHz, 2:8 DMSO-*d*$_6$/CDCl$_3$): δ 7.76 – 7.55 (m, 4H), 7.44 – 7.33 (m, 2H), 7.32 – 7.12 (m, 4H) ppm.

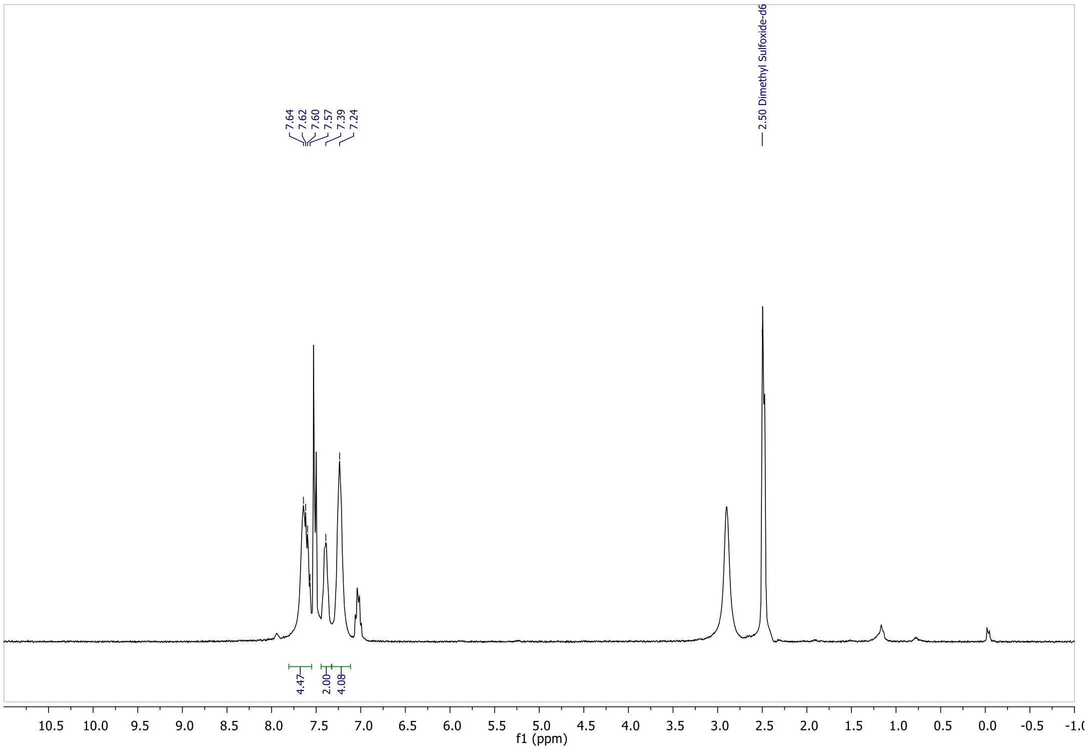

$(PhI)_2O(ClO_4)_2$ – Oxybis(phenyliodonium) bis(perchlorate) - V

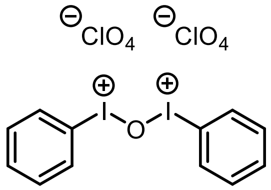

Following the general procedure (10.0 mmol scale), diacetoxyiodobenzene (3.30 g, 10.0 mmol, 1.00 eq.) and perchloric acid (1.60 mL, 12.0 mmol, 1.20 eq, 70 w% in H_2O) afforded **V** (1.71 g, 2.75 mmol, 55%) as a yellow solid.

Carbon spectrum could not be recorded as product decomposes readily in the presence of DMSO.

^{1}H NMR (400 MHz, 2:8 DMSO-d_6/$CDCl_3$): δ 7.73 – 4.56 (m, 4H), 7.45 – 7.35 (m, 2 H), 7.30 – 7.16 (m, 4H) ppm.

IR (neat) ν_{max}: 3054, 1470, 1442, 1095, 1057, 991, 925, 729, 677, 620, 570, 412 cm^{-1}.

HRMS (ESI$^+$): exact mass calculated for $[M - 2ClO_4^- + H_2O]^{2+}$ ($C_{12}H_{12}I_2O_2^{2+}$) requires *m/z* 220.9458, found *m/z* 220.9460.

HRMS (ESI$^-$): exact mass calculated for ClO_4^- requires *m/z* 98.9491, found *m/z* 98.9487; exact mass calculated for $[M+ClO_4]^-$ ($C_{12}H_{10}Cl_3I_2O_{13}^-$) requires *m/z* 720.7281, found *m/z* 720.7296.

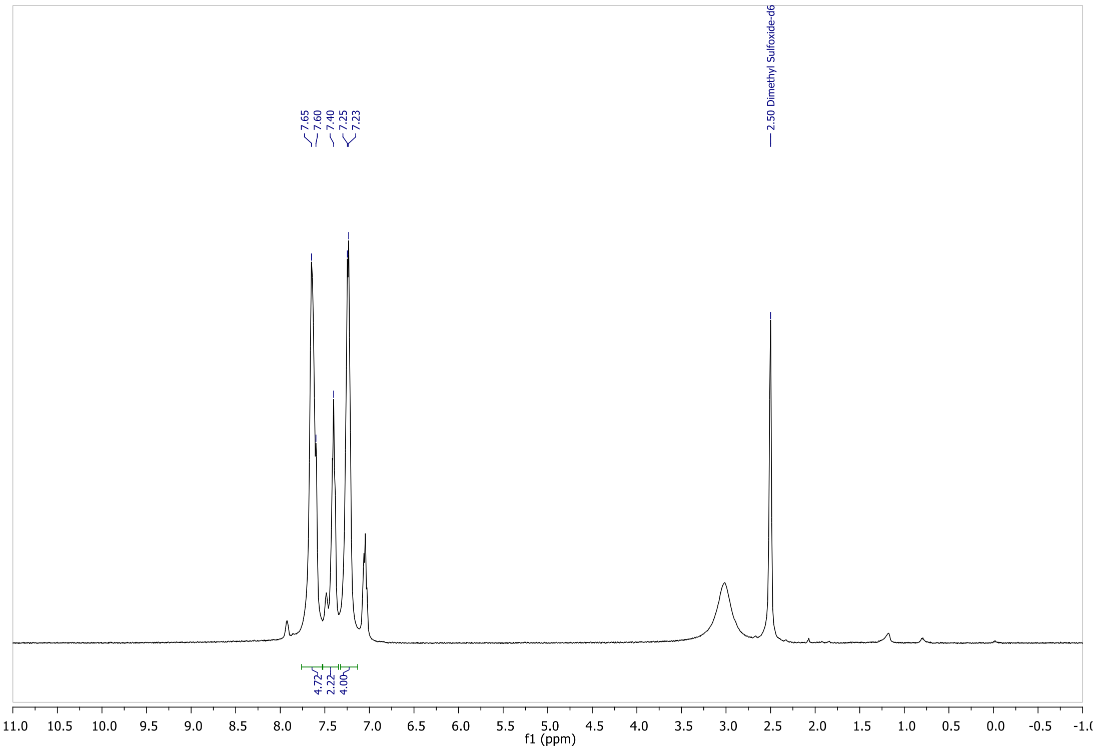

$(PhI)_2O(SbF_6)_2$ – Oxybis(phenyliodonium) bis[hexafluorostilbate(V)] - VI

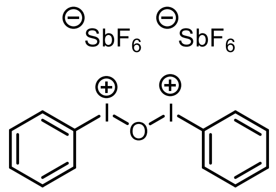

Following the general procedure (50.0 mmol scale), diacetoxyiodobenzene (16.4 g, 50.0 mmol, 1.0 eq.) and $HSbF_6 \cdot 6H_2O$ (22.0 g, 61.0 mmol, 1.2 eq.) afforded **VI** (12.7 g, 14.2 mmol, 57%) as a bright-yellow powder.

All NMR data were found to be in accordance with the literature.[1]

Carbon spectrum could not be recorded, as product decomposes readily in the presence of DMSO.

^{1}H NMR (400 MHz, 2:8 DMSO-*d*$_6$/$CDCl_3$): δ 7.74 – 7.53 (m, 4H), 7.47 – 7.35 (m, 2H), 7.32 – 7.14 (m, 4H) ppm.

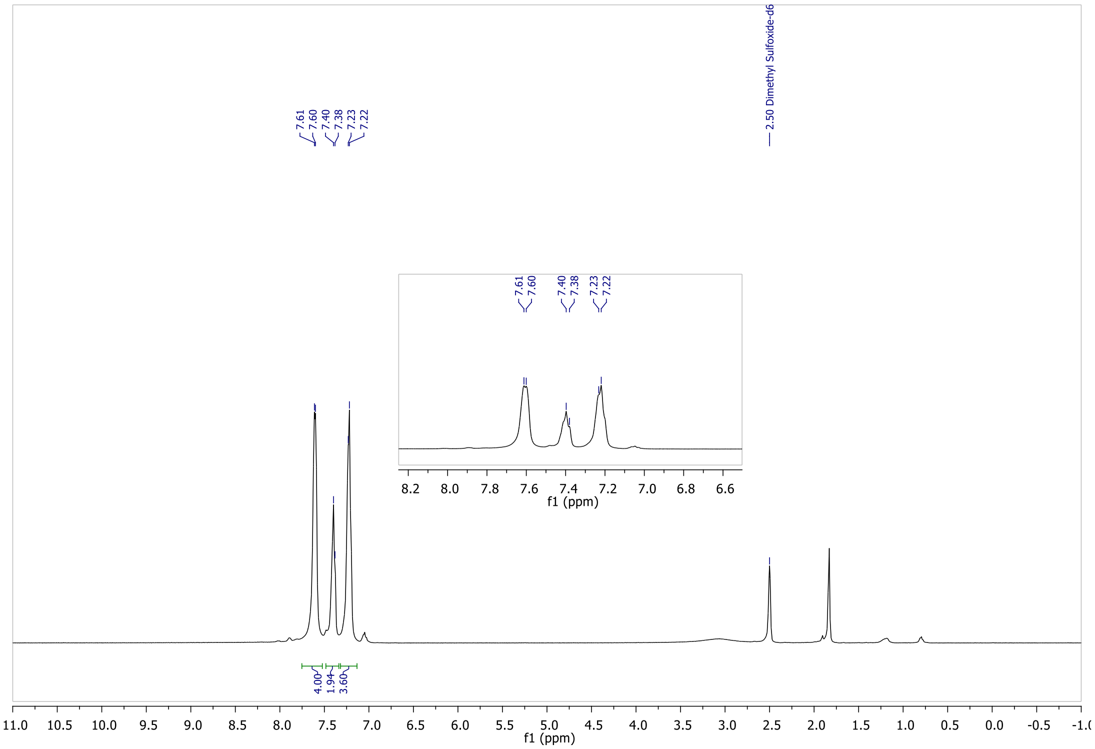

3.2. General procedures

3.2.1. General procedure 1 (GP1): Preparation of Weinreb amides

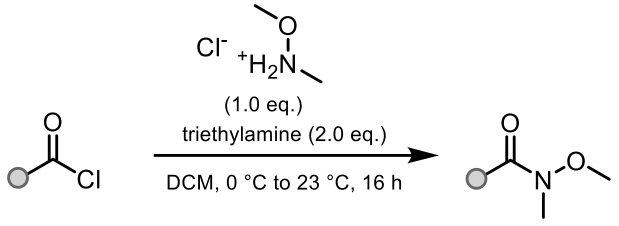

3a-3q

Triethylamine (2.0 eq.) was slowly added at 0 °C to a stirred suspension of *N,O*-dimethylhydroxylamine hydrochloride (1.0 eq.) in DCM (0.1 M). The corresponding acyl chloride (1.0 eq.) was then slowly added to the solution. The resulting mixture was allowed to warm to room temperature over 16 h before stopping the reaction by addition of a saturated aqueous solution of $NaHCO_3$. The two layers were separated, and the organic phase was washed with 1 M HCl and brine. The organic phase was dried over sodium sulfate before being concentrated in vacuo. The obtained products were used without further purification.

3.2.2. General procedure 2 (GP2): Weinreb amides for the preparation of 1,1-disubstituted olefins

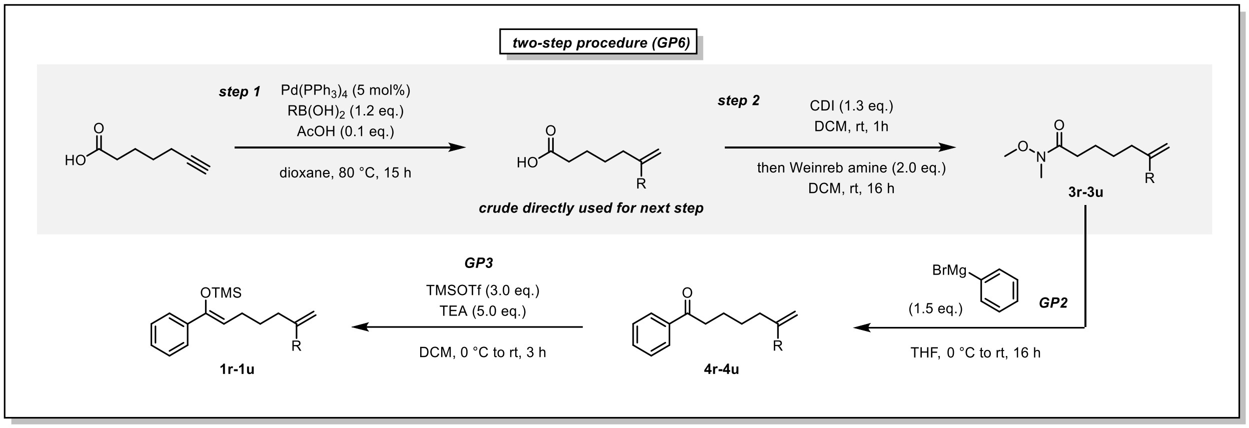

Step 1: A modified procedure for hydroarylation was used.[2] Under argon, a flame-dried Schlenk flask was loaded with $Pd(Ph_3)_4$ (0.05 eq.), the corresponding boronic acid (1.2 eq.) and 6-heptynoic acid (1.0 eq.). Lastly, dioxane (0.3 M) and acetic acid (0.1 eq.) were added. The mixture was heated at 80 °C for 15 h. After cooling to room temperature, dioxane was removed under reduced pressure. Then, the crude material was dissolved in EtOAc and passed through a short pad of Celite. The solution was concentrated and the crude material obtained was immediately used for the next step without further purification.

Step 2: The crude material was dissolved in anhydrous DCM (0.3 M) and 1,1'-carbonyldiimidazole (1.3 eq.) was added in portions. Strong gas evolution was observed immediately after addition and the solution was subsequently stirred at room temperature for 1 h. Then, *N*,*O*-dimethylhydroxylamine hydrochloride (2.0 eq.) was added and the resulting suspension was stirred at room temperature for 16 h. To stop the reaction, 1M HCl was added and the aqueous phase was extracted with DCM (3 x 10 mL). The organic phase was washed with a saturated solution of $NaHCO_3$ (3 x 10 mL) and then with brine (1 x 10 mL). The organic phase was dried over $MgSO_4$ and the solvent was removed under reduced pressure. The crude material was purified by column chromatography (heptane/EtOAc = 97:3 to 90:10), yielding the desired Weinreb amides.

3.2.3. General procedure 3 (GP3): Preparation of ketones from Weinreb amides

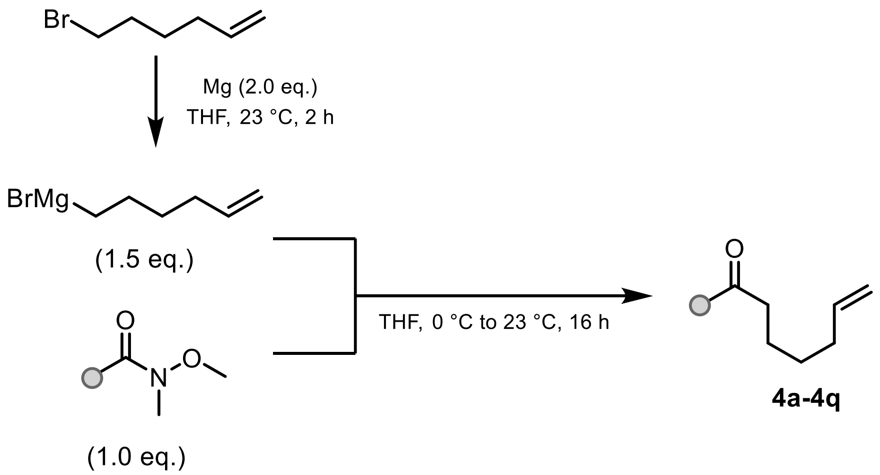

Step 1: Preparation of Grignard

A THF solution of the bromide was slowly added to a suspension of magnesium turnings (2.0 eq.) in THF (total targeted molarity 1 M). After complete addition of the bromide solution, the mixture was stirred for a further 2 h until most of the magnesium had dissolved. The concentration of the Grignard solution was determined by titration with I_2 before use.

Step 2: Preparation of ketones

In a separate flask, the corresponding Weinreb amide was dissolved in THF (0.4 M) and cooled to 0 °C. A solution of Grignard reagent was then added dropwise. The resulting mixture was allowed to warm to room temperature over 16 h before stopping the reaction by addition of a saturated aqueous solution of $NaHCO_3$ (10 mL). The aqueous layer was extracted with DCM (3 x 10 mL) and the combined organic layers

were dried over Na_2SO_4 and filtered. Evaporation of the solvent under reduced pressure yielded the crude ketone, which was purified by column chromatography (heptane/EtOAc 98:2 to 60:40).

3.2.4. General procedure 4 (GP4): Formation of silyl enol ethers from ketones

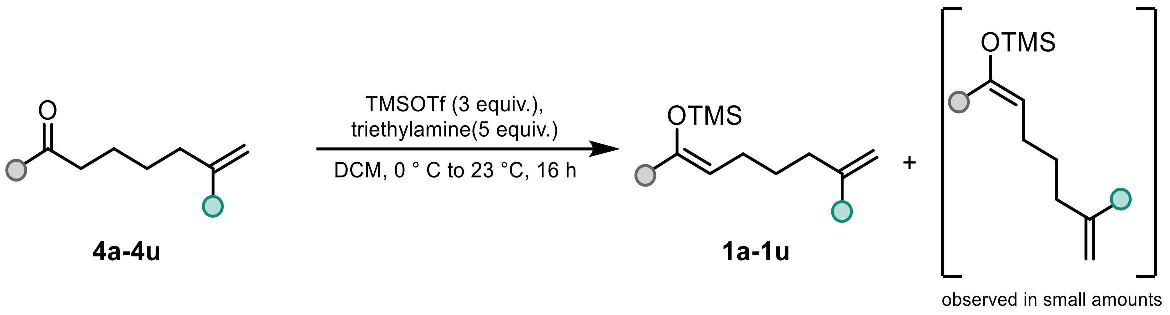

A vial was loaded with the corresponding ketone (1.0 eq.), triethylamine (5.0 eq.), and DCM (0.2 M). The solution was cooled to 0 °C and TMSOTf (3.0 eq.) was added dropwise. The resulting mixture was allowed to warm to room temperature and then stirred for 16 h before stopping the reaction by addition of a saturated aqueous solution of NH_4Cl. The aqueous layer was extracted with DCM (3 x 10 mL). The combined organic phases were dried over $MgSO_4$. After evaporation of the organic solvent under reduced pressure, the crude material was further purified by column chromatography (heptane/toluene 95:5).
All products were obtained at greater than 20:1 Z/E ratio (as judged by NMR analysis), unless otherwise indicated.

3.2.5. General procedure 5 (GP5): Diasteroselective Umpolung cyclization

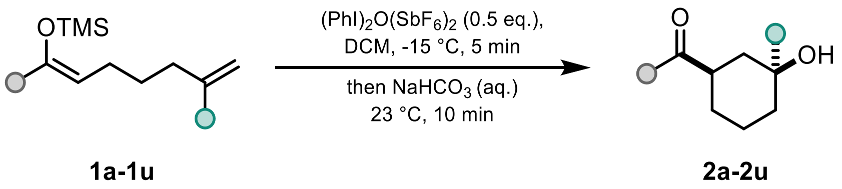

To a vigorously stirred solution of $(PhI)_2O(SbF_6)_2$ (**VI**, 0.5 eq.) in dichloromethane (0.1 M) at -15 °C, a solution of the corresponding silyl enol ether (1.0 eq., 0.1 M in DCM) was added dropwise. The resulting solution was stirred for 5 min at -15 °C before saturated aqueous solution of $NaHCO_3$ was added. The mixture was allowed to warm to room temperature and left stirring for an additional 10 min. The phases were separated and the aqueous phase was extracted once again with CH_2Cl_2. The combined organic phases were washed with brine, dried over $MgSO_4$, filtered and concentrated in vacuo. The resulting crude

material was purified by flash column chromatography on silica gel (heptane/ethyl acetate, 1:1) to afford the desired products.

3.3. Characterization of starting materials

3.3.1. Characterization of Weinreb Amides

N-Methoxy-N-methylbenzamide (3a)

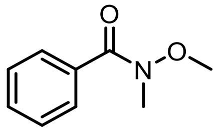

Following GP1 (10.0 mmol scale), the title compound was obtained as a colorless oil (1.50 g, 9.08 mmol, 91%).

All NMR data were in good agreement with the literature.[3]

^{1}H NMR (400 MHz, $CDCl_3$): δ 7.77 – 7.61 (m, 2H), 7.50 – 7.35 (m, 3H), 3.55 (s, 3H), 3.36 (s, 3H) ppm.

3-Fluoro-N-methoxy-N-methylbenzamide (3b)

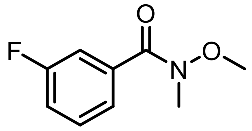

Following GP1 (4.0 mmol scale), the title compound was obtained as a colorless oil (701 mg, 3.83 mmol, 96%).

All NMR data were in good agreement with the literature.[4]

^{1}H NMR (400 MHz, $CDCl_3$): δ 7.49 – 7.44 (m, 1H), 7.41 – 7.33 (m, 2H), 7.14 (tdd, *J* = 8.4, 2.6, 1.0 Hz, 1H), 3.54 (s, 3H), 3.35 (s, 3H) ppm.

4-Fluoro-N-methoxy-N-methylbenzamide (3c)

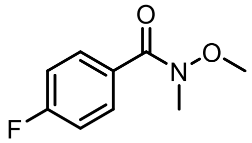

Following GP1 (5.0 mmol scale), the title compound was obtained as a colorless oil (915 mg, 4.99 mmol, 99%).

All NMR data were in good agreement with the literature.[5]

^{1}H NMR (400 MHz, $CDCl_3$): δ 7.75 – 7.68 (m, 2H), 7.10 – 7.02 (m, 2H), 3.52 (s, 3H), 3.34 (s, 3H) ppm.

N-Methoxy-N-methyl-3,5-bis(trifluoromethyl)benzamide (3d)

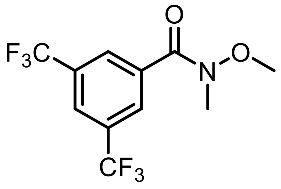

Following GP1 (2.6 mmol scale), the title compound was obtained as a colorless liquid (0.65 g, 2.2 mmol, 83%).

All NMR data were in good agreement with the literature.[6]

^{1}H NMR (400 MHz, $CDCl_3$) δ 8.19 (s, 2H), 7.97 (s, 1H), 3.56 (s, 3H), 3.41 (s, 3H) ppm.

3,5-Difluoro-N-methoxy-N-methylbenzamide (3e)

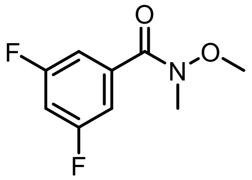

Following GP1 (5.0 mmol scale), the title compound was obtained as a colorless liquid (800 mg, 4.00 mmol, 80%).

All NMR data were in good accordance to the literature.[7]

^{1}H NMR (700 MHz, $CDCl_3$): δ 7.25 – 7.20 (m, 2H), 6.91 (tt, *J* = 8.7, 2.4 Hz, 1H), 3.56 (s, 3H), 3.36 (s, 3H) ppm.

N-Methoxy-N-methyl-4-(trifluoromethyl)benzamide (3f)

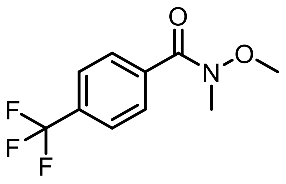

Following GP1 (10.0 mmol scale), the title compound was obtained as a colorless liquid (2.25 g, 9.49 mmol, 95%).

All NMR data were in good accordance to the literature.[8]

^{1}H NMR (400 MHz, CDCl3): δ 7.77 (d, *J* = 7.8 Hz, 2H), 7.65 (d, *J* = 8.0 Hz, 2H), 3.51 (s, 3H), 3.36 (s, 3H) ppm.

3,4-Dichloro-*N*-methoxy-*N*-methylbenzamide (3g)

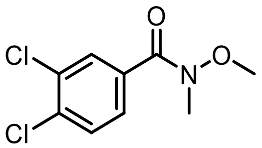

Following GP1 (5.0 mmol scale), the title compound was obtained as a colorless liquid (700 mg, 4.51 mmol, 90%).

All NMR data were in good accordance to the literature.[9]

^{1}H NMR (400 MHz, $CDCl_3$): δ 7.82 (d, *J* = 1.8 Hz, 1H), 7.55 (dt, *J* = 10.1, 5.1 Hz, 1H), 7.51 – 7.44 (m, 1H), 3.54 (s, 3H), 3.36 (s, 3H) ppm.

3-Chloro-*N*-methoxy-*N*-methylbenzamide (3h)

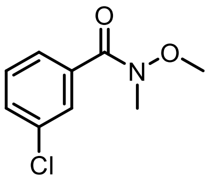

Following GP1 (0.5 mmol scale), the title compound was obtained as a colorless liquid (102 mg, 0.5 mmol, 99%).

All NMR data were in good accordance to the literature.[10]

^{1}H NMR (400 MHz, $CDCl_3$): δ 7.67 (s, 1H), 7.57 (d, *J* = 7.6 Hz, 1H), 7.44 – 7.41 (m, 1H), 7.34 (t, *J* = 7.8 Hz, 1H), 3.55 (s, 3H), 3.36 (s, 3H) ppm.

4-(*tert*-Butyl)-*N*-methoxy-*N*-methylbenzamide (3j)

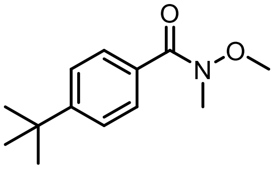

Following GP1 (5.0 mmol scale), the title compound was obtained as a colorless liquid (1100 mg, 5.0 mmol, 99%).

All NMR data were in good accordance to the literature.[11]

^{1}H NMR (400 MHz, $CDCl_3$): δ 7.63 (d, *J* = 8.3 Hz, 2H), 7.41 (d, *J* = 8.3 Hz, 2H), 3.58 (s, 3H), 3.36 (s, 3H), 1.33 (s, 9H) ppm.

4-Cyano-*N*-methoxy-*N*-methylbenzamide (3k)

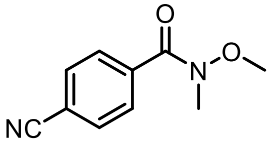

Following GP1 (5.0 mmol scale), the title compound was obtained as a colorless oil (770 mg, 4.06 mmol, 81%).

All NMR data were in good agreement with the literature.[12]

^{1}H NMR (400 MHz, CDCl$_3$): δ 7.80 – 7.75 (m, 2H), 7.73 – 7.69 (m, 2H), 3.52 (s, 3H), 3.39 – 3.35 (m, 3H) ppm.

Methyl 4-(methoxy(methyl)carbamoyl)benzoate (3l)

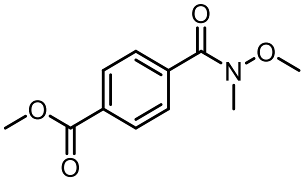

Following GP1 (5.0 mmol scale), the title compound was obtained as a colorless crystals (1.03 g, 4.61 mmol, 92%).

All NMR data were found to be in accordance with the literature.[13]

^{1}H NMR (400 MHz, CDCl$_3$): δ 8.09 – 8.04 (m, 2H), 7.75 – 7.69 (m, 2H), 3.93 (s, 3H), 3.52 (s, 3H), 3.37 (s, 3H) ppm.

***N*-Methoxy-*N*-methyl-4-(trifluoromethoxy)benzamide (3m)**

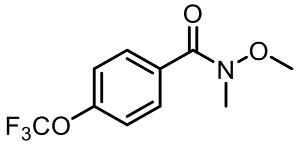

Following GP1 (5.0 mmol scale), the title compound was obtained as a colorless oil (980 mg, 3.93 mmol, 79%).

All NMR data were in good accordance with the literature.[14]

^{1}H NMR (700 MHz, CDCl$_3$): δ 7.77 (d, *J* = 8.8 Hz, 2H), 7.24 (dd, *J* = 8.8, 2H), 3.55 (s, 3H), 3.38 (s, 3H) ppm.

N-Methoxy-N-methylthiophene-2-carboxamide (3n)

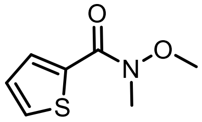

Following GP1 (6.75 mmol), the title compound was obtained as a colorless oil (1.16 g, 6.75 mmol, 100%). All NMR data were in good accordance with the literature.[15]

^{1}H NMR (400 MHz, $CDCl_3$): δ 7.97 (dd, *J* = 3.8, 1.2 Hz, 1H), 7.55 (dd, *J* = 5.0, 1.2 Hz, 1H), 7.11 (dd, *J* = 5.0, 3.9 Hz, 1H), 3.78 (s, 3H), 3.38 (s, 3H) ppm.

N-Methoxy-N-methylfuran-2-carboxamide (3o)

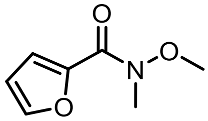

Following GP1 (5.0 mmol scale), the title compound was obtained as a colorless liquid (700 mg, 4.51 mmol, 90%).

All NMR data were in good accordance to the literature.[16]

^{1}H NMR (400 MHz, $CDCl_3$): δ 7.57 (dd, *J* = 1.7, 0.8 Hz, 1H), 7.13 (dd, *J* = 3.5, 0.8 Hz, 1H), 6.49 (dd, *J* = 3.5, 1.7 Hz, 1H), 3.75 (s, 3H), 3.34 (s, 3H) ppm.

N-Methoxy-N-methyladamantane-1-carboxamide (3q)

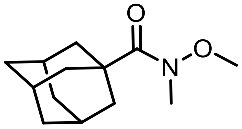

Following GP1 (5.0 mmol scale), the title compound was obtained as a white solid (1.11 g, 5.0 mmol, 99%). All NMR data were in good accordance to the literature.[17]

^{1}H NMR (400 MHz, $CDCl_3$): δ 3.67 (s, 3H), 3.16 (s, 3H), 1.99 (app s, 9H), 1.71 (app s, 6H) ppm.

N-methoxy-N-methylcyclohexanecarboxamide (3r)

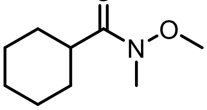

Following GP1 (5.0 mmol scale), the title compound was obtained as a colorless oil (801 mg, 4.68 mmol, 94%).

All NMR data were in good agreement with the literature[18]

^{1}H NMR (400 MHz, $CDCl_3$) δ 3.69 (d, *J* = 0.7 Hz, 1H), 3.17 (s, 1H), 2.67 (t, *J* = 12.0 Hz, 0H), 1.85 – 1.70 (m, 2H), 1.54 – 1.38 (m, 1H), 1.38 – 1.11 (m, 1H) ppm.

***N*-Methoxy-*N*-methyl-6-phenylhept-6-enamide (3t)**

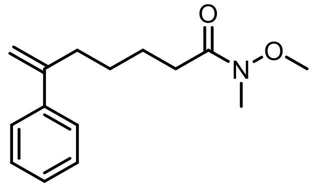

Following GP2 (5 mmol scale), the desired Weinreb amide was obtained as a colorless liquid (782 mg, 3.16 mmol, 63 %).

^{1}H NMR (400 MHz, $CDCl_3$): δ 7.42 – 7.37 (m, 2H), 7.34 – 7.29 (m, 2H), 7.28 – 7.26 (m, 1H), 5.26 (d, *J* = 1.4 Hz, 1H), 5.07 (dd, *J* = 2.6, 1.2 Hz, 1H), 3.65 (s, 3H), 3.16 (s, 3H), 2.58 – 2.51 (m, 2H), 2.41 (t, *J* = 7.5 Hz, 2H), 1.72 – 1.62 (m, 2H), 1.56 – 1.46 (m, 2H) ppm.

^{13}C NMR (101 MHz, $CDCl_3$): δ 174.7, 148.4, 141.4, 128.4 (2C), 127.4, 126.3 (2C), 112.5, 61.3, 35.3, 32.3, 31.9, 28.1, 24.4 ppm.

IR (neat) ν_{max}: 1658, 1460, 1438, 1414, 1382, 994, 891, 859, 819, 750 cm^{-1}.

HRMS (ESI^+): exact mass calculated for $[M+H]^+$ ($C_{15}H_{22}NO_2$) requires *m/z* 248.1645, found *m/z* 248.1643.

***N*-Methoxy-*N*-methyl-6-(naphthalen-2-yl)hept-6-enamide (3u)**

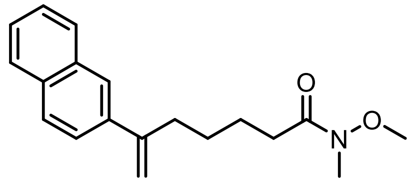

Following GP2 (4.8 mmol scale), the desired Weinreb amide was obtained as a yellow oil (188 mg, 0.63 mmol, 13%).

^{1}H NMR (400 MHz, $CDCl_3$): δ 7.85 – 7.78 (m, 4H), 7.57 (dd, *J* = 8.6, 1.8 Hz, 1H), 7.48 – 7.43 (m, 2H), 5.41 (d, *J* = 1.3 Hz, 1H), 5.18 (d, *J* = 1.3 Hz, 1H), 3.64 (s, 3H), 3.16 (s, 3H), 2.66 (t, *J* = 7.5 Hz, 2H), 2.44 – 2.38 (m, 2H), 1.75 – 1.67 (m, 2H), 1.59 – 1.52 (m, 2H) ppm.

^{13}C NMR (101 MHz, CDCl$_3$): δ 174.7, 148.2, 138.6, 133.5, 132.9, 128.3, 127.9, 127.7, 126.2, 125.9, 124.9, 124.8, 113.2, 61.3, 35.3, 28.2, 24.5 ppm.

IR (neat) ν_{max}: 1658, 1460, 1438, 1414, 1382, 994, 891, 859, 819, 750 cm^{-1}.

HRMS (ESI$^+$): exact mass calculated for [M+Na]$^+$ ($C_{19}H_{23}NO_2Na$) requires *m/z* 320.1621, found *m/z* 320.1608.

6-(Dibenzo[*b,d*]furan-1-yl)-*N*-methoxy-*N*-methylhept-6-enamide (3v)

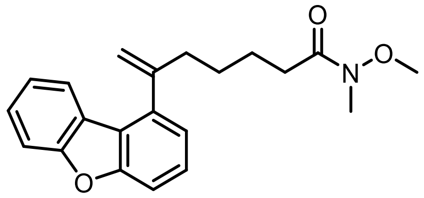

Following GP2 (4.7 mmol scale), the desired Weinreb amide was obtained as a colorless oil (744 mg, 2.2 mmol, 47%).

^{1}H NMR (400 MHz, CDCl$_3$): δ 8.06 (dd, *J* = 7.8, 0.6 Hz, 1H), 7.56 (d, *J* = 8.2 Hz, 1H), 7.48 – 7.36 (m, 3H), 7.31 – 7.26 (m, 1H), 7.11 (dd, *J* = 7.4, 0.9 Hz, 1H), 5.42 (d, *J* = 1.6 Hz, 1H), 5.25 (d, *J* = 1.7 Hz, 1H), 3.58 (s, 3H), 3.13 (s, 3H), 2.67 – 2.56 (m, 2H), 2.37 (t, *J* = 7.4 Hz, 2H), 1.75 – 1.62 (m, 2H), 1.58 – 1.50 (m, 2H) ppm.

^{13}C NMR (101 MHz, CDCl$_3$): δ 156.4, 156.4, 148.0, 139.0, 126.9, 126.9, 124.0, 122.8, 122.6, 122.5, 121.4, 114.9, 111.6, 110.3, 61.3, 37.3, 32.3, 31.8, 28.1, 24.5 ppm.

IR (neat) ν_{max}: 2936, 1713, 1660, 1450, 1414, 1219, 751, 728 cm^{-1}.

HRMS (ESI$^+$): exact mass calculated for [M+Na]$^+$ ($C_{21}H_{23}O_3NNa$) requires *m/z* 360.1570, found *m/z* 360.1565.

3.3.2. Characterization of Ketones

1-Phenylhept-6-en-1-one (4a)

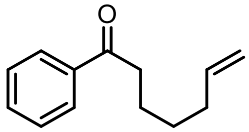

Following GP3 (7.0 mmol scale), the title compound was obtained as a colorless oil (1.15 g, 6.10 mmol, 87%).

All NMR data were in good agreement with the literature.[19]

^{1}H NMR (400 MHz, $CDCl_3$): δ 7.99 – 7.87 (m, 2H), 7.61 – 7.51 (m, 1H), 7.51 – 7.38 (m, 2H), 5.82 (ddt, *J* = 16.9, 10.2, 6.7 Hz, 1H), 5.08 – 4.88 (m, 2H), 3.06 – 2.82 (t, *J* = 7.4 Hz, 2H), 2.11 (dd, *J* = 14.3, 7.2 Hz, 2H), 1.77 (dt, *J* = 15.1, 7.4 Hz, 2H), 1.54 – 1.35 (m, 2H) ppm.

1-(3-Fluorophenyl)hept-6-en-1-one (4b)

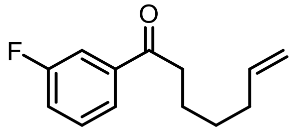

Following GP3 (3.83 mmol scale), the title compound was obtained as a colorless liquid (360 mg, 1.75 mmol, 46%).

All NMR data were in good agreement with the literature.[20]

^{1}H NMR (400 MHz, $CDCl_3$): δ 7.73 (dd, *J* = 7.7, 1.0 Hz, 1H), 7.63 (ddd, *J* = 9.5, 2.5, 1.5 Hz, 1H), 7.44 (td, *J* = 8.0, 5.6 Hz, 1H), 7.28 – 7.21 (m, 1H), 5.81 (ddt, *J* = 16.9, 10.2, 6.7 Hz, 1H), 5.09 – 4.91 (m, 2H), 2.95 (t, *J* = 7.3 Hz, 2H), 2.17 – 2.05 (m, 2H), 1.83 – 1.69 (m, 2H), 1.52 – 1.42 (m, 2H).

1-(4-Fluorophenyl)hept-6-en-1-one (4c)

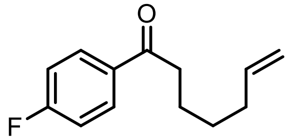

Following GP3 (2.75 mmol scale), the title compound was obtained as a colorless oil (0.430 g, 2.10 mmol, 76%).

^{1}H NMR (700 MHz, $CDCl_3$) δ 8.02 – 7.81 (m, 2H), 7.17 – 7.01 (m, 2H), 5.81 (ddt, *J* = 16.9, 10.2, 6.7 Hz, 1H), 5.05 – 4.93 (m, 2H), 2.94 (t, *J* = 7.4 Hz, 2H), 2.11 (dd, *J* = 14.4, 7.1 Hz, 2H), 1.80 – 1.69 (m, 2H), 1.48 (dd, *J* = 8.7, 6.5 Hz, 2H) ppm.

^{13}C NMR (176 MHz, CDCl$_3$) δ 198.6, 165.8 (d, J = 254.4 Hz), 138.4, 133.6 (d, J = 3.0 Hz), 130.8 (d, J = 9.2 Hz, 2C), 115. 8 (d, J = 21.8 Hz, 2C), 114.8, 38.3, 33.5, 28.5, 23.7 ppm.

^{19}F NMR (376 MHz, CDCl$_3$) δ -105.17 ppm.

IR (neat) ν_{max}: 3076, 2933, 2859, 2358, 1685, 1640, 1597, 1506, 1458, 1409, 1358, 1297, 1280, 1227, 1198, 1156, 991, 912, 841, 750 cm^{-1}.

HRMS (ESI$^+$): exact mass calculated for [M+Na]$^+$ ($C_{13}H_{15}OFNa$) requires m/z 229.1005, found m/z 229.0993.

1-(3,5-Bis(trifluoromethyl)phenyl)hept-6-en-1-one (4d)

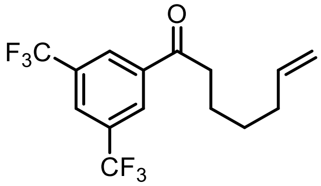

Following GP3 (1.3 mmol scale), the title compound was obtained as a colorless liquid (270 mg, 0.80 mmol, 67%).

^{1}H NMR (600 MHz, CDCl$_3$): δ 8.38 (s, 2H), 8.06 (s, 1H), 5.82 (ddt, J = 16.9, 10.2, 6.7 Hz, 1H), 5.12 – 4.80 (m, 2H), 3.03 (t, J = 7.3 Hz, 2H), 2.19 – 1.99 (m, 2H), 1.83 – 1.62 (m, 2H), 1.54 – 1.44 (m, 2H) ppm.

^{13}C NMR (151 MHz, CDCl$_3$): δ 197.4, 138.6, 138.4, 132.51 (q, J = 33.9 Hz, 2C), 128.2 – 128.1 (m, 2C), 126.4 – 126.2 (m), 123.08 (q, J = 273.0 Hz, 2C), 115.08, 38.73, 33.59, 28.46, 23.41 ppm.

^{19}F NMR (376 MHz, CDCl$_3$): δ -62.94 ppm.

IR (neat) ν_{max}: 3074, 2926, 2857, 1661, 1593, 1552, 1526, 1483, 1443, 1412, 1366, 1327, 1283, 1235, 1184, 1167, 1112, 992, 975, 909, 817, 751 cm^{-1}.

HRMS (ESI$^+$): exact mass calculated for [M+H]$^+$ ($C_{15}H_{15}OF_6$)$^+$ requires m/z 324.0949, found m/z 325.2502.

1-(3,5-Difluorophenyl)hept-6-en-1-one (4e)

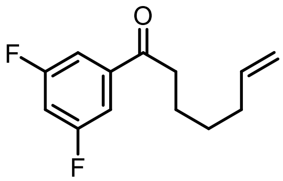

Following GP3 (0.50 mmol scale), the title compound was obtained as a colorless liquid (68.6 mg, 0.306 mmol, 61%).

^{1}H NMR (400 MHz, CDCl$_3$): δ 7.50 – 7.37 (m, 2H), 7.01 (tt, *J* = 8.5, 2.4 Hz, 1H), 5.81 (ddt, *J* = 16.9, 10.2, 6.7 Hz, 1H), 5.08 – 4.92 (m, 2H), 2.92 (t, *J* = 7.3 Hz, 2H), 2.16 – 2.06 (m, 2H), 1.76 (dt, *J* = 20.3, 7.4 Hz, 2H), 1.55 – 1.44 (m, 2H) ppm.

^{13}C NMR (101 MHz, CDCl$_3$): δ 197.7 (dd, *J* = 2.3, 2.3 Hz), 163.2 (dd, *J* = 250.7, 11.7 Hz, 2C), 140.1 (dd, *J* = 7.3, 7.3 Hz), 138.5, 115.0, 111.6 – 110.6 (m, 2C), 108.4 (dd, *J* = 25.5, 25.5 Hz), 38.7, 33.6, 28.5, 23.6 ppm.

^{19}F NMR (659 MHz, CDCl$_3$): δ -109.09 ppm.

IR (neat) ν_{max}: 2935, 2860, 2358, 1685, 1640, 1597, 1506, 1458, 1409, 1358, 129, 1227, 1198, 1156, 991, 750 cm^{-1}.

HRMS (ESI$^+$): exact mass calculated for $[M+H]^+$ ($C_{13}H_{15}OF_2$) requires *m/z* 225.1085, found *m/z* 225.1084.

1-(4-(Trifluoromethyl)phenyl)hept-6-en-1-one (4f)

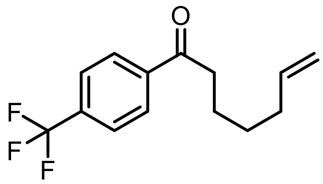

Following GP3 (3.0 mmol scale), the title compound was obtained as a colorless liquid (636 mg, 2.48 mmol, 83%).

^{1}H NMR (400 MHz, CDCl$_3$): δ 8.05 (d, *J* = 8.1 Hz, 2H), 7.73 (d, *J* = 8.2 Hz, 2H), 5.81 (ddt, *J* = 16.9, 10.2, 6.7 Hz, 1H), 5.11 – 4.85 (m, 2H), 3.00 (t, *J* = 7.3 Hz, 2H), 2.17 – 2.01 (m, 2H), 1.86 – 1.67 (m, 2H), 1.54 – 1.43 (m, 2H) ppm.

^{13}C NMR (101 MHz, CDCl$_3$): δ 199.2, 139.7, 138.4, 134.4 (q, *J* = 32.7 Hz), 128.4 (2C), 125.8 (q, *J* = 3.7 Hz, 2C), 123.7 (q, *J* = 271 Hz), 114.8, 38.7, 33.5, 28.5, 23.5 ppm.

^{19}F NMR (376 MHz, CDCl$_3$): δ -63.11 ppm.

IR (neat) ν_{max}:2932, 1691, 1641, 1581, 1511, 1409, 1322, 1167, 1127, 1065, 848, 731, 601 cm^{-1}.

HRMS (ESI$^+$): exact mass calculated for $[M+H]^+$ ($C_{14}H_{16}F_3O$) requires m/z 257.1148, found m/z 257.1142.

1-(3,4-Dichlorophenyl)hept-6-en-1-one (4g)

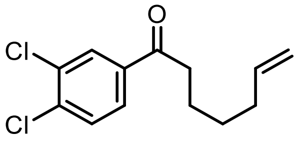

Following GP3 (0.40 mmol scale), the title compound was obtained as pale-yellow oil (91.0 mg, 0.35 mmol, 83%).

All NMR data were in good accordance to the literature.[20]

^{1}H NMR (400 MHz, CDCl$_3$): δ 8.02 (d, *J* = 2.0 Hz, 1H), 7.77 (dd, *J* = 8.4, 2.0 Hz, 1H), 7.54 (d, *J* = 8.4 Hz, 1H), 5.81 (ddt, *J* = 16.9, 10.2, 6.7 Hz, 1H), 5.07 – 4.90 (m, 2H), 2.93 (t, *J* = 7.3 Hz, 2H), 2.16 – 2.04 (m, *J* = 14.3, 7.2 Hz, 2H), 1.81 – 1.66 (m, 2H), 1.54 – 1.44 (m, 2H) ppm.

1-(3-Chlorophenyl)hept-6-en-1-one (4h)

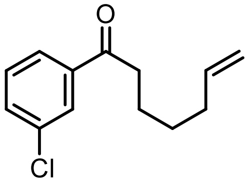

Following GP3 (0.5 mmol scale), the title compound was obtained as a colorless liquid (86.4 mg, 0.39 mmol, 77%).

All NMR data were in good accordance to the literature.[20]

^{1}H NMR (400 MHz, CDCl$_3$): δ 7.92 (t, *J* = 1.8 Hz, 1H), 7.90 – 7.80 (m, 1H), 7.54 – 7.52 (m, 1H), 7.40 (t, *J* = 8.9 Hz, 1H), 5.86 – 5.77 (m, 1H), 5.05 – 4.95 (m, 2H), 2.95 (t, *J* = 7.4 Hz, 2H), 2.14 – 2.08 (m, 2H), 1.79 – 1.72 (m, 2H), 1.52 – 1.45 (m, 2H) ppm.

1-(4-(*tert*-Butyl)phenyl)hept-6-en-1-one (4j)

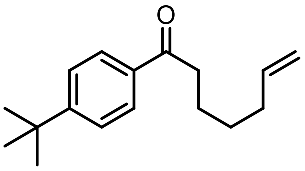

Following GP3 (2.3 mmol scale), the title compound was obtained as a colorless liquid (0.31 g, 1.3 mmol, 56%).

^{1}H NMR (400 MHz, CDCl$_3$): δ 7.90 (d, *J* = 8.3 Hz, 2H), 7.47 (d, *J* = 8.3 Hz, 2H), 5.82 (dd, *J* = 17.0, 10.2 Hz, 1H), 4.99 (dd, *J* = 25.4, 13.5 Hz, 2H), 2.95 (t, *J* = 7.4 Hz, 2H), 2.18 – 2.01 (m, 2H), 1.76 (dt, *J* = 15.1, 7.4 Hz, 2H), 1.48 (dt, *J* = 13.5, 7.0 Hz, 2H), 1.40 – 1.26 (s, 9H) ppm.

^{13}C NMR (176 MHz, CDCl$_3$): δ 200.0, 156.5, 138.6, 134.4, 128.0 (2C), 125.4 (2C), 114.6, 38.3, 35.2, 33.5, 31.0 (3C), 28.6, 23.9 ppm.

IR (neat) ν_{max}: 3075, 2962, 2866, 1680, 1640, 1605, 1566, 1461, 1406, 1363, 1269, 1229, 1189, 1107, 990, 909, 842, 826, 730, 707 cm^{-1}.

HRMS (ESI$^+$): exact mass calculated for [M+Na]$^+$ ($C_{17}H_{24}ONa$) requires *m/z* 267.1725, found *m/z* 267.1713.

4-(Hept-6-enoyl)benzonitrile (4k)

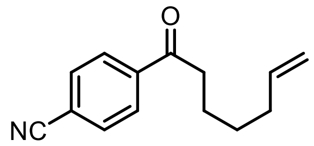

Following GP3 (2.82 mmol scale), the title compound was obtained as a colorless oil (248 mg, 1.16 mmol, 41%).

^{1}H NMR (400 MHz, CDCl$_3$): δ 8.06 – 7.97 (m, 2H), 7.80 – 7.73 (m, 2H), 5.81 (ddt, *J* = 16.9, 10.2, 6.7 Hz, 1H), 5.05 – 4.92 (m, 2H), 2.98 (t, *J* = 7.3 Hz, 2H), 2.15 – 2.06 (m, 2H), 1.82 – 1.73 (m, 2H), 1.55 – 1.42 (m, 2H) ppm.

^{13}C NMR (101 MHz, CDCl$_3$): δ 198.9, 140.1, 138.4, 132.7 (2C), 128.6 (2C), 118.1, 116.4, 115.0, 38.8, 33.6, 28.6, 23.6 ppm.

IR (neat) ν_{max}: 2230, 1688, 1639, 1403, 1220, 990, 911, 844 cm^{-1}.

HRMS (ESI$^+$): exact mass calculated for [M+H]$^+$ ($C_{14}H_{16}NO$) requires *m/z* 214.1226, found *m/z* 214.1224.

Methyl 4-(hept-6-enoyl)benzoate (4l)

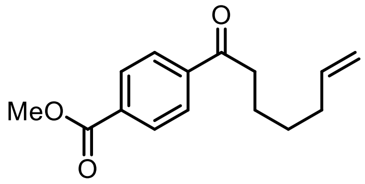

Following the GP3 (3 mmol scale), the title compound was obtained as a white solid (215 mg, 0.87 mmol, 29%).

^{1}H NMR (400 MHz, CDCl$_3$): δ 8.14 – 8.10 (m, 2H), 8.00 (d, *J* = 8.6 Hz, 2H), 5.81 (ddt, *J* = 16.9, 10.2, 6.7 Hz, 1H), 5.07 – 4.92 (m, 2H), 3.95 (s, 3H), 3.00 (t, *J* = 7.3 Hz, 2H), 2.11 (dd, *J* = 14.3, 7.2 Hz, 2H), 1.76 (dd, *J* = 15.3, 7.6 Hz, 2H), 1.54 – 1.44 (m, 2H) ppm.

^{13}C NMR (101 MHz, CDCl$_3$): δ 199.9, 166.4, 140.4, 138.6, 133.9, 130.0 (2C), 128.1 (2C), 114.9, 52.6, 38.9, 33.7, 28.6, 23.8 ppm.

IR (neat) ν_{max}: 1720, 1679, 1435, 1407, 1277, 1239, 1196, 1109, 959, 921, 761, 730 cm^{-1}.

HRMS (QTOF): exact mass calculated for $[M]^+$ ($C_{15}H_{18}O_3$) requires *m/z* 246.1250, found *m/z* 246.1243.

1-(4-(Trifluoromethoxy)phenyl)hept-6-en-1-one (4m)

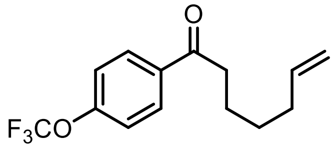

Following GP3 (4.2 mmol scale), the title compound was obtained as a colorless liquid (710 mg, 2.61 mmol, 62%).

All NMR data were in good agreement with the literature.[20]

^{1}H NMR (400 MHz, CDCl$_3$): δ 8.03 – 7.95 (m, 2H), 7.28 (d, *J* = 8.6 Hz, 2H), 5.81 (ddt, *J* = 16.9, 10.2, 6.7 Hz, 1H), 5.06 – 4.93 (m, 2H), 2.96 (t, *J* = 7.4 Hz, 2H), 2.11 (dd, *J* = 14.3, 7.2 Hz, 2H), 1.76 (dt, *J* = 20.2, 7.4 Hz, 2H), 1.53 – 1.44 (m, 2H) ppm.

1-(Thiophen-2-yl)hept-6-en-1-one (4n)

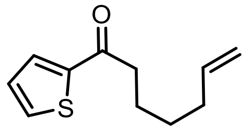

Following GP3 (7.5 mmol scale), the title compound was obtained as a colorless oil (622 mg, 3.2 mmol, 43%).

^{1}H NMR (400 MHz, CDCl$_3$): δ 7.71 (dd, *J* = 3.8, 1.0 Hz, 1H), 7.62 (dd, *J* = 4.9, 1.1 Hz, 1H), 7.13 (dd, *J* = 4.9, 3.8 Hz, 1H), 5.81 (ddt, *J* = 16.9, 10.2, 6.7 Hz, 1H), 5.07 – 4.91 (m, 2H), 2.93 – 2.86 (m, 2H), 2.14 – 2.06 (m, 2H), 1.82 – 1.71 (m, 2H), 1.53 – 1.45 (m, 2H).

^{13}C NMR (101 MHz, CDCl$_3$): δ 193.5, 144.6, 138.6, 133.5, 131.8, 128.2, 114.9, 39.4, 33.7, 28.7, 24.4 ppm.

IR (neat) ν_{max}: 1657, 1518, 1414, 1355, 1233, 1199, 1055, 909, 856, 753, 718 cm^{-1}.

HRMS (ESI$^+$): exact mass calculated for $[M+H]^+$ ($C_{11}H_{15}OS)^+$ requires *m/z* 195.0838, found *m/z* 195.0833.

1-(Furan-2-yl)hept-6-en-1-one (4o)

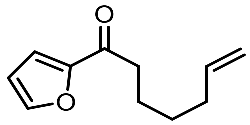

Following GP3 (2.5 mmol scale), the title compound was obtained as a colorless liquid (230 mg, 1.29 mmol, 52%)

^{1}H NMR (400 MHz, CDCl$_3$): δ 7.59 – 7.55 (m, 1H), 7.17 (dd, *J* = 3.5, 0.6 Hz, 1H), 6.52 (dd, *J* = 3.5, 1.7 Hz, 1H), 5.80 (ddt, *J* = 16.9, 10.2, 6.7 Hz, 1H), 5.06 – 4.88 (m, 2H), 2.87 – 2.77 (m, 2H), 2.15 – 2.01 (m, 2H), 1.80 – 1.67 (m, 2H), 1.51 – 1.43 (m, 2H) ppm.

^{13}C NMR (101 MHz, CDCl$_3$): δ 189.8, 153.0, 146.1, 138.6, 116.9, 114.8, 112.3, 38.4, 33.6, 28.7, 23.9 ppm.

IR (neat) ν_{max}: 1672, 1568, 1467, 1011, 994, 909, 882, 758, 594 cm^{-1}.

HRMS (ESI$^+$): exact mass calculated for $[M+H]^+$ $(C_{11}H_{15}O_2)^+$ requires *m/z* 179.1067, found *m/z* 179.1064.

2,2-Dimethylnon-8-en-3-one (4p)

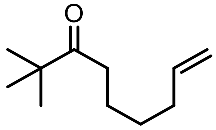

Using *N*-methoxy-*N*-methylpivalamide (purchased from BLDpharm, CAS: 64214-60-4), following GP2 (3.2 mmol scale), the title compound was obtained as a colorless liquid (91 mg, 0.54 mmol, 17%).

Careful handling is required as the ketone is volatile.

All NMR data were found to be in accordance with the literature.[21]

^{1}H NMR (400 MHz, CDCl$_3$): δ 5.80 (ddt, *J* = 16.9, 10.2, 6.7 Hz, 1H), 5.05 – 4.87 (m, 2H), 2.48 (t, *J* = 7.3 Hz, 2H), 2.11 – 1.99 (m, 2H), 1.62 – 1.51 (m, 2H), 1.43 – 1.31 (m, 2H), 1.13 (s, 9H) ppm.

1-(Adamantan-1-yl)hept-6-en-1-one (4q)

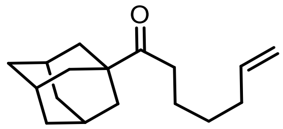

Following GP3 (5.0 mmol scale), the title compound was obtained as a colorless oil (451 mg, 1.83 mmol, 37%).

^{1}H NMR (400 MHz, CDCl$_3$): δ 5.80 (ddt, *J* = 16.9, 10.2, 6.7 Hz, 1H), 5.05 – 4.90 (m, 2H), 2.44 (t, *J* = 7.3 Hz, 2H), 2.10 – 1.99 (m, 5H), 1.83 – 1.78 (m, 5H), 1.78 – 1.65 (m, 7H), 1.58 – 1.50 (m, 2H), 1.41 – 1.32 (m, 2H) ppm.

^{13}C NMR (101 MHz, CDCl$_3$): δ 215.8, 138.9, 114.6, 46.5, 38.4 (3C), 36.8 (3C), 35.9, 33.8, 28.8 (3C), 28.1, 23.4 ppm.

IR (neat) ν_{max}: 2902, 2849, 1697, 1451, 991, 907 cm^{-1}.

HRMS (ESI$^+$): exact mass calculated for [M+Na]$^+$ ($C_{17}H_{26}ONa$)$^+$ requires *m/z* 269.1876, found *m/z* 269.1867.

1-cyclohexylhept-6-en-1-one (4r)

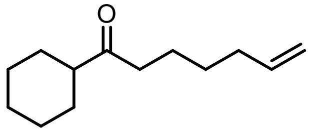

Following GP3 (3.00 mmol scale), the title compound was obtained as a colorless oil (0.470 g, 2.42 mmol, 81%).

^{1}H NMR (400 MHz, CDCl$_3$) δ 5.79 (ddt, *J* = 16.9, 10.1, 6.7 Hz, 1H), 5.13 – 4.82 (m, 2H), 2.43 (t, *J* = 7.3 Hz, 2H), 2.38 – 2.23 (m, 1H), 2.14 – 1.95 (m, 2H), 1.80 (ddtd, *J* = 15.9, 11.4, 3.8, 2.1 Hz, 4H), 1.71 – 1.61 (m, 1H), 1.63 – 1.50 (m, 2H), 1.45 – 1.19 (m, 5H), 0.94 – 0.80 (m, 2H) ppm.

^{13}C NMR (101 MHz, CDCl$_3$) δ 214.4, 138.7, 114.7, 50.9, 40.6, 33.7, 28.8, 28.7 (2C), 26.0, 25.8 (2C), 23.3.

IR (neat) ν_{max}: : 2927, 1656, 1450, 1250, 1176, 1136, 1041, 992, 977, 930, 838 cm^{-1}.

HRMS (ESI$^+$): exact mass calculated for [M+Na]$^+$ ($C_{17}H_{26}ONa$)$^+$ requires *m/z* 195.1743, found *m/z* 195.1740.

6-Methyl-1-phenylhept-6-en-1-one (4s)

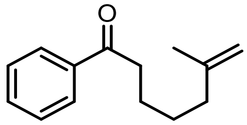

Following GP3, using 6-bromo-2-methylhex-1-ene (3.0 mmol), the title compound was obtained as a colorless oil (0.65 g, 3.7 mmol, 73%).

^{1}H NMR (600 MHz, CDCl$_3$): δ 7.96 (d, *J* = 8.0 Hz, 2H), 7.55 (dd, *J* = 14.2, 6.9 Hz, 1H), 7.46 (t, *J* = 7.7 Hz, 2H), 4.70 (d, *J* = 14.1 Hz, 2H), 2.99 (t, *J* = 7.3 Hz, 2H), 2.09 – 1.98 (m, 2H), 1.79 – 1.68 (m, 2H), 1.68 – 1.61 (m, 3H), 1.61 – 1.46 (m, 2H) ppm.

^{13}C NMR (151 MHz, CDCl$_3$): δ 200.3, 145.6, 137.0, 132.8, 128.5 (2C), 128.0 (2C), 109.9, 38.4, 37.5, 27.2, 23.9, 22.3 ppm.

IR (neat) ν_{max}: 3069, 2925, 2854, 1752, 1685, 1649, 1597, 1580, 1448, 1409, 1372, 1239, 1221, 1196, 1178, 1079, 1050, 1024, 1001, 975, 916, 885, 750, 690 cm^{-1}.

HRMS (ESI$^+$): exact mass calculated for [M+Na]$^+$ ($C_{14}H_{18}ONa$) requires *m/z* 225.1256, found *m/z* 225.1245.

1,6-Diphenylhept-6-en-1-one (4t)

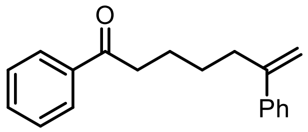

Following GP3 using **3s** and commercially available phenylmagnesium bromide, the desired ketone was obtained as a colorless oil (707 mg, 2.67 mmol, 89%).

^{1}H NMR (400 MHz, CDCl$_3$): δ 7.98 – 7.91 (m, 2H), 7.58 – 7.52 (m, 1H), 7.47 – 7.43 (m, 2H), 7.42 – 7.38 (m, 2H), 7.35 – 7.30 (m, 2H), 7.29 – 7.26 (m, 1H), 5.28 (d, *J* = 1.4 Hz, 1H), 5.08 (dd, *J* = 2.7, 1.3 Hz, 1H), 3.00 – 2.92 (m, 2H), 2.62 – 2.53 (m, 2H), 1.85 – 1.72 (m, 2H), 1.62 – 1.49 (m, 2H) ppm.

^{13}C NMR (101 MHz, CDCl$_3$): δ 200.4, 148.3, 141.4, 137.2, 133.0, 128.7 (2C), 128.4 (2C), 128.2 (2C), 127.5, 126.3 (2C), 112.6, 38.6, 35.3, 28.0, 24.1 ppm.

IR (neat) ν_{max}: 1677, 1595, 1491, 1445, 1370, 1271, 1234, 1193, 979, 895, 776, 748, 727, 697 cm^{-1}.

HRMS (ESI$^+$): exact mass calculated for [M+Na]$^+$ ($C_{19}H_{20}ONa$)$^+$ requires *m/z* 287.1406, found *m/z* 287.1406.

6-(Naphthalen-2-yl)-1-phenylhept-6-en-1-one (4u)

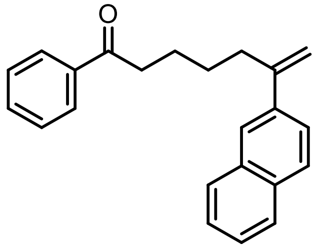

Following GP3 using **3t** and commercially available phenylmagnesium bromide, the desired ketone was obtained a yellow oil (96 mg, 0.31 mmol, 54%).

^{1}H NMR (400 MHz, CDCl$_3$): δ 7.94 – 7.91 (m, 2H), 7.84 – 7.78 (m, 4H), 7.59 – 7.53 (m, 2H), 7.48 – 7.42 (m, 4H), 5.42 (d, *J* = 1.3 Hz, 1H), 5.18 (d, *J* = 1.3 Hz, 1H), 2.99 – 2.92 (m, 2H), 2.69 (t, *J* = 7.5 Hz, 2H), 1.87 – 1.77 (m, 2H), 1.65 – 1.56 (m, 2H) ppm.

^{13}C NMR (101 MHz, CDCl$_3$): δ 200.5, 148.2, 138.6, 137.2, 133.5, 133.0, 132.9, 128.7 (2C), 128.3, 128.2 (2C), 128.0, 127.7, 126.2, 125.9, 124.9, 113.3, 38.6, 35.4, 28.1, 24.1 ppm.

IR (neat) ν_{max}: 1677, 1445, 1191, 890, 822, 742, 727, 685 cm^{-1}.

HRMS (ESI⁺): exact mass calculated for [M+Na]⁺ $(C_{23}H_{22}ONa)^+$ requires *m/z* 337.1563, found *m/z* 337.1559.

6-(Dibenzo[*b,d*]furan-1-yl)-1-phenylhept-6-en-1-one (4v)

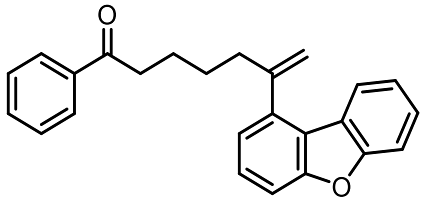

Following GP3 (2.2 mmol scale) using **3u** and commercially available phenylmagnesium bromide, the desired ketone was obtained as a colorless oil (601 mg, 1.7 mmol, 77%).

^{1}H NMR (400 MHz, $CDCl_3$): δ 8.05 (d, *J* = 7.8 Hz, 1H), 7.89 (dd, *J* = 8.2, 1.0 Hz, 2H), 7.58 – 7.39 (m, 7H), 7.30 – 7.26 (m, 1H), 7.11 (d, *J* = 7.4 Hz, 1H), 5.44 (s, 1H), 5.26 (s, 1H), 2.92 (t, *J* = 7.3 Hz, 2H), 2.65 (t, *J* = 7.6 Hz, 2H), 1.87 – 1.73 (m, 2H), 1.61 – 1.52 (m, 2H) ppm.

^{13}C NMR (101 MHz, $CDCl_3$): δ 200.3, 156.4, 156.4, 147.9, 138.9, 137.1, 133.0, 128.7 (2C), 128.1 (2C), 127.0, 126.9, 124.0, 122.7, 122.6, 122.5, 121.4, 115.1, 111.7, 110.3, 38.5, 37.4, 28.0, 24.2 ppm.

IR (neat) ν_{max}: 1682, 1448, 1414, 1193, 905, 798, 749, 726, 688 cm^{-1}.

HRMS (ESI⁺): exact mass calculated for [M+Na]⁺ $(C_{25}H_{22}O_3Na)^+$ requires *m/z* 377.1512, found *m/z* 377.1506.

1-Phenylprop-2-en-1-one (S1)

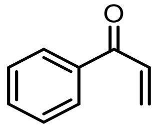

The title compound was prepared according to literature.[22]

^{1}H NMR (400 MHz, $CDCl_3$): δ 7.98 – 7.88 (m, 2H), 7.62 – 7.54 (m, 1H), 7.52 – 7.43 (m, 2H), 7.16 (dd, *J* = 17.1, 10.6 Hz, 1H), 6.49 – 6.28 (m, 1H), 5.94 (dd, *J* = 10.6, 1.7 Hz, 1H) ppm.

3.3.3. Characterization of Silyl Enol Ethers

(*Z*)-Trimethyl((1-phenylhepta-1,6-dien-1-yl)oxy)silane (1a)

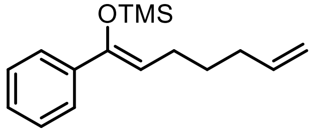

Following GP4 (1.05 mmol scale), the title compound was obtained as a colorless oil (220 mg, 0.84 mmol, 80%; Z/E 6:1).

All NMR data were in good agreement with the literature.[23]

^{1}H NMR (600 MHz, CDCl$_3$): δ 7.49 – 7.41 (m, 2H), 7.29 (t, *J* = 7.6 Hz, 2H), 7.26 – 7.19 (m, 1H), 5.90 – 5.73 (m, *J* = 6.8 Hz, 1H), 5.25 (t, *J* = 7.2 Hz, 1H), 5.08 – 4.89 (m, 2H), 2.21 (dd, *J* = 15.0, 7.4 Hz, 2H), 2.12 (dd, *J* = 14.6, 7.0 Hz, 2H), 1.66 – 1.42 (m, 2H), 0.13 (s, 9H) ppm.

(*Z*)-((1-(3-Fluorophenyl)hepta-1,6-dien-1-yl)oxy)trimethylsilane (1b)

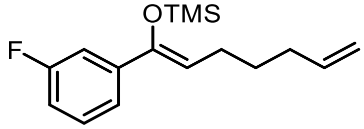

Following GP4 (1.73 mmol scale), the title compound was obtained as a colorless liquid (165 mg, 0.59 mmol, 34%).

^{1}H NMR (600 MHz, CDCl$_3$): δ 7.26 – 7.23 (m, *J* = 4.9 Hz, 2H), 7.15 (d, *J* = 10.7 Hz, 1H), 6.94 – 6.89 (m, 1H), 5.84 (ddt, *J* = 16.9, 10.2, 6.7 Hz, 1H), 5.28 (t, *J* = 7.2 Hz, 1H), 5.03 (dd, *J* = 17.1, 1.4 Hz, 1H), 4.97 (d, *J* = 10.2 Hz, 1H), 2.23 – 2.17 (m, *J* = 15.0, 7.4 Hz, 2H), 2.14 – 2.08 (m, *J* = 7.2 Hz, 2H), 1.55 – 1.49 (m, 2H), 0.14 (s, 9H) ppm.

^{13}C NMR (151 MHz, CDCl$_3$): δ 162.9 (d, *J* = 244.5 Hz), 148.2 (d, *J* = 2.2 Hz), 141.8 (d, *J* = 7.5 Hz), 138.9, 129.6 (d, *J* = 8.5 Hz), 121.0 (d, *J* = 2.5 Hz), 114.8, 114.3, 114.2, 112.3, 33.8, 29.0, 25.9, 0.7 (3C) ppm.

^{19}F NMR (565 MHz, CDCl$_3$): δ -113.80 (dd, *J* = 12.7, 8.7 Hz) ppm.

IR (neat) ν_{max}: 3077, 2958, 2361, 1642, 1611, 1584, 1484, 1334, 1287, 1177, 1156, 1055 cm^{-1}.

HRMS (ESI$^+$): exact mass calculated for [M+H]$^+$ ($C_{16}H_{24}FOSi$) requires *m/z* 279.1575, found *m/z* 279.1572.

(*Z*)-((1-(4-Fluorophenyl)hepta-1,6-dien-1-yl)oxy)trimethylsilane (1c)

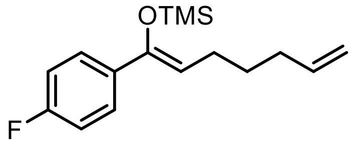

Following GP4 (0.780 mmol scale), the title compound was obtained as a colorless liquid (200 mg, 0.718 mmol, 93%).

^{1}H NMR (400 MHz, $CDCl_3$): δ 7.47 – 7.37 (m, 2H), 7.02 – 6.88 (m, 2H), 5.93 – 5.74 (m, 1H), 5.16 (t, *J* = 7.2 Hz, 1H), 5.09 – 4.90 (m, 2H), 2.24 – 2.13 (m, 2H), 2.16 – 2.05 (m, 2H), 1.59 – 1.45 (m, 2H), 0.12 (s, 9H) ppm.

^{13}C NMR (101 MHz, $CDCl_3$): δ 162.4 (d, *J* = 253.2 Hz), 148.4, 138.3, 135.6 (d, *J* = 3.1 Hz), 127.1 (d, *J* = 8.0 Hz, 2C), 115.0 (d, *J* = 21.0 Hz, 2C), 114.7, 111.1, 33.8, 29.1, 25.9, 0.8 (3C) ppm.

^{19}F NMR (376 MHz, $CDCl_3$): δ -115.17 ppm.

IR (neat) ν_{max}: 2957, 2856, 1689, 1599, 1278, 1095, 837, 753 cm^{-1}.

HRMS - GC (+EI): exact mass calculated for $[M]^+$ ($C_{16}H_{23}FOSi$) requires *m/z* 277.1418, found *m/z* 277.1494.

(*Z*)-((1-(3,5-Bis(trifluoromethyl)phenyl)hepta-1,6-dien-1-yl)oxy)trimethylsilane (1d)

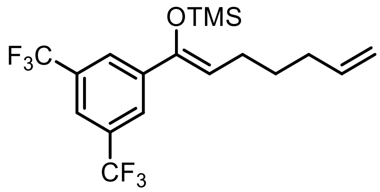

Following GP4 (0.70 mmol scale), the title compound was obtained as a colorless liquid (242 mg, 0.61 mmol, 84%).

^{1}H NMR (400 MHz, $CDCl_3$): δ 7.90 (app s, 2H), 7.73 (s, 1H), 5.84 (ddt, *J* = 16.9, 10.2, 6.7 Hz, 1H), 5.45 (t, *J* = 7.3 Hz, 1H), 5.09 – 4.91 (m, 2H), 2.29 – 2.17 (m, 2H), 2.18 – 2.05 (m, 2H), 1.61 – 1.48 (m, 2H), 0.15 (s, 9H) ppm.

^{13}C NMR (101 MHz, $CDCl_3$): δ 146.1, 140.7, 137.9, 131.02 (q, *J* = 33.2 Hz, 2C), 125.2 –125.1 (m, 2C), 123.5 (q, *J* = 273.5 Hz, 2C), 121.1 – 120.8 (m), 114.9, 114.3, 33.7, 28.7, 26.1, 0.6 (3C) ppm.

^{19}F NMR (376 MHz, $CDCl_3$): δ -63.02 ppm.

IR (neat) ν_{max}: 2956, 2860, 1702, 1382, 1276, 1130, 993, 842, 681 cm^{-1}.

HRMS - GC (+EI): exact mass calculated for $[M]^+$ ($C_{18}H_{22}F_6OSi$) requires *m/z* 396.1333, found *m/z* 396.1333.

(*Z*)-((1-(3,5-Difluorophenyl)hepta-1,6-dien-1-yl)oxy)trimethylsilane (1e)

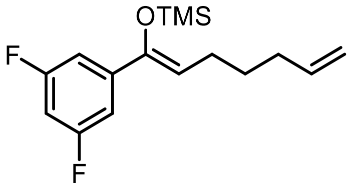

Following GP4 (1.6 mmol scale), the title compound was obtained as a colorless liquid (348 mg, 1.17 mmol, 76%).

^{1}H NMR (700 MHz, $CDCl_3$): δ 6.99 – 6.94 (m, 2H), 6.67 (tt, *J* = 5.8, 2.2 Hz, 1H), 5.83 (ddt, *J* = 17.1, 10.2, 6.7 Hz, 1H), 5.30 (t, *J* = 7.2 Hz, 1H), 5.03 (app dd, *J* = 17.1, 1.4 Hz, 1H), 4.97 (app dd, *J* = 10.2, 1.1 Hz, 1H), 2.20 (td, *J* = 7.5, 7.2 Hz, 2H), 2.11 (td, *J* = 7.5, 6.7 Hz, 2H), 1.52 (tt, *J* = 7.5, 7.5 Hz, 2H), 0.15 (s, 9H) ppm.

^{13}C NMR (176 MHz, $CDCl_3$): δ 163.0 (dd, *J* = 247.1 Hz, 13.2 Hz, 2C), 147.4 (dd, *J* = 3.0, 3.0 Hz), 142.9 (dd, *J* = 9.2, 9.2 Hz), 138.7, 114.9, 113.3, 108.1 (dd, *J* = 21.4 Hz, 4.7 Hz, 2C), 102.6 (dd, *J* = 25.7, 25.7 Hz) 33.7, 28.9, 25.9, 0.7 (3C) ppm.

^{19}F NMR (659 MHz, $CDCl_3$): δ −110.5 ppm.

IR (neat) ν_{max}: 3079, 2959, 2858, 1621, 1590, 1442, 1335, 1253, 1180, 1116, 1083 cm^{-1}.

HRMS - GC (+EI): exact mass calculated for $[M]^+$ ($C_{16}H_{22}F_2OSi$) requires *m/z* 296.1408, found *m/z* 296.1395.

(*Z*)-Trimethyl((1-(4-(trifluoromethyl)phenyl)hepta-1,6-dien-1-yl)oxy)silane (1f)

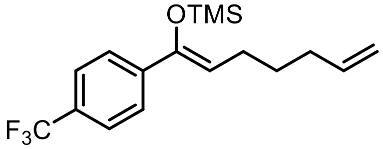

Following GP4 (2.4 mmol scale), the title compound was obtained as a colorless liquid (632 mg, 1.92 mmol, 80%).

^{1}H NMR (400 MHz, $CDCl_3$): δ 7.67 – 7.40 (m, 4H), 5.97 – 5.74 (m, 1H), 5.42 – 5.21 (m, 1H), 5.10 – 4.84 (m, 2H), 2.29 – 2.18 (m, 2H), 2.18 – 2.05 (m, 2H), 1.61 – 1.42 (m, 2H), 0.20 (s, 9H) ppm.

^{13}C NMR (101 MHz, $CDCl_3$): δ 148.1, 142.8, 138.8, 129.38 (q, *J* = 32.7 Hz), 125.5 (2C), 125.21 (q, *J* = 3.7 Hz, 2C), 124.89 (q, *J* = 272 Hz), 114.8, 113.5, 33.7, 29.9, 26.0, 0.7 (3C) ppm.

^{19}F NMR (376 MHz, $CDCl_3$): δ -62.44 (d, *J* = 2.4 Hz) ppm.

IR (neat) ν_{max}: 2927, 1642, 1616, 1409, 1322, 1252, 1123, 1067, 885, 753, 605 cm^{-1}.

HRMS (ESI$^+$): exact mass calculated for $[M+H]^+$ ($C_{17}H_{24}F_3OSi$) requires *m/z* 329.1543, found *m/z* 329.1543.

(*Z*)-((1-(3,4-Dichlorophenyl)hepta-1,6-dien-1-yl)oxy)trimethylsilane (1g)

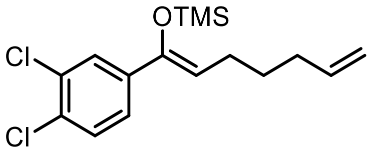

Following GP4 (2.23 mmol scale), the title compound was obtained as a colorless liquid (360 mg, 1.09 mmol, 49%).

^{1}H NMR (700 MHz, $CDCl_3$): δ 7.54 (d, *J* = 2.0 Hz, 1H), 7.35 (d, *J* = 8.4 Hz, 1H), 7.29 (dd, *J* = 8.4, 2.0 Hz, 1H), 5.83 (ddt, *J* = 17.0, 10.3, 6.7 Hz, 1H), 5.27 (t, *J* = 7.2 Hz, 1H), 5.03 (app dd, *J* = 17.0, 1.7 Hz, 1H), 4.97 (app dd, *J* = 10.3, 1.0 Hz, 1H), 2.19 (td, *J* = 7.5, 7.2 Hz, 2H), 2.11 (td, *J* = 7.5, 6.7 Hz, 2H), 1.52 (tt, *J* = 7.5, 7.5 Hz, 2H), 0.14 (s, 9 H) ppm.

^{13}C NMR (176 MHz, $CDCl_3$): δ 147.2, 139.5, 138.7, 132.4, 131.2, 130.1, 127.2, 124.6, 114.8, 112.9, 33.7, 28.9, 25.9, 0.7 (3C) ppm.

IR (neat) ν_{max}: 3077, 2958, 2856, 2362, 1642, 1589, 1469, 1380, 1286, 1136, 1087, 1028 cm^{-1}.

HRMS - GC (+EI): exact mass calculated for $[M]^+$ ($C_{16}H_{22}Cl_2OSi$) requires *m/z* 328.0817, found *m/z* 328.0796.

(*Z*)-((1-(3-Chlorophenyl)hepta-1,6-dien-1-yl)oxy)trimethylsilane (1h)

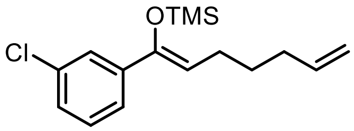

Following GP4 (1.75 mmol scale), the title compound was obtained as a colorless liquid (165 mg, 0. 60 mmol, 34%).

^{1}H NMR (600 MHz, $CDCl_3$): δ 7.44 (app s, 1H), 7.34 (app d, *J* = 7.1 Hz, 1H), 7.24 – 7.18 (m, 2H), 5.84 (ddt, *J* = 17.0, 10.3, 6.7 Hz, 1H), 5.27 (t, *J* = 7.2 Hz, 1H), 5.03 (app d, *J* = 17.0 Hz, 1H), 4.97 (app d, *J* = 10.3 Hz, 1H), 2.20 (td, *J* = 7.5, 7.2 Hz, 2H), 2.11 (td, *J* = 7.5, 6.7 Hz, 2H), 1.52 (tt, *J* = 7.5, 7.5 Hz, 2H), 0.14 (s, 9H) ppm.

^{13}C NMR (151 MHz, $CDCl_3$): δ 148.0, 141.3, 138.8, 134.2, 129.4, 127.5, 125.6, 123.5, 114.8, 112.4, 33.7, 29.0, 25.9, 0.7 (3C) ppm.

IR (neat) ν_{max}: 3076, 2958, 2925, 2856, 2361, 1642, 1594, 1566, 1473, 1333, 1282, 1251, 1058 cm^{-1}.

HRMS - GC (+EI): exact mass calculated for $[M]^+$ ($C_{16}H_{23}ClOSi$) requires *m/z* 294.1206, found *m/z* 294.1191.

(*Z*)-((1-(2-Bromophenyl)hepta-1,6-dien-1-yl)oxy)trimethylsilane (1i)

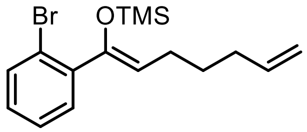

Following GP4 (0.73 mmol scale) with (1-(2-bromophenyl)hept-6-en-1-one (purchased from Enamine), the title compound was obtained as a colorless liquid (193 mg, 0.57 mmol, 78%).

^{1}H NMR (400 MHz, CDCl$_3$): δ 7.54 (dd, *J* = 8.0, 1.1 Hz, 1H), 7.34 (dd, *J* = 7.6, 1.8 Hz, 1H), 7.26 – 7.22 (m, 1H), 7.13 – 7.08 (m, 1H), 5.86 (ddt, *J* = 16.9, 10.2, 6.7 Hz, 1H), 5.06 – 5.00 (m, 1H), 4.98 – 4.90 (m, 2H), 2.27 – 2.19 (m, 2H), 2.14 (tt, *J* = 8.0, 1.3 Hz, 2H), 1.59 – 1.49 (m, 2H), 0.02 (d, *J* = 3.4 Hz, 9H) ppm.

^{13}C NMR (101 MHz, CDCl$_3$): δ 147.9, 140.8, 139.1, 133.3, 131.0, 129.1, 127.1, 122.4, 115.2, 114.6, 33.7, 29.0, 25.3, 0.5 (3C) ppm.

IR (neat) ν_{max}: 3075, 2957, 2855, 2362, 1701, 1660, 1561, 1468, 1428, 1307, 1251, 1038, 1024 cm^{-1}.

HRMS (ESI$^+$): exact mass calculated for $[M+H]^+$ ($C_{16}H_{24}BrOSi$)$^+$ requires *m/z* 341.0755, found *m/z* 341.0750.

(*Z*)-((1-(4-(*tert*-Butyl)phenyl)hepta-1,6-dien-1-yl)oxy)trimethylsilane (1j)

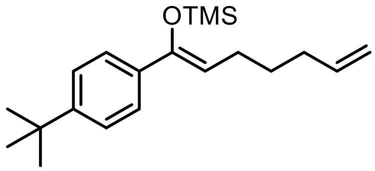

Following GP4 (0.6 mmol scale), the title compound was obtained as a colorless liquid (180 mg, 0.57 mmol, 93%).

^{1}H NMR (400 MHz, CDCl$_3$): δ 7.42 – 7.36 (m, 2H), 7.34 – 7.28 (m, 2H), 5.91 – 5.76 (m, 1H), 5.22 (t, *J* = 7.2 Hz, 1H), 5.08 – 4.89 (m, 2H), 2.25 – 2.16 (m, 2H), 2.16 – 2.06 (m, 2H), 1.57 – 1.45 (m, 2H), 1.33 (s, 9H), 0.14 (s, 9H) ppm.

^{13}C NMR (101 MHz, CDCl$_3$): δ 150.5, 149.2, 139.0, 136.4, 125.1 (2C), 125.0 (2C), 114.6, 110.5, 34.6, 33.8, 31.4 (3C), 29.2, 25.9, 0.8 (3C) ppm.

IR (neat) ν_{max}: 2960, 1685, 1460, 1331, 1111, 991, 840, 753 cm^{-1}.

HRMS (ESI$^+$): exact mass calculated for $[M+H]^+$ ($C_{20}H_{33}OSi$) requires *m/z* 317.2295, found *m/z* 317.2294.

(*Z*)-4-(1-((Trimethylsilyl)oxy)hepta-1,6-dien-1-yl)benzonitrile (1k)

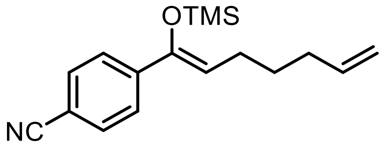

Following GP4 (1.00 mmol scale), the title compound was obtained as a colorless liquid (228 mg, 0.80 mmol, 80%).

^{1}H NMR (400 MHz, CDCl$_3$): δ 7.60 – 7.53 (m, 4H), 5.83 (ddt, *J* = 16.9, 10.1, 6.7 Hz, 1H), 5.40 (t, *J* = 7.2 Hz, 1H), 5.06 – 4.94 (m, 2H), 2.27 – 2.20 (m, 2H), 2.15 – 2.09 (m, 2H), 1.56 – 1.50 (m, 2H), 0.14 (s, 9H) ppm.

^{13}C NMR (101 MHz, CDCl$_3$): δ 147.8, 143.7, 138.6, 132.2 (2C), 125.7 (2C), 119.2, 114.9, 114.8, 110.8, 33.7, 28.8, 26.1, 0.7 (3C) ppm.

IR (neat) ν_{max}: 2227, 1704, 1640, 1605, 1281, 1252, 838, 754 cm^{-1}.

HRMS - GC (+EI): exact mass calculated for [M-H$^-$]$^+$ ($C_{17}H_{22}NOSi$) requires *m/z* 284.1470, found *m/z* 284.1458.

Methyl (*Z*)-4-(1-((trimethylsilyl)oxy)hepta-1,6-dien-1-yl)benzoate (1l)

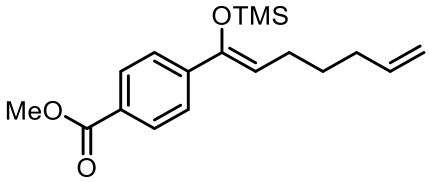

Following GP4, the title compound was obtained as a colorless oil (234 mg, 0.74 mmol, 86%).

^{1}H NMR (400 MHz, CDCl$_3$): δ 7.96 (d, *J* = 8.7 Hz, 2H), 7.52 (d, *J* = 8.6 Hz, 2H), 5.84 (ddt, *J* = 16.9, 10.2, 6.7 Hz, 1H), 5.39 (t, *J* = 7.2 Hz, 1H), 5.07 – 5.01 (m, 1H), 4.99 – 4.96 (m, 1H), 3.91 (s, 3H), 2.23 (dd, *J* = 15.1, 7.3 Hz, 2H), 2.12 (dd, *J* = 14.6, 7.1 Hz, 2H), 1.58 – 1.49 (m, 2H), 0.13 (s, 9H) ppm.

^{13}C NMR (101 MHz, CDCl$_3$): δ 167.1, 148.5, 143.8, 138.8, 129.6 (2C), 129.0, 125.2 (2C), 114.8, 113.7, 52.2, 33.8, 28.9, 26.0, 0.7 (3C) ppm.

IR (neat) ν_{max}: 1720, 1640, 1608, 1435, 1273, 1251, 1103, 834, 711 cm^{-1}.

HRMS (ESI$^+$): exact mass calculated for [M+Na]$^+$ ($C_{18}H_{26}O_3SiNa$)$^+$ requires *m/z* 341.1543, found *m/z* 341.1536.

(*Z*)-Trimethyl((1-(4-(trifluoromethoxy)phenyl)hepta-1,6-dien-1-yl)oxy)silane (1m)

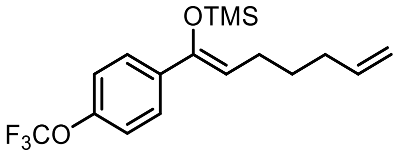

Following GP4 (1.0 mmol scale), the title compound was obtained as a colorless liquid (276 mg, 0.80 mmol, 80%).

^{1}H NMR (400 MHz, CDCl$_3$): δ 7.47 (d, *J* = 8.5 Hz, 2H), 7.13 (d, *J* = 8.8 Hz, 2H), 5.84 (ddt, *J* = 16.9, 10.1, 6.7 Hz, 1H), 5.23 (t, *J* = 7.2 Hz, 1H), 5.07 – 4.95 (m, 2H), 2.20 (dd, *J* = 14.9, 7.4 Hz, 2H), 2.12 (dd, *J* = 14.2, 7.0 Hz, 2H), 1.56 – 1.50 (m, 2H), 0.13 (s, 9H) ppm.

^{13}C NMR (101 MHz, CDCl$_3$): δ 148.6, 148.1, 138.9, 138.1, 126.7 (2C), 120.6 (2C), 120.6 (q, *J* = 257.2 Hz), 114.8, 112.1, 33.8, 29.0, 25.9, 0.7 (3C) ppm.

^{19}F NMR (377 MHz, CDCl$_3$): δ -57.85 ppm.

IR (neat) ν_{max}: 1643, 1505, 1250, 1219, 1161, 839 cm^{-1}.

HRMS (ESI$^+$): exact mass calculated for [M+H]$^+$ ($C_{17}H_{24}F_3O_2Si$)$^+$ requires *m/z* 345.1492, found *m/z* 345.1489.

(*Z*)-Trimethyl((1-(thiophen-2-yl)hepta-1,6-dien-1-yl)oxy)silane (1n)

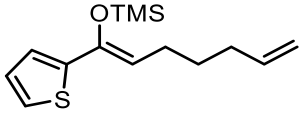

Following GP4 (3.19 mmol scale), the title compound was obtained as a colorless oil (680 mg, 2.55 mmol, 80%).

^{1}H NMR (400 MHz, CDCl$_3$): δ 7.11 (dd, *J* = 5.0, 1.1 Hz, 1H), 7.03 (dd, *J* = 3.6, 1.1 Hz, 1H), 6.93 (dd, *J* = 5.0, 3.6 Hz, 1H), 5.83 (ddt, *J* = 16.9, 10.2, 6.7 Hz, 1H), 5.24 (t, *J* = 7.3 Hz, 1H), 5.06 – 4.94 (m, 2H), 2.22 – 2.07 (m, 4H), 1.57 – 1.47 (m, 2H), 0.21 (s, 9H) ppm.

^{13}C NMR (101 MHz, CDCl$_3$): δ 144.2, 143.8, 138.9, 127.2, 123.8, 123.0, 114.7, 110.6, 33.7, 29.0, 25.8, 0.7 (3C) ppm.

IR (neat) ν_{max}: 1640, 1360, 1250, 1085, 1054, 1034, 910, 867, 840, 752, 692 cm^{-1}.

HRMS (ESI$^+$): exact mass calculated for [M+H]$^+$ ($C_{14}H_{23}OSSi$) requires *m/z* 267.1233, found *m/z* 267.1227.

(*Z*)-((1-(Furan-2-yl)hepta-1,6-dien-1-yl)oxy)trimethylsilane (1o)

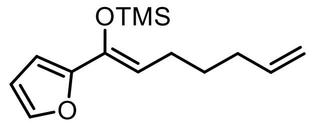

Following GP4 (1.2 mmol scale), the title compound was obtained as a colorless liquid (244 mg, 0.97 mmol, 81%).

^{1}H NMR (400 MHz, CDCl$_3$): δ 7.31 – 7.29 (m, 1H), 6.35 (dd, *J* = 3.3, 1.8 Hz, 1H), 6.24 (d, *J* = 3.3 Hz, 1H), 5.88 – 5.77 (m, 1H), 5.35 (t, *J* = 7.4 Hz, 1H), 5.05 – 4.99 (m, 1H), 4.98 – 4.93 (m, 1H), 2.22 – 2.08 (m, 4H), 1.56 – 1.47 (m, 2H), 0.21 (s, 9H) ppm.

^{13}C NMR (101 MHz, CDCl$_3$): δ 152.9, 141.6, 141.0, 138.9, 114.7, 111.1, 109.8, 105.7, 33.7, 29.0, 25.2, 0.6 ppm.

IR (neat): ν_{max} 2936, 1709, 1680, 1587, 1439, 1361, 1262, 1059, 768 cm^{-1}.

HRMS (ESI$^+$): exact mass calculated for [M+H]$^+$ ($C_{14}H_{23}O_2Si$) requires *m/z* 251.1462, found *m/z* 251.1459.

(*Z*)-((2,2-Dimethylnona-3,8-dien-3-yl)oxy)trimethylsilane (1p)

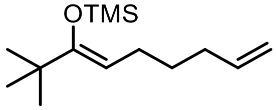

Following GP4 (0.48 mmol scale), the title compound was obtained as a colorless liquid (68 mg, 0.28 mmol, 59%).

All NMR data were found to be in accordance with the literature.[23]

^{1}H NMR (400 MHz, CDCl$_3$): δ 5.82 (dd, *J* = 17.0, 10.3 Hz, 1H), 5.04 – 4.88 (m, 2H), 4.50 (t, *J* = 6.8 Hz, 1H), 2.10 – 2.02 (m, 2H), 2.00 – 1.94 (m, 2H), 1.47 – 1.40 (m, 2H), 1.04 (s, 9H), 0.21 (s, 9H) ppm.

(((*Z*)-1-(Adamantan-1-yl)hepta-1,6-dien-1-yl)oxy)trimethylsilane (1q)

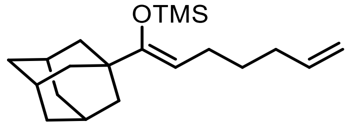

Following GP4 (1.83 mmol scale), the title compound was obtained as a colorless oil (452 mg, 1.42 mmol, 78%).

^{1}H NMR (400 MHz, CDCl$_3$): δ 5.81 (ddt, *J* = 16.9, 10.2, 6.7 Hz, 1H), 5.00 (ddd, *J* = 17.1, 3.6, 1.6 Hz, 1H), 4.95 – 4.91 (m, 1H), 4.41 (t, *J* = 6.8 Hz, 1H), 2.06 (dd, *J* = 14.6, 7.0 Hz, 2H), 2.01 – 1.94 (m, *J* = 7.0 Hz, 5H), 1.73 – 1.60 (m, 12H), 1.47 – 1.38 (m, 2H), 0.22 (s, 9H) ppm.

^{13}C NMR (101 MHz, CDCl$_3$): δ 159.0, 139.2, 114.5, 104.0, 40.7 (3C), 38.0, 37.1 (3C), 33.8, 29.5, 28.7 (3C), 25.7, 1.3 (3C) ppm.

IR (neat) ν_{max}: 1662, 1459, 1437, 1416, 1385, 1179, 996, 730 cm^{-1}.

HRMS (ESI$^+$): exact mass calculated for $[M+H]^+$ ($C_{20}H_{35}OSi$) requires *m/z* 319.2452, found *m/z* 319.2442.

((1-cyclohexylhepta-1,6-dien-1-yl)oxy)trimethylsilane (1r)

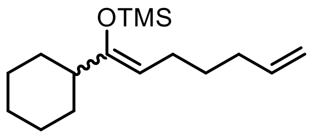

Freshly distilled diisopropylamine (1.3 eq., 1.3 mmol, 182 µL) was dissolved in THF (1 mL) at 0 °C and nBuLi in hexanes (2.5M, 1.2 eq., 1.2 mmol, 0.48 mL) was added dropwise. The mixture was stirred for 15 min at the same temperature and then cooled to -78 °C. A THF-solution of the ketone (1M, 1.0 eq., 1.0 mmol, 194 mg) was added dropwise. The mixture was warmed to room temperature over 30 min and TMSCl (1.3 eq., 1.3 mmol, 330 µL) was added. The reaction was stirred for 5 h at room temperature. The volatiles were removed under reduced pressure and the residue was dissolved in a minimal amount of CHCl3. The dissolved crude was loaded on a silica plug, which was rinsed quickly with heptanes (ca. 200 mL). After evaporating the solvent, the product was obtained as a colorless liquid (195 mg, 0.73 mmol, 73%).

(E/Z ≈ 1: 1)

^{1}H NMR (400 MHz, CDCl$_3$) δ 5.93 – 5.71 (m, 1H), 5.07 – 4.84 (m, 2H), 4.50 – 4.32 (m, 1H), 2.35 – 2.21 (m, 1H), 2.11 – 1.92 (m, 2H), 1.90 – 1.70 (m, 4H), 1.69 – 1.59 (m, 1H), 1.56 – 1.46 (m, 1H), 1.47 – 1.32 (m, 3H), 1.33 – 0.78 (m, 5H), 0.18 (dd, *J* = 3.0, 0.6 Hz, 9H).

^{13}C NMR (101 MHz, CDCl$_3$) δ 155.4, 152.8 (C') 139.3, 139.2 (C'), 114.5 (C'), 114.4, 105.7, 104.9 (C'), 44.6, 39.2 (C'), 33.8, 33.4 (C'), 31.4 (C + C'), 30.3, 30.2 (C'), 29.4, 26.6 (C + C'), 26.5, 26.2 (C'), 25.9 (C'), 25.1, 0.9, 0.7 (C').

IR (neat) ν_{max}: 2930, 2852, 1655, 1450, 1216, 1130, 1115, 1012, 990 cm^{-1}.

HRMS (ESI$^+$): exact mass calculated for $[M+H]^+$ ($C_{20}H_{35}OSi$) requires *m/z* 266.2066, found *m/z* 266.2063.

(*Z*)-Trimethyl((5-methyl-1-phenylhepta-1,6-dien-1-yl)oxy)silane (1s)

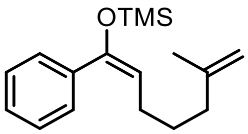

Following GP4 (0.25 mmol), the title compound was obtained as a colorless oil (40.0 mg, 0.15 mmol, 59%).

^{1}H NMR (400 MHz, $CDCl_3$) δ 7.46 (d, *J* = 7.7 Hz, 2H), 7.33 – 7.17 (m, 3H; overlaps with $CDCl_3$ peak), 5.25 (t, *J* = 7.2 Hz, 1H), 4.71 (app d, *J* = 5.5 Hz, 2H), 2.20 (dd, *J* = 15.0, 7.4 Hz, 2H), 2.12 – 2.00 (m, 2H), 1.73 (s, 3H), 1.56 (m, 2H), 0.13 (s, 9H) ppm.

^{13}C can not be recorded for this compound due to instability. The compound was used as a substrate *immediately* *after purification.*

IR (neat) ν_{max}: 3067, 2957, 1724, 1682, 1640, 1597, 1580, 1449, 1416, 1372, 1315, 1251, 1204, 1178, 1072, 1024, 994, 910, 842, 753, 732 cm^{-1}.

HRMS (ESI$^+$): exact mass calculated for $[M+Na]^+$ ($C_{17}H_{26}OSiNa$) requires *m/z* 297.1651, found *m/z* 297.1644.

(*Z*)-((1,6-Diphenylhepta-1,6-dien-1-yl)oxy)trimethylsilane (1t)

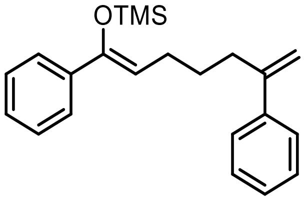

Following GP4 (1.92 mmol scale), the title compound was obtained as a yellow liquid (271 mg, 0.81 mmol, 42%).

^{1}H NMR (400 MHz, $CDCl_3$): δ 7.48 – 7.40 (m, 4H), 7.35 – 7.27 (m, 6H), 5.29 (d, *J* = 1.5 Hz, 1H), 5.23 (t, *J* = 7.1 Hz, 1H), 5.11 – 5.08 (m, 1H), 2.58 (t, *J* = 7.6 Hz, 2H), 2.24 (dd, *J* = 14.9, 7.4 Hz, 2H), 1.64 – 1.56 (m, 2H), 0.12 (s, 9H) ppm.

^{13}C NMR (101 MHz, $CDCl_3$): δ 149.3, 148.6, 141.5, 139.3, 128.4 (2C), 128.2 (2C), 127.5, 127.4, 126.3 (2C), 125.5 (2C), 112.5, 111.2, 35.3, 28.5, 26.1, 0.7 (3C) ppm.

IR (neat) ν_{max}: 1250, 1074, 1054, 885, 839, 776, 754, 694 cm^{-1}.

HRMS (ESI$^+$): exact mass calculated for $[M+H]^+$ ($C_{22}H_{29}OSi$) requires *m/z* 337.1982, found *m/z* 337.1983.

(*Z*)-Trimethyl((6-(naphthalen-2-yl)-1-phenylhepta-1,6-dien-1-yl)oxy)silane (1u)

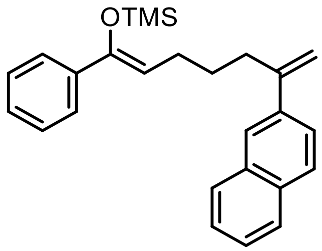

Following GP4 (0.30 mmol scale), the title compound was obtained as a yellow oil (98.2 mg, 0.25 mmol, 83%).

^{1}H NMR (400 MHz, CDCl$_3$): δ 7.85 – 7.78 (m, 4H), 7.60 (dd, *J* = 8.6, 1.8 Hz, 1H), 7.47 – 7.43 (m, 4H), 7.30 – 7.27 (m, 2H), 7.26 – 7.23 (m, 1H), 5.44 (d, *J* = 1.3 Hz, 1H), 5.24 (t, *J* = 7.2 Hz, 1H), 5.20 (d, *J* = 1.3 Hz, 1H), 2.70 (t, *J* = 7.6 Hz, 2H), 2.27 (dd, *J* = 14.8, 7.3 Hz, 2H), 1.70 – 1.61 (m, 2H), 0.12 (s, 9H) ppm.

^{13}C NMR (101 MHz, CDCl$_3$): δ 149.4, 148.4, 139.3, 138.7, 133.6, 132.9, 128.3, 128.2 (2C), 127.9, 127.7, 127.5, 126.2, 125.9, 125.5 (2C), 124.9, 124.9, 113.1, 111.2, 35.3, 28.6, 26.1, 0.7 (3C) ppm.

IR (neat) ν_{max}: 1250, 1108, 1055, 887, 840, 817, 748, 695 cm^{-1}.

HRMS (ESI^{+}): exact mass calculated for [M+H]$^{+}$ ($C_{26}H_{31}OSi$) requires *m/z* 387.2139, found *m/z* 387.2130.

(*Z*)-((6-(Dibenzo[*b,d*]furan-1-yl)-1-phenylhepta-1,6-dien-1-yl)oxy)trimethylsilane (1v)

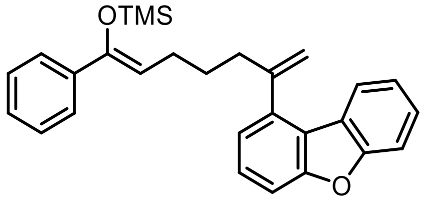

Following GP4 (0.85 mmol scale), the title compound was obtained as a colorless oil (118 mg, 0.28 mmol, 33%).

^{1}H NMR (400 MHz, CDCl$_3$): δ 8.07 (d, *J* = 7.8 Hz, 1H), 7.57 (d, *J* = 8.2 Hz, 1H), 7.47 (dd, *J* = 8.0, 7.2 Hz, 1H), 7.41 (dt, *J* = 14.1, 5.9 Hz, 4H), 7.28 – 7.22 (m, 4H), 7.12 (dd, *J* = 7.4, 0.8 Hz, 1H), 5.44 (d, *J* = 1.5 Hz, 1H), 5.26 (d, *J* = 1.7 Hz, 1H), 5.19 (t, *J* = 7.1 Hz, 1H), 2.69 – 2.62 (m, 2H), 2.24 (dd, *J* = 14.8, 7.4 Hz, 2H), 1.62 (dt, *J* = 15.3, 7.7 Hz, 2H), 0.08 (s, 9H) ppm.

^{13}C NMR (101 MHz, CDCl$_3$): δ 156.4, 156.4, 149.4, 148.2, 139.3, 139.1, 128.1 (2C), 127.5, 126.9, 126.9, 125.5 (2C), 124.1, 122.8, 122.6, 122.5, 121.4, 114.8, 111.6, 111.0, 110.3, 37.4, 28.4, 26.1, 0.7 (3C) ppm.

IR (neat) ν_{max}: 1710, 1688, 1450, 1359, 1220, 1194, 752, 727, 713 cm^{-1}.

HRMS - GC (+EI): exact mass calculated for [M]$^{+}$ ($C_{28}H_{30}O_2Si$) requires *m/z* 426.2015, found *m/z* 426.2004.

(*E*)-Trimethyl((7-methyl-1-phenylocta-1,6-dien-1-yl)oxy)silane (1y)

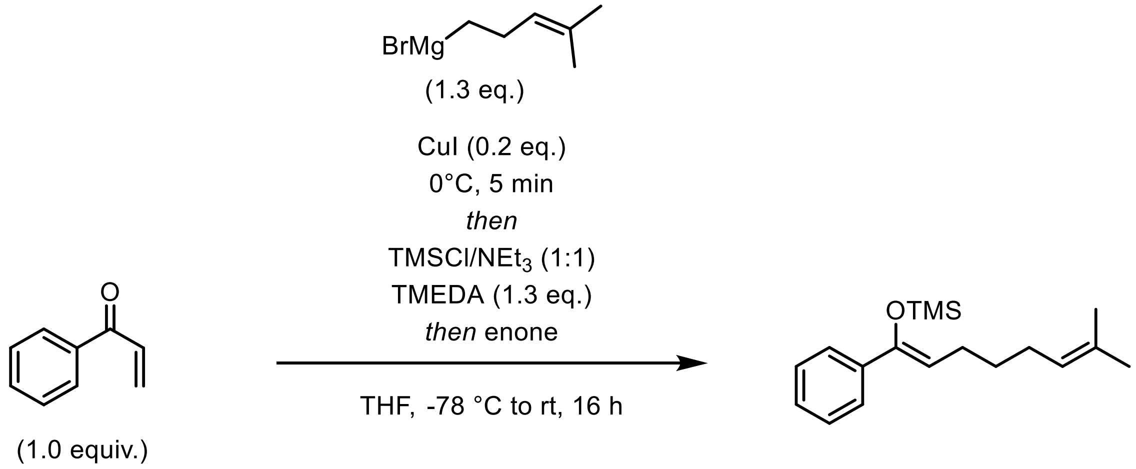

A modified reported procedure was used (2.5 mmol scale).[24]

(4-Methylpent-3-en-1-yl)-magnesium bromide was prepared according to step 1 of GP2 (3.25 mmol). The Grignard solution was subsequently cooled to 0 °C and CuI (95.7 mg, 0.5 mmol, 0.2 eq.) was added in one portion. The resulting suspension was stirred at 0 °C for 5 min before the solution was further cooled to -78 °C. Next, chlorotrimethylsilane (1.5 mL) and triethylamine (1.5 mL) were rapidly added, followed by *N*,*N*,*N*',*N*'-tetramethylethylenediamine (0.49 mL, 3.25 mmol, 1.3 eq.). Finally, 1-phenylprop-2-en-1-one (330 mg, 2.5 mmol, 1.0 eq.) was added dropwise to this mixture at -78°C and the reaction mixture was allowed to warm to room temperature and subsequently stirred for 16 h.

The solvent was removed in vacuo and the resulting crude material was immediately subjected to column chromatography. The title compound was obtained as a colorless liquid (148 mg, 0.68 mmol, 27%).

^{1}H NMR (400 MHz, CDCl$_3$): δ 7.47 – 7.44 (m, 2H), 7.32 – 7.27 (m, 3H), 5.25 (t, *J* = 7.2 Hz, 1H), 5.18 – 5.13 (m, 1H), 2.23 – 2.16 (m, 2H), 2.07 – 2.02 (m, 2H), 1.70 (s, 3H), 1.62 (s, 3H), 1.49 – 1.43 (m, 2H), 0.13 (s, 9H) ppm.

^{13}C NMR (101 MHz, CDCl$_3$): δ 149.1, 139.4, 131.7, 128.2 (2C), 127.4, 125.5 (2C), 124.7, 111.6, 30.1, 28.1, 26.1, 25.9, 17.9, 0.7 (3C) ppm.

IR (neat) ν_{max}: 2935, 1711, 1664, 1446, 1330, 1281, 1250, 1095, 1059, 1025, 884, 838, 754, 695 cm^{-1}.

HRMS (ESI$^+$): exact mass calculated for [M+Na]$^+$ ($C_{18}H_{28}OSiNa$) requires *m/z* 289.1982, found *m/z* 289.1981.

3.4. Characterization of cyclized ketones

***cis*-3-Hydroxycyclohexyl)(phenyl)methanone (2a)**

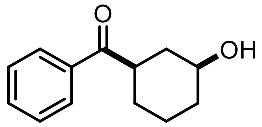

Following GP5 (0.2 mmol scale), the title compound was obtained as a colorless oil (23.0 mg, 0.110 mmol, 56%).

^{1}H NMR (600 MHz, CDCl$_3$): δ 7.95 – 7.90 (m, 2H,), 7.59 – 7.52 (m, 1H,), 7.50 – 7.42 (m, 2H,), 3.77 (tt, *J* = 10.5, 4.2 Hz, 1H,), 3.37 (tt, *J* = 11.2, 3.4 Hz, 1H), 2.18 – 2.11 (m, 1H), 2.02 (app d, *J* = 12.4 Hz, 1H), 1.97 – 1.81 (m, 2H), 1.58 – 1.49 (m, 1H), 1.47 – 1.39 (m, 2H), 1.33 – 1.22 (m, 1H) ppm. *The OH proton was not observed.*

^{13}C NMR (151 MHz, CDCl$_3$): δ 202.3, 135.9, 133.0, 128.6 (2C), 128.3 (2C), 69.9, 44.0, 37.6, 35.1, 28.5, 23.3 ppm.

IR (neat) ν_{max}: 3390, 2935, 2858, 1676, 1596, 1580, 1447, 1361, 1261, 1234, 1208, 1180, 1135, 1113, 1059, 1012,948, 899, 852, 805, 750, 699 cm^{-1}.

HRMS (ESI^{+}): exact mass calculated for [M+Na]$^{+}$ ($C_{13}H_{16}O_2Na$) requires *m/z* 227.1050, found *m/z* 227.1043.

***cis*-(3-Fluorophenyl)(3-hydroxycyclohexyl)methanone (2b)**

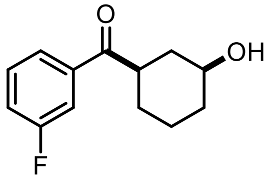

Following GP5 (0.1 mmol scale), the title compound was obtained as a colorless solid (11.0 mg, 50 µmol, 50%).

^{1}H NMR (400 MHz, CDCl$_3$): δ 7.71 (d, *J* = 7.8 Hz, 1H), 7.66 – 7.58 (m, 1H), 7.47 – 7.41 (m, 1H), 7.29 – 7.26 (m, *J* = 2.6, 0.8 Hz, 1H), 3.83 – 3.69 (m, 1H), 3.30 (tt, *J* = 11.3, 3.4 Hz, 1H), 2.18 – 2.10 (m, 1H), 2.08 – 2.00 (m, 1H), 1.95 – 1.83 (m, 2H), 1.75 (s, 1H), 1.60 – 1.26 (m, 4H) ppm.

^{13}C NMR (101 MHz, CDCl$_3$): δ 201.2, 163.1 (d, *J* = 248.0 Hz), 138.3 (d, *J* = 6.1 Hz), 130.5 (d, *J* = 7.8 Hz), 124.1 (d, *J* = 2.9 Hz), 120.2 (d, *J* = 21.5 Hz), 115.3 (d, *J* = 22.4 Hz), 70.1, 44.4, 37.8, 35.3, 28.6, 23.5 ppm.

^{19}F NMR (377 MHz, CDCl$_3$): δ -111.71 ppm.

IR (neat) ν_{max}: 2936, 1709, 1680, 1587, 1439, 1361, 1262, 1059, 768 cm^{-1}.

HRMS (ESI$^+$): exact mass calculated for $[M+Na]^+$ ($C_{13}H_{15}FO_2Na$) requires *m/z* 245.0948, found *m/z* 245.0945.

***cis*-(4-Fluorophenyl)(3-hydroxycyclohexyl)methanone (2c)**

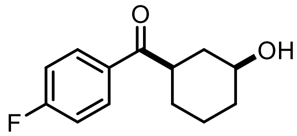

Following GP5 (0.1 mmol scale), the title compound was obtained as a colorless oil (9.10 mg, 0.041 mmol, 41%).

^{1}H NMR (600 MHz, $CDCl_3$): δ 7.98 – 7.95 (m, 2H), 7.15 – 7.12 (m, 2H), 3.77 (app s, 1H), 3.34 –3.31 (m, 1H), 2.17 – 2.06 (m, 1H), 2.08 – 1.97 (m, 1H), 1.93 – 1.80 (m, 2H), 1.77 (brs, 1H), 1.57 – 1.46 (m, 1H), 1.49 – 1.39 (m, 2H), 1.37 – 1.19 (m, 1H) ppm.

^{13}C NMR (151 MHz, $CDCl_3$): δ 200.9, 165.9 (d, *J* = 253.5 Hz), 132.5(d, *J* = 3.0 Hz), 131.1 (d, *J* = 9.0 Hz, 2C), 115.9 (d, *J* = 22.5 Hz, 2C), 70.1, 44.1, 37.8, 35.4, 28.7, 23.5 ppm.

^{19}F (372 MHz, $CDCl_3$): δ -105.3 ppm.

IR (neat) ν_{max}: 3353, 2935, 2859, 1676, 1596, 1505, 1450, 1410, 1361, 1298, 1262, 1230, 1156, 1105, 1060, 1012, 955, 882, 844, 823, 777, 749 cm^{-1}.

HRMS (ESI$^+$): exact mass calculated for $[M+Na]^+$ ($C_{13}H_{15}FO_2Na$) requires *m/z* 245.0954, found *m/z* 245.0949.

***cis*-(3,5-Bis(trifluoromethyl)phenyl)(3-hydroxycyclohexyl)methanone (2d)**

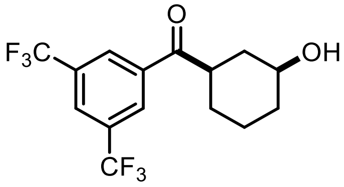

Following GP5 (0.1 mmol scale), the title compound was obtained as a colorless liquid (19.8 mg, 0.058 mmol, 58%).

^{1}H NMR (400 MHz, $CDCl_3$): δ 8.34 (s, 2H), 8.07 (s, 1H), 3.92 – 3.70 (m, 1H), 3.34 (qt, *J* = 13.9, 4.9 Hz, 1H), 2.19 – 2.10 (m, 1H), 2.10 – 2.02 (m, 1H), 2.01 – 1.92 (m, 1H), 1.90 – 1.81 (m, 1H), 1.66 (brs, 1H), 1.61 – 1.43 (m, 3H), 1.42 – 1.23 (m, 1H) ppm.

^{13}C NMR (101 MHz, CDCl$_3$): δ 199.4, 137.6, 132.5 (q, *J* = 33.9 Hz, 2C), 128.3 – 128.2 (m, 2C), 126.3 – 126.2 (m), 122.9 (q, *J* = 273.0 Hz, 2C), 69.8, 44.2, 37.4, 35.1, 28.2, 23.3 ppm.

^{19}F NMR (376 MHz, CDCl$_3$): δ -62.91 ppm.

IR (neat) ν_{max}: 3297, 2939, 2861, 1684, 1451, 1409, 1374, 1326, 1265, 1233, 1213, 1169, 1129, 1112, 1068, 1015, 984, 946, 910, 856, 767, 743 cm^{-1}.

HRMS - GC (EI$^+$): exact mass calculated for $[M]^+$ ($C_{15}H_{14}F_6O_2$) requires *m/z* 340.0893, found *m/z* 340.0886.

cis-(3,5-Difluorophenyl)(3-hydroxycyclohexyl)methanone (2e)

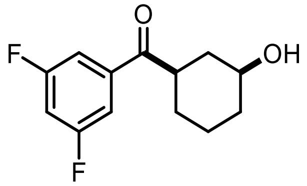

Following GP5 (0.1 mmol scale), the title compound was obtained as a colorless liquid (11.8 mg, 0.049 mmol, 49%).

^{1}H NMR (400 MHz, CDCl$_3$): δ 7.47 – 7.38 (m, 2H), 7.07 – 6.97 (m, 1H), 3.77 (tt, *J* = 10.6, 4.2 Hz, 1H), 3.33 – 3.14 (m, 1H), 2.19 – 2.08 (m, 1H), 2.04 (dt, *J* = 24.5, 10.4 Hz, 1H), 1.98 – 1.87 (m, 1H), 1.88 – 1.80 (m, 1H), 1.71 (brs, 1H), 1.56 – 1.22 (m, 4H) ppm.

^{13}C NMR (101 MHz, CDCl$_3$): δ 199.8 (dd, *J* = 1.6, 1.6 Hz), 163.3 (dd, *J* = 251.1, 11.7 Hz, 2C), 139.2 (dd, *J* = 7.2, 7.2 Hz), 111.7 – 111.1 (m, 2C), 108.5 (dd, *J* = 25.4, 25.4 Hz), 70.0, 44.4, 37.7, 35.3, 28.5, 23.5 ppm.

^{19}F NMR (376 MHz, CDCl$_3$). δ -107.95 ppm.

IR (neat) ν_{max}: 3394, 2937, 1686, 1593, 1437, 1313, 1120, 1045, 909, 753 cm^{-1}.

HRMS (ESI$^+$): exact mass calculated for $[M+Na]^+$ ($C_{13}H_{14}F_2ONa$) requires *m/z* 263.0854, found *m/z* 263.0847.

cis-(3-Hydroxycyclohexyl)(4-(trifluoromethyl)phenyl)methanone (2f)

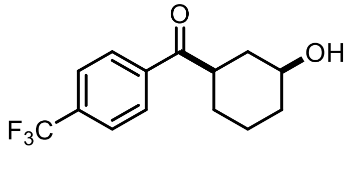

Following GP5 (0.1 mmol scale), the title compound was obtained as white solid (16.4 mg, 0.060 mmol, 60%).

All NMR data were in good accordance to the literature.[25]

^{1}**H NMR (600 MHz, CDCl₃):** δ 8.02 (d, *J* = 8.1 Hz, 2H), 7.73 (d, *J* = 8.2 Hz, 2H), 3.78 (tt, *J* = 10.5, 4.2 Hz, 1H), 3.35 (tt, *J* = 11.2, 3.3 Hz, 1H), 2.18 – 2.12 (m, 1H), 2.04 (d, *J* = 12.6 Hz, 1H), 1.95 – 1.89 (m, 1H), 1.89 – 1.84 (m, 1H), 1.80 (br s, 1H), 1.56 – 1.38 (m, 3H), 1.30 (ddd, *J* = 23.1, 12.4, 3.8 Hz, 1H) ppm.
^{13}**C NMR (151 MHz, CDCl₃):** δ 201.5, 139.0, 134.5 (q, *J* = 32.8 Hz), 128.8 (2C), 125.9 (q, *J* = 3.4 Hz, 2C), 123.7 (q, *J* = 272.5 Hz), 70.1, 44.5, 37.7, 35.3, 28.5, 23.5.

***cis*-(3,4-Dichlorophenyl)(3-hydroxycyclohexyl)methanone (2g)**

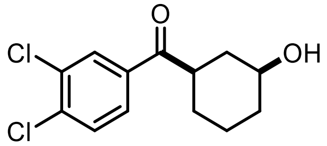

Following GP5 (0.1 mmol scale), the title compound was obtained as a colorless liquid (17.1 mg, 0.063 mmol, 63%).
^{1}**H NMR (400 MHz, CDCl$_3$):** δ 8.00 (d, *J* = 2.0 Hz, 1H), 7.75 (dd, *J* = 8.4, 2.0 Hz, 1H), 7.55 (d, *J* = 8.4 Hz, 1H), 3.84 – 3.65 (m, 1H), 3.32 – 3.17 (m, 1H), 2.19 – 2.07 (m, 1H), 2.02 – 1.99 (m, 1H), 1.99 – 1.86 (m, 1H), 1.88 – 1.78 (m, 1H), 1.69 (brs, 1H), 1.55 – 1.38 (m, 3H), 1.35 – 1.21 (m, 1H) ppm.
^{13}**C NMR (101 MHz, CDCl$_3$):** δ 200.0, 137.7, 135.6, 133.4, 130.8, 130.3, 127.3, 69.9, 44.1, 37.6, 35.1, 28.4, 23.4 ppm.
IR (neat) ν_{max}: 3366, 2936, 2859, 1682, 1582, 1556, 1465, 1450, 1390, 1264, 1204, 1141, 1062, 1030, 958, 888, 859, 832, 748, 674 cm^{-1}.
HRMS (ESI$^+$): exact mass calculated for $[M+Na]^+$ ($C_{13}H_{14}Cl_2O_2Na$) requires *m/z* 295.0263, found *m/z* 295.0263.

***cis*-(3-chlorophenyl)(3-hydroxycyclohexyl)methanone (2h)**

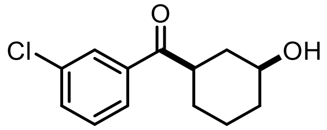

Following GP5 (0.1 mmol scale), the title compound was obtained as a colorless liquid (10.8 mg, 0.045 mmol, 45%).
^{1}**H NMR (400 MHz, CDCl$_3$):** δ 7.89 (t, *J* = 1.8 Hz, 1H), 7.83 – 7.77 (m, 1H), 7.54 (ddd, *J* = 8.0, 2.1, 1.0 Hz, 1H), 7.45 – 7.38 (m, 1H), 3.77 (ddd, *J* = 14.6, 10.4, 4.1 Hz, 1H), 3.30 (tt, *J* = 11.3, 3.4 Hz, 1H), 2.19 – 2.08 (m, 1H), 2.04 (d, *J* = 12.1 Hz, 1H), 1.96 – 1.79 (m, 2H), 1.67 (brs, 1H), 1.60 – 1.37 (m, 4H) ppm.

^{13}C NMR (101 MHz, CDCl$_3$): δ 201.2, 137.8, 135.2, 133.1, 130.2, 128.6, 126.5, 70.1, 44.4, 37.8, 35.2, 28.5, 23.5 ppm.

IR (neat) ν_{max}: 3298, 2931, 2858, 1682, 1555, 1466, 1391, 1264,1141, 1062, 1030, 958, 888, 748 cm^{-1}.

HRMS (ESI$^+$): exact mass calculated for $[M+Na]^+$ ($C_{13}H_{15}ClO_2Na$) requires m/z 261.0653, found m/z 261.0652.

***cis*-3-Hydroxycyclohexyl(2-bromophenyl)methanone (2i)**

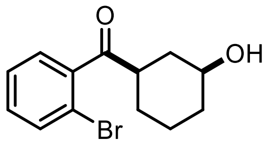

Following GP5 (0.1 mmol scale), the title compound was obtained as a colorless liquid (14.2 mg, 50 µmol, 50%).

All NMR data were in good accordance with the literature.[25]

^{1}H NMR (400 MHz, CDCl$_3$): δ 7.61 – 7.58 (m, 1H), 7.36 (td, *J* = 7.5, 1.2 Hz, 1H), 7.32 – 7.26 (m, 2H), 3.73 – 3.57 (m, 1H), 3.17 – 3.07 (m, 1H), 2.24 – 2.16 (m, 1H), 2.04 – 1.96 (m, 1H), 1.93 – 1.84 (m, 2H), 1.75 – 1.58 (m, 1H), 1.48 – 1.22 (m, 4H) ppm.

^{13}C NMR (101 MHz, CDCl$_3$): δ 206.6, 141.9, 133.5, 131.4, 128.3, 127.5, 118.7, 70.2, 48.6, 37.1, 35.2, 27.5, 23.5 ppm.

***cis*-(4-(*tert*-butyl)phenyl)(3-hydroxycyclohexyl)methanone (2j)**

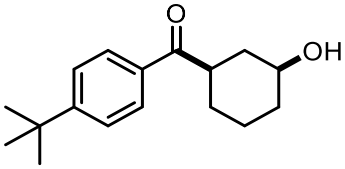

Following GP5 (0.1 mmol scale), the title compound was obtained as a colorless liquid (10.2 mg, 0.039 mmol, 39%).

^{1}H NMR (600 MHz, CDCl$_3$): δ 7.89 – 7.87 (m, 2H), 7.48 – 7.42 (m, 2H), 3.78 (tt, *J* = 10.4, 4.2 Hz, 1H), 3.37 (tt, *J* = 11.0, 3.5 Hz, 1H), 2.15 – 2.11 (m, 1H), 2.04 – 1.99 (m, 1H), 1.91 – 1.84 (m, 2H), 1.60 (brs, 1H), 1.57 – 1.51 (m, 1H), 1.47 – 1.42 (m, 2H), 1.34 (s, 9H), 1.32 – 1.29 (m, 1H) ppm.

^{13}C NMR (151 MHz, CDCl$_3$): δ 202.2, 156.9, 133.5, 128.5 (2C), 125.8 (2C), 70.1, 44.0, 37.8, 35.4, 32.3, 31.2 (3C), 28.7, 23.5 ppm.

IR (neat) ν_{max}: 3400, 2935, 2861, 1674, 1604, 1563, 1463, 1450, 1408, 1363, 1320, 1297, 1267, 1234, 1190, 1109, 1061, 1009, 955, 946, 882, 844, 804, 763, 715, 699 cm^{-1}.

HRMS (ESI$^+$): exact mass calculated for $[M+Na]^+$ ($C_{17}H_{24}O_2Na$) requires *m/z* 283.1669, found *m/z* 283.1658.

***cis*-4-(3-Hydroxycyclohexane-1-carbonyl)benzonitrile (2k)**

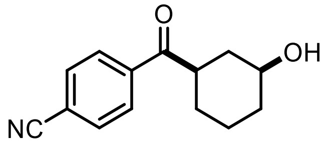

Following GP5 (0.1 mmol scale), the title compound was obtained as a colorless solid (11.7 mg, 51 µmol, 51%).

^{1}H NMR (400 MHz, $CDCl_3$): δ 8.04 – 7.93 (m, 2H), 7.81 – 7.69 (m, 2H), 3.83 – 3.70 (m, 1H), 3.32 (tt, *J* = 11.4, 3.4 Hz, 1H), 2.17 – 2.10 (m, 1H), 2.08 – 2.02 (m, 1H), 1.96 – 1.81 (m, 2H), 1.71 (app s, 1H), 1.56 – 1.23 (m, 4H) ppm.

^{13}C NMR (101 MHz, $CDCl_3$): δ 201.1, 139.3, 132.7 (2C), 128.8 (2C), 118.1, 116.4, 70.0, 44.5, 37.6, 35.3, 28.4, 23.5 ppm.

IR (neat) ν_{max}: 2933, 1708, 1685, 1406, 1360, 1290, 1261, 1220, 1063, 956, 945, 855, 758, 732 cm^{-1}.

HRMS (ESI$^+$): exact mass calculated for $[M+Na]^+$ ($C_{14}H_{15}NO_2$) requires *m/z* 230.1176, found *m/z* 230.1169.

syn-Methyl 4-(3-hydroxycyclohexane-1-carbonyl)benzoate (2l)

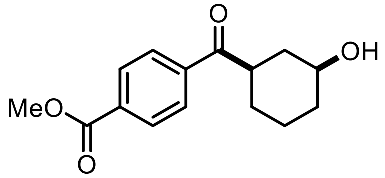

Following GP5 (0.1 mmol scale), the title compound was obtained as a colorless solid (15.7 mg, 60 µmol, 60%).

All NMR data were in good agreement with the literature.[25]

^{1}H NMR (400 MHz, $CDCl_3$): δ 8.15 – 8.06 (m, 2H), 8.00 – 7.90 (m, 2H), 3.95 (s, 3H), 3.83 – 3.73 (m, 1H), 3.36 (tt, *J* = 11.2, 3.4 Hz, 1H), 2.18 – 2.11 (m, 1H), 2.08 – 2.00 (m, *J* = 12.3, 1.7 Hz, 1H), 1.94 – 1.84 (m, 2H), 1.75 (s, 1H), 1.59 – 1.26 (m, 4H) ppm.

^{13}C NMR (101 MHz, $CDCl_3$): δ 202.0, 166.4, 139.5, 134.0, 130.1 (2C), 128.4 (2C), 70.1, 52.6, 44.5, 37.7, 35.3, 28.5, 23.5 ppm.

***cis*-(3-Hydroxycyclohexyl)(4-(trifluoromethoxy)phenyl)methanone (2m)**

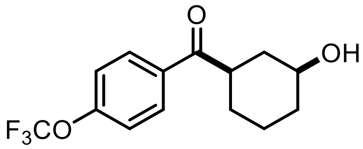

Following GP5 (0.1 mmol scale), the title compound was obtained as a colorless oil (15.1 mg, 52 µmol, 52%).

All NMR data were in good agreement with the literature.[25]

^{1}H NMR (400 MHz, CDCl$_3$): δ 8.01 – 7.96 (m, 2H), 7.29 (d, *J* = 8.0 Hz, 2H), 3.77 (tt, *J* = 10.5, 4.2 Hz, 1H), 3.33 (tt, *J* = 11.3, 3.5 Hz, 1H), 2.17 – 2.10 (m, 1H), 2.06 – 2.00 (m, 1H), 1.90 – 1.30 (m, 7H) ppm.

^{13}C NMR (101 MHz, CDCl$_3$): δ 200.9, 152.7, 134.3, 130.5 (2C), 120.7 (2C), 120.4 (q, *J* = 258.8 Hz), 70.1, 44.2, 37.8, 35.3, 28.6, 23.5 ppm.

^{19}F NMR (376 MHz, CDCl$_3$): δ -57.62 ppm.

***cis*-(3-Hydroxycyclohexyl)(thiophen-2-yl)methanone (2s)**

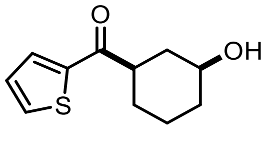

Following GP5 (0.1 mmol scale), the title compound was obtained as a white solid (10.1 mg, 48 µmol, 48%).

All NMR data were in good accordance with the literature.[25]

^{1}H NMR (400 MHz, CDCl$_3$): 7.72 (dd, *J* = 3.8, 1.0 Hz, 1H), 7.64 (dd, *J* = 5.0, 1.1 Hz, 1H), 7.13 (dd, *J* = 4.9, 3.8 Hz, 1H), 3.80 – 3.65 (m, 1H), 3.23 – 3.18 (m, 1H), 2.20 – 2.13 (m, 1H), 2.04 – 1.98 (m, 1H), 1.94 – 1.84 (m, 3H), 1.64 – 1.25 (m, 4H) ppm.

^{13}C NMR (101 MHz, CDCl$_3$): δ 195.4, 143.5, 134.0, 131.9, 128.3, 69.9, 45.8, 37.9, 35.2, 28.9, 23.4 ppm.

***cis*-Furan-2-yl(3-hydroxycyclohexyl)methanone (2o)**

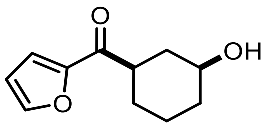

Following GP5 (0.1 mmol scale), the title compound was obtained as a colorless oil (6.3 mg, 32 µmol, 32%).

All NMR data were in good accordance with the literature.[25]

^{1}H NMR (400 MHz, CDCl$_3$): δ 7.59 – 7.56 (m, 1H), 7.21 (d, *J* = 3.6 Hz, 1H), 6.54 (dd, *J* = 3.6, 1.7 Hz, 1H), 3.74 (t, *J* = 10.3 Hz, 1H), 3.24 – 3.14 (m, 1H), 2.19 – 2.10 (m, 1H), 2.04 – 1.97 (m, 1H), 1.91 – 1.84 (m, 2H), 1.78 (app s, 1H), 1.56 – 1.50 (m, 1H), 1.48 – 1.40 (m, 2H), 1.34 – 1.24 (m, 1H) ppm.
^{13}C NMR (101 MHz, CDCl$_3$): δ 191.5, 152.3, 146.5, 117.5, 112.4, 70.0, 44.8, 37.3, 35.3, 28.1, 23.4 ppm.

***cis*-1-(-3-Hydroxycyclohexyl)-2,2-dimethylpropan-1-one (2p)**

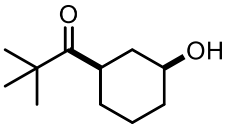

Following GP5 (0.1 mmol scale), the title compound was obtained as a colorless oil (13.9 mg, 75 µmol, 75%).
All NMR data were in good agreement with the literature.[25]
^{1}H NMR (400 MHz, CDCl$_3$): δ 3.67 – 3.55 (m, 1H), 2.92 (tt, *J* = 11.5, 3.5 Hz, 1H), 2.01 – 1.94 (m, 1H), 1.90 – 1.81 (m, 2H), 1.67 (app s, 1H), 1.61 – 1.53 (m, 1H), 1.36 – 1.21 (m, 4H), 1.14 (s, 9H) ppm.
^{13}C NMR (101 MHz, CDCl$_3$): δ 217.5, 70.1, 44.9, 43.1, 38.6, 35.3, 29.0, 26.1 (3C), 23.8 ppm.

***cis*-Adamantan-1-yl-(3-hydroxycyclohexyl)methanone (2q)**

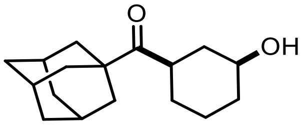

Following GP5 (0.1 mmol scale), the title compound was obtained as a white solid (18.7 mg, 71 µmol, 71%).
All NMR data were in good accordance with the literature.[25]
^{1}H NMR (400 MHz, CDCl$_3$): δ 3.65 – 3.55 (m, 1H), 2.94 (tt, *J* = 11.3, 3.5 Hz, 1H), 2.04 (app s, 3H), 2.01 – 1.93 (m, 1H), 1.86 – 1.66 (m, 15H), 1.55 – 1.48 (m, 1H), 1.41 – 1.20 (m, 4H) ppm.
^{13}C NMR (101 MHz, CDCl$_3$): δ 217.0, 70.1, 47.1, 42.1, 38.3, 37.8 (3C), 36.7 (3C), 35.3, 28.8, 27.9 (3C), 23.7 ppm.

***cis-cyclohexyl((1R,3S)-3-hydroxycyclohexyl)methanone* (2r)**

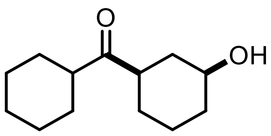

Following GP5 (0.1 mmol scale), the title compound was obtained as a white solid (8.1 mg, 39 µmol, 39%).

¹H NMR (600 MHz, CDCl₃) δ 3.64 (tt, *J* = 10.4, 4.2 Hz, 1H), 2.59 (tt, *J* = 11.3, 3.6 Hz, 1H), 2.50 (tt, *J* = 11.2, 3.0 Hz, 1H), 2.06 – 2.00 (m, 1H), 1.98 – 1.92 (m, 1H), 1.87 – 1.81 (m, 1H), 1.80 – 1.71 (m, 5H), 1.70 – 1.62 (m, 1H), 1.38 – 1.17 (m, 8H) ppm.

¹³C NMR (151 MHz, CDCl₃) δ 215.8, 70.1, 49.4, 47.6, 37.2, 35.3, 28.9, 28.7, 27.7, 26.0, 25.9, 25.8 ppm.

IR (neat) ν_{max}: 3372, 2910, 2750, 1690, 1450, 1304, 1256, 1067, 1012, 953 cm^{-1}.

HRMS (ESI$^+$): exact mass calculated for $[M+Na]^+$ ($C_{13}H_{22}O_2Na$) requires *m/z* 233.1512, found *m/z* 233.1511.

***cis*-3-Hydroxy-3-methylcyclohexyl)(phenyl)methanone (2s)**

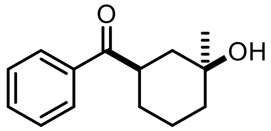

Following GP5 (0.1 mmol scale), the title compound was obtained as a colorless oil (12.2 mg, 0.057 mmol, 57%).

¹H NMR (600 MHz, CDCl₃): δ 7.97 – 7.88 (m, 2H), 7.58 (m, 1H), 7.47 (t, *J* = 7.8 Hz, 2H), 3.52 (tt, *J* = 9.0, 4.4 Hz, 1H), 2.67 (br s, 1H), 1.84 (m, 2H), 1.78 (app ddd, *J* = 13.5, 8.7, 4.9 Hz, 2H), 1.64 (app ddd, *J* = 32.9, 14.4, 7.6 Hz, 2H), 1.55 – 1.43 (m, 2H), 1.34 (s, 3H) ppm.

¹³C NMR (151 MHz, CDCl₃): δ 203.5, 135.9, 133.0, 128.6 (2C), 128.3 (2C), 69.8, 42.8, 41.44, 39.8, 28.4, 27.4, 21.7 ppm.

IR (neat) ν_{max}: 3387, 2930, 2858, 1646, 1600, 1580, 1449, 1371, 1240, 1208, 1171, 1135, 1110, 1059, 1010, 948, 900, 852, 807, 750 cm^{-1}.

HRMS (ESI$^+$): exact mass calculated for $[M+Na]^+$ ($C_{14}H_{18}O_2Na$) requires *m/z* 241.1205, found *m/z* 241.1208.

***cis*-(3-Hydroxy-3-phenylcyclohexyl)(phenyl)methanone (2t)**

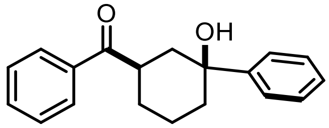

Following GP5 (0.1 mmol scale), the title compound was obtained as a white solid (15.0 mg, 54 µmol, 54%).

¹H NMR (600 MHz, CDCl₃): δ 7.94 – 7.86 (m, 2H), 7.64 – 7.60 (m, 2H), 7.59 – 7.55 (m, 1H), 7.50 – 7.43 (m, 2H), 7.41 – 7.37 (m, 2H), 7.30 – 7.26 (m, 1H), 4.44 (s, 1H), 3.68 (p, *J* = 5.3 Hz, 1H), 2.32 (dd, *J* = 14.4, 5.2 Hz, 1H), 2.19 – 2.10 (m, 2H), 1.99 – 1.81 (m, 4H), 1.62 – 1.56 (m, 1H) ppm.

^{13}C NMR (151 MHz, $CDCl_3$): δ 205.2, 147.5, 135.9, 133.4, 128.9 (2C), 128.8 (2C), 128.5 (2C), 127.1, 125.6 (2C), 72.1, 42.2, 39.7, 38.8, 28.3, 20.0 ppm.

IR (neat) ν_{max}: 3360, 2925, 1653, 1447, 1416, 1227, 979, 699, 687 cm^{-1}.

HRMS (ESI$^+$): exact mass calculated for $[M+Na]^+$ ($C_{19}H_{20}O_2Na$) requires *m/z* 303.1356, found *m/z* 303.1354.

***cis*-3-Hydroxy-3-(naphthalen-2-yl)cyclohexyl)(phenyl)methanone (2u)**

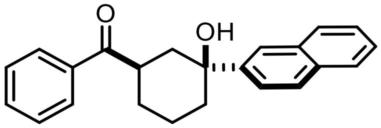

Following GP5 (0.1 mmol scale), the title compound was obtained as a white solid (10.0 mg, 30 µmol, 30%).

^{1}H NMR (400 MHz, $CDCl_3$): δ 8.09 (s, 1H), 7.95 – 7.90 (m, 2H), 7.89 – 7.81 (m, 3H), 7.71 (dd, *J* = 8.7, 1.8 Hz, 1H), 7.60 – 7.54 (m, 1H), 7.51 – 7.45 (m, 4H), 4.61 (s, 1H), 3.78 – 3.67 (m, 1H), 2.44 (dd, *J* = 14.5, 5.2 Hz, 1H), 2.31 – 2.18 (m, 2H), 2.00 – 1.86 (m, 4H), 1.70 – 1.60 (m, 1H) ppm.

^{13}C NMR (101 MHz, $CDCl_3$): δ 205.3, 144.8, 135.8, 133.5, 132.6, 128.9, 128.8, 128.4, 128.2, 127.6, 126.2, 126.0, 124.3, 124.3, 72.2, 42.3, 39.6, 38.8, 28.3, 20.1 ppm.

IR (neat) ν_{max}: 1667, 1661, 1448, 1225, 750, 700 cm^{-1}.

HRMS (ESI$^+$): exact mass calculated for $[M+Na]^+$ ($C_{23}H_{22}O_2Na$) requires *m/z* 353.1512, found *m/z* 353.1507.

***cis*-3-(Dibenzo[*b,d*]furan-2-yl)-3-hydroxycyclohexyl)(phenyl)methanone (2v)**

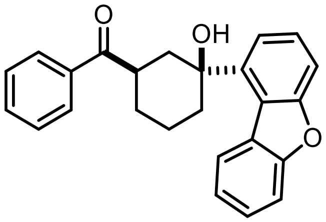

Following GP5 (0.1 mmol scale), the title compound was obtained as a colorless oil (20.7 mg, 56 µmol, 56%).

^{1}H NMR (400 MHz, $CDCl_3$): δ 8.54 (d, *J* = 7.4 Hz, 1H), 7.91 – 7.85 (m, 2H), 7.56 – 7.54 (m, 2H), 7.48 – 7.35 (m, 6H), 7.23 – 7.18 (m, 1H), 4.09 (s, 1H), 3.71 (p, *J* = 5.5 Hz, 1H), 2.60 (t, *J* = 5.2 Hz, 2H), 2.43 – 2.34 (m, 1H), 2.24 – 2.14 (m, 1H), 2.12 – 1.90 (m, 3H), 1.80 – 1.70 (m, 1H) ppm.

^{13}C NMR (101 MHz, $CDCl_3$): δ 204.8, 157.5, 156.4, 143.0, 136.0, 133.3, 128.9 (2C), 128.7 (2C), 127.2, 126.9, 126.7, 123.7, 122.5, 119.7, 111.5, 111.3, 73.2, 42.3, 37.9, 37.7, 28.1, 20.5 ppm.

IR (neat) ν_{max}: 3360, 2925, 1653, 1447, 1416, 1227, 979, 699, 687 cm^{-1}.

HRMS (ESI$^+$): exact mass calculated for $[M+Na]^+$ ($C_{25}H_{22}O_3Na$) requires *m/z* 393.1461, found *m/z* 393.1460.

3.5. Formation of an Unexpected Ring-Contraction Product

cis-1-(2-Benzoyl-1-methylcyclopentyl)ethan-1-one (2y)

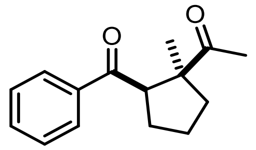

Following GP5 (0.1 mmol scale), the title compound was obtained as a colorless oil (6.3 mg, 27 µmol, 27%) and a single diastereoisomer.

^{1}H NMR (600 MHz, $CDCl_3$): δ 7.93 – 7.87 (m, 2H), 7.56 – 7.52 (m, 1H), 7.44 (t, *J* = 7.8 Hz, 2H), 3.84 (dd, *J* = 9.3, 2.3 Hz, 1H), 2.55 – 2.47 (m, 1H), 2.30 – 2.24 (m, 1H), 2.19 (s, 3H), 1.92 – 1.83 (m, 3H), 1.75 – 1.71 (m, 1H), 1.27 (s, 3H) ppm.

^{13}C NMR (151 MHz, $CDCl_3$): δ 213.3, 202.5, 136.6, 133.0, 128.7 (2C), 128.7 (2C), 59.9, 56.2, 34.9, 29.1, 27.4, 25.6, 22.7 ppm.

IR (neat) ν_{max}: 1700, 1675, 1447, 1353, 1222, 1177, 1107, 1000, 978, 700, 661 cm^{-1}.

HRMS (ESI$^+$): exact mass calculated for $[M+Na]^+$ ($C_{15}H_{18}O_2Na$) requires *m/z* 253.1199, found *m/z* 253.1197.

3.5.1 Proposed Mechanism and Experimental Hints

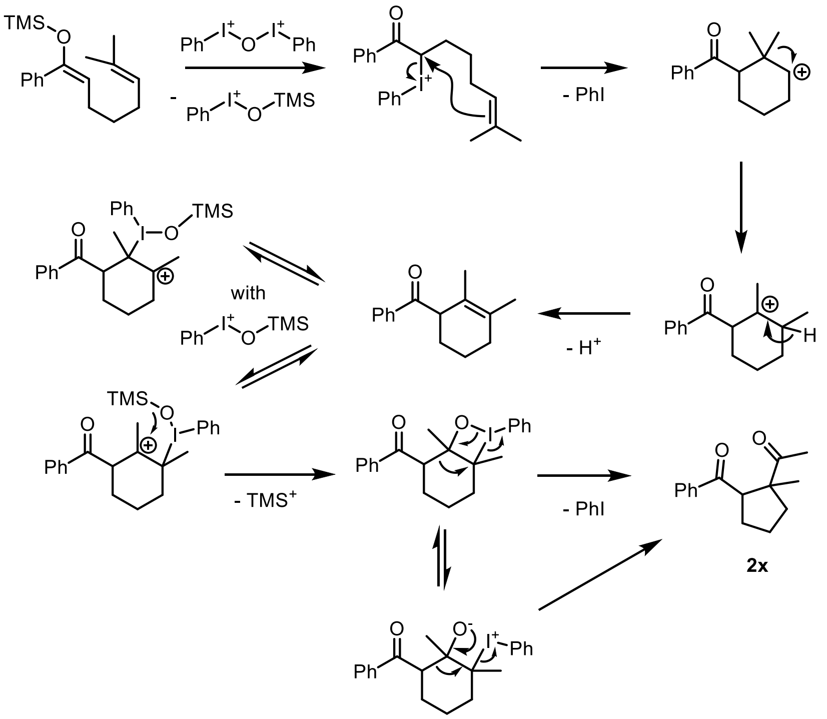

*Scheme S1. Proposed mechanism for the formation of **2x**.*

<u>**Control experiment to prove the olefin intermediate:**</u>

To validate the mechanistic hypothesis presented in Scheme S1, we designed a simpler substrate that we anticipated to react similarly. With this olefin, the ring-contracted product **S2** was also obtained in moderate yield, giving credence to the mechanism proposed in Scheme S1.

2-(4-(Trifluoromethyl)benzoyl)cyclopentane-1-carbaldehyde (S2)

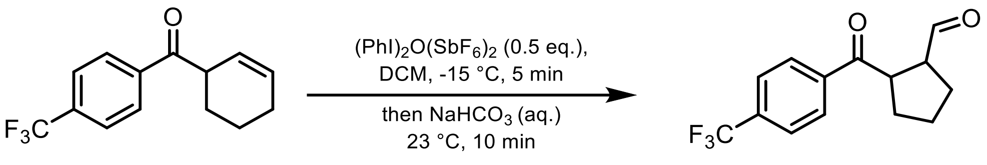

S2

Following GP5 (0.2 mmol scale), **S2** was obtained as a colorless oil (24.32 mg, 0.09 mmol, 45%).

^{1}H NMR (400 MHz, CDCl$_3$): δ 9.75 (s, 1H), 8.10 (d, *J* = 8.1 Hz, 2H), 7.74 (d, *J* = 8.2 Hz, 2H), 4.26 – 4.16 (m, 1H), 3.49 (dt, *J* = 9.5, 5.7 Hz, 1H), 2.19 – 2.04 (m, 2H), 2.02 – 1.90 (m, 1H), 1.86 – 1.75 (m, 2H), 1.71 – 1.59 (m, 1H) ppm.

^{19}F NMR (376 MHz, CDCl$_3$): δ -63.15 ppm.

^{13}C NMR (101 MHz, CDCl$_3$): δ 201.9, 200.0, 134.67 (q, *J* = 32.2 Hz), 129.2 (2C), 125.89 (q, *J* = 3.7 Hz, 2C), 123.72 (q, *J* = 272.9 Hz), 54.0, 45.8, 31.2, 27.3, 25.7.

HRMS (ESI$^+$): exact mass calculated for $[M+H]^+$ ($C_{14}H_{14}F_3O_2$) requires *m/z* 271.0940, found *m/z* 271.0942.

3.6. Unsuccessful Examples of the Umpolung Cyclization

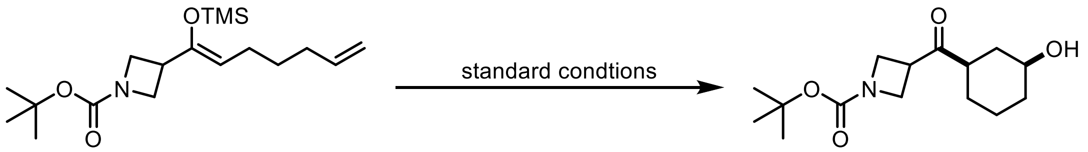

complex mixture

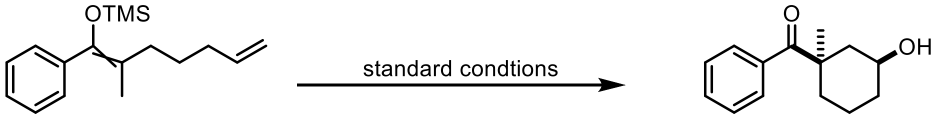

complex mixture

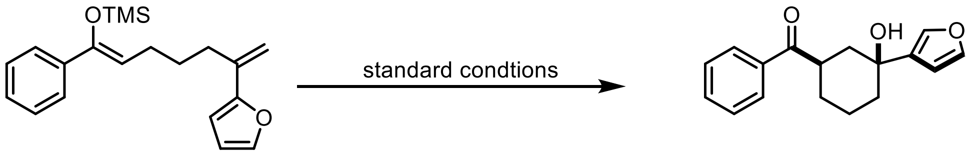

decomposition

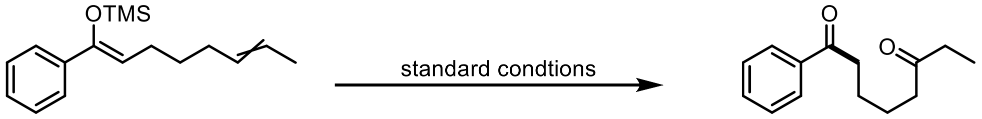

18%

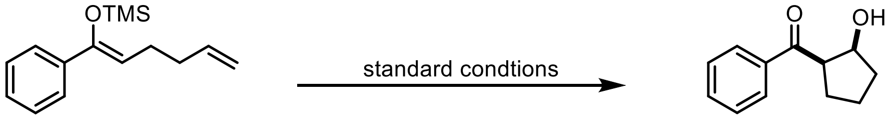

decomposition

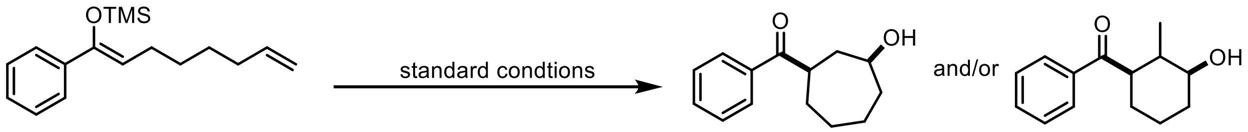

decomposition

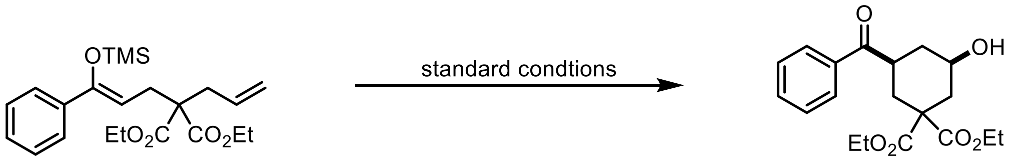

decomposition

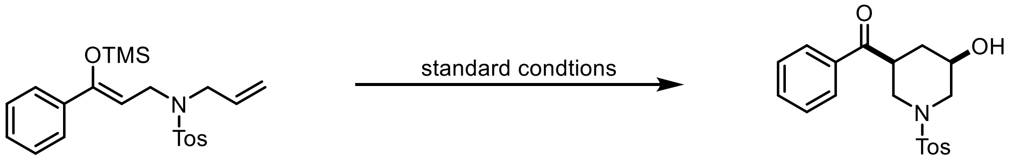

decomposition

4. Experimental Mechanistic Studies

4.1. Detection of oxocarbenium **VII** and TMS_2O (**VIII**)

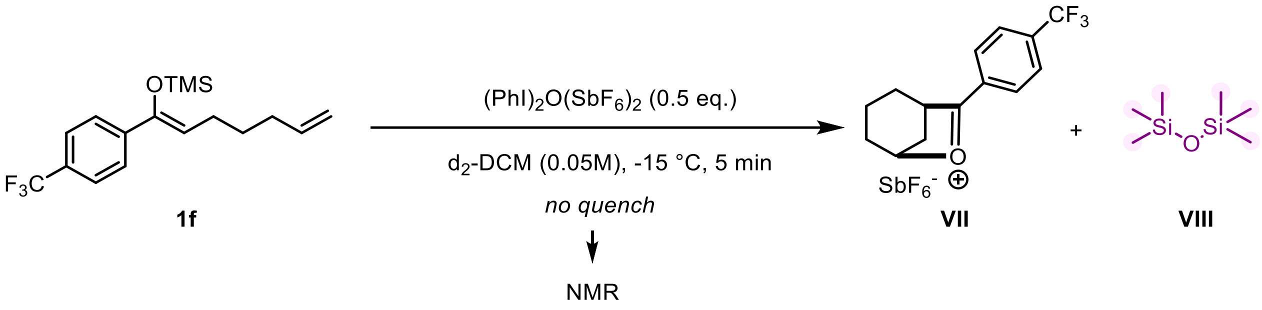

^{13}C NMR (101 MHz, CD_2Cl_2)

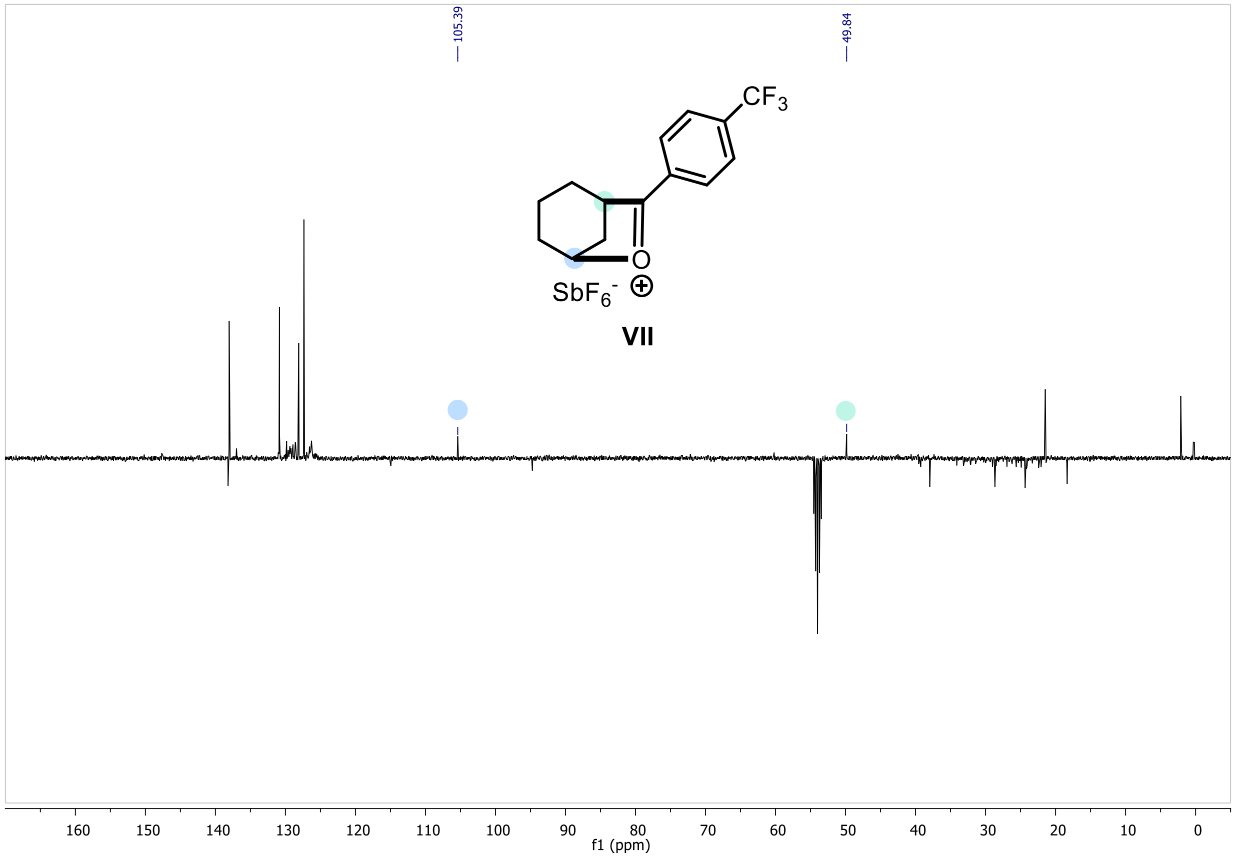

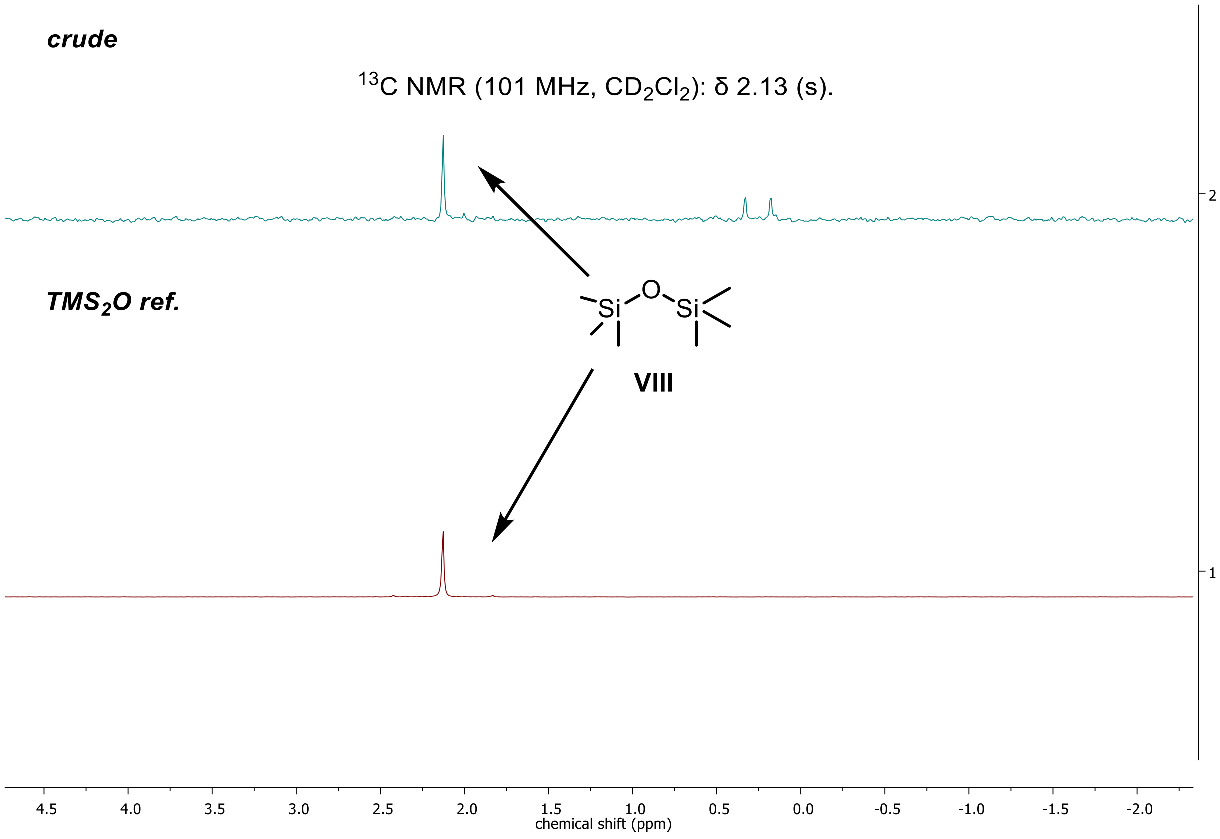

crude
^{13}C NMR (101 MHz, CD_2Cl_2): δ 2.13 (s).
TMS2O ref.
VIII
2
1
4.5
4.0
3.5
3.0
2.5
2.0
1.5
1.0
0.5
0.0
-0.5
-1.0
-1.5
-2.0
chemical shift (ppm)

4.2. Umpolung Cyclization with **1f-d2**

Preparation of substrate 1f-d2:

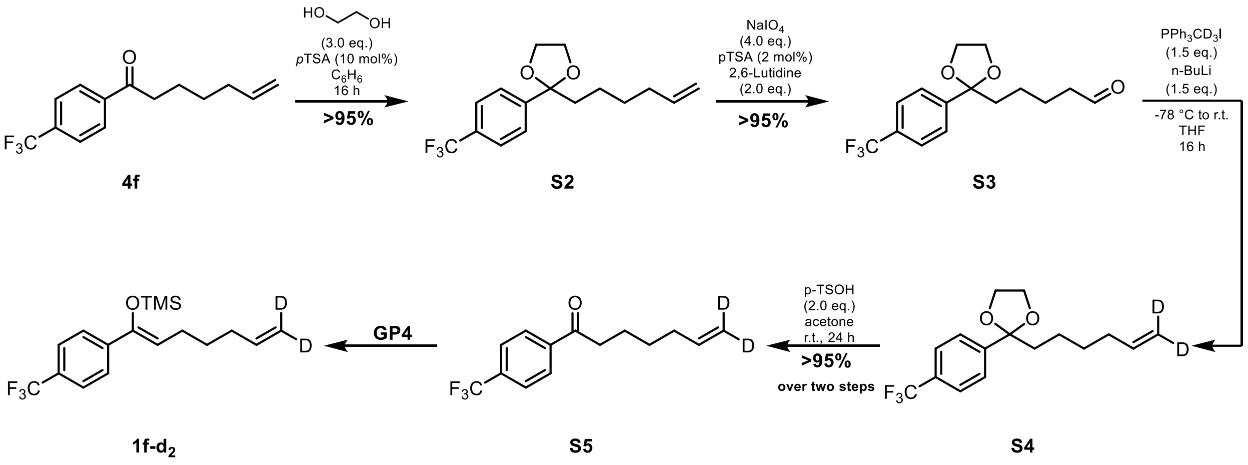

Compound **1f-d2** was prepared using a previously slightly modified procedure.[26]

1-(4-(Trifluoromethyl)phenyl)hept-6-en-1-one (1.0 eq., 0.37 mmol, 95.1 mg) and ethylene glycol (3.0 eq., 1.11 mmol, 62 μL) were dissolved in benzene (2 mL). *p*-TSA (10 mol%, 0.04 mmol, 7.5 mg) was added to the solution, and the mixture was heated at reflux (oil bath temperature *ca*. 95 °C) using a Dean-Stark apparatus and a condenser. After 16 h, the reaction was allowed to cool to room temperature and a saturated aqueous solution of $NaHCO_3$ was added (25 mL). The separated aqueous layer was extracted with Et_2O (3 x 10 mL) and the combined organic phases were dried over Na_2SO_4 and filtered. After removing the solvent under reduced pressure, the ketal **S2** was obtained as a colourless liquid (110 mg, >95% yield).

The crude ketal (1.0 eq., 0.36 mmol, 108 mg) was dissolved in a mixture of 1,4-dioxane (3.75 mL) and H_2O (1.25 mL). Lutidine (2.0 eq., 0.71 mmol, 80 μL), sodium periodate (4.0 eq., 1.44 mmol, 0.5 mg) and a solution of OsO_4 in H_2O (2 mol%, 4 w/w%, 46 μL) were added in that order and the resulting solution was stirred until full consumption of the starting material was observed by TLC (3 h). The reaction was stopped by addition of H_2O (10 mL) and a saturated aqueous solution of $Na_2S_2O_4$ (10 mL). CH_2Cl_2 (10 mL) was added, the phases were separated and the aqueous layer was further extracted with CH_2Cl_2 (3 x 10 mL). The combined organic layers were dried over Na_2SO_4, filtered and volatiles were removed under reduced pressure. The crude mixture was purified by column chromatography (heptanes:EtOAc, 100:0 to 50:50 v/v%) to yield the aldehyde **S3** as a yellow liquid (110 mg, >95% yield).

The Wittig salt (PPh_3CD_3I, 1.5 eq., 0.525 mmol, 213 mg) was suspended in THF (11 mL) and cooled to 0 °C. A solution of *n*-BuLi (1.5 eq., 0.525 mmol, 33.7 mg, 0.200 mL, 2.5 M in hexane) in THF was added dropwise and the reaction mixture was stirred until the solution became clear. Then the mixture was cooled to -78 °C and a solution of the aldehyde (1.0 eq., 0.35 mmol, 106 mg) in THF (2 mL) was added. The mixture was warmed to room temperature and stirred until no aldehyde was observable by TLC. Thereafter, a saturated aqueous solution of NH_4Cl was added (25 mL). Then the aqueous phase was extracted with more Et_2O (3 x 10 mL), the combined organic layers were dried over Na_2SO_4, filtered, and the volatiles were removed under reduced pressure. Column chromatography (heptanes:EtOAc, 100:0 to 60:40) gave the compound **S4** as a colorless liquid. The latter was then suspended in acetone (3 mL) and *p*-TsOH (2.0 eq., 0.70 mmol, 133 mg) was added. The mixture was stirred at 25 °C for 16 h. Then, saturated aqueous $NaHCO_3$ solution (25 mL) was added and the aqueous phase was extracted with EtOAc (3 x 10 mL). The combined organic phases were dried over Na_2SO_4, filtered, and the volatiles were removed under reduced pressure. The crude material was directly used in the next step without further purification.

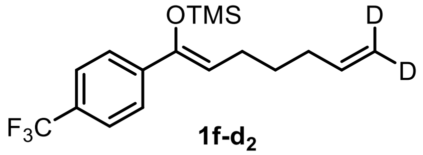

1f-d$_2$

A vial was loaded with ketone **S5** (1.0 eq., 0.35 mmol, 65 mg), triethylamine (5.0 eq., 1.71 mmol, 173 mg, 2.35 mL), and DCM (0.2 M). The solution was cooled to 0 °C and TMSOTf (3.0 eq., 1.02 mmol, 232 mg, 0.829 mL) was added dropwise. The resulting mixture was allowed to warm to room temperature and subsequently stirred for 16 h before stopping the reaction by addition of a saturated aqueous solution of NH_4Cl (25 mL). The aqueous was extracted with DCM (2 x 10 mL). The combined organic phases were dried over $MgSO_4$ and filtered. After evaporation of the organic solvent, the crude material was further purified by column chromatography (heptanes:EtOAc, 100:0 to 95:5) to yield the **1f-d$_2$** as a yellow liquid (0.16 mmol, 43.0 mg, 48%).

^{1}H NMR (400 MHz, CDCl$_3$): δ 7.59 – 7.52 (m, 4H), 5.86 – 5.79 (m, 1H), 5.39 – 5.31 (m, 1H), 2.26 – 2.18 (m, 2H), 2.16 – 2.07 (m, *J* = 14.5, 7.1 Hz, 2H), 1.59 – 1.47 (m, 2H), 0.14 (s, 9H) ppm.
^{13}C NMR (101 MHz, CDCl$_3$): δ 148.1, 142.8, 138.6, 129.5, 125.7, 125.5 (2C), 125.21 (q, *J* = 3.9 Hz, 2C), 113.5, 113.0 – 112.9 (m), 33.6, 28.9, 25.9, 0.7 (3C) ppm.
^{19}F NMR (376 MHz, CDCl$_3$). δ -62.43 ppm.

IR (neat) ν_{max}: 2930, 1641, 1615, 1411, 1321, 1247, 1120, 1067, 885, 753 cm^{-1}.

HRMS (ESI$^+$): exact mass calculated for $[M+H]^+$ ($C_{17}H_{22}D_2F_3OSi$) requires m/z 331.1669, found m/z 331.1674.

Cyclization of **1f-d$_2$:**

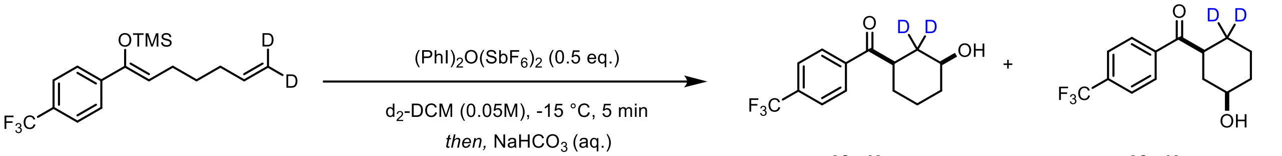

1f-d2 **2f$_1$-d2** *a* **2f$_2$-d2** *b*

Following GP4 (0.1 mmol scale), the deuterated product afforded a mixture of **2f$_1$-d$_2$** (a) and **2f$_2$-d$_2$** (b) (15.2 mg, 0.055 mmol, 54%) in a 2:1 ratio, respectively.

^{1}H NMR (400 MHz, CDCl$_3$) δ 8.03 (d, *J* = 8.1 Hz, 2H), 7.74 (d, *J* = 8.2 Hz, 2H), 3.84 – 3.68 (m, 1H), 3.31 (dd, *J* = 22.6, 9.7 Hz, 1H), 2.21 – 2.08 (m, 0.5H), 2.09 – 1.97 (m, 1H), 1.98 – 1.80 (m, 2H), 1.79 – 1.37 (m, 2H), 1.36 – 1.18 (m, 2H), 0.91 – 0.79 (m, 1H) ppm. *The OH protons were not observed.*

^{13}C NMR (176 MHz, CDCl$_3$) δ 201.5 (*a* + *b*), 134.5 (q, *J* = 32.7 Hz, *a* + *b*), 138.9 (*a* + *b*), 128.7 (2C, *a* + *b*), 125.9 (dd, *J* = 7.2, 3.6 Hz, 2C, *a* + *b*), 124.8 (q, *J = it cannot be calculated due to signal overlap, a + b*), 70.1 (*b*), 69.9 (*a*), 44.4 (*a* + *b*), 37.6 (*b*), 37.22 – 36.55 (m, *CD$_2$*-**2f$_1$-d$_2$,** *a*), 35.3 (*a* + *b*), 28.4 (*a*), 27.94 – 27.39 (m, *CD$_2$*-**2f$_2$-d$_2$,** *b*), 23.5 (*a*), 23.3 (*b*) ppm.

^{19}F NMR (376 MHz, CDCl3) δ -63.13 ppm.

HRMS (ESI+): exact mass calculated for $[M+Na]^+$ ($C_{14}H_{13}D_2F_3O_2Na$) requires m/z 297.1042, found m/z 297.1037.

IR (neat) ν_{max}: 3357, 2933, 2859, 1684, 1510, 1449, 1409, 1322, 1127, 1112, 697, 814, 592 cm^{-1}.

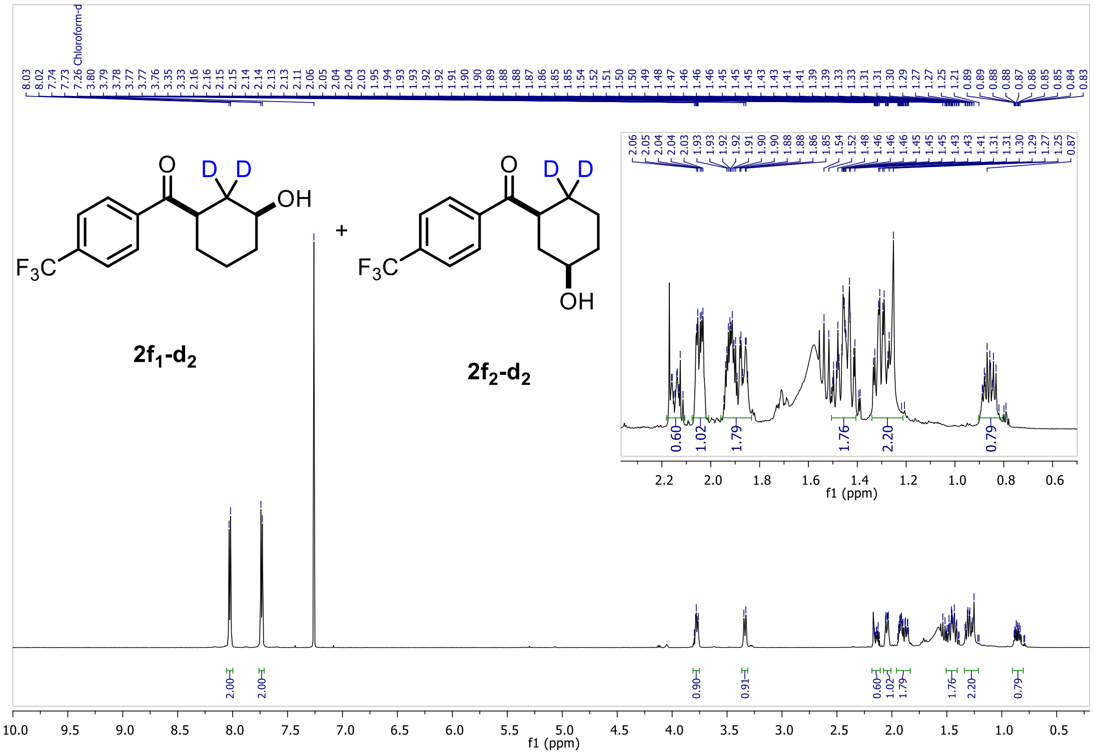
2f1-d2
2f2-d2
f1 (ppm)

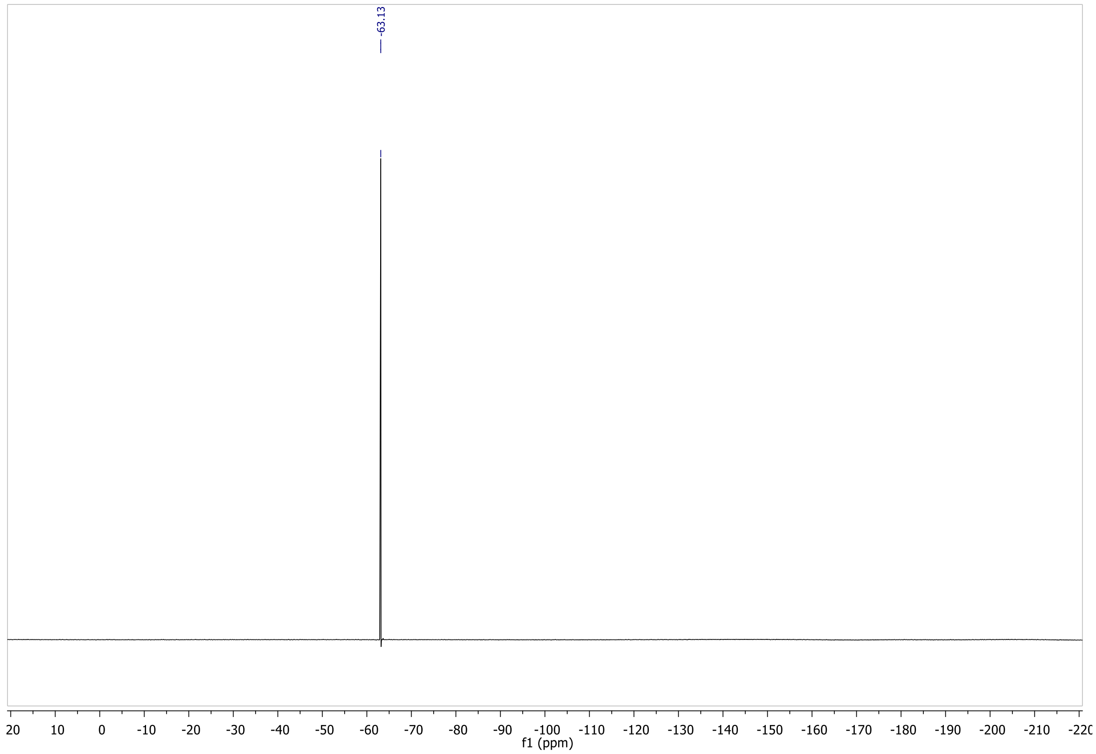
-63.13
f1 (ppm)

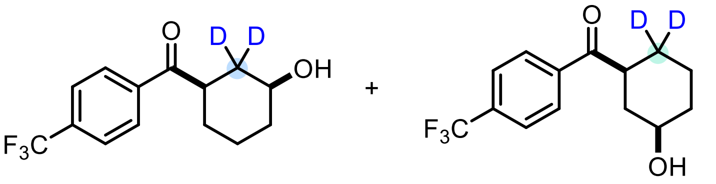
O
D D
OH
F3C
+
O
D D
F3C
OH

2f₁-d₂ **2f₂-d₂**

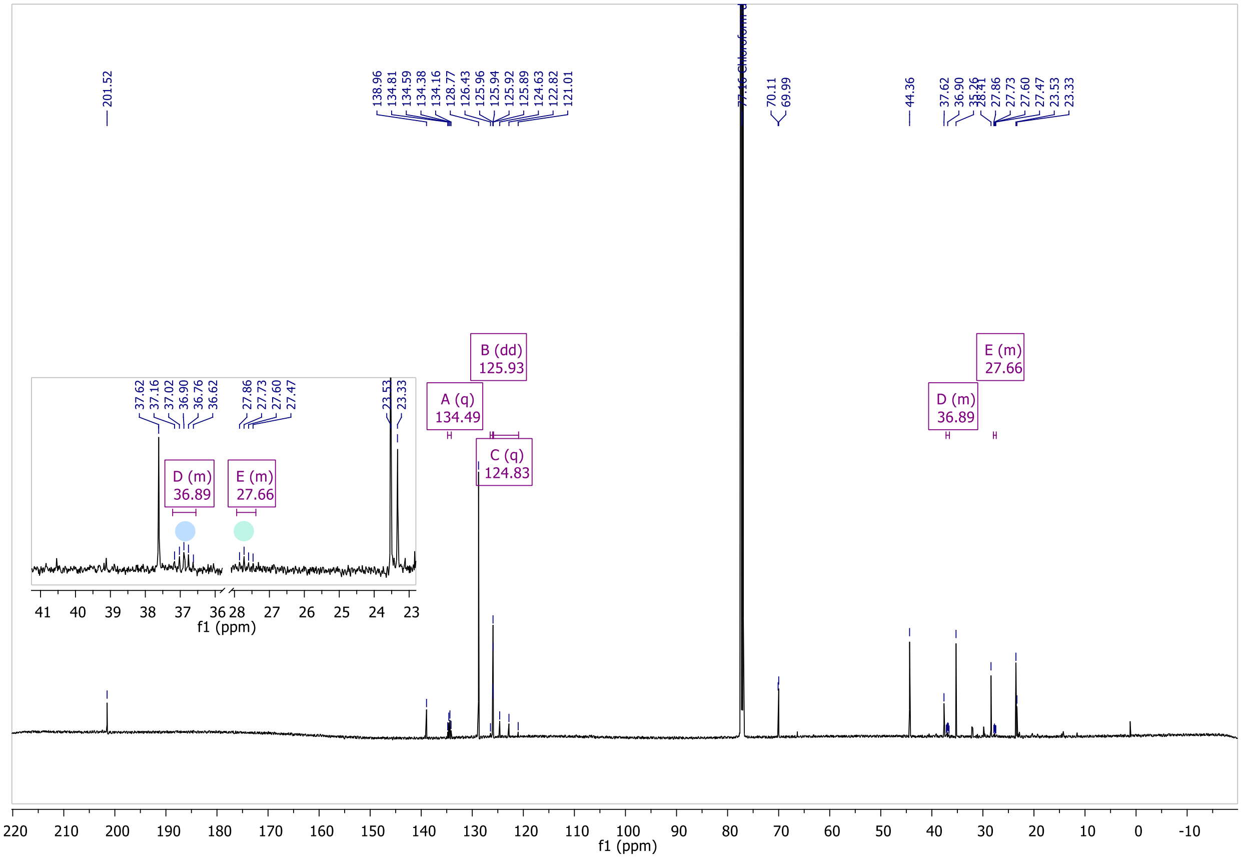
201.52
138.96
134.81
134.59
134.38
134.16
128.77
126.43
125.96
125.94
125.92
125.89
124.63
122.82
121.01
70.11
69.99
44.36
37.62
36.90
27.86
27.73
27.60
27.47
23.53
23.33
A (q)
134.49
B (dd)
125.93
C (q)
124.83
D (m)
36.89
E (m)
27.66
37.62
37.16
37.02
36.90
36.76
36.62
f1 (ppm)

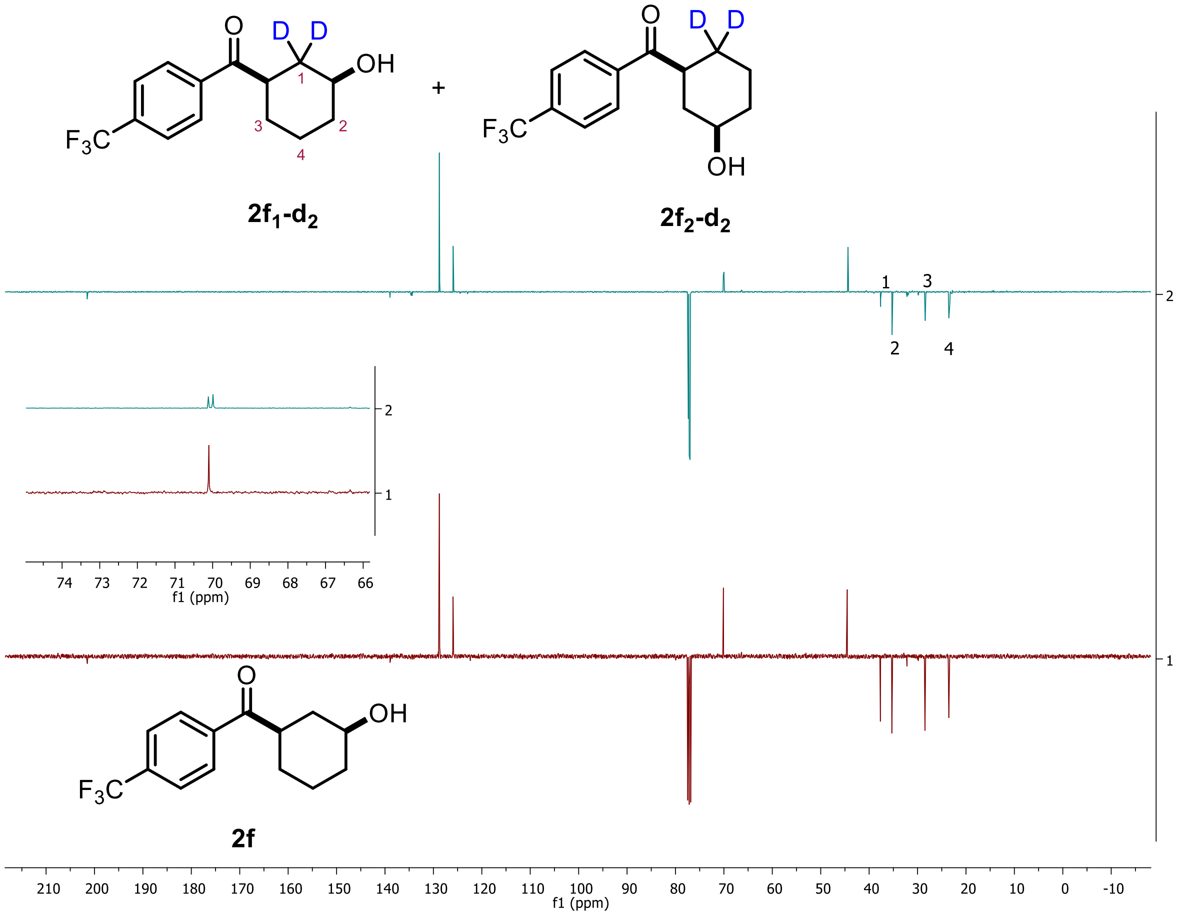

2f1-d2
2f2-d2
2f
1
2
3
4
f1 (ppm)

5. DFT calculations

The conformational space of all molecules has been initially searched using meta-dynamics simulations based on tight-binding quantum chemical calculations as implemented in the software package conformer-rotamer ensemble sampling tool CREST.[27,28] The structures located with CREST have then been subjected to PBE0-D3(BJ)/def2-SVP geometry optimization.[29–33] The nature of all stationary points (minima and transition states) was verified through the computation of the vibrational frequencies. The thermal corrections to the Gibbs free energies were combined with the single point energies calculated at the PBE0-D3(BJ)/def2-TZVP level of theory to yield Gibbs free energies ("G_{298}") at 298.15 K. All energies are reported in kcal·mol^{-1}. The energy profiles were constructed using the most stable conformation (the global minimum) of each intermediate and transition state. The density functional theory (DFT) calculations have been performed with the Gaussian 16 program package.[34] The polarizable continuum model (PCM) with SMD parameters, including refined iodine parameters (SMD18),[35] for dichloromethane,[36–38] was applied to consider solvent effects for both geometries and energies. Free energies in solution have been corrected to a reference state of 1 mol·l^{-1} at 298.15 K through the addition of RTln(24.46) = +7.925 kJ·mol^{-1} to the gas phase (1 atm) free energies.

5.1. Thermodynamic comparison of I–C and I–O interactions

In this work, we have computationally studied the mechanism for the formation of an oxocarbenium intermediate, which undergoes hydrolysis to form a hydroxylated product. As discussed in the main text, the reaction of the silyl enol ether substrate with the used hypervalent iodine reagent, $I(OPh)_2^{2+}$, is promoted through an initial I–C bond formation. It was previously observed by the Szpilman group that an I–O interaction can be preferential with the use of PhI(OH)(OTs) as a hypervalent iodine reagent.[39] We have therefore computed the relative thermodynamic stability of the possible I-O and I-C interactions considering all four combinations of the silyl enol ether and the iodine reagent studied in this work and the silyl enol ether and iodine reagent reported by Szpilman and co-workers (Figure SI-1). In the four presented systems, the intermediates featuring an I–C bond are more stable than the alternative structure with an I–O bond. Specifically, **SI-A** is 20.4 kcal/mol more stable than **SI-A',** and **SI-B** is 25.1 kcal/mol more stable than **SI-B'** (Figure SI-1-A). Similarly, in the neutral system, **SI-C** is 8.7 kcal/mol more stable than **SI-C',** and **SI-D** is 5.7 kcal/mol more stable than **SI-D'** (Figure SI-1-B). The increased stability of the intermediates presenting an I–C bond is highly accentuated by the electron-deficient nature of the cationic iodine reagent used in this work.

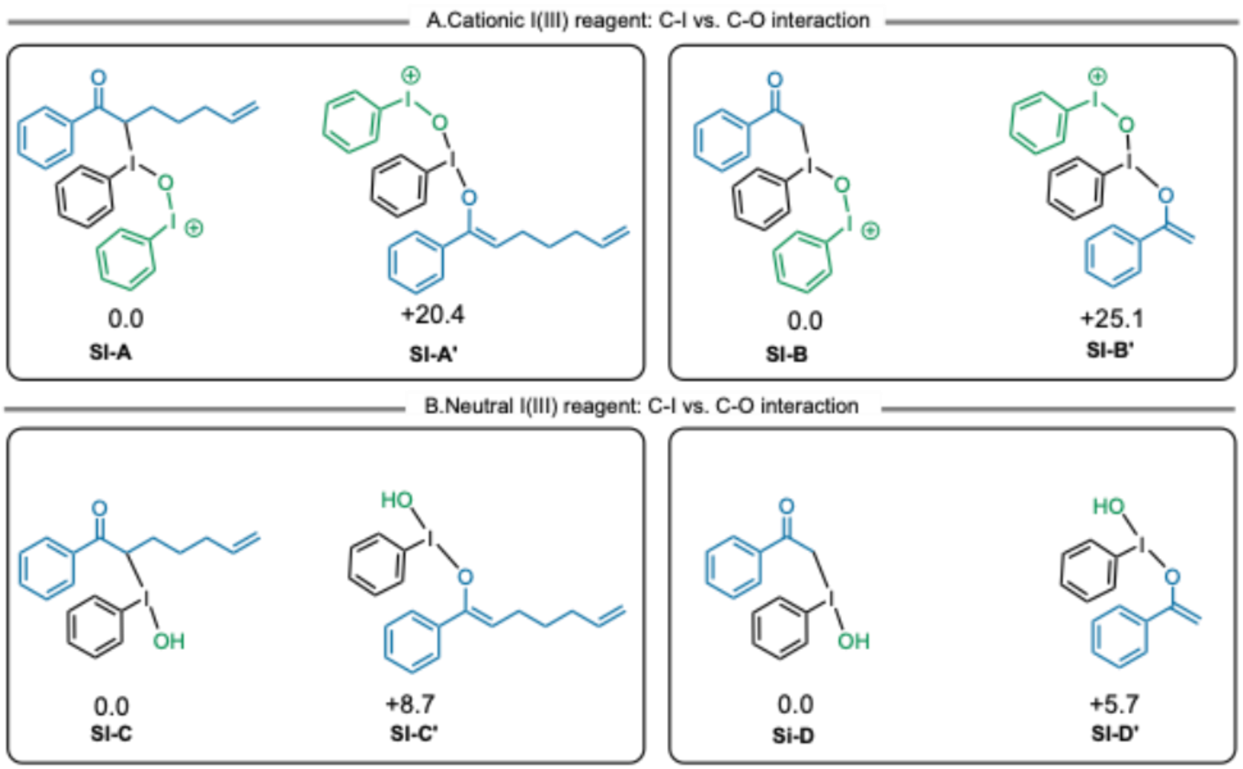

Figure SI-1 *– Thermodynamic comparison of key intermediates presenting either C-I or C-O bond. A) considering the hypervalent iodine reagent studied in this work; B) Considering the neutral hypervalent iodine reagent studied by Szpilman and co-workers. Relative Gibbs free energies are presented in kcal/mol.*

5.2. The influence of the counterion on the reaction mechanism

In some cases, the inclusion of counterions in quantum chemical calculations of charged species can be crucial. However, for the studied mechanism, we do not expect any dramatic effect from the counterion, as it cannot form strong intermolecular interactions with either the intermediates or the transition states due to its chemical nature (SBF_6^-). Nevertheless, we have assessed the influence of the counterion by repeating the calculations for the **C→D** step, this time including SBF_6^-. Figure SI-2 shows the resulting pathway comparison: the black line represents the **C→D** step without the counterion, while the red line shows the pathway with it included. Naturally, various possible relative positions of the ions were considered through a full conformational search. Although we observe some quantitative differences in the characteristics of the Gibbs free energy reaction profile, the overall mechanism remains unchanged. The transformation proceeds as a concerted process that is highly favorable both kinetically and thermodynamically, regardless of whether the counterion is included.

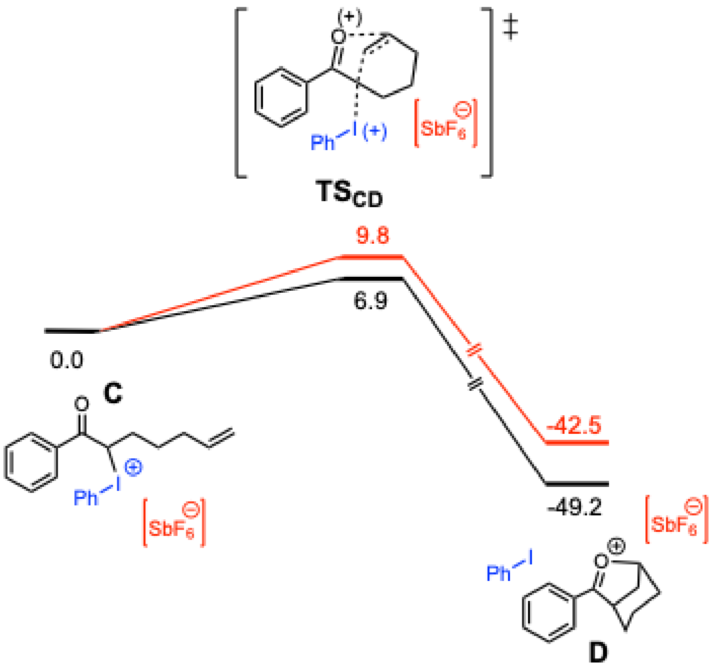

Figure SI-2 *– Computed energy profiles (relative Gibbs free energies in kcal/mol) for step **C→D** without considering counterion (shown in black) and involving the counterion (shown in red color) in comparison. The intermediate **C** is taken as the reference (0.0 kcal/mol).*

5.3. XYZ Structures

Cartesian coordinates of the most stable (ΔG_{298}) conformations, computed at the PBE0-D3(BJ),SMD(DCM)/def2-TZVP//PBE0-D3(BJ)/def2-SVP level of theory. For the structures **SI-A**, **SI-A'**, **SI-B**, **SI-B'**, **SI-C**, **SI-C'**, **SI-D** and **SI-D'** the SMD model was also applied during geometry optimization.

25

OIPh2

I	-4.252823000	1.004455000	-0.447131000
C	-5.181988000	1.009637000	1.381106000
C	-5.291764000	2.227677000	2.081800000
C	-5.732496000	-0.201631000	1.846171000
C	-5.986781000	2.218578000	3.281341000
C	-6.415191000	-0.175616000	3.052898000
C	-6.542083000	1.025135000	3.764075000
H	-4.865175000	3.155302000	1.694325000
H	-5.637088000	-1.131086000	1.280566000
H	-6.104184000	3.146815000	3.845090000
H	-6.860157000	-1.094780000	3.440751000
H	-7.088851000	1.032703000	4.710597000
O	-2.407523000	0.271372000	-0.022916000
I	-0.706132000	1.306292000	0.372900000
C	0.214951000	1.404726000	-1.456818000
C	0.955580000	0.284688000	-1.885198000
C	0.128156000	2.602134000	-2.195310000
C	1.628606000	0.383414000	-3.093591000
C	0.819009000	2.668439000	-3.395467000
C	1.561569000	1.566229000	-3.842028000
H	1.010176000	-0.629952000	-1.290766000
H	-0.444098000	3.460039000	-1.835974000

H	2.217611000	-0.463185000	-3.453637000
H	0.786272000	3.585484000	-3.988070000
H	2.102653000	1.632820000	-4.789503000

42

Sub

C	2.41820	0.15838	0.16051
O	3.00727	-0.60393	1.11754
C	1.99972	1.51554	0.57731
C	2.03299	2.59964	-0.31156
C	1.56335	1.73870	1.89112
C	1.62858	3.86695	0.09848
C	1.15938	3.00658	2.30039
C	1.19035	4.07614	1.40612
H	2.40349	2.44591	-1.32769
H	1.53239	0.89745	2.58597
H	1.66868	4.70197	-0.60525
H	0.81339	3.16029	3.32559
H	0.87891	5.07242	1.72872
C	2.18683	-0.34550	-1.06577
H	1.64280	0.28624	-1.77156
C	2.61687	-1.71432	-1.49626
H	2.47179	-2.41594	-0.65883
C	4.08141	-1.79327	-1.93901
H	4.72914	-1.57839	-1.07417
H	4.31407	-2.82890	-2.24280
C	4.44396	-0.83205	-3.06901
H	3.81955	-1.06441	-3.95195
H	4.19280	0.19871	-2.76814
C	5.88928	-0.90478	-3.44429
H	6.23889	-1.87098	-3.83285
C	6.76924	0.08947	-3.31292
H	6.46703	1.06875	-2.92614
H	7.81995	-0.03324	-3.58878
Si	4.53360	-0.34949	1.82815
C	4.31458	0.37024	3.54253
H	3.61438	-0.23763	4.13691
H	5.28016	0.38670	4.07342
H	3.92885	1.39974	3.50201
C	5.52233	0.80923	0.74067
H	5.01157	1.78005	0.64320
H	6.51494	0.99081	1.18285
H	5.67276	0.39776	-0.26914
C	5.29929	-2.05046	1.94771
H	5.45285	-2.49303	0.95197
H	6.27473	-2.00757	2.45756
H	4.64526	-2.72568	2.52127
H	1.97053	-2.05848	-2.32050

67

A

C	-2.467873000	0.043309000	-0.198598000
O	-3.135665000	-0.612993000	-1.056248000
C	-2.200836000	1.462493000	-0.352846000
C	-1.925090000	2.284611000	0.759546000
C	-2.179727000	2.022990000	-1.646525000
C	-1.654888000	3.633146000	0.578033000
C	-1.886976000	3.366644000	-1.822112000
C	-1.631821000	4.173993000	-0.709644000
H	-1.970917000	1.883838000	1.773231000
H	-2.353072000	1.385149000	-2.513692000
H	-1.472300000	4.272069000	1.444030000

H	-1.856377000	3.792729000	-2.827010000
H	-1.417674000	5.236484000	-0.848270000
C	-1.885331000	-0.721872000	0.908423000
H	-1.574958000	-0.076949000	1.736766000
C	-2.609942000	-1.981085000	1.339523000
H	-2.653667000	-2.698613000	0.503789000
C	-4.035845000	-1.697391000	1.821007000
H	-4.656655000	-1.383884000	0.967402000
H	-4.470271000	-2.648650000	2.167954000
C	-4.153487000	-0.646216000	2.923247000
H	-3.568485000	-0.970505000	3.803513000
H	-3.720403000	0.313460000	2.589762000
C	-5.578860000	-0.407466000	3.315084000
H	-6.106883000	-1.258599000	3.762843000
C	-6.230771000	0.741915000	3.136394000
H	-5.737360000	1.620016000	2.704249000
H	-7.275823000	0.859597000	3.432768000
Si	-4.629547000	-0.482778000	-2.077520000
C	-4.052499000	-0.193083000	-3.818638000
H	-3.209230000	-0.850175000	-4.081893000
H	-4.882996000	-0.451750000	-4.496413000
H	-3.778675000	0.849936000	-4.031506000
C	-5.593528000	0.897647000	-1.298004000
H	-5.097516000	1.874758000	-1.390981000
H	-6.567152000	0.972178000	-1.810561000
H	-5.800133000	0.700832000	-0.233903000
C	-5.364185000	-2.167741000	-1.857603000
H	-5.647781000	-2.373914000	-0.815532000
H	-6.278102000	-2.242833000	-2.469425000
H	-4.673720000	-2.953940000	-2.199313000
I	3.897829000	-1.923411000	-0.065335000
C	4.644692000	0.033945000	-0.128343000
C	4.784589000	0.735549000	1.069983000
C	5.025482000	0.557834000	-1.365764000
C	5.342438000	2.011552000	1.017488000
C	5.560328000	1.843274000	-1.392261000
C	5.724817000	2.561686000	-0.205939000
H	4.480815000	0.302459000	2.024843000
H	4.920516000	-0.018996000	-2.286994000
H	5.483332000	2.574476000	1.942827000
H	5.874005000	2.274855000	-2.345398000
H	6.169499000	3.559014000	-0.235744000
O	2.167147000	-1.756550000	-0.899868000
I	0.158907000	-1.340387000	0.065066000
C	0.966710000	0.551302000	0.524005000
C	1.087663000	0.922123000	1.861089000
C	1.361051000	1.369279000	-0.531112000
C	1.602056000	2.187767000	2.144298000
C	1.874200000	2.627624000	-0.221692000
C	1.988160000	3.036540000	1.107305000
H	0.795206000	0.252153000	2.673118000
H	1.281668000	1.037743000	-1.567478000
H	1.706257000	2.503615000	3.184932000
H	2.188881000	3.290215000	-1.030785000
H	2.390708000	4.025270000	1.337950000
H	-2.029093000	-2.457732000	2.145293000

67

A'

C	1.94397	0.85459	-0.36428
O	1.58971	0.48687	0.92626
C	1.12610	1.93950	-0.93199
C	0.98896	2.08907	-2.32402

C	0.44245	2.83795	-0.09555
C	0.22160	3.11931	-2.85667
C	-0.32812	3.86707	-0.62992
C	-0.43648	4.01559	-2.01184
H	1.49219	1.39549	-3.00126
H	0.53417	2.75098	0.98808
H	0.14255	3.22949	-3.94048
H	-0.82984	4.57004	0.03879
H	-1.02037	4.83717	-2.43361
C	2.91089	0.17268	-1.01318
H	3.19046	0.56697	-1.99397
C	3.64441	-1.02928	-0.53843
H	3.16443	-1.43532	0.36319
C	5.14122	-0.80481	-0.28358
H	5.27584	-0.08149	0.53775
H	5.56724	-1.75522	0.07828
C	5.93259	-0.32495	-1.49876
H	5.76788	-1.03125	-2.33394
H	5.56197	0.65743	-1.83707
C	7.39865	-0.22123	-1.21165
H	7.90636	-1.15847	-0.94989
C	8.10221	0.91033	-1.24251
H	7.63925	1.86718	-1.50687
H	9.17183	0.92398	-1.01970
Si	2.51383	0.93864	2.41909
C	1.17359	1.51520	3.57933
H	0.49103	0.70951	3.89271
H	1.66314	1.87301	4.50104
H	0.58483	2.35990	3.19055
C	3.66602	2.28477	1.88382
H	3.12979	3.13373	1.43440
H	4.19900	2.65864	2.77374

H	4.42143	1.93065	1.16831
C	3.30936	-0.61682	3.04340
H	4.15257	-0.94827	2.42216
H	3.70144	-0.42429	4.05593
H	2.58532	-1.44184	3.13081
I	-3.65315	-1.54484	-0.24311
C	-4.24141	0.44574	-0.31153
C	-3.54734	1.31835	-1.16067
C	-5.31309	0.85110	0.49411
C	-3.95090	2.64878	-1.19113
C	-5.69616	2.18722	0.43706
C	-5.01962	3.07816	-0.40005
H	-2.72102	0.97858	-1.78845
H	-5.84038	0.15018	1.14451
H	-3.42938	3.34997	-1.84526
H	-6.53348	2.53199	1.04777
H	-5.33595	4.12306	-0.44081
O	-2.34946	-1.49983	1.23977
I	-0.51037	-0.50339	1.07200
C	0.21099	-1.90975	-0.28381
C	0.63563	-3.14736	0.20665
C	0.23917	-1.56971	-1.63912
C	1.10501	-4.08013	-0.71282
C	0.71712	-2.52446	-2.53621
C	1.14828	-3.76825	-2.07509
H	0.60015	-3.38612	1.27141
H	-0.09030	-0.58971	-1.99030
H	1.44138	-5.05802	-0.36114
H	0.75198	-2.29050	-3.60256
H	1.52137	-4.50894	-2.78615
H	3.54337	-1.80138	-1.32389

67

TSAB

C	-2.105979000	-0.068220000	-0.298117000
O	-2.113844000	-1.312169000	-0.463572000
C	-1.645703000	0.877354000	-1.284513000
C	-1.930786000	2.258836000	-1.171100000
C	-0.854839000	0.426816000	-2.366210000
C	-1.448782000	3.150616000	-2.115230000
C	-0.365597000	1.325846000	-3.297058000
C	-0.665611000	2.687225000	-3.175637000
H	-2.562017000	2.640856000	-0.368508000
H	-0.586544000	-0.625691000	-2.440571000
H	-1.689612000	4.212020000	-2.033956000
H	0.254985000	0.974259000	-4.123508000
H	-0.287255000	3.394161000	-3.918200000
C	-2.526940000	0.380212000	1.070038000
H	-2.741676000	1.450591000	1.132897000
C	-3.541919000	-0.488560000	1.777817000
H	-3.149506000	-1.510286000	1.899351000
C	-4.863608000	-0.549552000	1.001057000
H	-4.723862000	-1.116855000	0.066120000
H	-5.566150000	-1.143864000	1.606926000
C	-5.485506000	0.808753000	0.679584000
H	-5.590822000	1.389837000	1.614722000
H	-4.823549000	1.392656000	0.014628000
C	-6.821427000	0.672035000	0.016659000
H	-7.609096000	0.197473000	0.615111000
C	-7.096804000	1.070835000	-1.224650000
H	-6.342643000	1.557826000	-1.852790000
H	-8.091230000	0.942096000	-1.658523000
Si	-1.962676000	-2.824906000	-1.455631000
C	-0.126703000	-3.021355000	-1.663197000
H	0.403636000	-2.528469000	-0.825351000
H	0.114738000	-4.096383000	-1.642882000
H	0.245007000	-2.621928000	-2.618836000
C	-2.968926000	-2.478469000	-2.973951000
H	-2.556954000	-1.677062000	-3.603684000
H	-2.998944000	-3.395852000	-3.585731000
H	-4.008917000	-2.225603000	-2.715892000
C	-2.723657000	-4.051369000	-0.299388000
H	-3.766932000	-3.796100000	-0.059379000
H	-2.729044000	-5.047916000	-0.770430000
H	-2.150059000	-4.131783000	0.636525000
I	3.200571000	-1.954304000	0.657552000
C	4.195436000	-0.394573000	-0.356882000
C	4.882410000	0.563190000	0.390550000
C	4.142984000	-0.355092000	-1.751683000
C	5.536745000	1.590632000	-0.288148000
C	4.798690000	0.683966000	-2.410809000
C	5.494445000	1.650160000	-1.681349000
H	4.920109000	0.510928000	1.480575000
H	3.619199000	-1.127483000	-2.318993000
H	6.092098000	2.341423000	0.278378000
H	4.786672000	0.723434000	-3.502489000
H	6.022487000	2.449606000	-2.206115000
O	1.396204000	-1.339928000	0.770106000
I	-0.579400000	0.240262000	2.171460000
C	0.357518000	1.883953000	1.243741000
C	0.056055000	3.168263000	1.697749000
C	1.243054000	1.610977000	0.205902000
C	0.669621000	4.234727000	1.042799000
C	1.845122000	2.698804000	-0.424262000
C	1.556253000	4.000151000	-0.011182000

H	-0.620661000	3.345047000	2.536360000
H	1.457304000	0.570961000	-0.060409000
H	0.462914000	5.255260000	1.373030000
H	2.556937000	2.516604000	-1.232915000
H	2.039276000	4.846694000	-0.504553000
H	-3.716615000	-0.073728000	2.782817000

67

B

C	1.844126000	-0.070589000	0.173865000
O	2.007741000	-1.292228000	-0.054253000
C	1.259769000	0.459806000	1.381592000
C	1.512539000	1.784953000	1.808216000
C	0.389128000	-0.356123000	2.138635000
C	0.932776000	2.263520000	2.971876000
C	-0.200861000	0.138879000	3.289092000
C	0.076688000	1.442878000	3.711944000
H	2.201566000	2.433031000	1.264920000
H	0.119319000	-1.339405000	1.757231000
H	1.153100000	3.276477000	3.313370000
H	-0.889119000	-0.486020000	3.861593000
H	-0.380037000	1.825505000	4.628088000
C	2.292477000	0.850301000	-0.924413000
H	2.497618000	1.859272000	-0.552721000
C	3.366419000	0.328885000	-1.852840000
H	3.016298000	-0.577244000	-2.371188000
C	4.657510000	-0.001530000	-1.090045000
H	4.514065000	-0.920888000	-0.503038000
H	5.422977000	-0.246656000	-1.843285000
C	5.176738000	1.099981000	-0.167479000
H	5.340882000	2.024263000	-0.751908000
H	4.429512000	1.342895000	0.609757000
C	6.445908000	0.694918000	0.516718000
H	7.310345000	0.516480000	-0.134819000
C	6.575258000	0.527895000	1.833127000
H	5.740771000	0.709593000	2.519941000
H	7.524926000	0.224738000	2.280325000
Si	2.330641000	-2.909171000	0.744753000
C	0.661559000	-3.658193000	1.053949000
H	-0.077083000	-3.105038000	0.448176000
H	0.660041000	-4.714123000	0.743453000
H	0.371663000	-3.614236000	2.114117000
C	3.333885000	-2.431779000	2.231081000
H	2.758322000	-1.854322000	2.969343000
H	3.669272000	-3.357328000	2.728873000
H	4.236399000	-1.863570000	1.955830000
C	3.280153000	-3.746281000	-0.604000000
H	4.217994000	-3.227379000	-0.849969000
H	3.543439000	-4.765756000	-0.277142000
H	2.674114000	-3.840442000	-1.518113000
I	-2.554963000	-1.910230000	-1.152688000
C	-3.915657000	-0.939389000	0.138379000
C	-4.780788000	0.020958000	-0.384442000
C	-3.891561000	-1.269214000	1.494006000
C	-5.659377000	0.663667000	0.488128000
C	-4.766600000	-0.603292000	2.350791000
C	-5.649340000	0.355315000	1.848257000
H	-4.782144000	0.266996000	-1.448360000
H	-3.218975000	-2.039914000	1.876842000
H	-6.356631000	1.408082000	0.097340000
H	-4.777804000	-0.855502000	3.413714000
H	-6.346106000	0.857379000	2.523224000
O	-0.877279000	-1.262451000	-0.523101000

I	0.471079000	1.279467000	-2.144861000
C	-0.631738000	2.391790000	-0.736494000
C	-0.500127000	3.781147000	-0.736227000
C	-1.464455000	1.683561000	0.125110000
C	-1.251900000	4.488234000	0.200145000
C	-2.206086000	2.421857000	1.045595000
C	-2.099957000	3.812794000	1.081779000
H	0.151200000	4.305297000	-1.438403000
H	-1.494288000	0.588645000	0.082951000
H	-1.180279000	5.577825000	0.229560000
H	-2.877322000	1.896057000	1.728875000
H	-2.691133000	4.384590000	1.800746000
H	3.571359000	1.097264000	-2.614987000

67

TSBC

C	1.763622000	-0.304486000	0.310987000
O	1.082967000	-1.320731000	0.103123000
C	1.694767000	0.495921000	1.514156000
C	2.371539000	1.732146000	1.627243000
C	0.909589000	0.045591000	2.598066000
C	2.247394000	2.495334000	2.777385000
C	0.799439000	0.806756000	3.748737000
C	1.464185000	2.034720000	3.838696000
H	2.986647000	2.120211000	0.815742000
H	0.394878000	-0.911719000	2.538032000
H	2.769424000	3.450717000	2.855493000
H	0.200759000	0.445652000	4.587164000
H	1.378006000	2.633484000	4.748785000
C	2.692376000	0.066629000	-0.822288000
H	3.343246000	0.916978000	-0.604321000
C	3.437336000	-1.097938000	-1.433035000
H	2.723418000	-1.861917000	-1.776729000
C	4.392991000	-1.719510000	-0.403430000
H	3.815390000	-2.174510000	0.418129000
H	4.901283000	-2.556960000	-0.907956000
C	5.433312000	-0.759915000	0.173007000
H	5.975540000	-0.271861000	-0.658345000
H	4.945704000	0.044280000	0.752223000
C	6.408800000	-1.462503000	1.066424000
H	7.040453000	-2.221365000	0.587996000
C	6.541277000	-1.233063000	2.372251000
H	5.936104000	-0.478831000	2.887154000
H	7.269106000	-1.779136000	2.976994000
Si	-0.139906000	-2.700310000	0.558881000
C	-1.451334000	-2.548134000	1.907268000
H	-1.817440000	-1.524003000	2.060451000
H	-2.314915000	-3.170959000	1.619235000
H	-1.079869000	-2.946624000	2.862959000
C	1.297697000	-3.563831000	1.439998000
H	1.714733000	-3.001435000	2.290761000
H	0.906546000	-4.513177000	1.844975000
H	2.123823000	-3.820382000	0.759003000
C	-0.432789000	-3.776236000	-0.944878000
H	0.158378000	-3.395723000	-1.792657000
H	-0.089782000	-4.801197000	-0.738353000
H	-1.481875000	-3.842016000	-1.267995000
I	-2.958342000	-1.307941000	-1.091606000
C	-4.095932000	-0.176914000	0.260405000
C	-4.285507000	1.181075000	-0.011027000
C	-4.674252000	-0.814567000	1.360523000
C	-5.073646000	1.921992000	0.866824000
C	-5.458203000	-0.051493000	2.223282000

C	-5.654946000	1.308200000	1.977914000
H	-3.844823000	1.652659000	-0.891723000
H	-4.531712000	-1.880898000	1.543203000
H	-5.248830000	2.981998000	0.669474000
H	-5.927901000	-0.528954000	3.086000000
H	-6.281447000	1.894250000	2.654087000
O	-1.150737000	-1.106639000	-0.455221000
I	1.341876000	0.947362000	-2.377296000
C	0.464322000	2.422636000	-1.146395000
C	0.962848000	3.722099000	-1.214797000
C	-0.552916000	2.019532000	-0.283954000
C	0.403729000	4.666385000	-0.354028000
C	-1.082568000	2.987481000	0.569346000
C	-0.608482000	4.299371000	0.534093000
H	1.754173000	4.005810000	-1.911456000
H	-0.899232000	0.979368000	-0.259655000
H	0.764925000	5.696835000	-0.385254000
H	-1.876411000	2.705006000	1.264824000
H	-1.035227000	5.049752000	1.203352000
H	4.012619000	-0.745008000	-2.303973000

41

C

C	0.518043000	0.442203000	1.081723000
O	0.368120000	0.108889000	2.236602000
C	-0.231271000	1.552306000	0.459163000
C	0.007128000	2.011153000	-0.846549000
C	-1.254749000	2.137959000	1.221720000
C	-0.778160000	3.026447000	-1.382344000
C	-2.038664000	3.147939000	0.681701000
C	-1.803183000	3.590807000	-0.622118000
H	0.817675000	1.600303000	-1.451248000
H	-1.419458000	1.771697000	2.236752000
H	-0.584963000	3.387575000	-2.394614000
H	-2.835658000	3.598273000	1.277076000
H	-2.417067000	4.389089000	-1.045904000
C	1.483673000	-0.376032000	0.222238000
H	1.838763000	0.132357000	-0.679761000
C	2.560846000	-1.091726000	0.988837000
H	2.098917000	-1.631298000	1.828486000
C	3.604149000	-0.109331000	1.549040000
H	3.102549000	0.626682000	2.198786000
H	4.251917000	-0.699170000	2.215872000
C	4.474632000	0.600758000	0.513286000
H	5.299639000	1.090132000	1.060764000
H	4.950659000	-0.134790000	-0.156930000
C	3.775424000	1.652624000	-0.290892000
H	3.242026000	2.420392000	0.287405000
C	3.786825000	1.747465000	-1.624196000
H	4.325752000	1.023965000	-2.245345000
H	3.292973000	2.575012000	-2.140876000
I	0.132529000	-1.958020000	-0.704320000
C	-1.759579000	-1.177108000	-0.198439000
C	-2.434998000	-0.417779000	-1.151728000
C	-2.248545000	-1.423448000	1.082115000
C	-3.666136000	0.122558000	-0.789140000
C	-3.482828000	-0.867275000	1.415466000
C	-4.184813000	-0.100230000	0.486842000
H	-2.019071000	-0.237609000	-2.143795000
H	-1.689445000	-2.014030000	1.809113000
H	-4.218938000	0.724293000	-1.513340000
H	-3.893038000	-1.041110000	2.412493000
H	-5.151175000	0.328745000	0.760140000

H	3.072176000	-1.822309000	0.340715000

41

C'

C	-1.172400000	0.503021000	0.198758000
O	-1.180199000	-0.546764000	-0.463874000
C	-0.241370000	1.597675000	-0.085063000
C	0.064103000	2.573806000	0.881430000
C	0.439244000	1.593901000	-1.315382000
C	1.068537000	3.499846000	0.635191000
C	1.430968000	2.531257000	-1.562165000
C	1.752271000	3.475977000	-0.583612000
H	-0.455874000	2.590894000	1.842762000
H	0.172169000	0.843170000	-2.061704000
H	1.321459000	4.246034000	1.390848000
H	1.962472000	2.529147000	-2.515481000
H	2.537035000	4.210849000	-0.777145000
C	-2.174538000	0.612449000	1.239227000
H	-2.447766000	1.614654000	1.591243000
C	-2.958677000	-0.520614000	1.663491000
H	-2.370770000	-1.446605000	1.570668000
C	-4.237052000	-0.726523000	0.756449000
H	-3.881066000	-0.984315000	-0.252252000
H	-4.744792000	-1.613450000	1.167004000
C	-5.179165000	0.468581000	0.713580000
H	-5.416849000	0.794706000	1.741746000
H	-4.691865000	1.320471000	0.208117000
C	-6.438961000	0.105827000	-0.009606000
H	-7.137532000	-0.546463000	0.528838000
C	-6.737879000	0.498062000	-1.250226000
H	-6.071429000	1.153131000	-1.821171000
H	-7.667743000	0.192365000	-1.735839000
I	1.403214000	-1.699079000	-0.314812000
C	3.028310000	-0.411908000	-0.583329000
C	3.478571000	0.355675000	0.494525000
C	3.629379000	-0.335818000	-1.842828000
C	4.565200000	1.205288000	0.301712000
C	4.714960000	0.519598000	-2.013593000
C	5.183230000	1.287854000	-0.946386000
H	2.994822000	0.288174000	1.470332000
H	3.265409000	-0.941370000	-2.674820000
H	4.933722000	1.802844000	1.138459000
H	5.202725000	0.578443000	-2.989196000
H	6.038493000	1.952051000	-1.088124000
H	-3.322009000	-0.360713000	2.691596000

41

TSCD

C	0.924537000	0.548794000	0.627215000
O	0.925934000	0.331862000	1.824378000
C	0.020145000	1.537489000	0.000631000
C	-0.093419000	1.724551000	-1.386627000
C	-0.787135000	2.291815000	0.864932000
C	-0.998724000	2.648494000	-1.895366000
C	-1.689232000	3.215797000	0.354146000
C	-1.795928000	3.395345000	-1.025856000
H	0.507651000	1.143171000	-2.088450000
H	-0.692452000	2.126084000	1.939447000
H	-1.085315000	2.788888000	-2.974782000
H	-2.313778000	3.800562000	1.032521000
H	-2.503984000	4.123230000	-1.428807000
C	1.871464000	-0.252262000	-0.226228000

H	1.867231000	-0.117070000	-1.306435000
C	2.859697000	-1.160788000	0.406782000
H	2.295626000	-1.882451000	1.019419000
C	3.857353000	-0.498276000	1.380959000
H	3.298635000	-0.111663000	2.245354000
H	4.537588000	-1.278177000	1.753961000
C	4.637471000	0.622467000	0.700993000
H	5.332089000	1.067841000	1.432813000
H	5.241581000	0.227952000	-0.131459000
C	3.682884000	1.650439000	0.215240000
H	3.166273000	2.247071000	0.977601000
C	3.380191000	1.857416000	-1.083629000
H	3.907679000	1.319513000	-1.879178000
H	2.640414000	2.603761000	-1.384274000
I	-0.251602000	-2.072535000	-0.766659000
C	-1.832980000	-1.059401000	0.178338000
C	-2.750697000	-0.369289000	-0.613236000
C	-1.913188000	-1.092967000	1.569817000
C	-3.785808000	0.310235000	0.023399000
C	-2.957563000	-0.401683000	2.182779000
C	-3.889022000	0.294840000	1.414971000
H	-2.664162000	-0.355960000	-1.700512000
H	-1.182006000	-1.637139000	2.168826000
H	-4.517578000	0.853932000	-0.577793000
H	-3.040469000	-0.416971000	3.271639000
H	-4.706618000	0.827965000	1.904783000
H	3.398737000	-1.719194000	-0.372315000

41

D

C	2.198350000	0.975020000	-0.422335000
O	2.672788000	1.445137000	0.677552000
C	0.933989000	1.414495000	-0.896328000
C	0.386283000	0.850876000	-2.071401000
C	0.225676000	2.420644000	-0.195818000
C	-0.844435000	1.286323000	-2.530907000
C	-0.992543000	2.860377000	-0.675927000
C	-1.528342000	2.288225000	-1.835758000
H	0.923497000	0.070419000	-2.612186000
H	0.655480000	2.845521000	0.712378000
H	-1.278560000	0.848512000	-3.431126000
H	-1.542796000	3.638276000	-0.145488000
H	-2.496117000	2.634159000	-2.206338000
C	3.121327000	-0.051625000	-1.002785000
H	3.097098000	-0.084185000	-2.098751000
C	2.754461000	-1.424017000	-0.383319000
H	1.829650000	-1.811776000	-0.835124000
C	2.583598000	-1.327230000	1.134018000
H	1.586980000	-0.904752000	1.346496000
H	2.570020000	-2.332699000	1.577467000
C	3.647025000	-0.459854000	1.816240000
H	3.320770000	-0.172950000	2.826711000
H	4.594768000	-1.008869000	1.937362000
C	3.962550000	0.776966000	0.991313000
H	4.555056000	1.524230000	1.531816000
C	4.447131000	0.429834000	-0.403929000
H	5.220651000	-0.348479000	-0.404739000
H	4.837447000	1.316066000	-0.925870000
I	-1.294336000	-2.067514000	-0.278921000
C	-2.096416000	-0.343978000	0.623712000
C	-3.259949000	0.218726000	0.098804000
C	-1.481006000	0.199645000	1.751338000
C	-3.808631000	1.343702000	0.715972000

C	-2.039332000	1.326767000	2.355142000
C	-3.201014000	1.900571000	1.840281000
H	-3.746647000	-0.224437000	-0.772040000
H	-0.587672000	-0.263880000	2.174417000
H	-4.729312000	1.776999000	0.317659000
H	-1.568473000	1.746416000	3.247437000
H	-3.643042000	2.772964000	2.326484000
H	3.566343000	-2.117199000	-0.654028000

26

E

Si	2.928089000	0.603693000	0.551451000
C	3.976225000	0.381813000	2.076824000
H	4.358735000	-0.646642000	2.164400000
H	4.826140000	1.081684000	2.104970000
H	3.367972000	0.588015000	2.972719000
C	1.460454000	-0.527209000	0.461124000
H	1.755224000	-1.578298000	0.594548000
H	0.750604000	-0.264228000	1.262705000
H	0.939543000	-0.427978000	-0.502462000
C	2.547120000	2.394232000	0.215447000
H	2.006520000	2.519763000	-0.735238000
H	1.906306000	2.797793000	1.016395000
H	3.453971000	3.019917000	0.190354000
I	5.609740000	0.668801000	-1.336255000
C	6.865492000	-0.658642000	-0.334218000
C	7.084318000	-1.915394000	-0.906485000
C	7.447320000	-0.254011000	0.870911000
C	7.918375000	-2.799284000	-0.228279000
C	8.283855000	-1.155687000	1.521885000
C	8.514025000	-2.420156000	0.976265000
H	6.619023000	-2.203795000	-1.851003000
H	7.260949000	0.734328000	1.294686000
H	8.106493000	-3.789315000	-0.648905000
H	8.756701000	-0.866608000	2.462792000
H	9.170726000	-3.120604000	1.496986000
O	3.853937000	-0.000306000	-0.822638000

68

F

C	-2.854579000	0.764820000	0.419595000
O	-2.679355000	1.996851000	0.824552000
C	-2.728266000	-0.328684000	1.395008000
C	-3.436706000	-1.530751000	1.241200000
C	-1.853010000	-0.182198000	2.482941000
C	-3.276949000	-2.559316000	2.162448000
C	-1.691613000	-1.215695000	3.398718000
C	-2.405916000	-2.403985000	3.241646000
H	-4.139707000	-1.649354000	0.414426000
H	-1.277697000	0.738853000	2.586525000
H	-3.842981000	-3.485446000	2.044321000
H	-1.002579000	-1.096735000	4.237759000
H	-2.285807000	-3.212862000	3.965915000
C	-3.054222000	0.524962000	-0.941602000
H	-3.313973000	-0.503322000	-1.204174000
C	-3.489835000	1.623317000	-1.874959000
H	-2.821558000	2.494079000	-1.765739000
C	-4.927279000	2.084636000	-1.618372000
H	-4.981143000	2.561261000	-0.627088000
H	-5.177214000	2.874529000	-2.345910000
C	-5.972417000	0.973157000	-1.684837000
H	-5.955435000	0.518902000	-2.692796000

H	-5.716836000	0.170663000	-0.971937000
C	-7.348616000	1.473676000	-1.377297000
H	-7.755844000	2.223520000	-2.067757000
C	-8.081595000	1.093827000	-0.329785000
H	-7.716921000	0.344268000	0.381296000
H	-9.078069000	1.504079000	-0.148433000
Si	-3.440023000	2.985329000	2.058709000
C	-2.270661000	3.138349000	3.500827000
H	-1.249101000	3.378558000	3.166979000
H	-2.607545000	3.967378000	4.144206000
H	-2.238944000	2.230878000	4.120652000
C	-5.036884000	2.132367000	2.497196000
H	-4.857989000	1.134767000	2.926818000
H	-5.573048000	2.728444000	3.253143000
H	-5.703828000	2.026210000	1.627154000
C	-3.661620000	4.619492000	1.200868000
H	-4.327647000	4.542697000	0.329028000
H	-4.100156000	5.353064000	1.895851000
H	-2.692831000	5.017839000	0.861913000
H	-3.390425000	1.272087000	-2.914649000
I	-0.550217000	0.314345000	-1.435024000
C	-0.747282000	-1.699261000	-0.853610000
C	-1.510272000	-2.560762000	-1.637720000
C	-0.040798000	-2.125897000	0.266372000
C	-1.578630000	-3.902677000	-1.265666000
C	-0.124433000	-3.471015000	0.620585000
C	-0.889865000	-4.354226000	-0.140028000
H	-2.034987000	-2.207137000	-2.527927000
H	0.568549000	-1.433669000	0.848856000
H	-2.165803000	-4.598472000	-1.868928000
H	0.418070000	-3.827910000	1.498538000
H	-0.944585000	-5.407640000	0.142610000
O	1.393134000	0.062736000	-1.890078000
Si	2.039537000	-0.908545000	-3.141899000
C	2.319075000	-2.638339000	-2.485700000
H	1.375309000	-3.149814000	-2.242251000
H	2.846840000	-3.248751000	-3.236177000
H	2.940523000	-2.614860000	-1.577107000
C	3.643780000	-0.075783000	-3.592632000
H	4.309139000	-0.011276000	-2.718336000
H	4.167922000	-0.644840000	-4.377038000
H	3.470867000	0.944791000	-3.965969000
C	0.832739000	-0.937127000	-4.577692000
H	0.602700000	0.077667000	-4.939016000
H	1.269970000	-1.495441000	-5.421147000
H	-0.114583000	-1.437760000	-4.320402000

94

G

C	3.260567000	-0.651657000	0.042793000
O	3.114078000	-1.823096000	-0.422160000
C	3.532597000	-0.435420000	1.448560000
C	3.777924000	0.856106000	1.966014000
C	3.542048000	-1.536004000	2.333031000
C	3.996833000	1.037195000	3.322222000
C	3.761685000	-1.349963000	3.687405000
C	3.984738000	-0.061849000	4.184657000
H	3.807198000	1.727198000	1.312427000
H	3.384372000	-2.542122000	1.947052000
H	4.190915000	2.037815000	3.712493000
H	3.771512000	-2.207085000	4.363475000
H	4.165767000	0.083656000	5.252436000
C	3.097760000	0.446999000	-0.912795000

H	3.453642000	1.408806000	-0.534246000	C	-2.883619000	2.342791000	2.728306000
C	3.532784000	0.167919000	-2.342903000	C	-3.407689000	0.200764000	3.715191000
H	2.944426000	-0.665004000	-2.761779000	C	-3.238530000	1.578820000	3.839307000
C	5.022204000	-0.166972000	-2.455252000	H	-2.403209000	2.329917000	0.617374000
H	5.226143000	-1.134924000	-1.967486000	H	-3.393035000	-1.498907000	2.353745000
H	5.250849000	-0.316772000	-3.522876000	H	-2.756606000	3.423695000	2.820817000
C	5.955362000	0.895890000	-1.876977000	H	-3.688397000	-0.400936000	4.582774000
H	5.717012000	1.872714000	-2.338243000	H	-3.392755000	2.061877000	4.806530000
H	5.787677000	1.008447000	-0.791312000	O	-3.733585000	-2.187902000	0.230332000
C	7.398682000	0.571072000	-2.108430000	Si	-5.432596000	-2.376834000	-0.048564000
H	7.718542000	0.519354000	-3.156825000	C	-5.868300000	-3.891644000	0.936780000
C	8.289326000	0.337246000	-1.145145000	H	-5.690585000	-3.737907000	2.011878000
H	8.014946000	0.385285000	-0.085630000	H	-6.934161000	-4.136896000	0.803885000
H	9.331186000	0.101786000	-1.375078000	H	-5.278297000	-4.760562000	0.608762000
Si	2.526611000	-3.480022000	-0.623956000	C	-5.675905000	-2.605183000	-1.887047000
C	0.935828000	-3.488080000	0.346322000	H	-5.104838000	-3.467595000	-2.264331000
H	0.219289000	-2.753023000	-0.052883000	H	-6.738808000	-2.786834000	-2.113653000
H	0.466704000	-4.481721000	0.256545000	H	-5.376170000	-1.711665000	-2.458911000
H	1.087619000	-3.294831000	1.418647000	C	-6.298821000	-0.830219000	0.548113000
C	3.868324000	-4.578230000	0.027577000	H	-5.968093000	0.069063000	0.003087000
H	4.008307000	-4.513554000	1.116329000	H	-7.384263000	-0.923270000	0.381895000
H	3.614535000	-5.624817000	-0.208292000	H	-6.141033000	-0.656930000	1.623489000
H	4.830191000	-4.357205000	-0.460518000	I	0.829224000	0.750753000	-1.069420000
C	2.272714000	-3.625851000	-2.453563000	C	0.652635000	1.320019000	0.946529000
H	3.211823000	-3.457061000	-3.002730000	C	0.831102000	2.661404000	1.274724000
H	1.925181000	-4.641562000	-2.702737000	C	0.375559000	0.336454000	1.893217000
H	1.515526000	-2.917388000	-2.822362000	C	0.737083000	3.021233000	2.618889000
H	3.308667000	1.060108000	-2.950627000	C	0.286114000	0.721694000	3.229267000
I	-2.707779000	-0.643002000	-0.482201000	C	0.467656000	2.056335000	3.589422000
C	-2.891875000	0.370327000	1.395313000	H	1.030854000	3.416909000	0.512600000
C	-2.698792000	1.741290000	1.483124000	H	0.225848000	-0.706067000	1.608321000
C	-3.237769000	-0.425254000	2.478763000	H	0.870721000	4.067497000	2.903110000

H	0.060749000	-0.029363000	3.988888000
H	0.389613000	2.350211000	4.638318000
O	-1.440203000	1.104789000	-1.192482000
Si	-2.025613000	2.352285000	-2.232981000
C	-1.299977000	3.964302000	-1.615963000
H	-0.201925000	3.981838000	-1.705563000
H	-1.681417000	4.800685000	-2.223691000
H	-1.567253000	4.174252000	-0.568628000
C	-3.890167000	2.348246000	-2.131028000
H	-4.262643000	2.462067000	-1.101221000
H	-4.278683000	3.199145000	-2.714355000
H	-4.328667000	1.434622000	-2.560705000
C	-1.431605000	1.973725000	-3.963731000
H	-1.789841000	0.990802000	-4.307944000
H	-1.813220000	2.727269000	-4.671403000
H	-0.332680000	1.989390000	-4.045155000

94

TSGH

C	2.716380000	-0.899470000	-0.158518000
O	1.917602000	-1.801648000	-0.479362000
C	3.597746000	-1.021646000	0.983736000
C	4.261083000	0.088865000	1.550837000
C	3.808407000	-2.308216000	1.527765000
C	5.099146000	-0.087454000	2.641563000
C	4.665506000	-2.478506000	2.602008000
C	5.308623000	-1.368383000	3.160697000
H	4.102136000	1.097273000	1.165089000
H	3.320115000	-3.167812000	1.065399000
H	5.601662000	0.772353000	3.088566000
H	4.847775000	-3.476387000	3.005638000
H	5.986014000	-1.504533000	4.007298000
C	2.810787000	0.294879000	-1.079349000
H	3.477826000	1.078385000	-0.706584000
C	3.150926000	-0.123660000	-2.507357000
H	2.333266000	-0.742724000	-2.911397000
C	4.457313000	-0.920027000	-2.569332000
H	4.344529000	-1.867345000	-2.014294000
H	4.616090000	-1.213270000	-3.619437000
C	5.686709000	-0.173249000	-2.052437000
H	5.833453000	0.740820000	-2.656209000
H	5.532336000	0.156572000	-1.011104000
C	6.920801000	-1.020184000	-2.096982000
H	7.271271000	-1.319082000	-3.092651000
C	7.586449000	-1.435479000	-1.019498000
H	7.271011000	-1.152825000	-0.008645000
H	8.478850000	-2.060296000	-1.103147000
Si	0.152846000	-2.630031000	-0.069493000
C	0.098919000	-1.641209000	1.516913000
H	-0.011945000	-0.556546000	1.406876000
H	-0.731957000	-2.006766000	2.132664000
H	1.040823000	-1.845068000	2.049028000
C	0.552528000	-4.394548000	0.339949000
H	1.184842000	-4.468166000	1.236564000
H	-0.377806000	-4.942172000	0.551550000
H	1.051927000	-4.904009000	-0.497215000
C	-0.264095000	-2.484197000	-1.887308000
H	0.597208000	-2.904992000	-2.429469000
H	-1.147838000	-3.090472000	-2.124141000
H	-0.432317000	-1.467376000	-2.266480000
H	3.224839000	0.781541000	-3.130539000
I	-2.372619000	-0.260360000	-0.390070000
C	-2.585890000	0.318874000	1.660233000

C	-2.254231000	1.620390000	2.006632000
C	-3.047547000	-0.623025000	2.568759000
C	-2.401433000	1.991118000	3.345284000
C	-3.188641000	-0.224440000	3.898364000
C	-2.868507000	1.076254000	4.286708000
H	-1.899940000	2.323258000	1.252904000
H	-3.282699000	-1.639726000	2.257640000
H	-2.154814000	3.012736000	3.643848000
H	-3.560076000	-0.942902000	4.632483000
H	-2.990771000	1.378648000	5.328797000
O	-2.321894000	-2.320308000	0.150026000
Si	-3.641322000	-3.352159000	-0.267835000
C	-3.021977000	-5.107371000	-0.137107000
H	-2.660788000	-5.342091000	0.876041000
H	-3.856200000	-5.795225000	-0.350917000
H	-2.225424000	-5.331797000	-0.862535000
C	-4.178389000	-2.956386000	-2.019920000
H	-3.368463000	-3.070758000	-2.757235000
H	-4.982236000	-3.649288000	-2.317463000
H	-4.591214000	-1.939068000	-2.114822000
C	-5.059077000	-3.044836000	0.909649000
H	-5.387336000	-1.993283000	0.911449000
H	-5.924949000	-3.650982000	0.597028000
H	-4.817697000	-3.336873000	1.943583000
I	0.816726000	1.270508000	-1.095159000
C	1.219161000	2.700718000	0.399261000
C	1.526718000	3.997597000	-0.006740000
C	1.140719000	2.311975000	1.735191000
C	1.776083000	4.944284000	0.986954000
C	1.400810000	3.278261000	2.705980000
C	1.714507000	4.586076000	2.333721000
H	1.568239000	4.277257000	-1.060885000
H	0.878414000	1.294355000	2.027387000
H	2.018459000	5.969589000	0.698728000
H	1.347192000	3.002008000	3.761366000
H	1.910341000	5.336710000	3.102542000
O	-1.869017000	1.721474000	-0.871821000
Si	-2.899440000	2.866161000	-1.638505000
C	-1.785423000	4.337898000	-1.934949000
H	-0.951094000	4.081755000	-2.607851000
H	-2.347099000	5.156978000	-2.411895000
H	-1.373855000	4.725113000	-0.989722000
C	-4.317152000	3.254122000	-0.488740000
H	-3.978615000	3.730538000	0.443634000
H	-5.023668000	3.944000000	-0.977744000
H	-4.883004000	2.347208000	-0.221498000
C	-3.505151000	2.110270000	-3.236693000
H	-4.128384000	1.218813000	-3.058063000
H	-4.129060000	2.828443000	-3.792565000
H	-2.670573000	1.822090000	-3.895154000

94

H

C	3.718326000	0.710490000	-0.508422000
O	2.648111000	0.897939000	-1.048841000
C	4.125698000	1.408442000	0.727588000
C	5.252959000	1.051549000	1.486961000
C	3.339379000	2.499941000	1.136337000
C	5.590031000	1.778228000	2.623394000
C	3.684077000	3.228896000	2.265686000
C	4.811193000	2.869126000	3.009743000
H	5.876493000	0.198951000	1.213806000
H	2.466066000	2.766340000	0.537520000

H	6.462219000	1.490684000	3.213618000	I	-4.561719000	-0.258814000	-0.254540000
H	3.076101000	4.082541000	2.572699000	C	-4.213283000	0.452014000	1.721486000
H	5.080911000	3.440824000	3.900858000	C	-4.829373000	1.655130000	2.041019000
C	4.694258000	-0.268929000	-1.160831000	C	-3.440703000	-0.280734000	2.611931000
H	5.465204000	-0.656006000	-0.485925000	C	-4.653056000	2.144631000	3.335780000
C	4.053680000	-1.338399000	-2.011144000	C	-3.280415000	0.234440000	3.899003000
H	3.422851000	-0.872038000	-2.783396000	C	-3.882570000	1.439231000	4.259424000
C	3.174037000	-2.264373000	-1.158940000	H	-5.444772000	2.187785000	1.312978000
H	2.289125000	-1.708032000	-0.817437000	H	-2.973532000	-1.219950000	2.321116000
H	2.800667000	-3.054485000	-1.830712000	H	-5.126179000	3.087945000	3.617470000
C	3.872093000	-2.901043000	0.041603000	H	-2.687627000	-0.324132000	4.626792000
H	4.802972000	-3.394368000	-0.295525000	H	-3.755498000	1.829386000	5.271331000
H	4.167447000	-2.129994000	0.774617000	O	-2.347529000	-1.351282000	-0.070305000
C	3.002487000	-3.907094000	0.729543000	Si	-2.510422000	-3.074282000	-0.266761000
H	2.700777000	-4.773145000	0.126324000	C	-0.856207000	-3.905308000	-0.029916000
C	2.583305000	-3.818928000	1.992490000	H	-0.474463000	-3.785318000	0.994303000
H	2.868771000	-2.978863000	2.634580000	H	-0.999193000	-4.984804000	-0.202700000
H	1.958591000	-4.595573000	2.440934000	H	-0.080693000	-3.563030000	-0.729443000
Si	-0.919012000	-0.382302000	-0.283760000	C	-3.176210000	-3.343227000	-1.989854000
C	-1.417286000	1.414223000	-0.195716000	H	-2.510842000	-2.908588000	-2.751571000
H	-2.100952000	1.717494000	-1.002626000	H	-3.258821000	-4.422215000	-2.196985000
H	-1.863138000	1.689378000	0.771750000	H	-4.181511000	-2.915002000	-2.128561000
H	-0.494672000	2.006194000	-0.316611000	C	-3.699640000	-3.670202000	1.043924000
C	0.259093000	-0.759123000	1.115307000	H	-4.686204000	-3.185353000	0.999214000
H	-0.236734000	-0.606256000	2.086715000	H	-3.865056000	-4.750395000	0.899066000
H	0.669644000	-1.778241000	1.091863000	H	-3.294454000	-3.540998000	2.059438000
H	1.102483000	-0.052075000	1.060486000	I	5.954922000	0.985504000	-2.526665000
C	-0.233004000	-0.755611000	-1.980790000	C	6.940863000	2.189035000	-1.105984000
H	0.666701000	-0.140637000	-2.140293000	C	8.140001000	1.725491000	-0.568327000
H	0.045351000	-1.810250000	-2.118856000	C	6.351102000	3.400737000	-0.751135000
H	-0.965780000	-0.492304000	-2.759624000	C	8.765450000	2.522334000	0.389378000
H	4.836756000	-1.931386000	-2.510448000	C	6.999321000	4.173604000	0.209732000

C	8.196421000	3.735743000	0.776859000
H	8.587220000	0.780340000	-0.881901000
H	5.415637000	3.740046000	-1.198432000
H	9.710240000	2.190865000	0.825797000
H	6.563136000	5.128804000	0.509185000
H	8.699249000	4.353879000	1.523751000
O	-6.305806000	0.633784000	-0.356764000
Si	-7.790815000	-0.176103000	0.070560000
C	-9.060376000	1.151403000	-0.193683000
H	-9.047078000	1.510125000	-1.233653000
H	-10.067117000	0.756480000	0.017593000
H	-8.886896000	2.010125000	0.471863000
C	-7.642738000	-0.737702000	1.846024000
H	-7.471057000	0.108598000	2.528416000
H	-8.575443000	-1.235062000	2.157890000
H	-6.827247000	-1.464932000	1.990237000
C	-7.974855000	-1.629384000	-1.087307000
H	-7.180115000	-2.380380000	-0.946828000
H	-8.932711000	-2.142435000	-0.903792000
H	-7.969347000	-1.306302000	-2.139866000

27

OTMS2

Si	-0.811853000	-1.526143000	0.089433000
C	-0.825254000	0.320601000	0.388916000
H	-1.183483000	0.858280000	-0.502755000
H	-1.494412000	0.573325000	1.226092000
H	0.182053000	0.694338000	0.632003000
C	-0.226853000	-2.439808000	1.620033000
H	-0.891113000	-2.233714000	2.474021000
H	-0.219721000	-3.528417000	1.452734000
H	0.793503000	-2.134579000	1.902304000
C	0.287624000	-1.951610000	-1.373070000
H	1.327740000	-1.634577000	-1.195220000
H	0.297673000	-3.037353000	-1.561096000
H	-0.065833000	-1.452723000	-2.289279000
O	-2.379578000	-1.982063000	-0.247672000
Si	-3.294420000	-3.210472000	-0.905946000
C	-2.633944000	-4.865300000	-0.310851000
H	-2.652107000	-4.923761000	0.788922000
H	-3.236870000	-5.699579000	-0.703834000
H	-1.594227000	-5.020987000	-0.641074000
C	-3.190578000	-3.112738000	-2.776621000
H	-2.154958000	-3.255132000	-3.123505000
H	-3.815692000	-3.885104000	-3.252689000
H	-3.534486000	-2.129626000	-3.134716000
C	-5.056284000	-2.957777000	-0.329451000
H	-5.431500000	-1.972938000	-0.648540000
H	-5.726972000	-3.728590000	-0.741101000
H	-5.118064000	-3.001999000	0.769061000

48

C-SbF6

C	2.77116	0.75698	-0.53961
O	3.23534	1.76432	-1.04980
C	3.59959	-0.40787	-0.17777
C	4.99388	-0.23828	-0.16427
C	3.04026	-1.66180	0.11292
C	5.81818	-1.30731	0.15631
C	3.87624	-2.73031	0.42388
C	5.25861	-2.55404	0.45098
H	5.40391	0.74380	-0.40769

H	1.96190	-1.83467	0.08998
H	6.90214	-1.17483	0.17506
H	3.43486	-3.70446	0.64343
H	5.90925	-3.39588	0.70021
C	1.28810	0.68141	-0.25778
H	0.77180	-0.08640	-0.86110
C	0.81921	0.63202	1.16813
H	-0.27402	0.76882	1.17211
C	1.50119	1.55953	2.15420
H	2.56361	1.28325	2.26465
H	1.49085	2.59691	1.77173
C	0.80788	1.51169	3.52090
H	0.75056	0.46279	3.85582
H	1.42784	2.04786	4.25770
C	-0.56685	2.10884	3.48331
H	-0.61301	3.20644	3.47606
C	-1.70442	1.41610	3.40619
H	-1.71512	0.32210	3.37660
H	-2.67292	1.91832	3.34032
I	0.62369	2.56224	-1.25589
C	-1.41064	2.40237	-0.71351
C	-2.16229	1.33380	-1.19049
C	-1.92733	3.36984	0.14779
C	-3.48404	1.22150	-0.76202
C	-3.26155	3.25623	0.53117
C	-4.03205	2.18280	0.08376
H	-1.73065	0.56226	-1.82900
H	-1.30917	4.18696	0.52392
H	-4.05743	0.34831	-1.07840
H	-3.69112	4.00286	1.20287
H	-5.06922	2.08648	0.41226
H	0.92909	-0.42479	1.47248
F	-1.83743	-0.79576	0.90058
Sb	-1.55101	-2.36190	-0.19152
F	-1.18361	-3.86470	-1.29824
F	-2.48082	-3.43029	1.07763
F	-0.55716	-1.24057	-1.45323
F	-3.14165	-1.92213	-1.16665
F	0.13392	-2.58807	0.73594

48

TSCD-SbF6

C	1.95908	1.91839	0.03324
O	2.54970	2.85722	-0.46417
C	2.59369	1.00862	1.02254
C	1.88998	0.01557	1.72031
C	3.96801	1.17325	1.24863
C	2.55857	-0.79103	2.63738
C	4.63171	0.35653	2.15519
C	3.92490	-0.62511	2.85433
H	0.82142	-0.14548	1.56779
H	4.49268	1.95396	0.69425
H	2.00272	-1.56086	3.17609
H	5.70322	0.48580	2.32405
H	4.44500	-1.26437	3.57202
C	0.53250	1.65264	-0.38783
H	-0.04390	0.84657	0.07552
C	-0.13854	2.55085	-1.35305
H	0.63307	3.03846	-1.96782
C	-1.02700	3.64464	-0.71363
H	-1.38670	4.29190	-1.52721
H	-1.91181	3.16399	-0.27339
C	-0.25734	4.45045	0.31455

H	0.61340	4.94253	-0.14837
H	-0.89444	5.26573	0.70615
C	0.21090	3.60909	1.45280
H	1.14994	3.90286	1.93561
C	-0.43200	2.51253	1.91080
H	-1.40241	2.18594	1.52761
H	-0.01338	1.93258	2.73648
I	1.49268	-0.20583	-2.20180
C	1.58054	-1.88030	-0.93700
C	2.82287	-2.32982	-0.49224
C	0.38104	-2.46452	-0.54440
C	2.85005	-3.41213	0.38413
C	0.43272	-3.53225	0.35019
C	1.66060	-4.00647	0.80925
H	3.74656	-1.83866	-0.80210
H	-0.57935	-2.09729	-0.90276
H	3.81174	-3.78071	0.74805
H	-0.51016	-3.97207	0.68129
H	1.69307	-4.84605	1.50749
H	-0.80357	1.92892	-1.96960
F	-4.48478	-0.54943	-0.75553
Sb	-2.91059	-0.48446	0.30711
F	-1.23372	-0.35396	1.29696
F	-3.06831	1.43296	0.50501
F	-2.59421	-2.36508	0.09509
F	-3.85274	-0.75999	1.93504
F	-1.82077	-0.15795	-1.26612

48

D-SbF6

C	0.07577	1.69301	0.80259
O	-0.86655	2.02667	-0.00211
C	-0.19085	0.73221	1.82898
C	0.88111	0.13681	2.51984
C	-1.52022	0.34767	2.09905
C	0.61665	-0.83942	3.46891
C	-1.77019	-0.60643	3.07010
C	-0.70180	-1.20321	3.74631
H	1.91096	0.38485	2.25771
H	-2.33702	0.80574	1.53902
H	1.44459	-1.33542	3.97786
H	-2.79554	-0.91138	3.28368
H	-0.90247	-1.97564	4.49280
C	1.27493	2.56117	0.60851
H	2.21900	2.04814	0.82374
C	1.06382	3.81124	1.49760
H	1.23235	3.56060	2.55589
C	-0.33267	4.41860	1.31749
H	-1.05238	3.83598	1.91677
H	-0.36155	5.43585	1.73458
C	-0.81617	4.43110	-0.13912
H	-1.90690	4.57179	-0.17676
H	-0.36771	5.26527	-0.70226
C	-0.41857	3.14904	-0.85588
H	-0.92770	3.00444	-1.81593
C	1.08483	2.94912	-0.85938
H	1.62736	3.86497	-1.12688
H	1.40180	2.13336	-1.52094
I	-3.80391	0.06550	-1.42446
C	-2.82919	-1.47852	-0.36635
C	-3.58140	-2.31503	0.45844
C	-1.44983	-1.62929	-0.49203
C	-2.92706	-3.31847	1.17302

C	-0.80868	-2.63312	0.23562
C	-1.54515	-3.47569	1.06576
H	-4.66263	-2.19009	0.54111
H	-0.85676	-0.97413	-1.12964
H	-3.50940	-3.98272	1.81684
H	0.27537	-2.73445	0.15677
H	-1.03747	-4.26062	1.63102
H	1.84779	4.52973	1.21084
F	3.43992	0.42591	0.69560
Sb	2.89081	-0.79595	-0.71306
F	2.20533	-1.97086	-2.04644
F	4.64289	-1.52403	-0.81222
F	1.10589	0.00204	-0.59116
F	2.35731	-2.01711	0.67226
F	3.27473	0.57820	-1.99176

54

SI-A

C	-0.09574	1.50615	-0.39804
O	-0.50537	1.47500	-1.53915
C	0.95026	2.47468	0.02188
C	1.21912	3.54937	-0.83993
C	1.69100	2.33427	1.20368
C	2.20242	4.47604	-0.51724
C	2.68290	3.25964	1.51807
C	2.93689	4.33115	0.66269
H	0.64095	3.64115	-1.76153
H	1.51071	1.49913	1.88357
H	2.40186	5.31559	-1.18736
H	3.26140	3.14012	2.43706
H	3.71269	5.05846	0.91497
C	-0.63242	0.57266	0.67958
H	0.17395	-0.05714	1.08544
C	-1.44969	1.24915	1.76035
H	-0.76247	1.98588	2.21158
C	-2.00193	0.33670	2.85191
H	-2.38070	0.98638	3.65778
H	-2.88285	-0.20715	2.47308
C	-1.01421	-0.67886	3.42794
H	-1.53389	-1.22161	4.23727
H	-0.74643	-1.43834	2.67350
C	0.24054	-0.07609	3.97693
H	0.11437	0.70534	4.73902
C	1.47402	-0.43016	3.60396
H	1.62736	-1.20435	2.84288
H	2.36332	0.02927	4.04602
I	1.79605	-2.70573	-0.48141
C	2.75622	-0.89411	-0.94686
C	3.76198	-0.43316	-0.09534
C	2.37097	-0.20705	-2.09962
C	4.40164	0.76131	-0.42067
C	3.02450	0.98558	-2.40219
C	4.03645	1.46365	-1.56949
H	4.04623	-0.98926	0.80056
H	1.57871	-0.58304	-2.75017
H	5.19081	1.14270	0.23105
H	2.73509	1.54364	-3.29534
H	4.54333	2.39936	-1.81541
O	0.31128	-2.18727	0.59876
I	-1.76222	-1.03196	-0.29969
C	-3.52637	0.09332	-0.68265
C	-3.57050	1.02184	-1.72219
C	-4.64032	-0.17682	0.11331
C	-4.76384	1.70557	-1.95332

C	-5.82647	0.51415	-0.13582
C	-5.88677	1.45380	-1.16419
H	-2.68627	1.22097	-2.32832
H	-4.59509	-0.91336	0.91940
H	-4.81273	2.44014	-2.76094
H	-6.70560	0.31274	0.48094
H	-6.81764	1.99360	-1.35426
H	-2.26136	1.83856	1.30401

54

SI-A-prime

C	2.82031	1.42637	-0.67403
O	1.81607	0.73316	-1.27153
C	3.78679	0.62455	0.11023
C	3.97502	-0.73190	-0.19791
C	4.50696	1.17627	1.18192
C	4.86272	-1.51284	0.53908
C	5.39792	0.39600	1.91337
C	5.57829	-0.95213	1.59671
H	3.42416	-1.17101	-1.03160
H	4.35060	2.22210	1.45749
H	4.99724	-2.56678	0.28212
H	5.94638	0.84039	2.74788
H	6.27222	-1.56460	2.17799
C	2.90098	2.76285	-0.85464
H	3.78409	3.27844	-0.46421
C	1.88538	3.58267	-1.57468
H	2.31779	3.94221	-2.52645
C	1.41603	4.79878	-0.77451
H	2.27756	5.45529	-0.56659
H	0.72651	5.38823	-1.40255
C	0.72616	4.46259	0.54571
H	1.42044	3.94599	1.23025
H	0.45913	5.41373	1.04247
C	-0.52538	3.65583	0.39615
H	-1.16683	3.91369	-0.45842
C	-0.93435	2.69354	1.23232
H	-0.34685	2.41765	2.11629
H	-1.89330	2.18519	1.09230
I	-2.77917	-1.68354	0.66981
C	-3.67960	0.08711	0.02295
C	-4.41727	0.83173	0.94557
C	-3.43743	0.51251	-1.28557
C	-4.92363	2.06060	0.53032
C	-3.95403	1.74717	-1.67215
C	-4.68904	2.51587	-0.76842
H	-4.58738	0.47494	1.96332
H	-2.86249	-0.09308	-1.98939
H	-5.50198	2.66634	1.23134
H	-3.77839	2.10671	-2.68847
H	-5.08781	3.48344	-1.08173
O	-1.12229	-0.94683	1.37243
I	0.33513	-0.01161	0.00690
C	0.76889	-1.97006	-0.64726
C	0.98075	-2.96695	0.30090
C	0.84731	-2.19304	-2.01867
C	1.28443	-4.24800	-0.15890
C	1.16122	-3.48106	-2.45276
C	1.37778	-4.50250	-1.52774
H	0.91290	-2.75855	1.37017
H	0.68189	-1.38861	-2.73815
H	1.45882	-5.04824	0.56417
H	1.23012	-3.68301	-3.52426
H	1.62182	-5.50844	-1.87716

H 1.03004 2.94499 -1.84782

41

SI-B

C	3.81875	-0.40466	0.23291
O	3.55536	-1.54884	0.56732
C	5.20738	0.08275	0.09651
C	5.49770	1.40470	-0.27349
C	6.25514	-0.81665	0.34732
C	6.82135	1.81815	-0.39132
C	7.57479	-0.39934	0.22778
C	7.85813	0.91802	-0.14185
H	4.69330	2.11748	-0.46906
H	6.01167	-1.84159	0.63498
H	7.04625	2.84768	-0.67884
H	8.38928	-1.10085	0.42267
H	8.89658	1.24572	-0.23570
C	2.68353	0.54148	-0.05227
H	2.65657	1.40064	0.63452
H	2.67424	0.90320	-1.09091
I	-2.73800	-1.70498	-0.56854
C	-3.82805	-0.06943	0.21671
C	-4.65175	0.68155	-0.61298
C	-3.65456	0.18973	1.57014
C	-5.35092	1.74370	-0.03653
C	-4.35853	1.26084	2.11934
C	-5.20470	2.03122	1.32043
H	-4.75257	0.46498	-1.67930
H	-2.97451	-0.41857	2.17209
H	-6.00637	2.35384	-0.66252
H	-4.24150	1.49072	3.18116
H	-5.75378	2.86714	1.75996
O	-1.30888	-1.74210	0.68337
I	0.89867	-0.64211	0.26970
C	-0.33948	0.97717	-0.26647
C	-0.80887	1.82367	0.73430
C	-0.67458	1.14309	-1.60932
C	-1.64069	2.88098	0.36630
C	-1.51379	2.20291	-1.95307
C	-1.99496	3.06666	-0.96931
H	-0.54141	1.66941	1.78156
H	-0.29480	0.46743	-2.37891
H	-2.01959	3.55668	1.13622
H	-1.79109	2.34813	-2.99976
H	-2.65457	3.89215	-1.24613

41

SI-B-prime

C	3.30827	0.64994	-1.25585
O	2.59372	-0.37626	-1.77534
C	3.94908	0.41446	0.05953
C	4.10954	1.45550	0.98545
C	4.38357	-0.87468	0.40437
C	4.70309	1.21432	2.22184
C	4.97722	-1.11343	1.64132
C	5.13857	-0.06985	2.55333
H	3.74577	2.45577	0.73784
H	4.26696	-1.68986	-0.31453
H	4.81714	2.03263	2.93732
H	5.31924	-2.12028	1.89388
H	5.60089	-0.25788	3.52569
C	3.43070	1.78949	-1.96105
H	4.10620	2.58132	-1.63279
H	2.90238	1.90742	-2.90929

I	-2.52283	-1.81837	0.02814
C	-3.27677	0.08770	0.44589
C	-3.57122	0.40715	1.77269
C	-3.48188	0.96615	-0.61880
C	-4.09114	1.67229	2.03293
C	-4.01823	2.21886	-0.32969
C	-4.31802	2.56879	0.98697
H	-3.40282	-0.30349	2.58441
H	-3.23195	0.69175	-1.64523
H	-4.32813	1.95262	3.06166
H	-4.19150	2.92701	-1.14285
H	-4.73385	3.55574	1.20252
O	-0.68168	-1.71637	0.63704
I	0.99635	-1.09573	-0.63195
C	0.18434	0.84320	-0.55636
C	-0.06741	1.40747	0.69095
C	-0.01648	1.51482	-1.75798
C	-0.56057	2.71039	0.72351
C	-0.50337	2.82056	-1.69634
C	-0.77465	3.41260	-0.46343
H	0.10700	0.85175	1.61411
H	0.20437	1.04440	-2.71826
H	-0.77184	3.17824	1.68769
H	-0.67245	3.37230	-2.62391
H	-1.15822	4.43471	-0.42579

43

SI-C

C	0.75738	-1.11725	-1.37596
O	1.19527	-1.23129	-2.51392
C	-0.50393	-1.83025	-0.98773
C	-1.39412	-2.18765	-2.00964
C	-0.82395	-2.15334	0.33743
C	-2.59175	-2.83048	-1.71278
C	-2.01419	-2.81455	0.63356
C	-2.90375	-3.14588	-0.38843
H	-1.12665	-1.94122	-3.03972
H	-0.13887	-1.90024	1.14842
H	-3.28606	-3.09012	-2.51604
H	-2.24973	-3.06800	1.66997
H	-3.84239	-3.65435	-0.15316
C	1.38024	-0.20758	-0.39220
H	1.15286	-0.45536	0.64956
C	2.83943	0.11489	-0.62671
H	2.96835	0.51132	-1.64835
C	3.76974	-1.08691	-0.45064
H	3.59106	-1.80843	-1.26377
H	4.81112	-0.73996	-0.56607
C	3.62066	-1.81040	0.89053
H	2.64423	-2.31975	0.94514
H	4.38638	-2.60550	0.93627
C	3.78275	-0.91039	2.07430
H	4.72372	-0.34428	2.12098
C	2.88221	-0.73973	3.04630
H	1.93108	-1.28518	3.04539
H	3.06527	-0.06312	3.88689
I	0.25541	1.79751	-0.71935
C	-1.18779	1.13922	0.67678
C	-2.49684	0.94730	0.24536
C	-0.80253	0.92575	1.99663
C	-3.44283	0.50529	1.16941
C	-1.76431	0.48839	2.90882
C	-3.07899	0.27447	2.49655
H	-2.78292	1.12480	-0.79321

H	0.22828	1.08922	2.32054
H	-4.47131	0.33422	0.84227
H	-1.47754	0.31887	3.94967
H	-3.82597	-0.07337	3.21403
H	3.14262	0.91608	0.06821
O	-0.90004	3.59868	-0.78040
H	-1.50684	3.48182	-1.52602

43

SI-C-prime

C	1.78993	0.67802	0.65532
O	0.88338	-0.30617	0.80470
C	3.19859	0.20819	0.53605
C	3.46284	-1.16664	0.63656
C	4.28563	1.07387	0.32127
C	4.76156	-1.66166	0.52507
C	5.58157	0.58002	0.21135
C	5.82897	-0.79154	0.31200
H	2.62429	-1.84313	0.80961
H	4.12226	2.14975	0.23174
H	4.93928	-2.73740	0.60774
H	6.40918	1.27404	0.04183
H	6.84832	-1.17599	0.22366
C	1.43507	1.98510	0.62731
H	2.22031	2.73861	0.52313
C	0.02898	2.47705	0.70162
H	-0.64520	1.63736	0.92958
C	-0.43932	3.18014	-0.57440
H	0.17465	4.08002	-0.74829
H	-0.26144	2.51778	-1.43978
C	-1.91710	3.57463	-0.53698
H	-2.11051	4.22739	0.33146
H	-2.14244	4.16929	-1.44020
C	-2.83284	2.39169	-0.49423
H	-2.75962	1.70106	-1.34664
C	-3.69028	2.10827	0.48888
H	-3.78948	2.76115	1.36387
H	-4.32204	1.21568	0.45889
I	-0.21434	-0.81913	-0.98984
C	-1.88245	-1.14200	0.31146
C	-3.08792	-1.49667	-0.27855
C	-1.72317	-0.98462	1.68229
C	-4.18820	-1.70453	0.55484
C	-2.84055	-1.19349	2.49317
C	-4.06721	-1.55298	1.93589
H	-3.16229	-1.58897	-1.36377
H	-0.75431	-0.70291	2.09643
H	-5.14780	-1.98090	0.11079
H	-2.74055	-1.07291	3.57477
H	-4.93392	-1.71466	2.58120
H	-0.07259	3.18763	1.54346
O	-1.38679	-1.26633	-2.62024
H	-1.33358	-2.22605	-2.75679

30

SI-D

C	1.36932	1.19466	0.91648
O	1.02484	2.36434	0.80687
C	2.61868	0.72295	0.23593
C	3.29380	1.63516	-0.58770
C	3.13175	-0.57432	0.37615
C	4.45237	1.26136	-1.25904
C	4.29310	-0.94948	-0.29653
C	4.95440	-0.03402	-1.11480

H	2.88189	2.64154	-0.68791
H	2.63895	-1.30628	1.01922
H	4.96799	1.98053	-1.90043
H	4.68425	-1.96294	-0.17891
H	5.86472	-0.33050	-1.64229
C	0.54513	0.21718	1.66019
H	1.13529	-0.52195	2.21966
I	-0.59824	-1.22419	0.30057
C	-2.12740	0.24437	-0.08970
C	-3.22502	-0.19869	-0.81570
C	-2.00186	1.55543	0.35526
C	-4.25415	0.70529	-1.08159
C	-3.04502	2.44223	0.07492
C	-4.17005	2.02243	-0.63234
H	-3.23519	-1.23173	-1.17447
H	-1.11498	1.92317	0.87258
H	-5.12511	0.36922	-1.65014
H	-2.96053	3.47782	0.41397
H	-4.97837	2.72683	-0.84304
O	-1.72578	-2.61060	-0.91607
H	-0.16801	0.70903	2.33337
H	-2.28693	-3.08989	-0.28745

30

SI-D-prime

C	-1.45119	0.16149	0.59394
O	-0.72641	-0.39136	-0.38519
C	-2.89842	0.24209	0.24677
C	-3.41540	-0.60167	-0.74704
C	-3.76813	1.14671	0.87544
C	-4.76670	-0.56108	-1.08551
C	-5.11777	1.18741	0.53606
C	-5.62478	0.33165	-0.44395
H	-2.73717	-1.29324	-1.24962
H	-3.38214	1.83964	1.62627
H	-5.15207	-1.23203	-1.85798
H	-5.77859	1.90159	1.03446
H	-6.68410	0.36753	-0.71108
C	-0.96163	0.60345	1.77287
H	-1.62471	1.00335	2.54022
I	1.15751	-1.34734	-0.01941
C	2.06732	0.58233	-0.18153
C	3.37217	0.71722	0.27201
C	1.34039	1.62684	-0.73731
C	3.97205	1.97371	0.16782
C	1.96379	2.87173	-0.83455
C	3.27180	3.04682	-0.38236
H	3.90107	-0.14235	0.68938
H	0.31846	1.46944	-1.08606
H	4.99671	2.10676	0.52386
H	1.41416	3.70920	-1.27130
H	3.74903	4.02651	-0.46123
O	3.00024	-2.18228	0.31898
H	0.10429	0.56086	2.00738
H	3.43548	-2.29129	-0.54216

6. NMR Spectra

***N*-Methoxy-*N*-methylbenzamide (3a)**

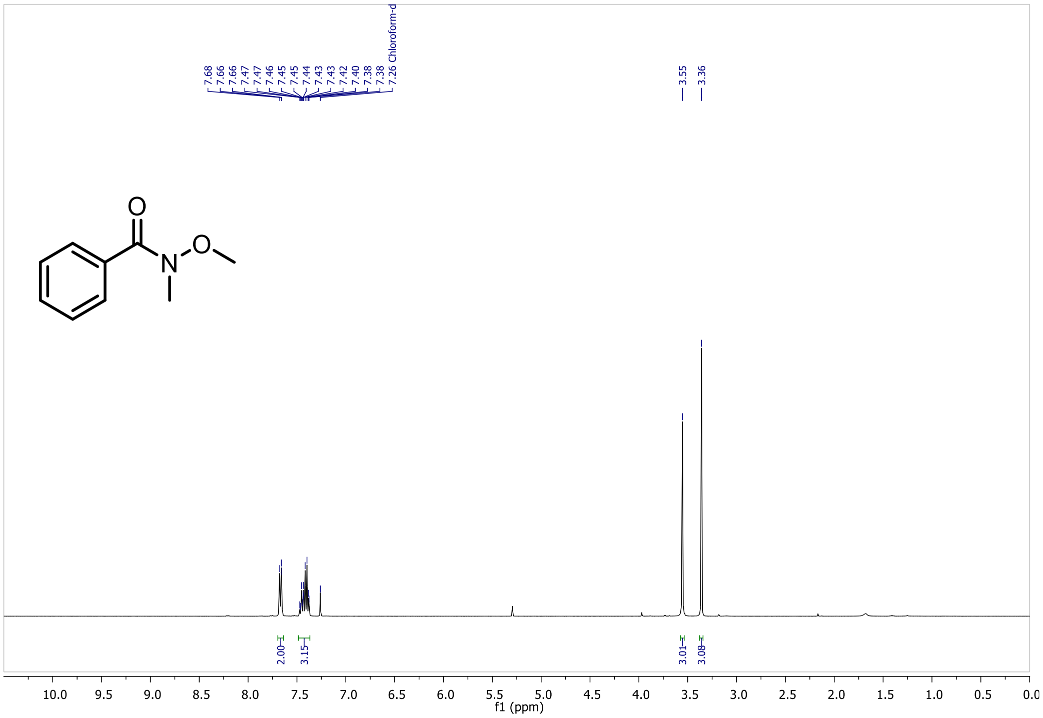

3-Fluoro-*N*-methoxy-*N*-methylbenzamide (3b)

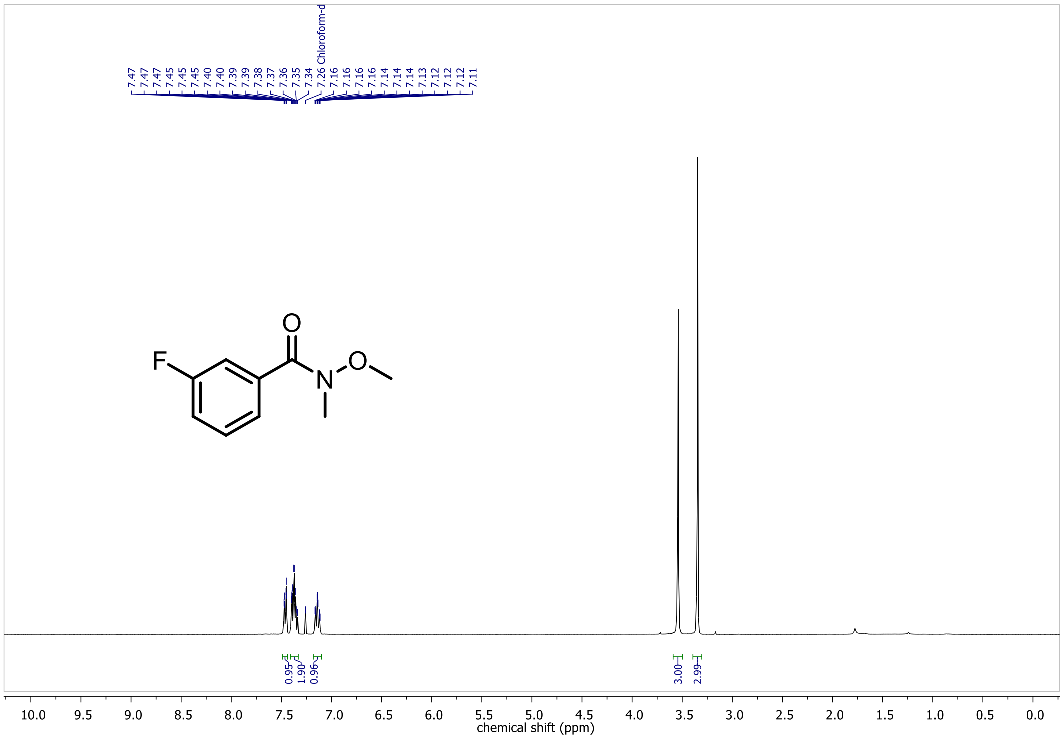

4-Fluoro-*N*-methoxy-*N*-methylbenzamide (3c)

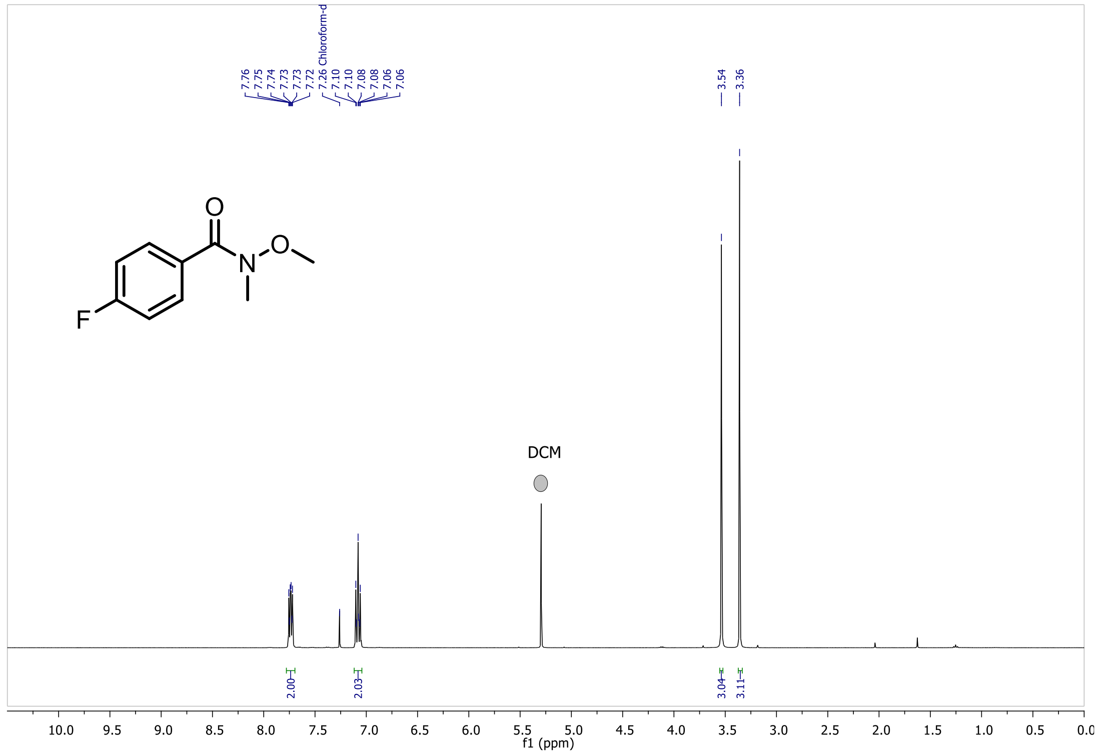

***N*-Methoxy-*N*-methyl-3,5-bis(trifluoromethyl)benzamide (3d)**

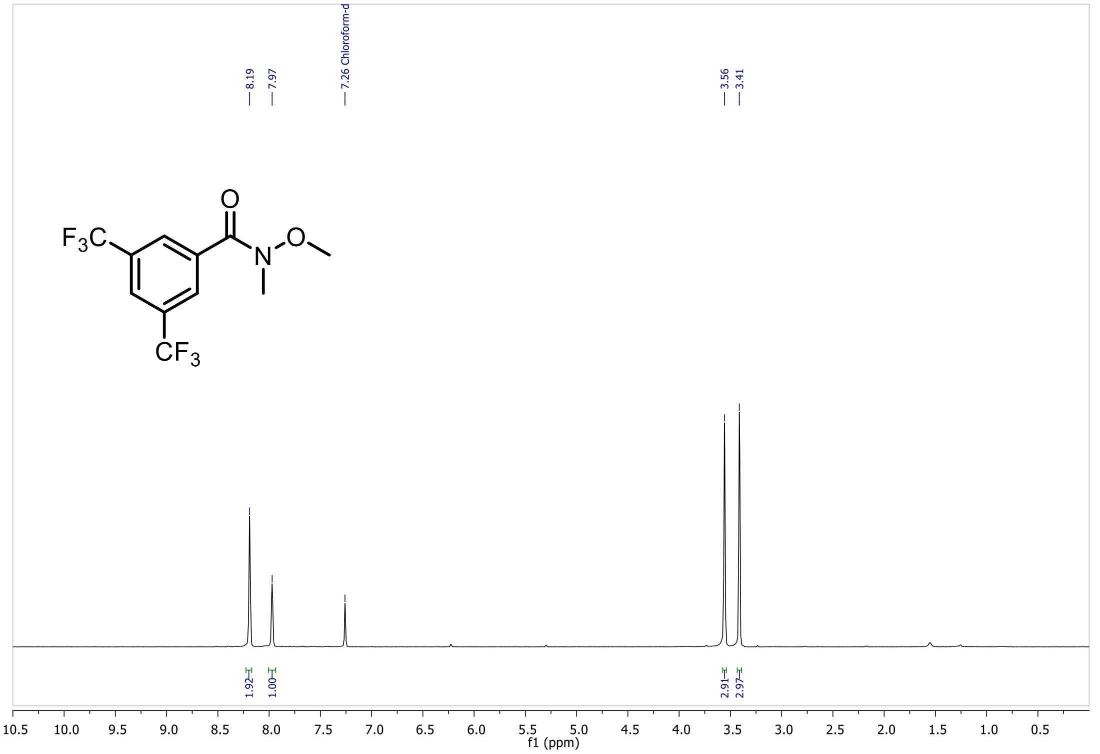

3,5-Difluoro-*N*-methoxy-*N*-methylbenzamide (3e)

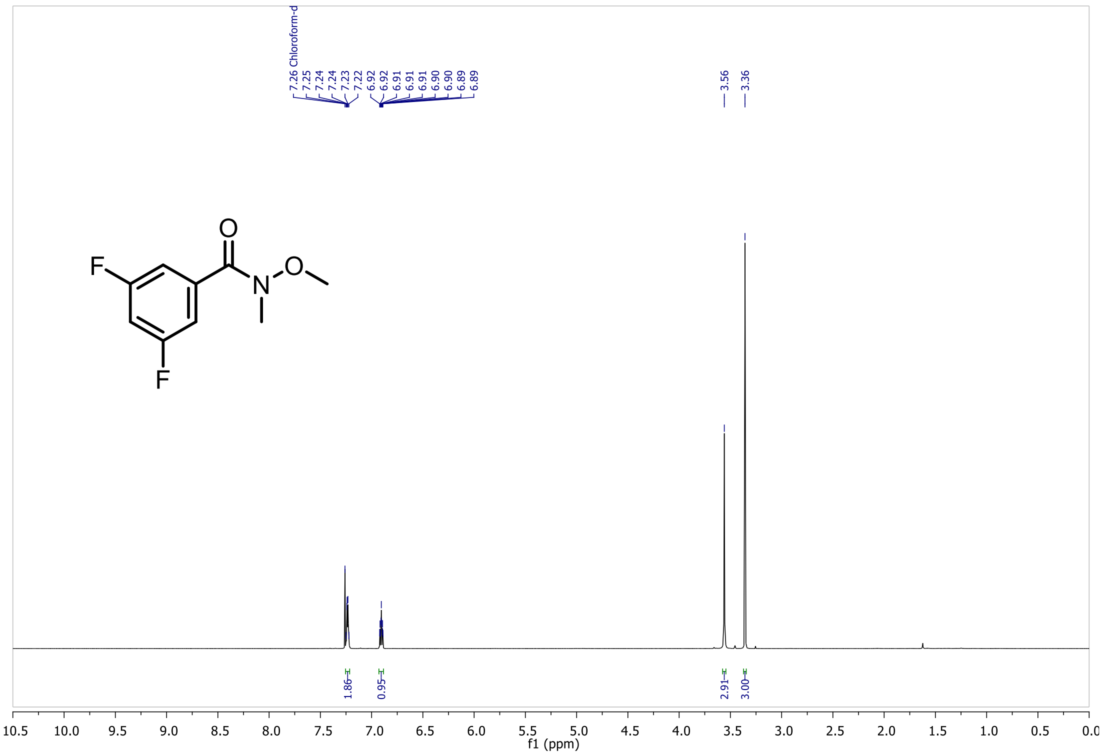

***N*-Methoxy-*N*-methyl-4-(trifluoromethyl)benzamide (3f)**

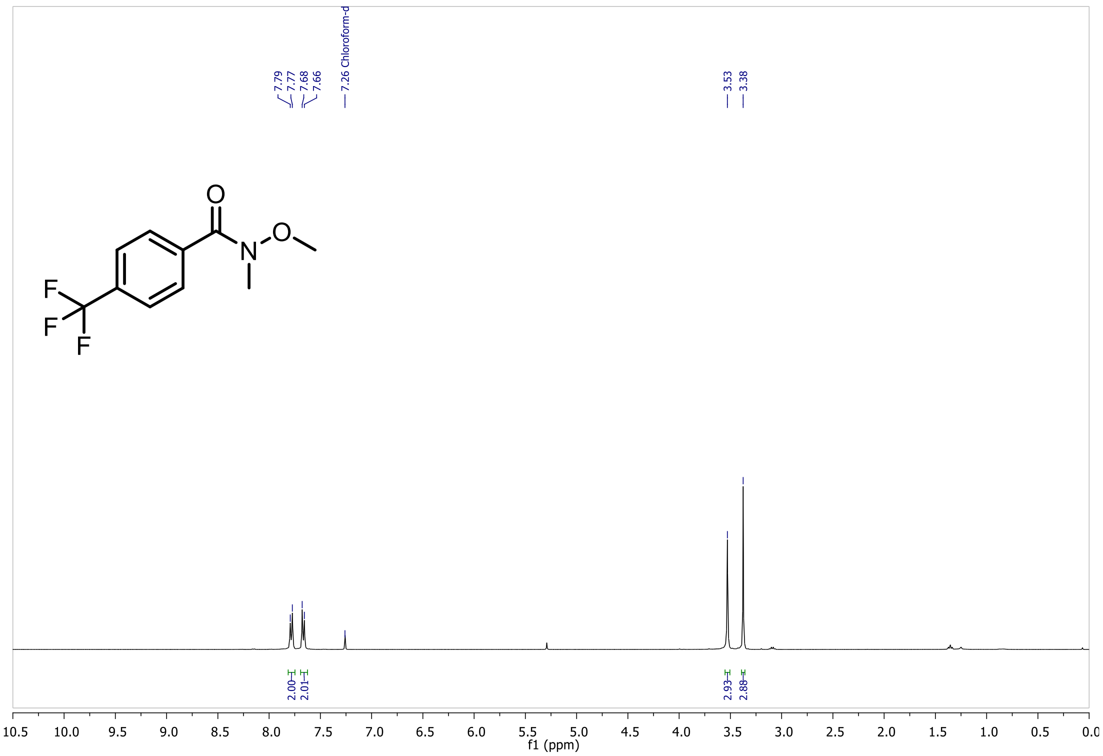

3,4-Dichloro-*N*-methoxy-*N*-methylbenzamide (3g)

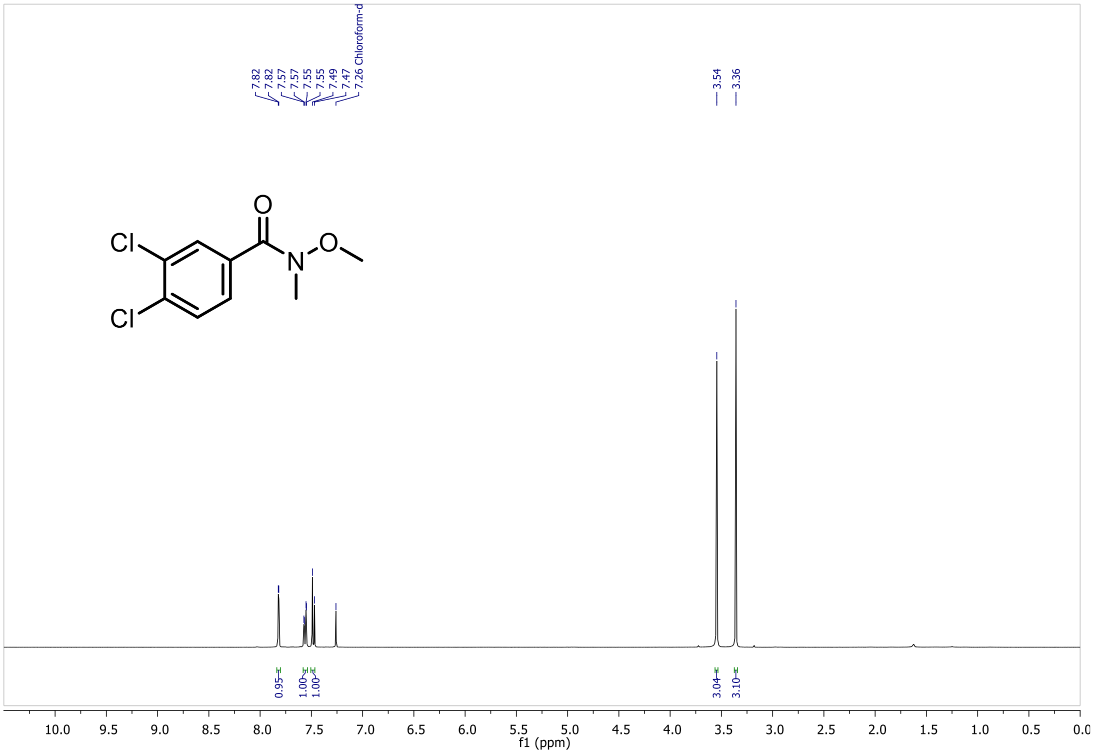

3-Chloro-*N*-methoxy-*N*-methylbenzamide (3h)

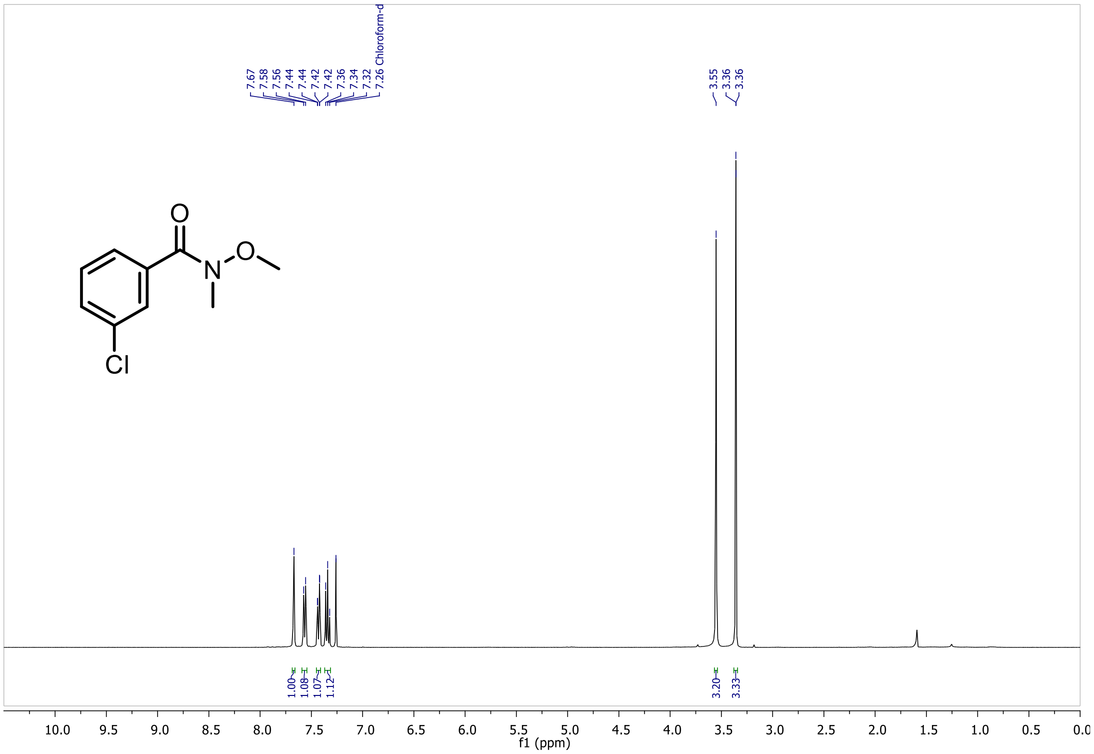

4-(*tert*-Butyl)-*N*-methoxy-*N*-methylbenzamide (3j)

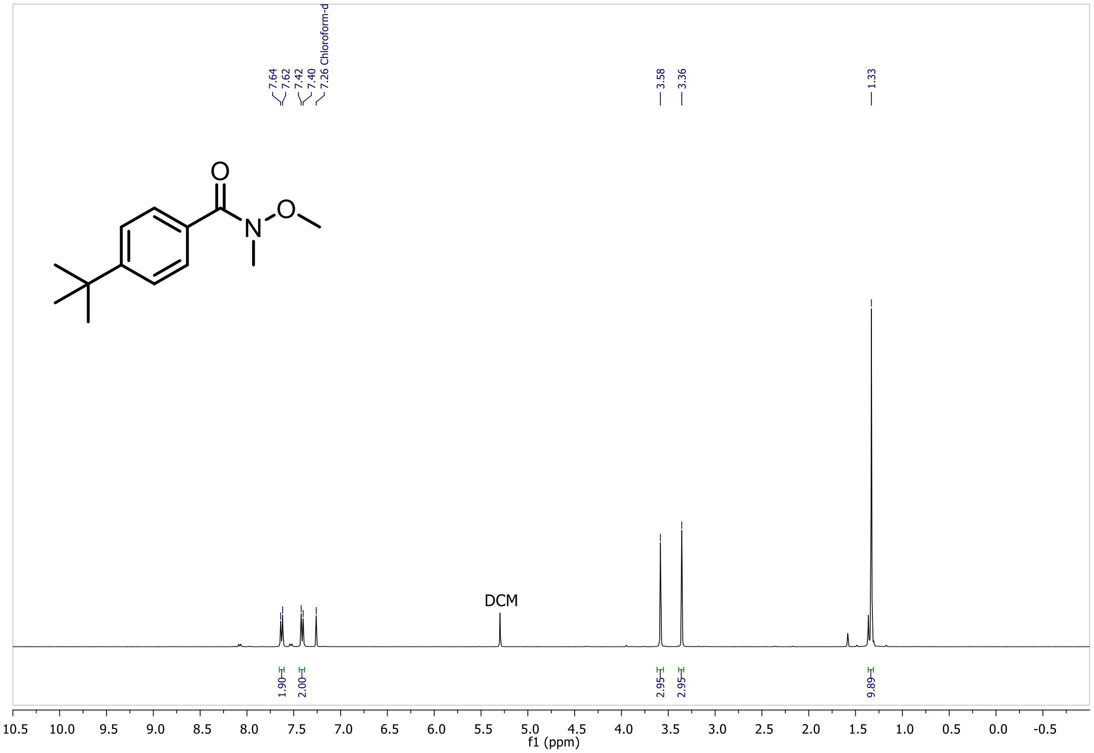

4-Cyano-*N*-methoxy-N-methylbenzamide (3k)

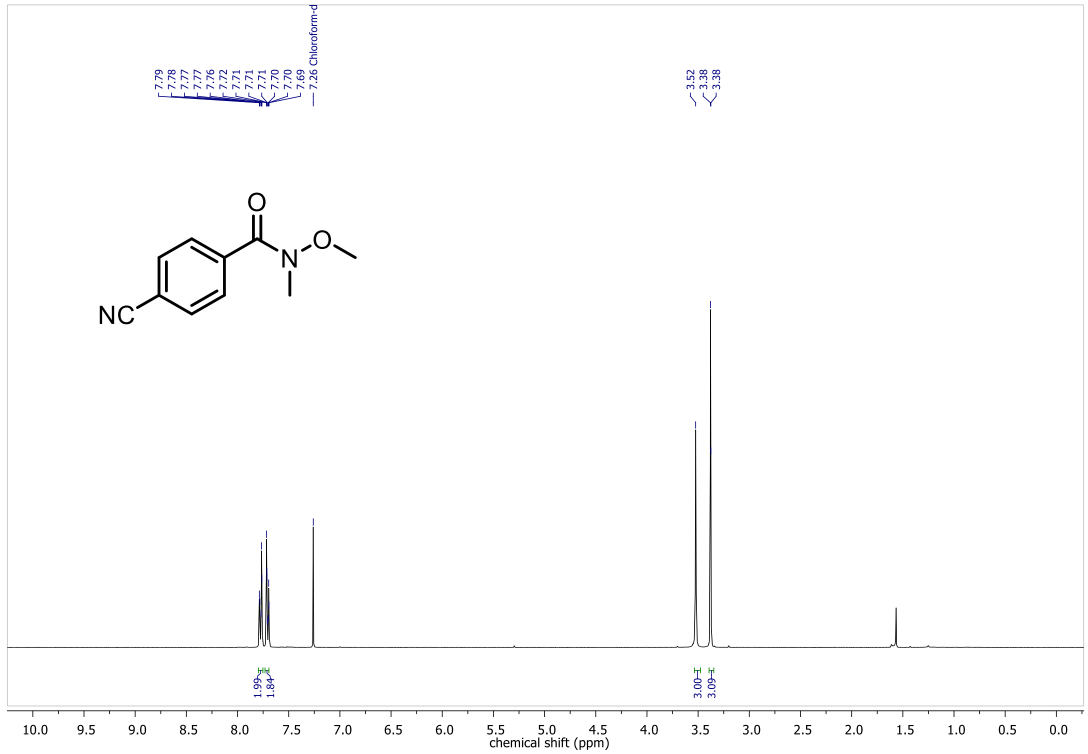

Methyl 4-(methoxy(methyl)carbamoyl)benzoate (3l)

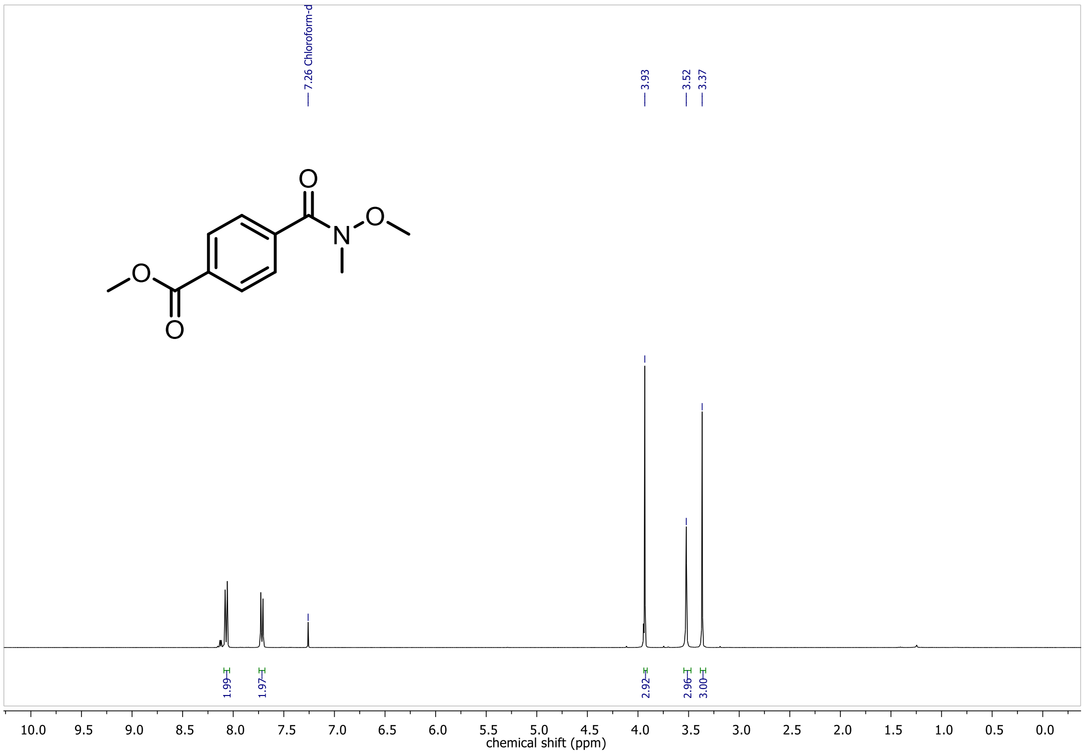

***N*-Methoxy-*N*-methyl-4-(trifluoromethoxy)benzamide (3m)**

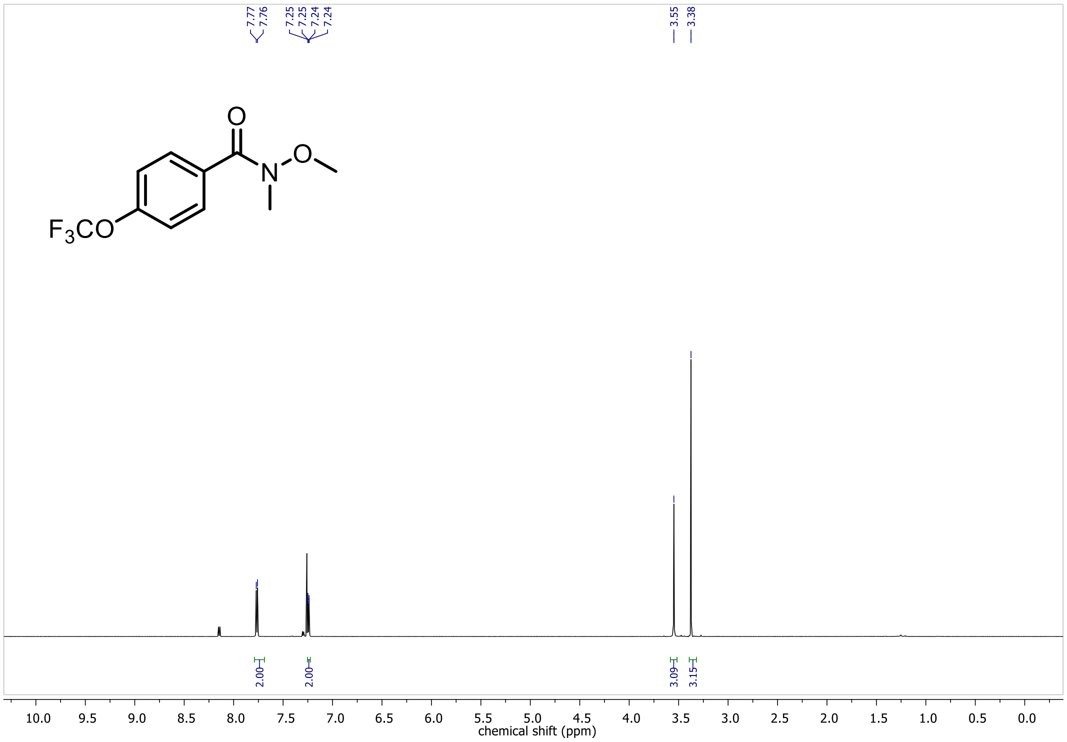

N-Methoxy-*N*-methylthiophene-2-carboxamide (3n)

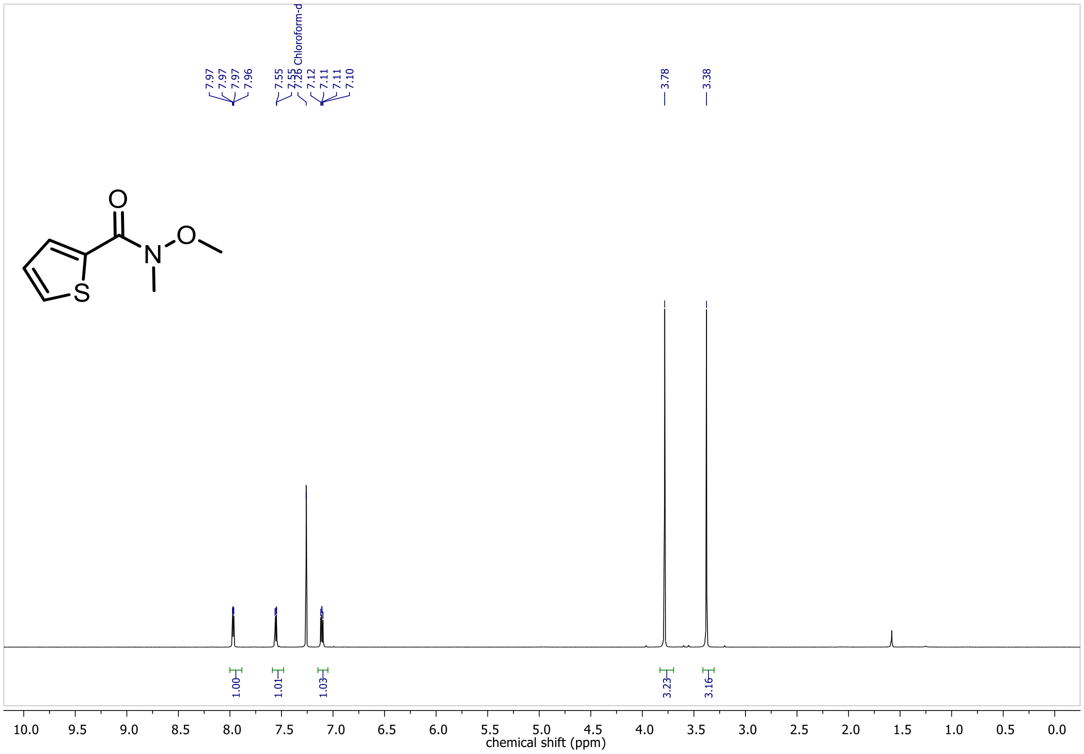

N-Methoxy-*N*-methylfuran-2-carboxamide (3o)

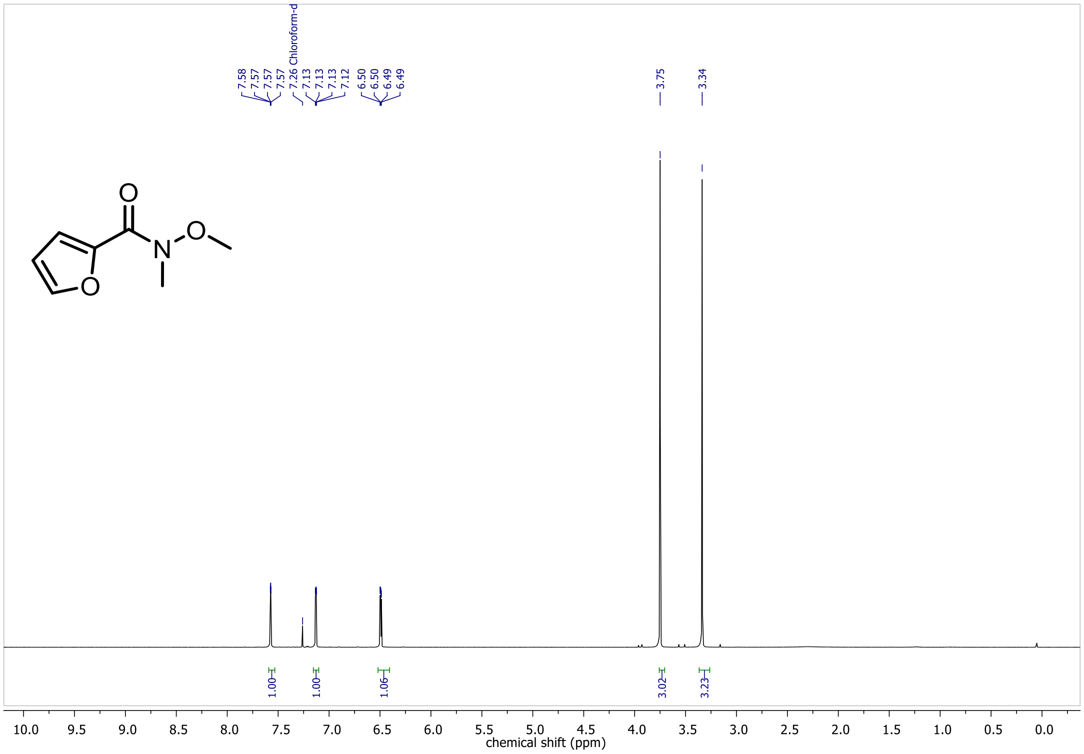

N-Methoxy-*N*-methyladamantane-1-carboxamide (3p)

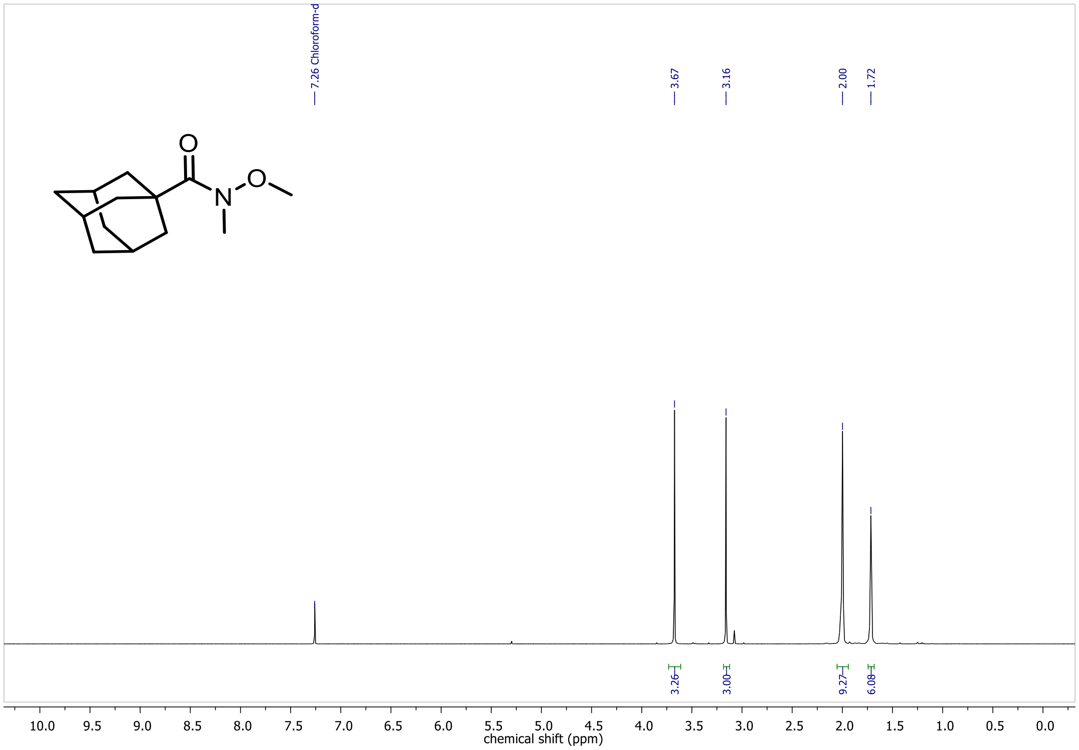

N-methoxy-N-methylcyclohexanecarboxamide (3r)

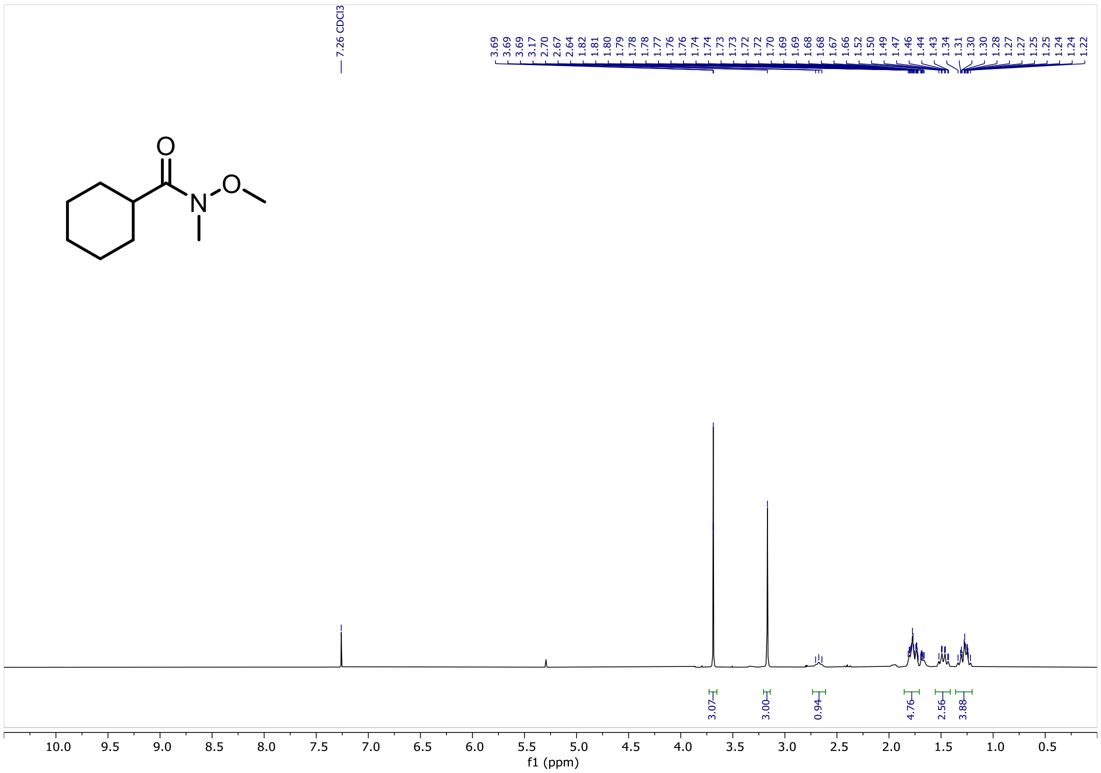

N-Methoxy-*N*-methyl-6-phenylhept-6-enamide (3t)

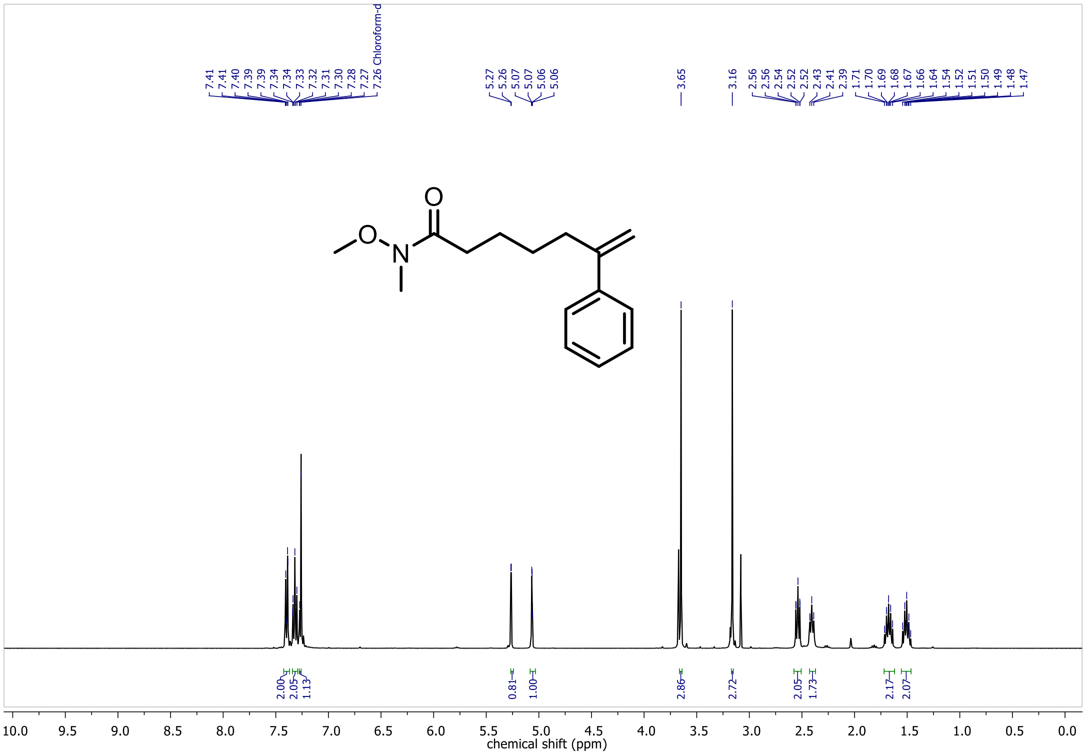

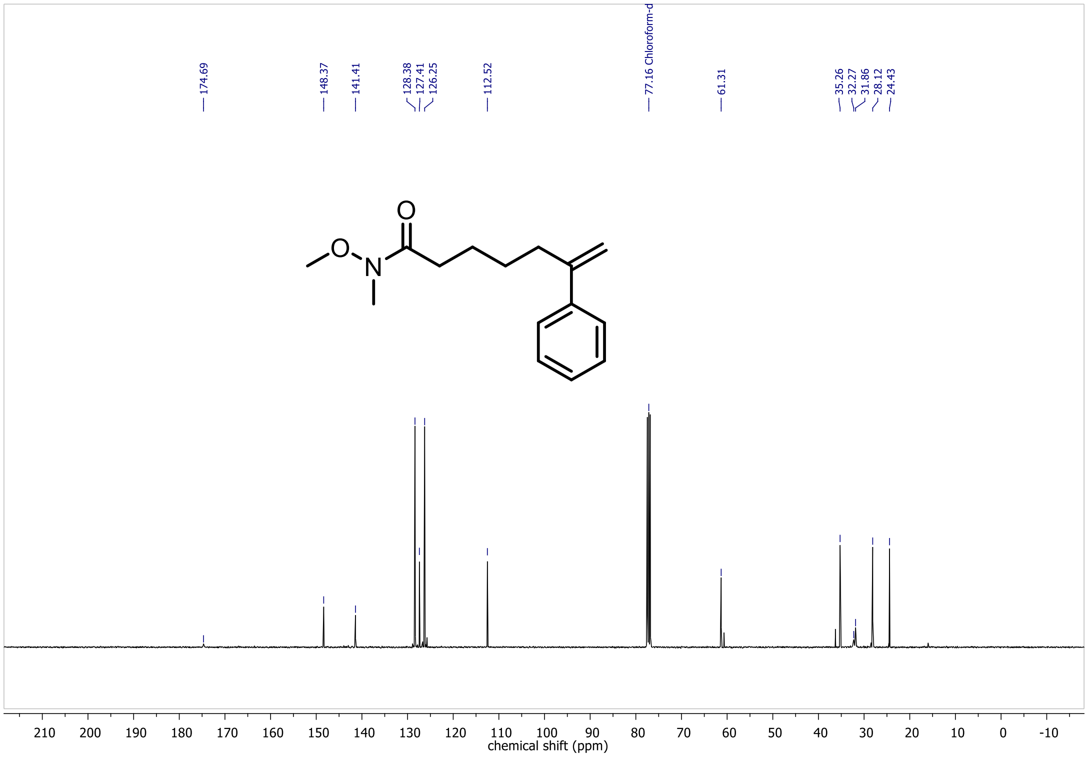

***N*-Methoxy-*N*-methyl-6-(naphthalen-2-yl)hept-6-enamide (3u)**

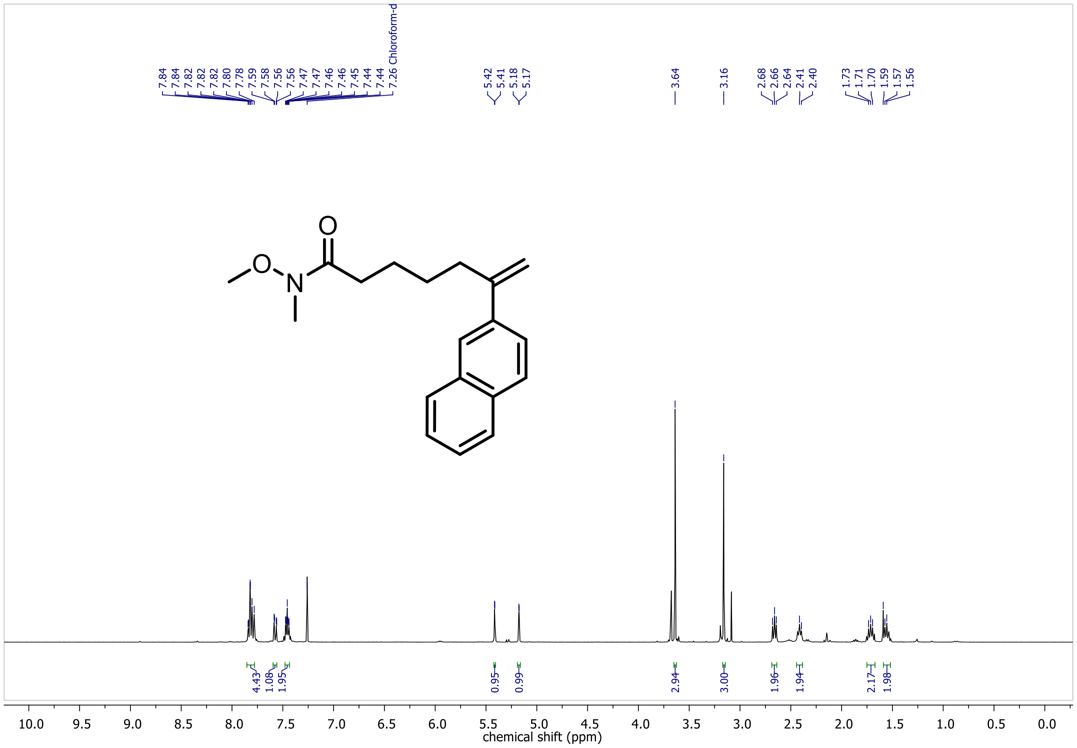

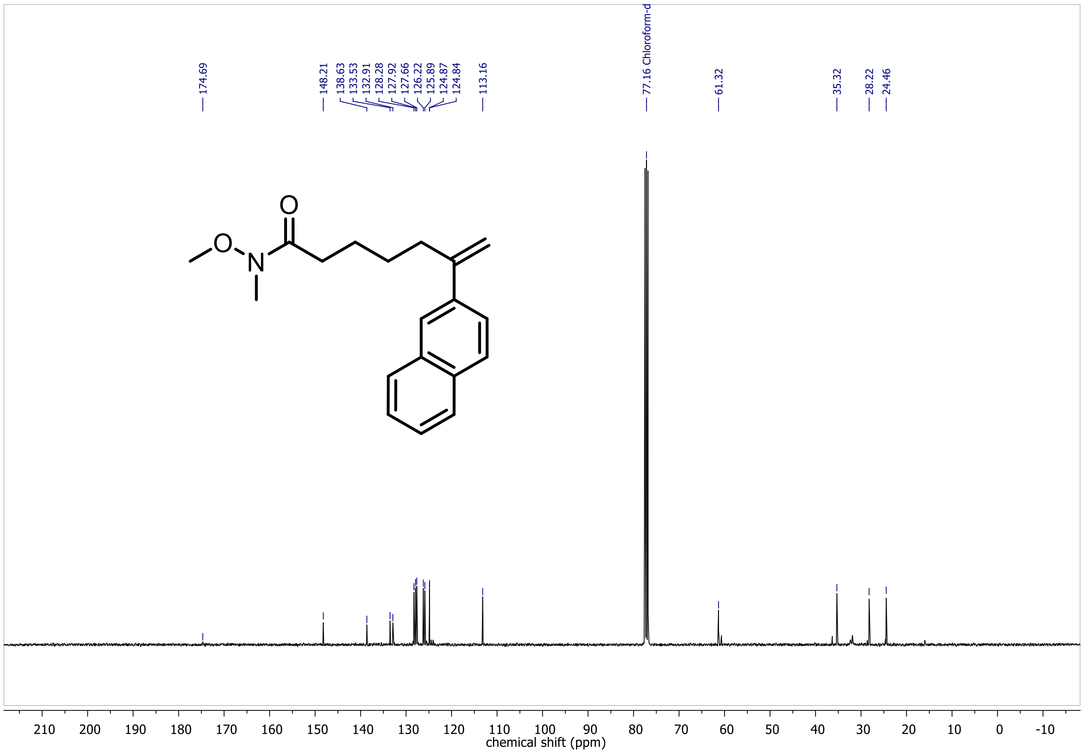

6-(Dibenzo[*b,d*]furan-1-yl)-*N*-methoxy-*N*-methylhept-6-enamide (3v)

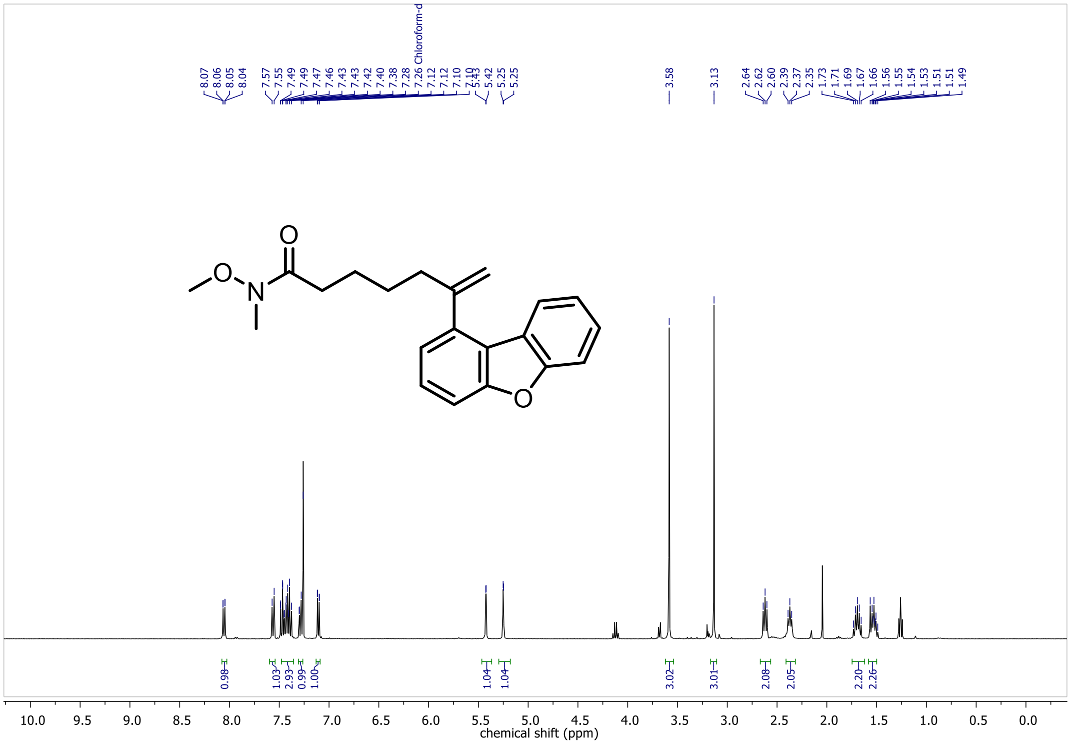

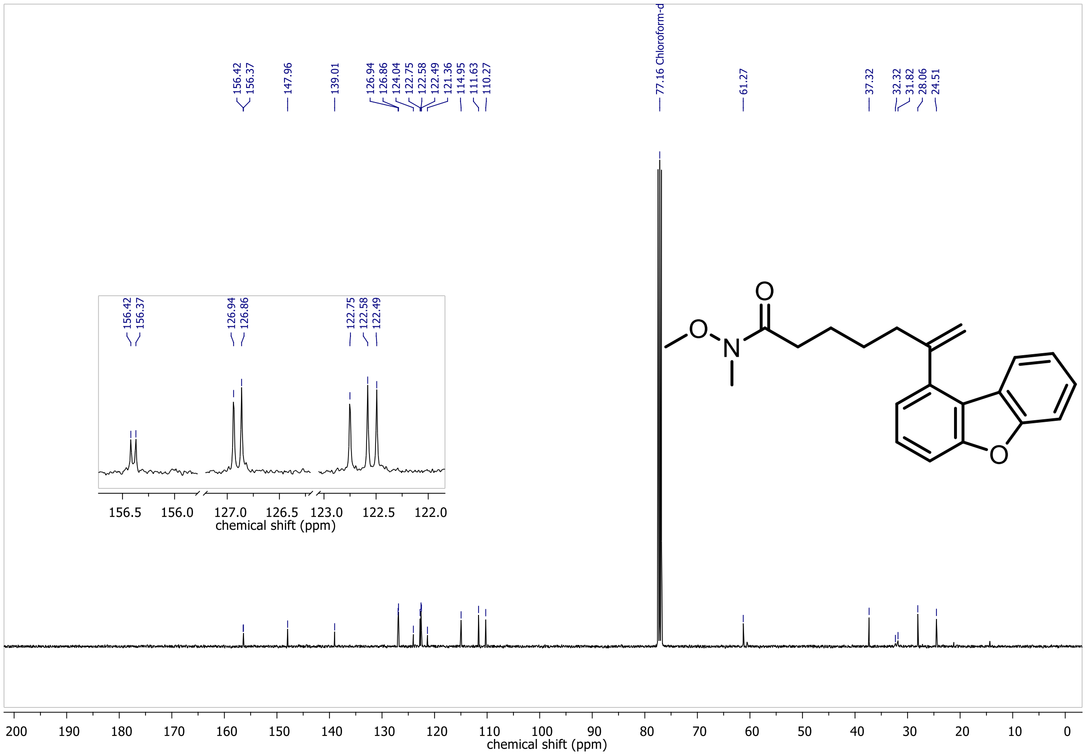

1-Phenylhept-6-en-1-one (4a)

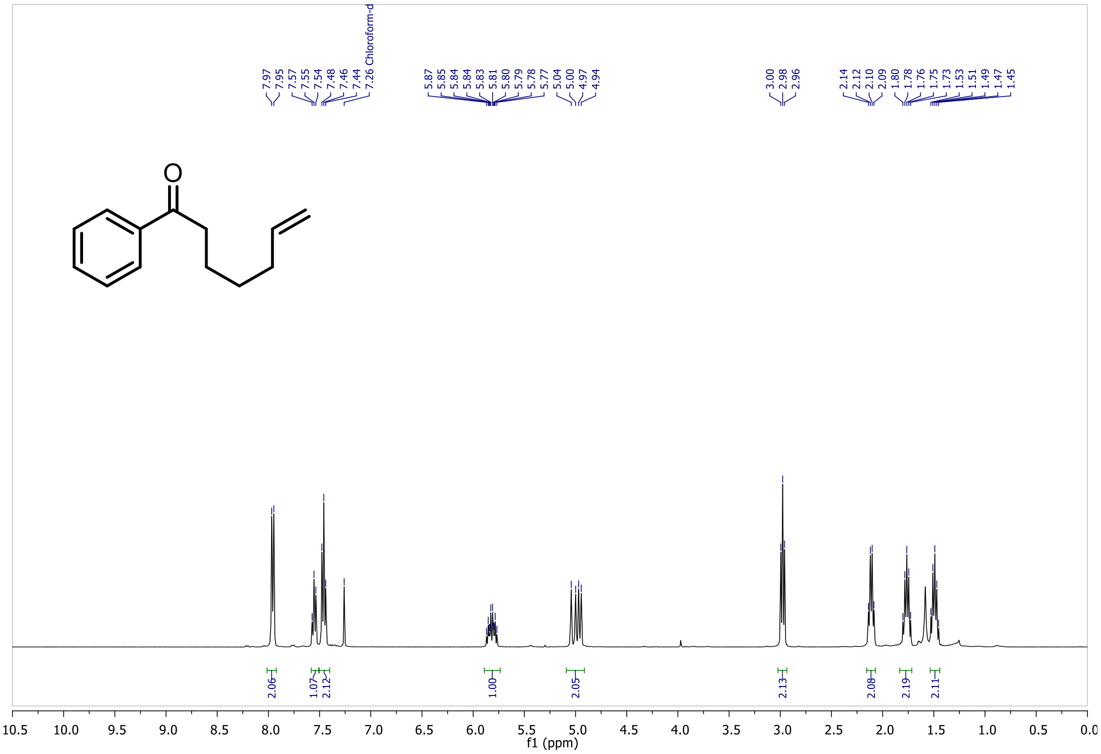

1-(3-Fluorophenyl)hept-6-en-1-one (4b)

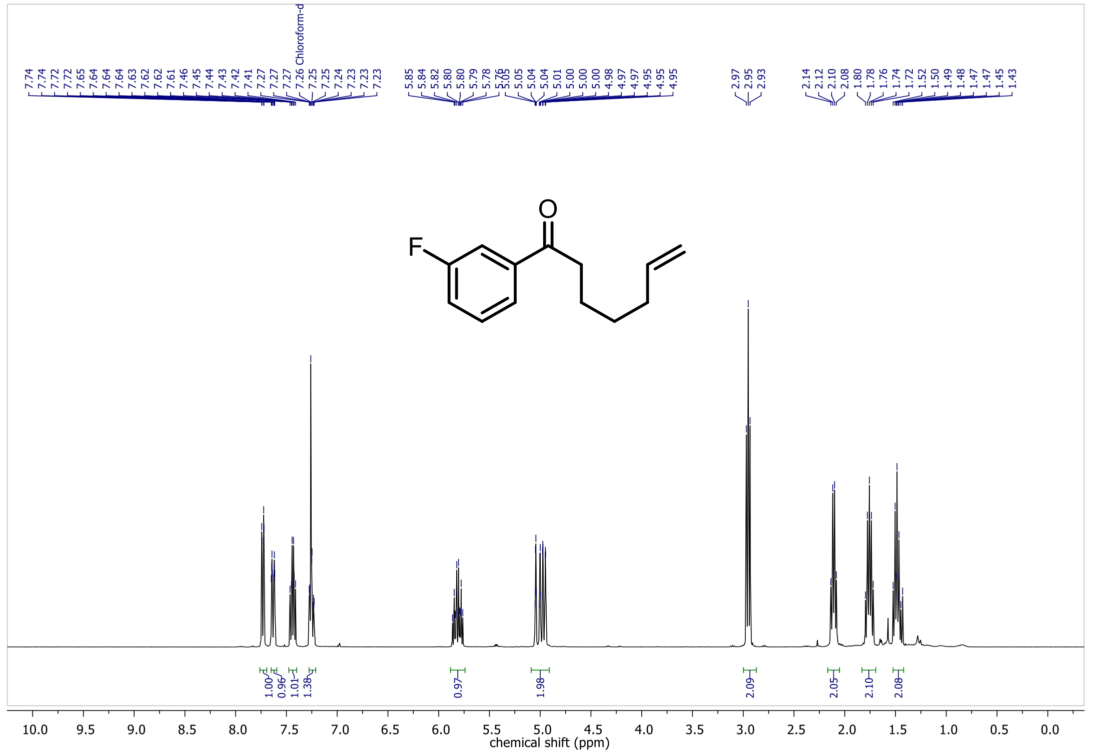

1-(4-Fluorophenyl)hept-6-en-1-one (4c)

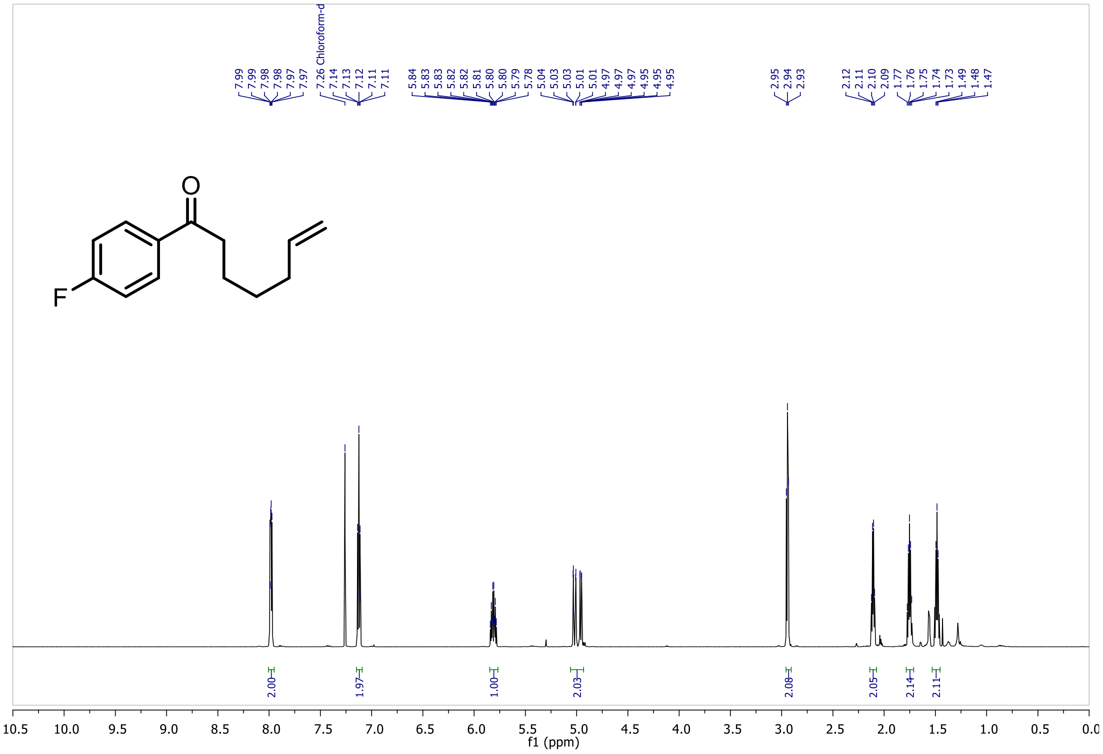

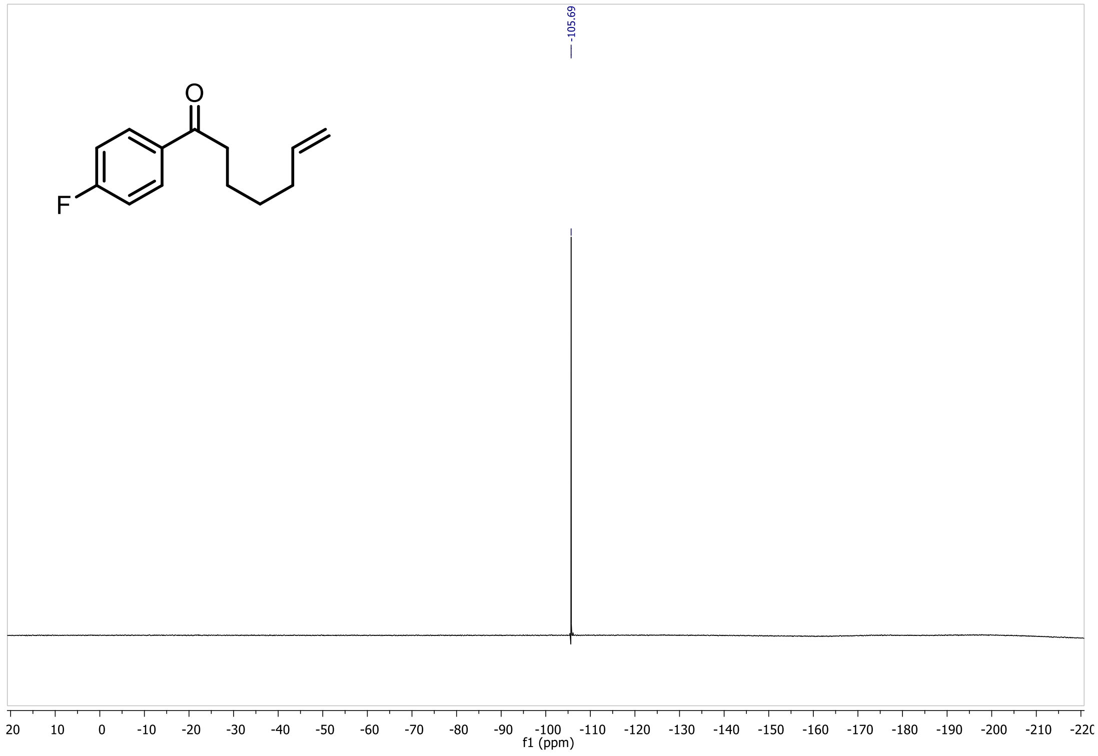

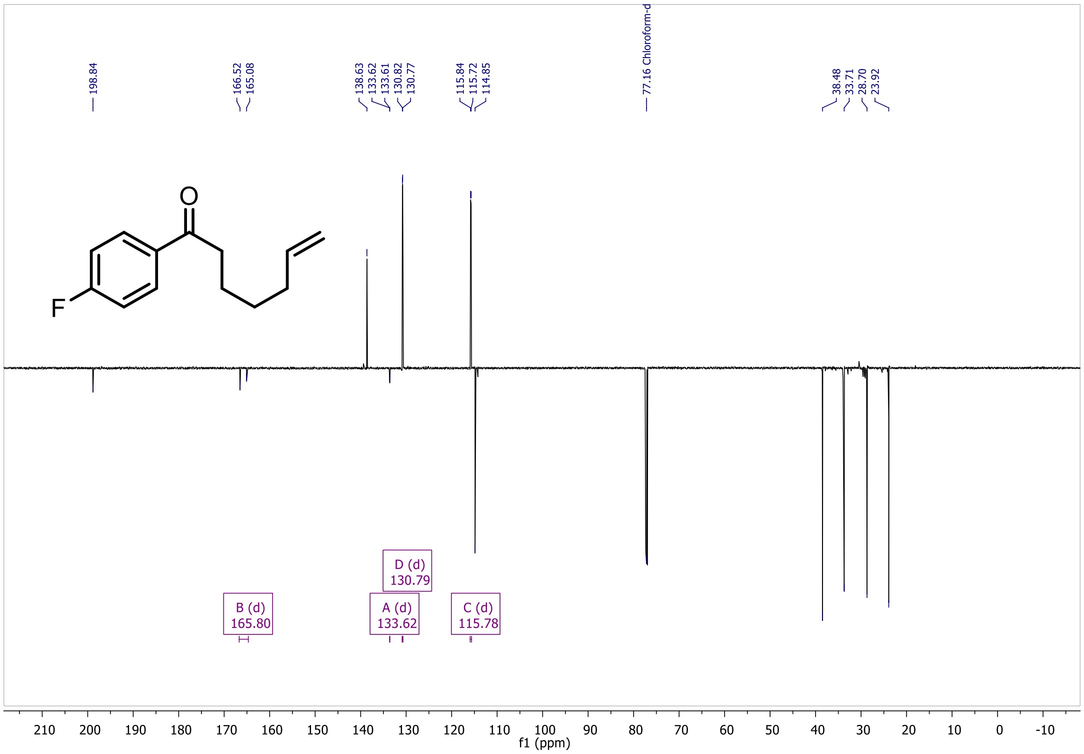
198.84
166.52
165.08
138.63
133.62
133.61
130.82
130.77
115.84
115.72
114.85
77.16 Chloroform-d
38.48
33.71
28.70
23.92
O
F
D (d)
130.79
B (d)
165.80
A (d)
133.62
C (d)
115.78
210
200
190
180
170
160
150
140
130
120
110
100
90
80
70
60
50
40
30
20
10
0
-10
f1 (ppm)

1-(3,5-Bis(trifluoromethyl)phenyl)hept-6-en-1-one (4d)

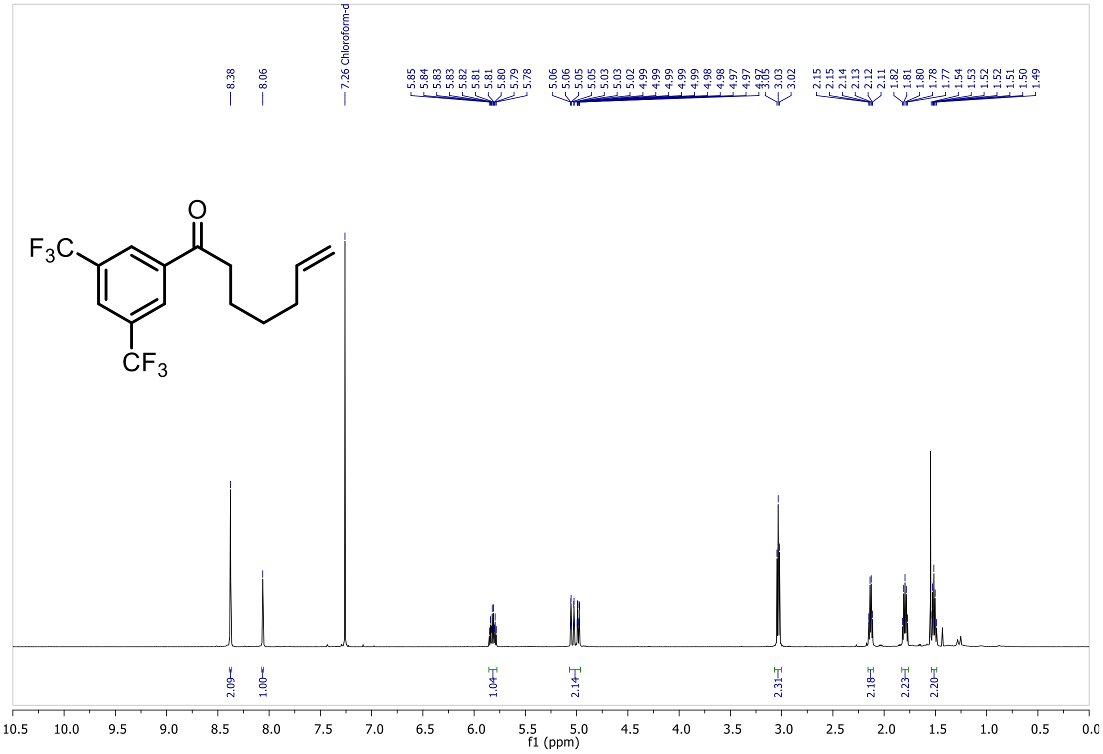

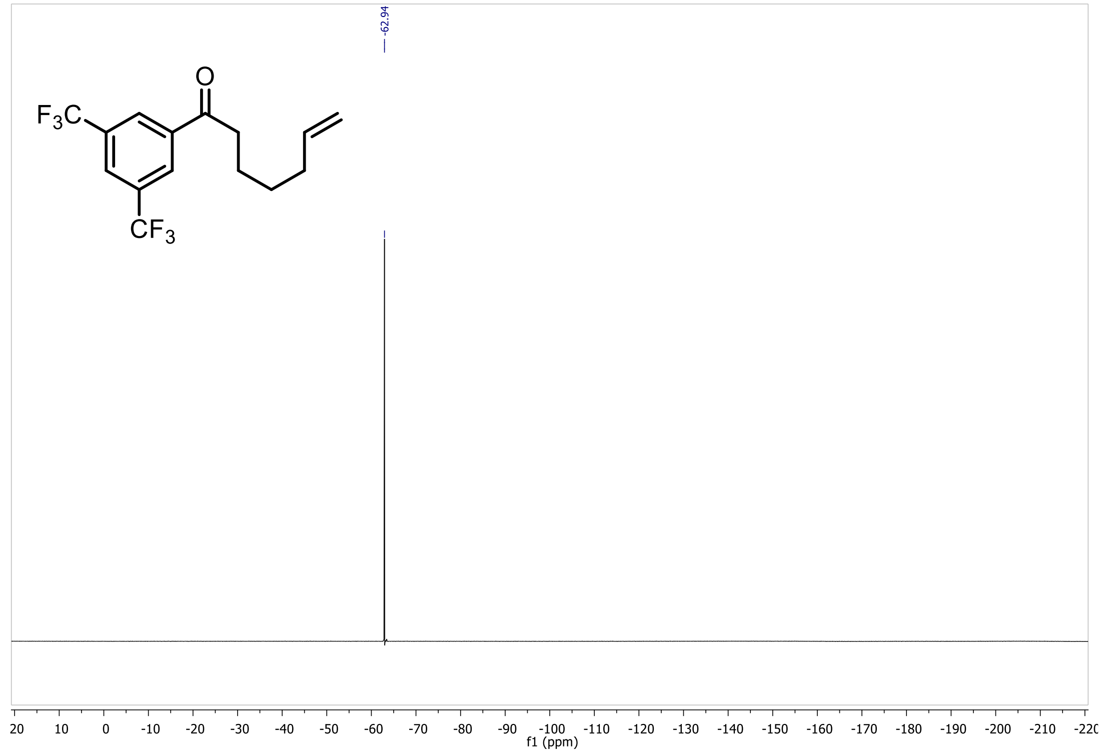

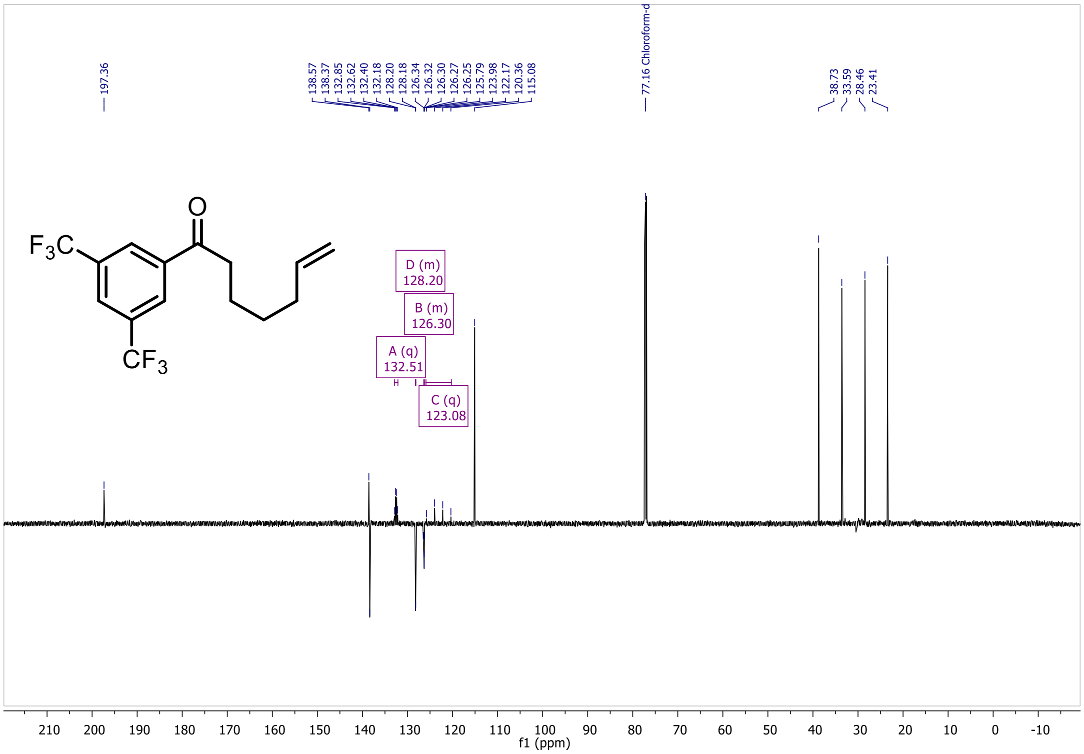

197.36
138.57
138.37
132.85
132.62
132.40
132.18
128.20
128.18
126.34
126.32
126.30
126.27
126.25
125.79
123.98
122.17
120.36
115.08
77.16 Chloroform-d
38.73
33.59
28.46
23.41
F3C
O
CF3
D (m)
128.20
B (m)
126.30
A (q)
132.51
C (q)
123.08
210 200 190 180 170 160 150 140 130 120 110 100 90 80 70 60 50 40 30 20 10 0 -10
f1 (ppm)

1-(3,5-Difluorophenyl)hept-6-en-1-one (4e)

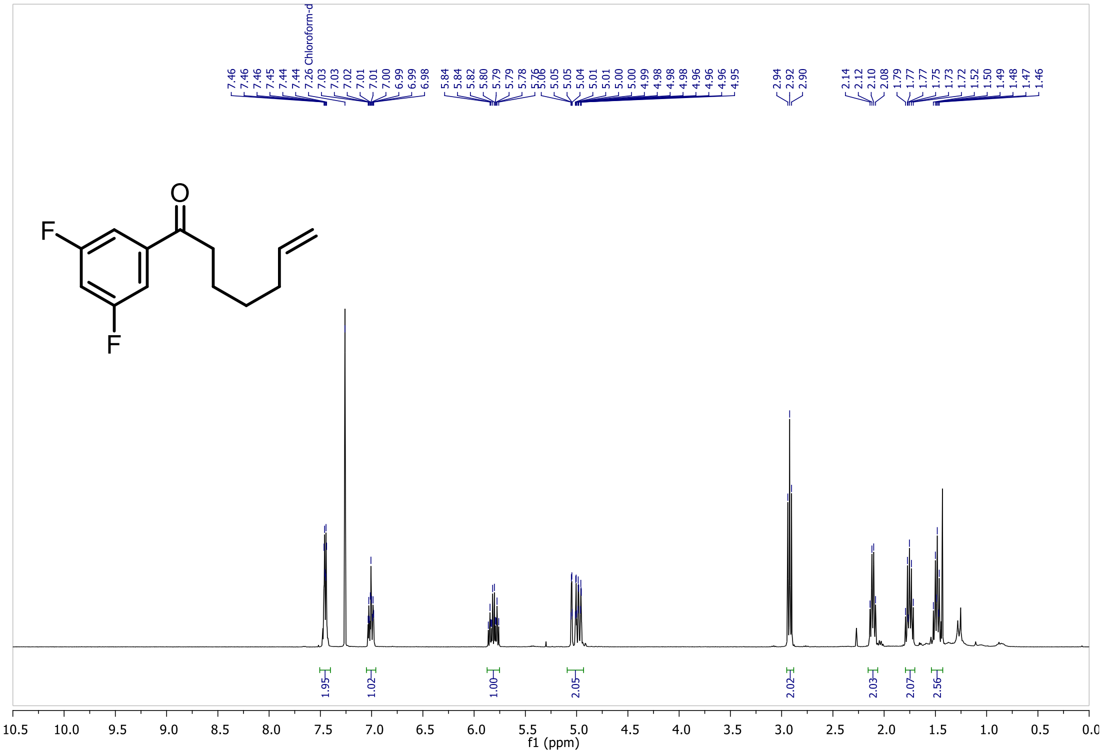

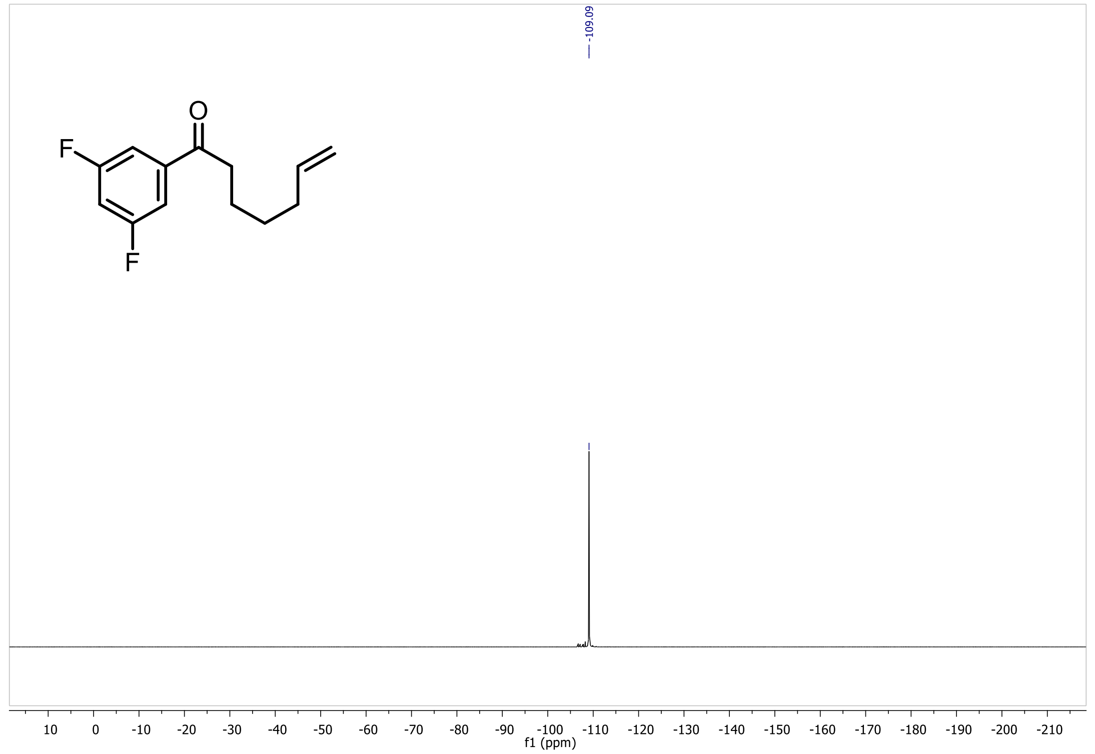

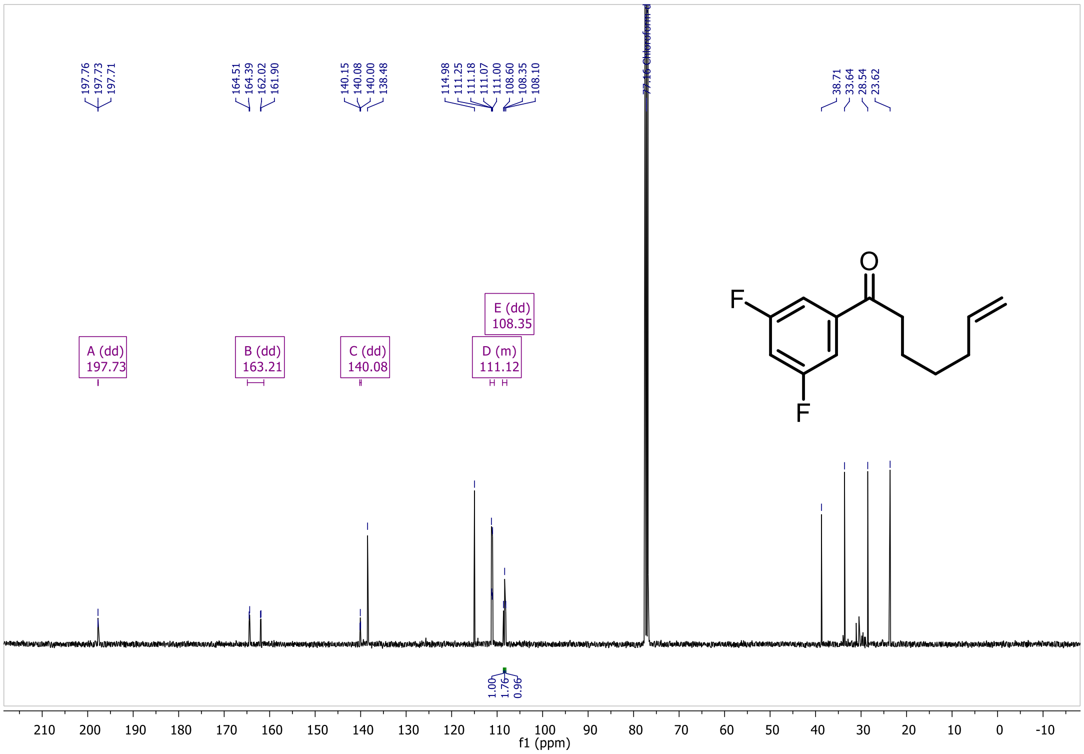

197.76
197.73
197.71
164.51
164.39
162.02
161.90
140.15
140.08
140.00
138.48
114.98
111.25
111.18
111.07
111.00
108.60
108.35
108.10
77.16 Chloroform-d
38.71
33.64
28.54
23.62
A (dd)
197.73
B (dd)
163.21
C (dd)
140.08
D (m)
111.12
E (dd)
108.35
1.00
1.76
0.96
f1 (ppm)
O
F
F

1-(4-(Trifluoromethyl)phenyl)hept-6-en-1-one (4f)

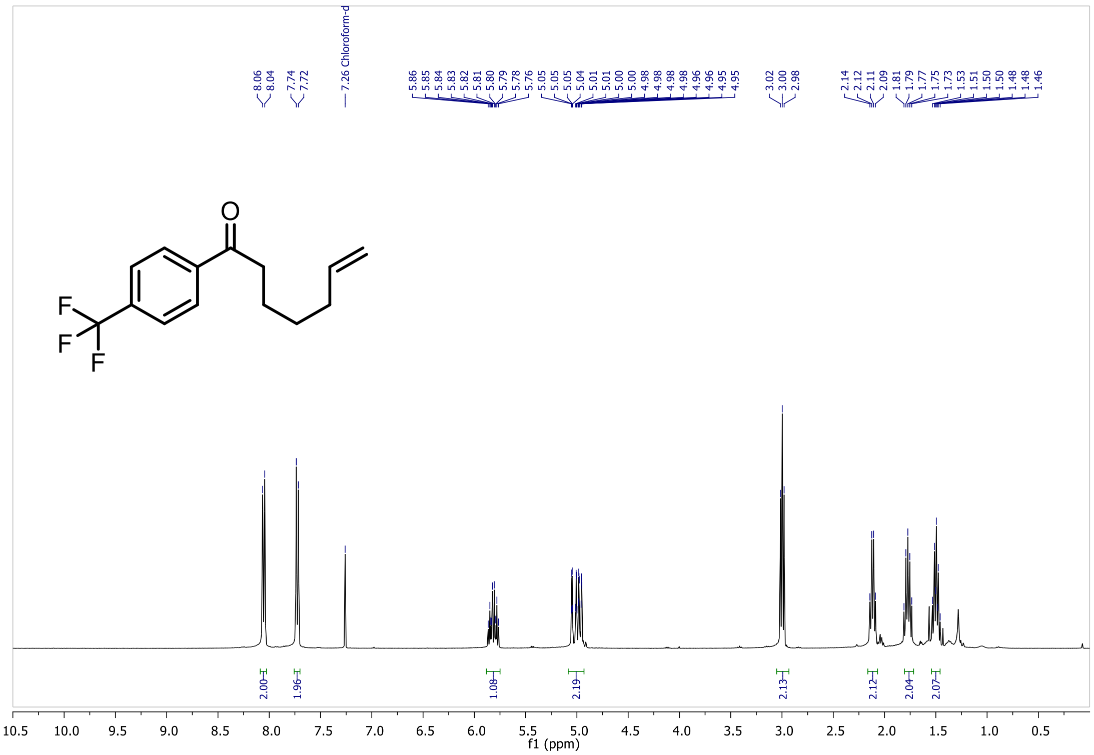

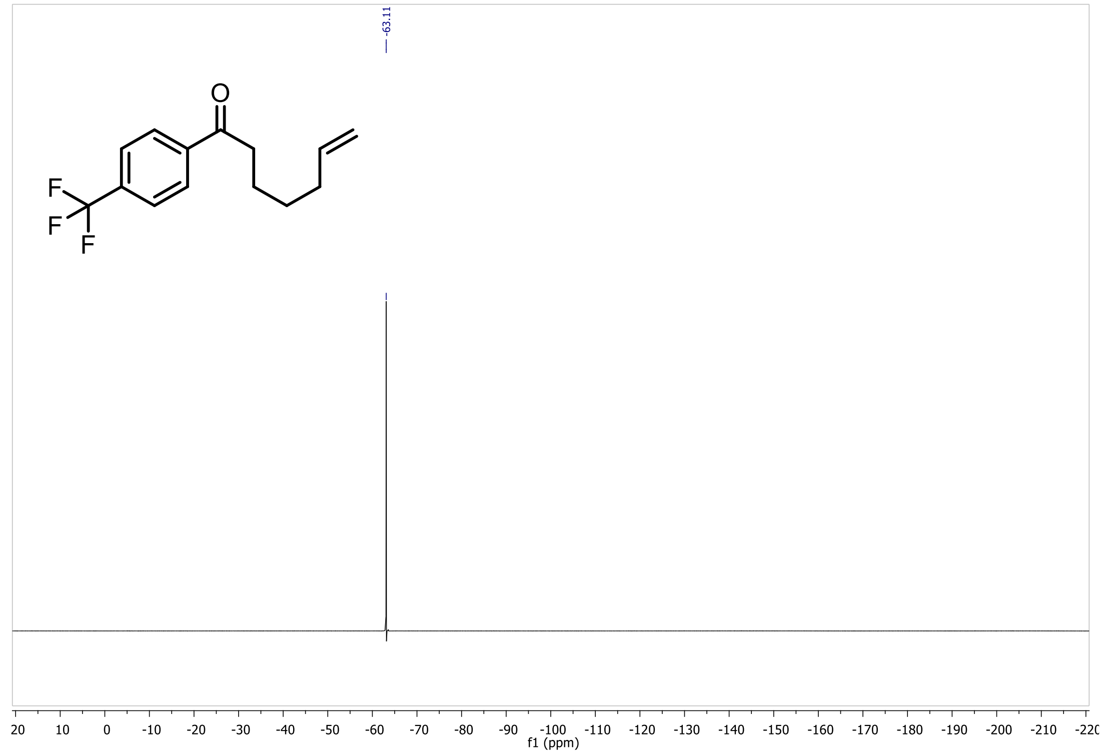

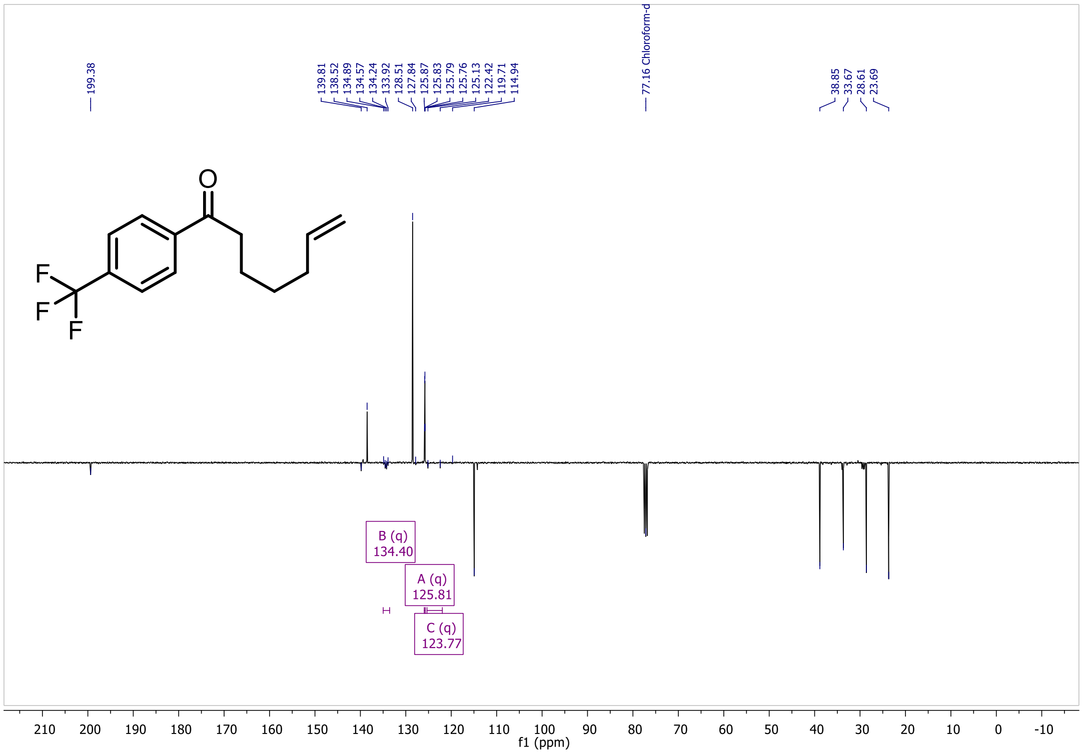

199.38
139.81
138.52
134.89
134.57
134.24
133.92
128.51
127.84
125.87
125.83
125.79
125.76
125.13
122.42
119.71
114.94
77.16 Chloroform-d
38.85
33.67
28.61
23.69
O
F
F
F
B (q)
134.40
A (q)
125.81
C (q)
123.77
210 200 190 180 170 160 150 140 130 120 110 100 90 80 70 60 50 40 30 20 10 0 -10
f1 (ppm)

1-(3,4-Dichlorophenyl)hept-6-en-1-one (4g)

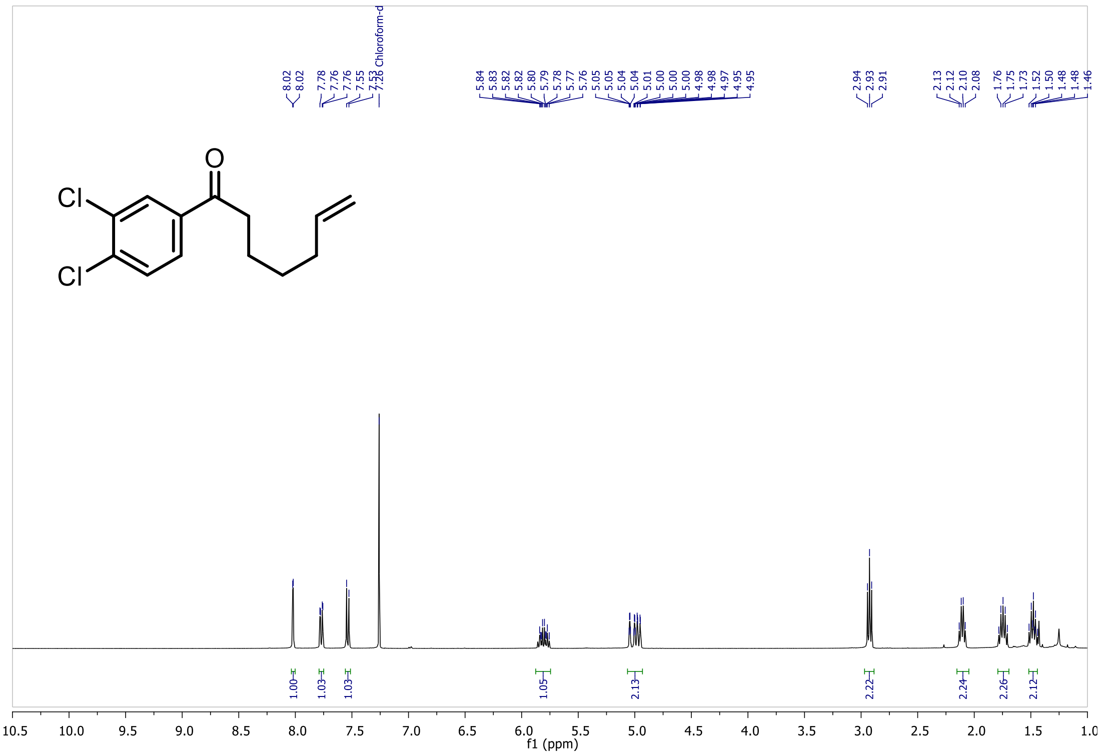

1-(3-Chlorophenyl)hept-6-en-1-one (4h)

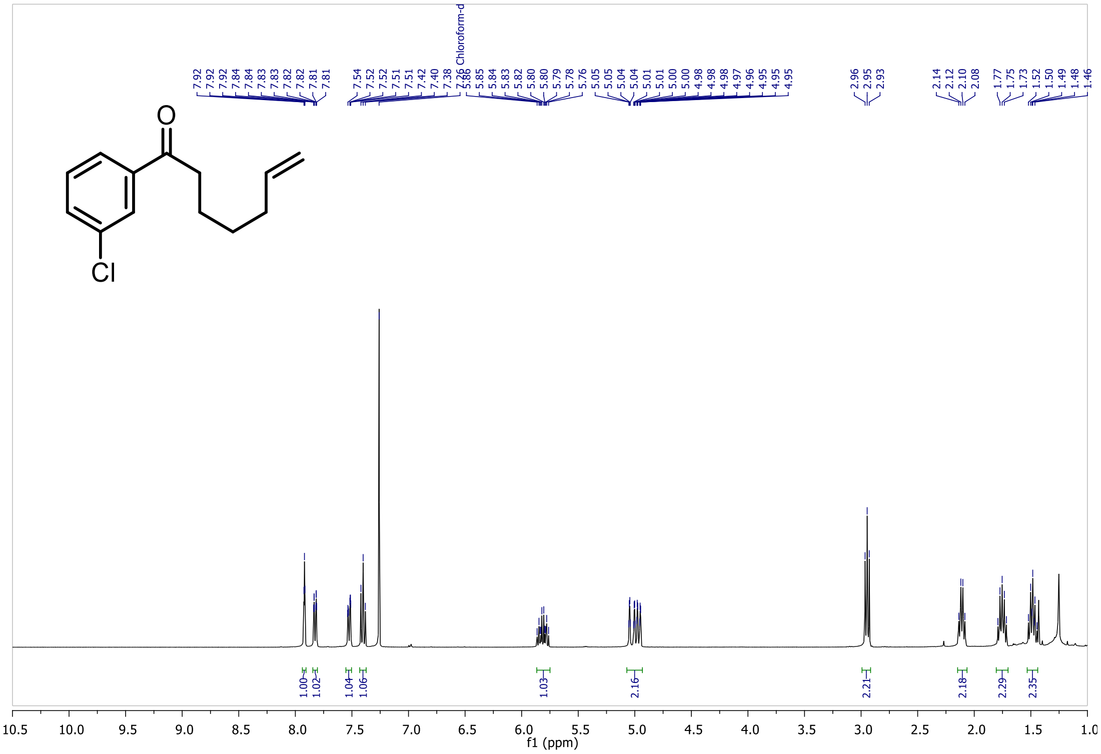

1-(4-(*tert*-Butyl)phenyl)hept-6-en-1-one (4j)

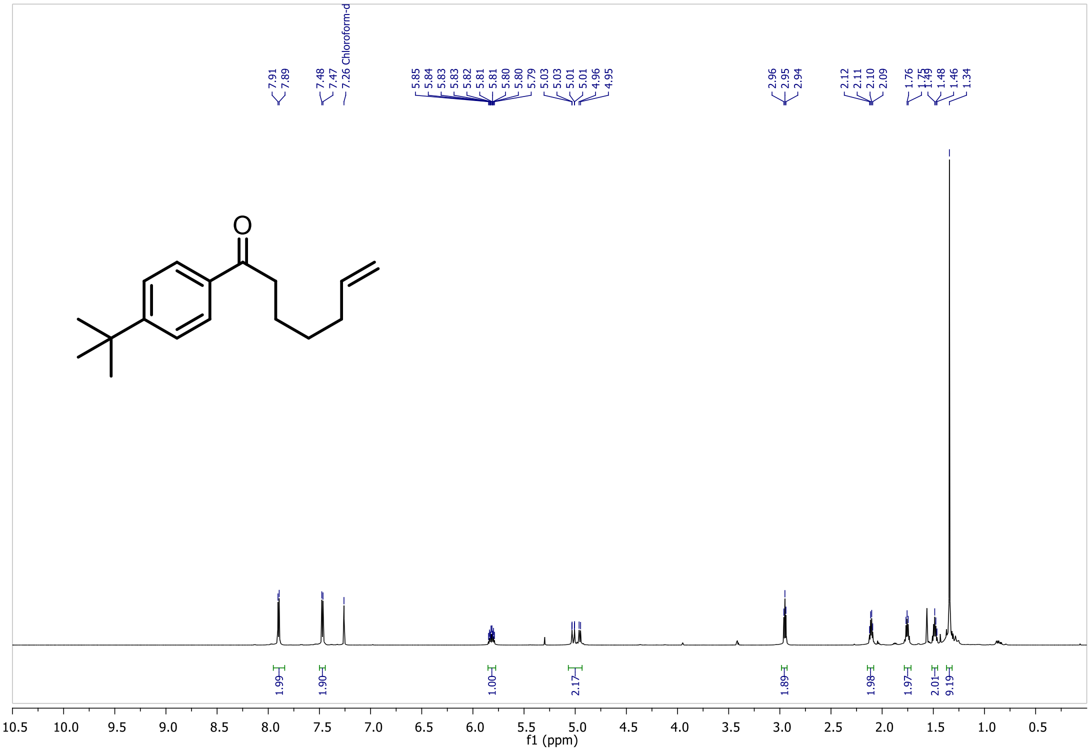

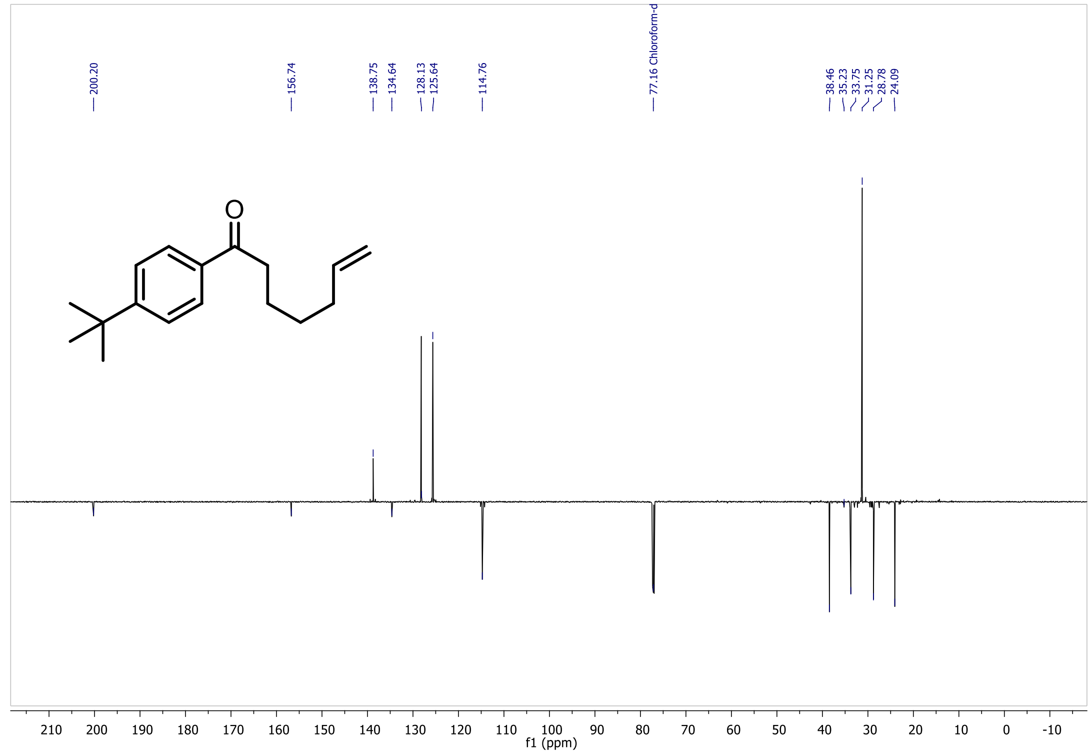

4-(Hept-6-enoyl)benzonitrile (4k)

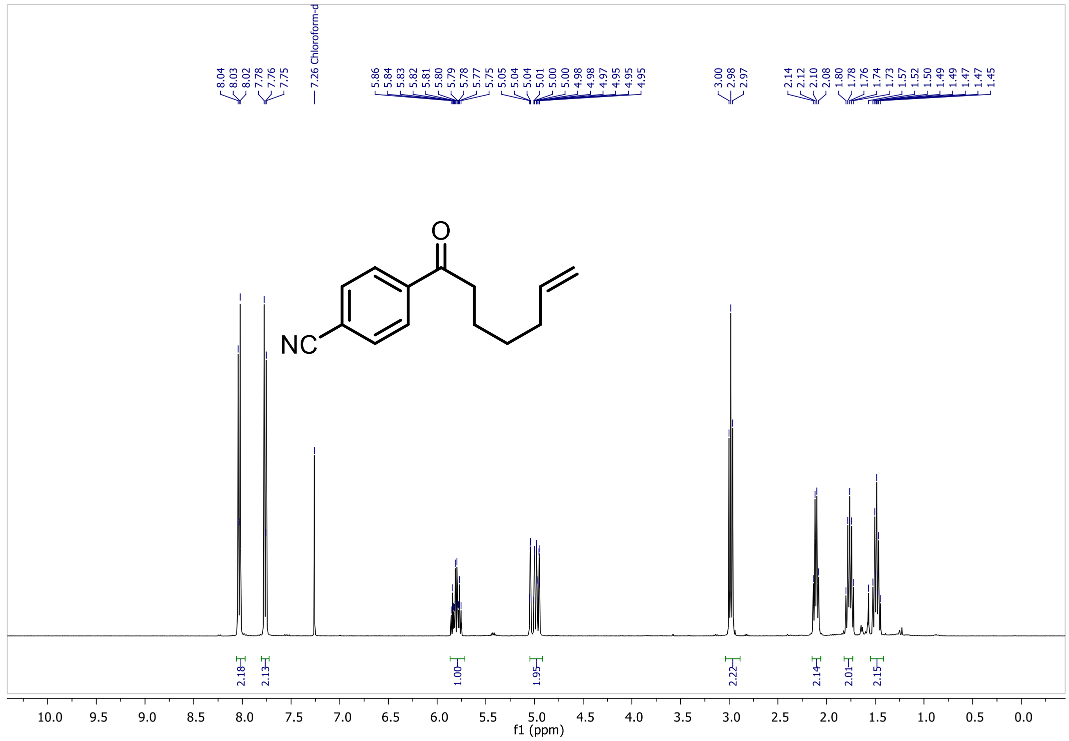

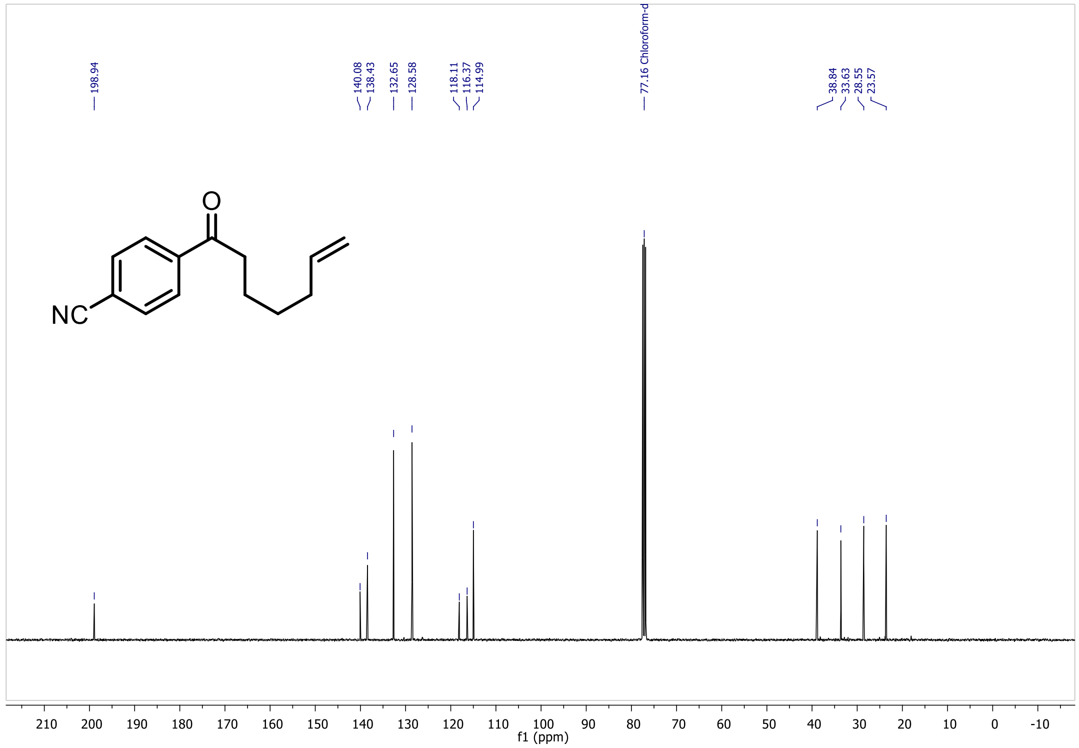

Methyl 4-(hept-6-enoyl)benzoate (4l)

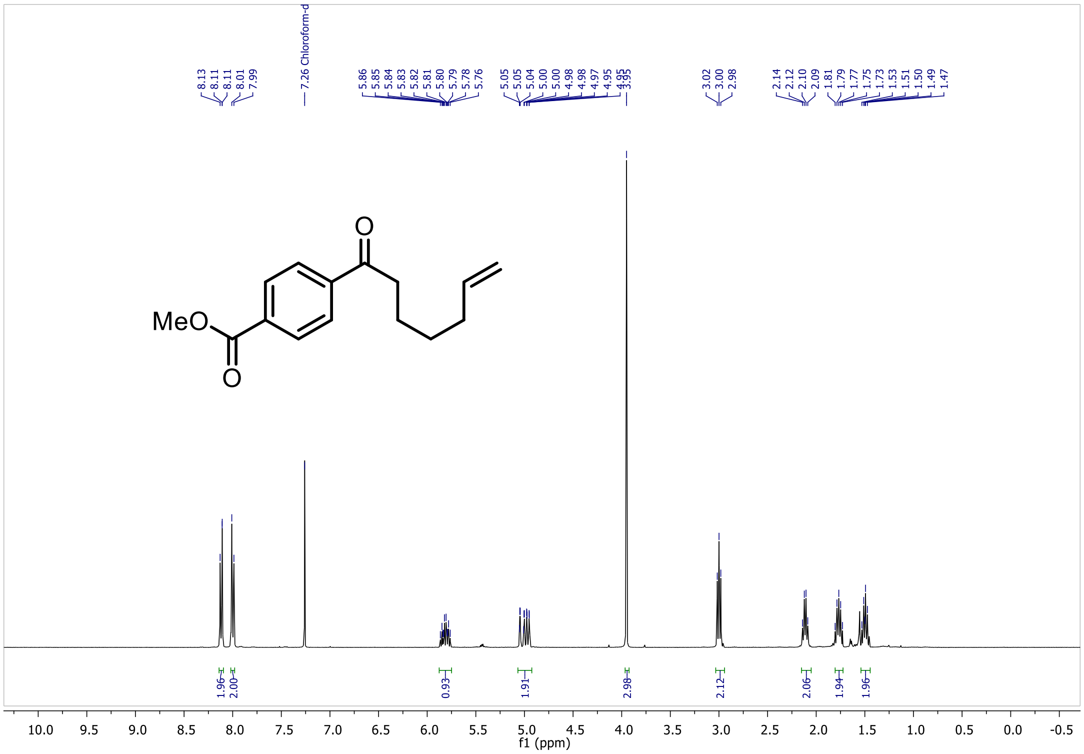

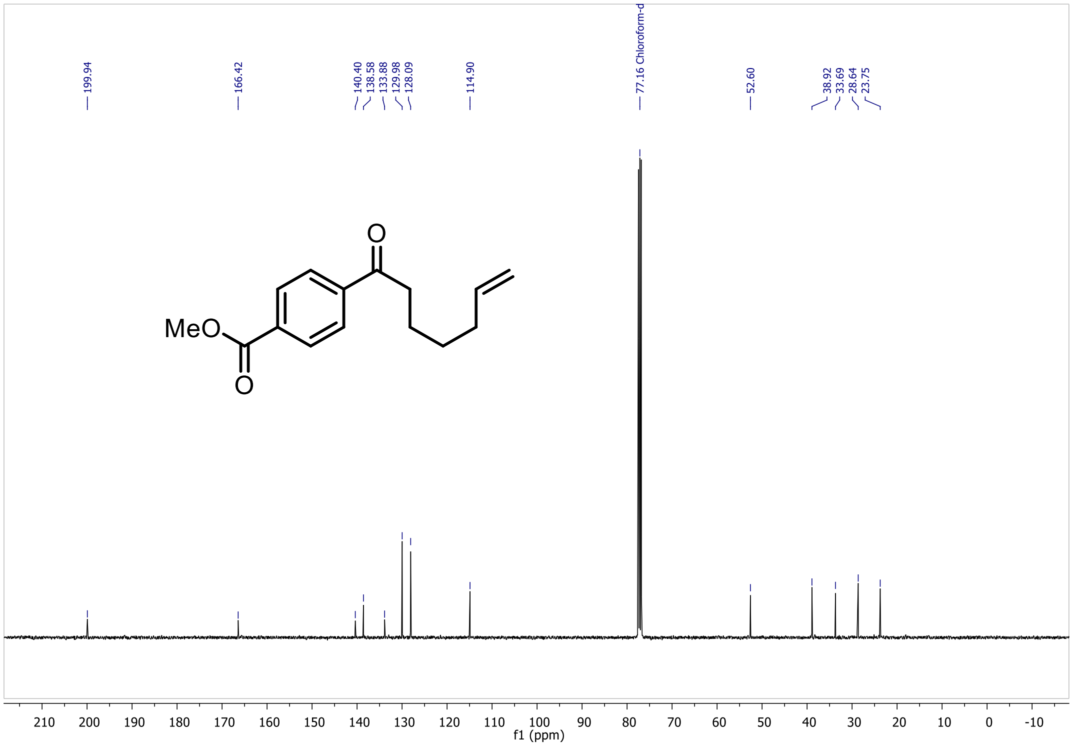

1-(4-(Trifluoromethoxy)phenyl)hept-6-en-1-one (4m)

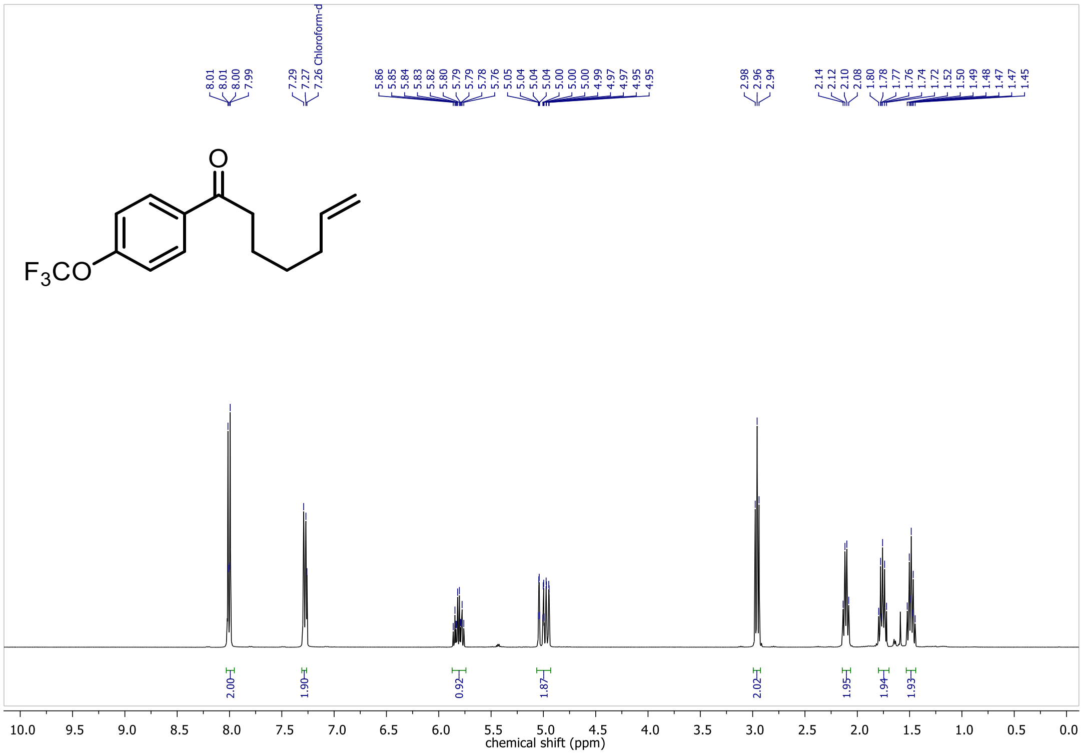

1-(Thiophen-2-yl)hept-6-en-1-one (4n)

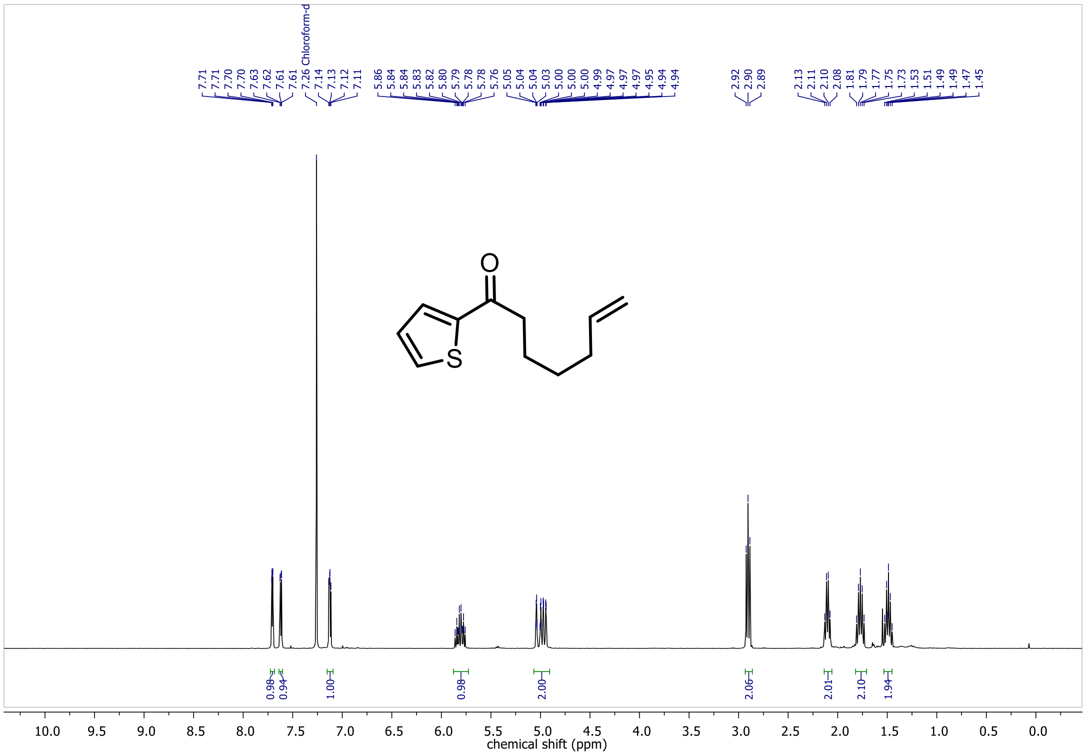

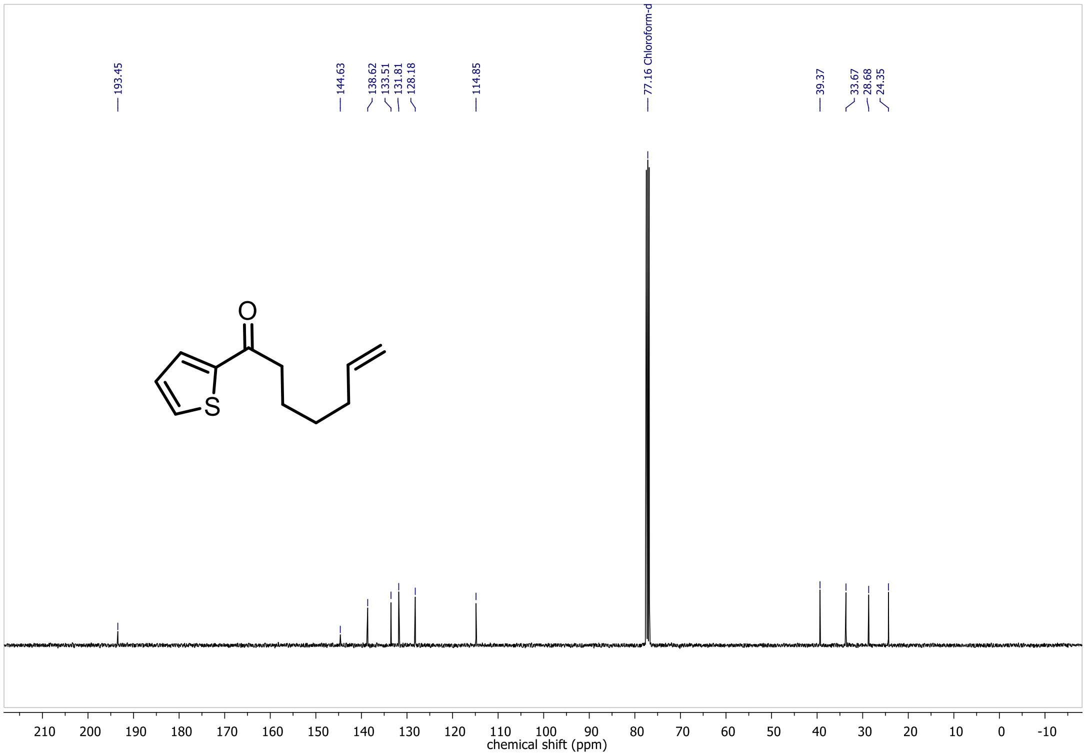

1-(Furan-2-yl)hept-6-en-1-one (4o)

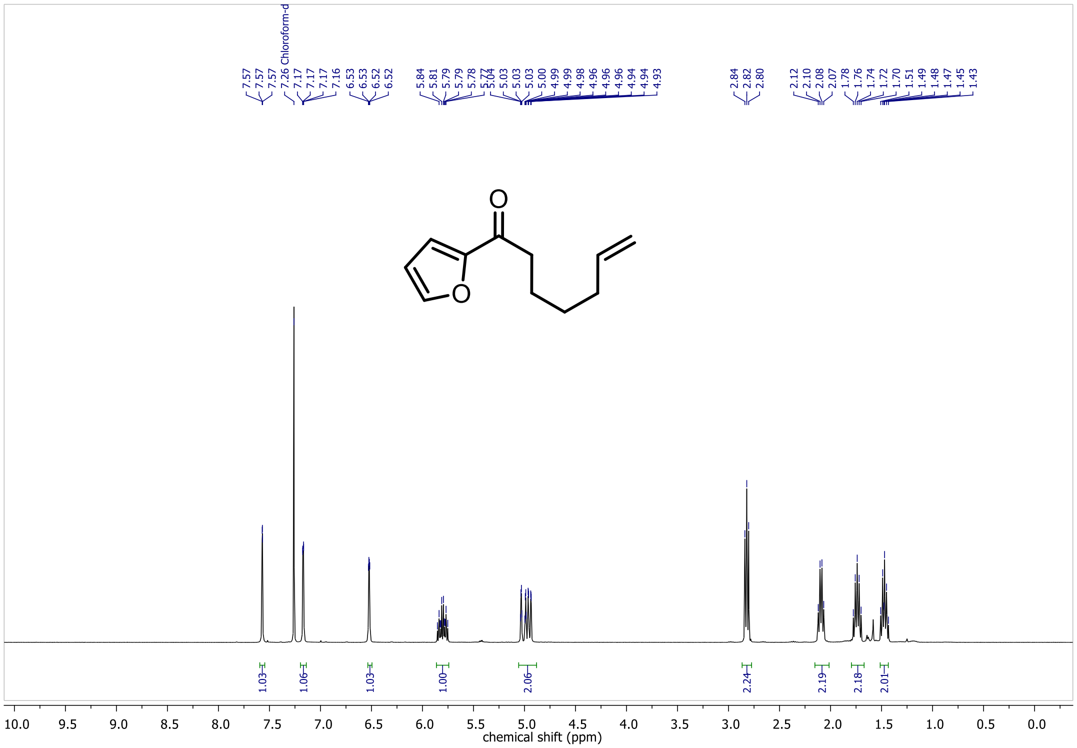

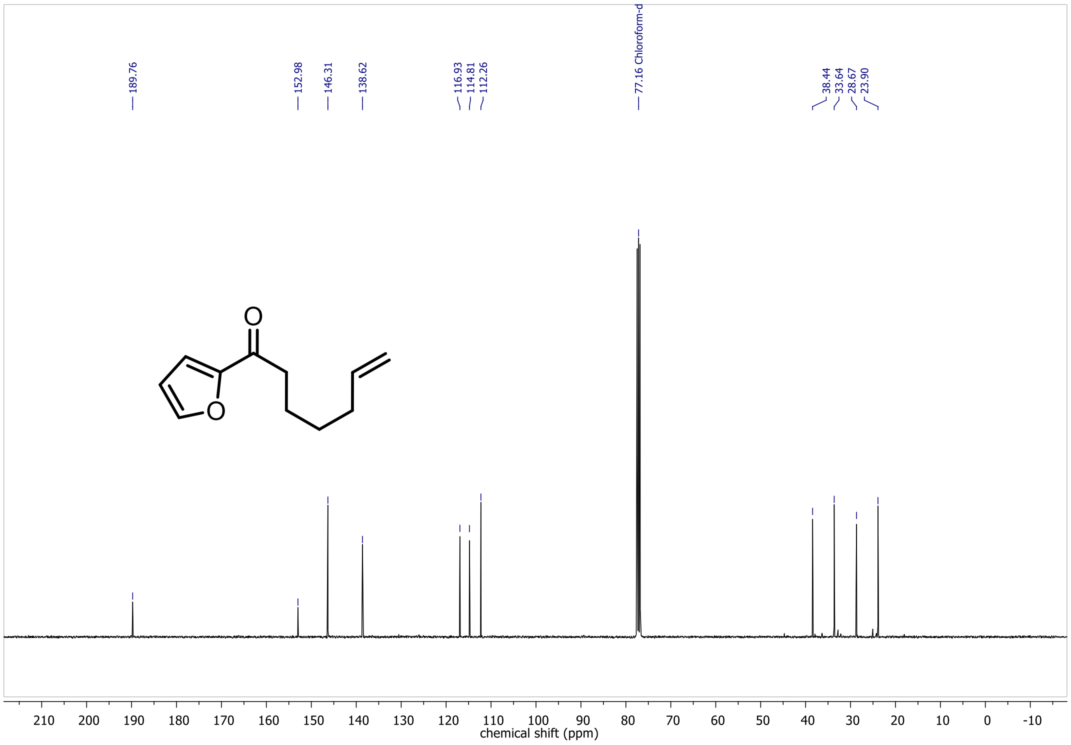

2,2-Dimethylnon-8-en-3-one (4p)

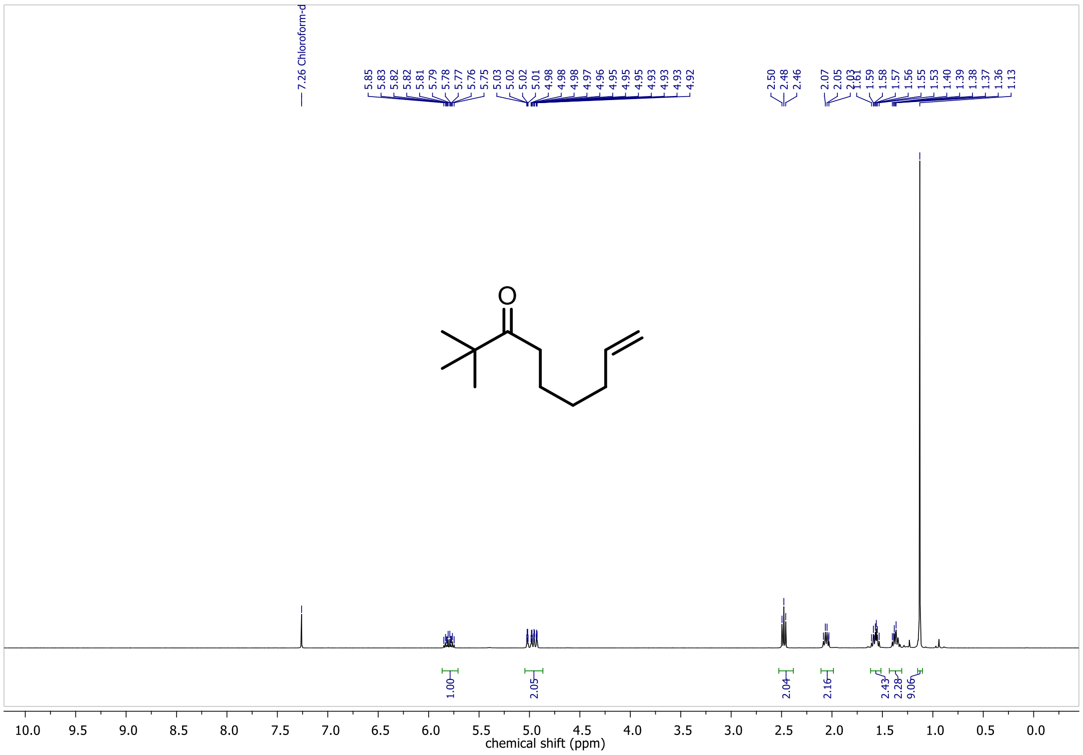

1-(Adamantan-1-yl)hept-6-en-1-one (4q)

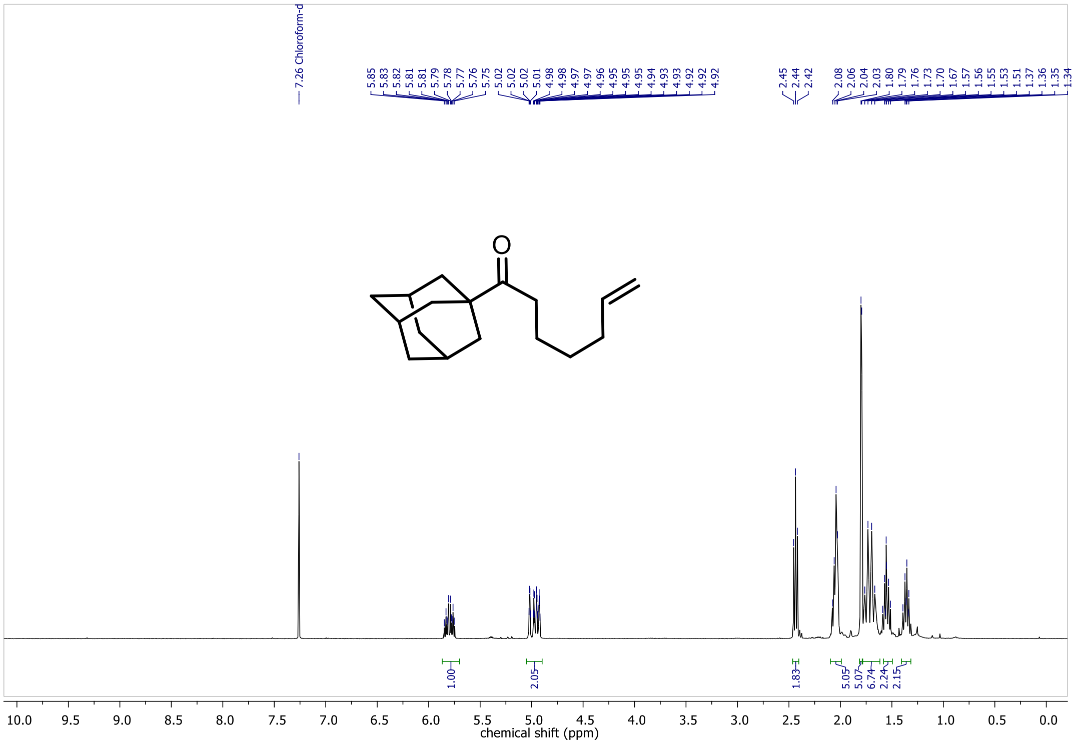

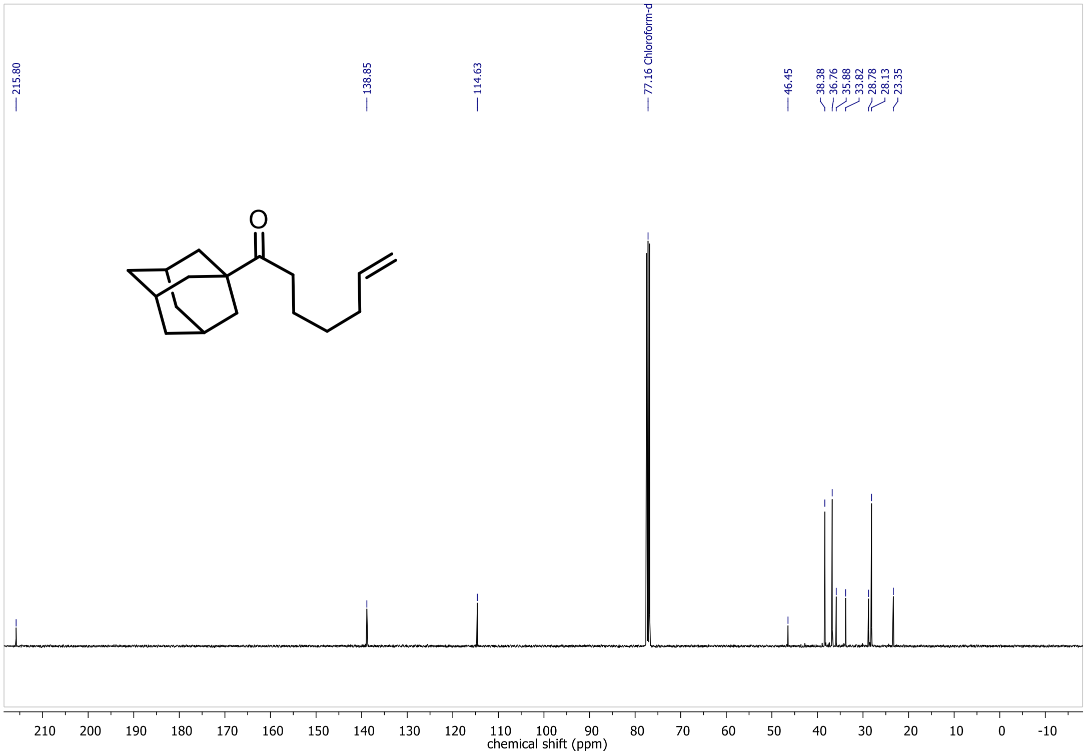

1-cyclohexylhept-6-en-1-one (4r)

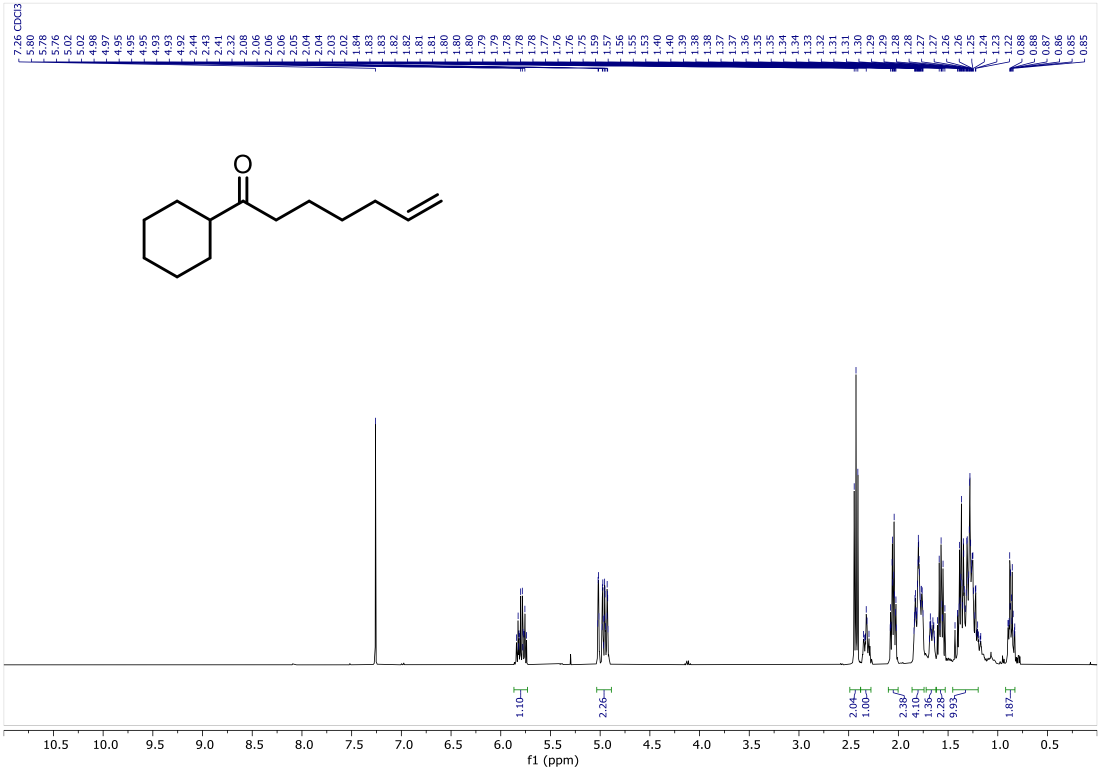

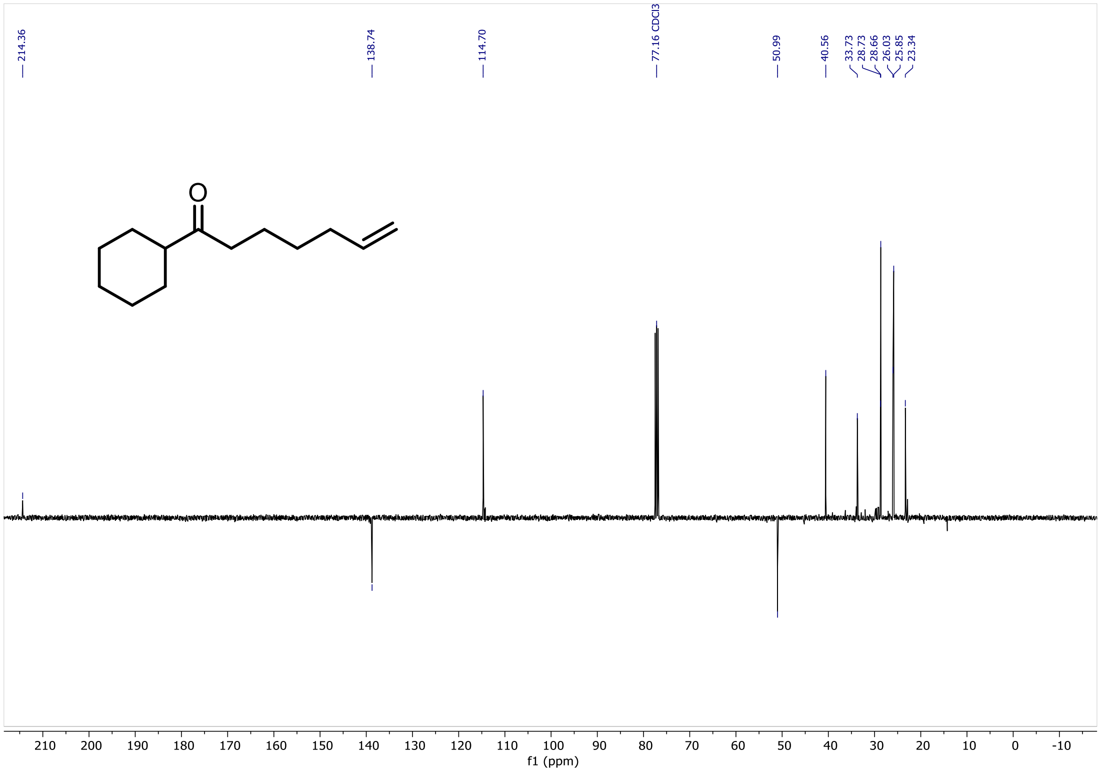

6-Methyl-1-phenylhept-6-en-1-one (4s)

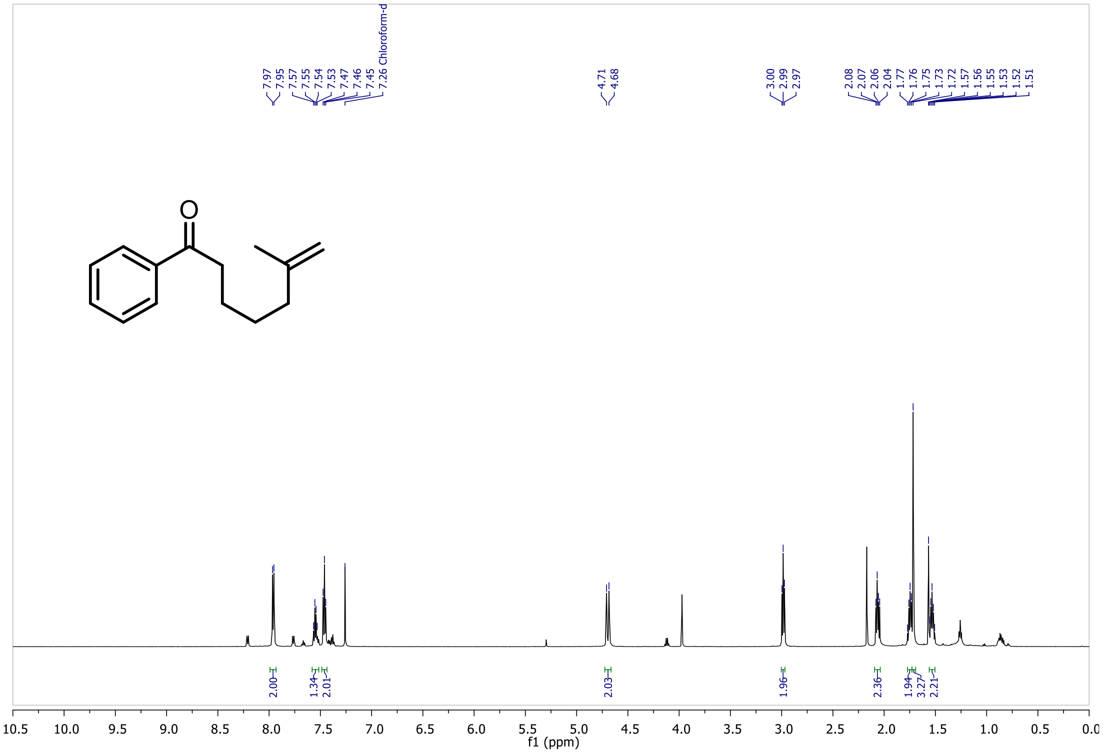

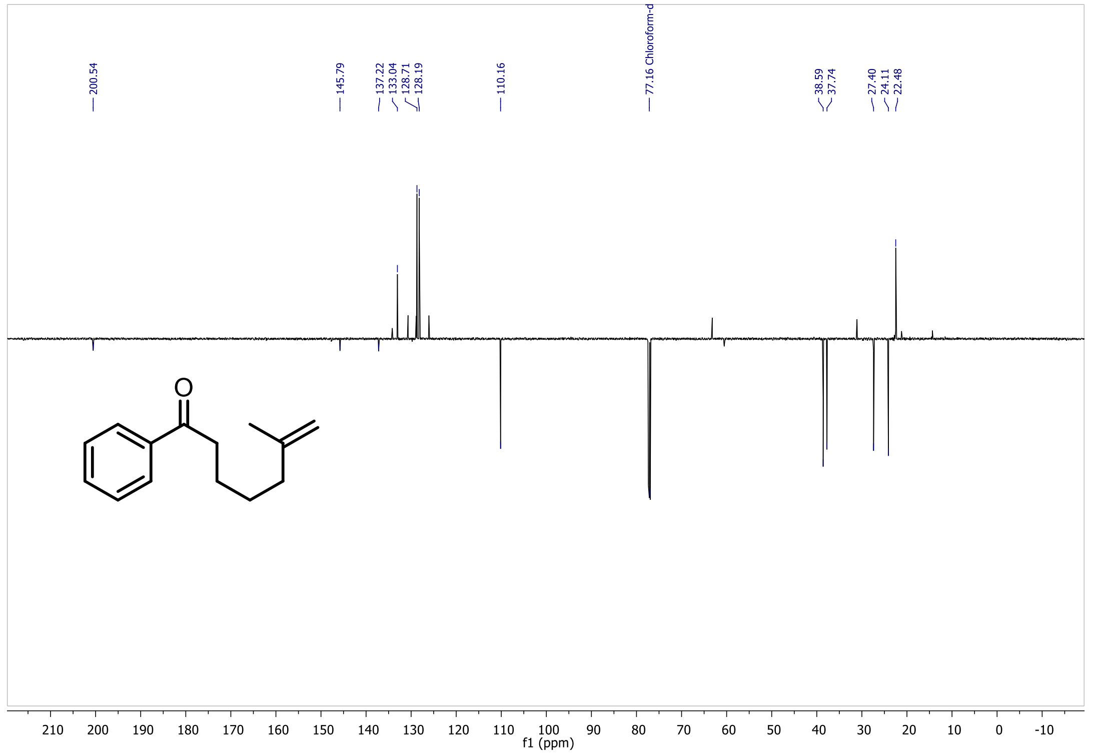

1,6-Diphenylhept-6-en-1-one (4t)

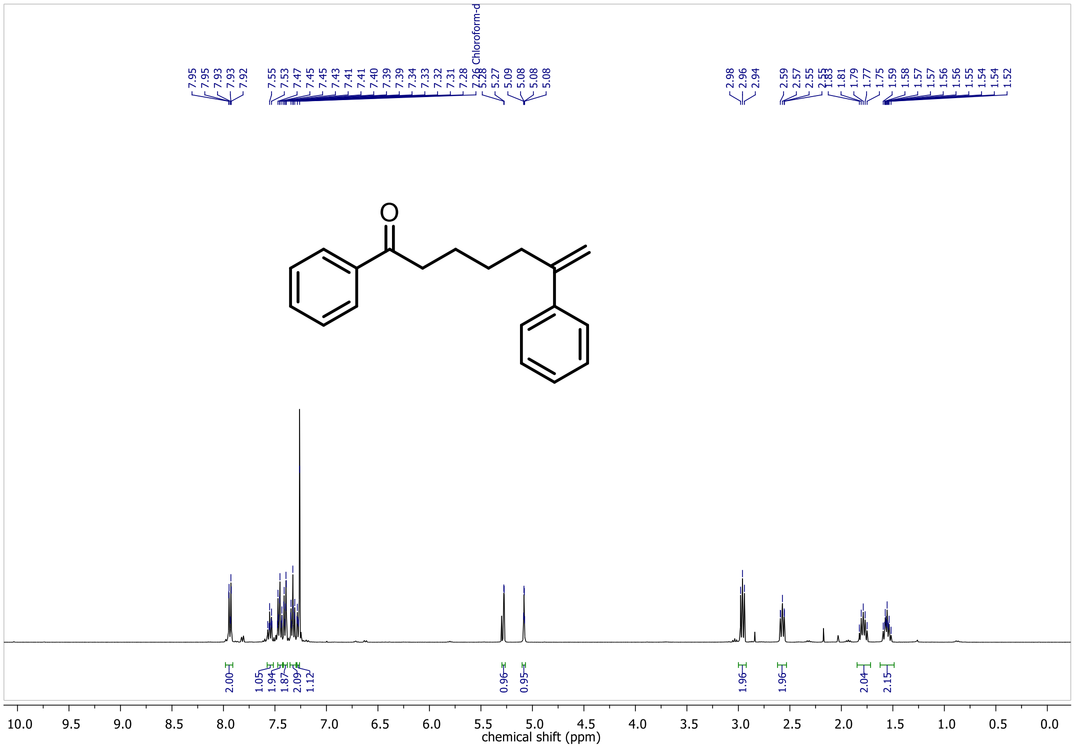

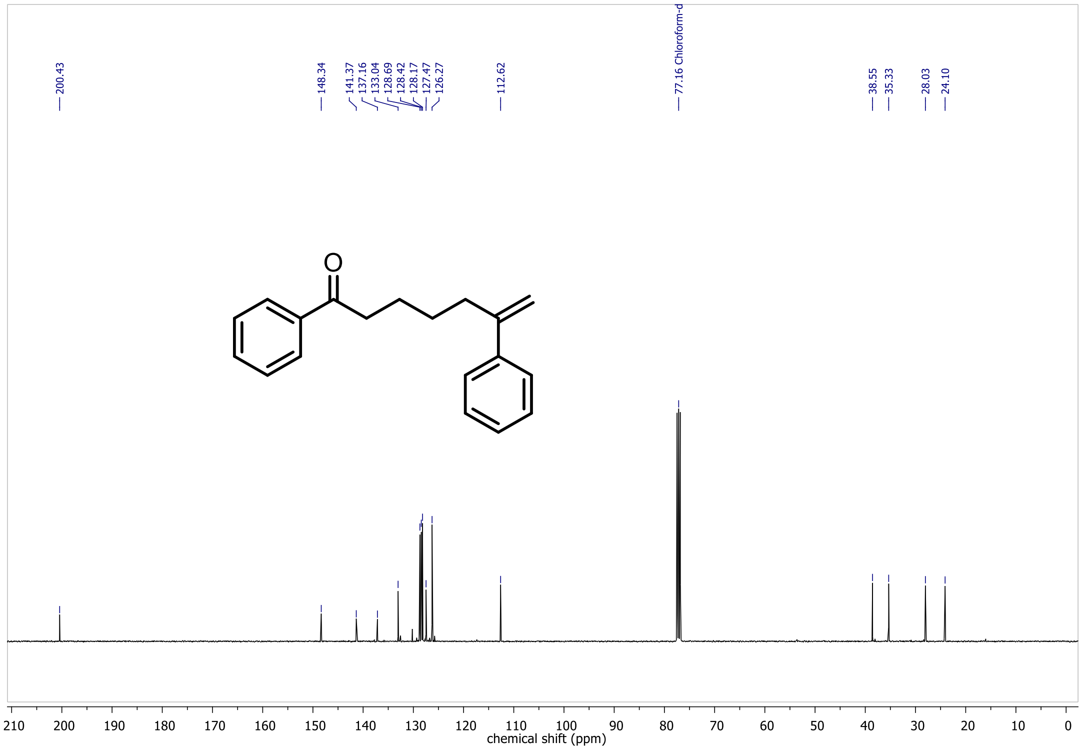

6-(Naphthalen-2-yl)-1-phenylhept-6-en-1-one (4u)

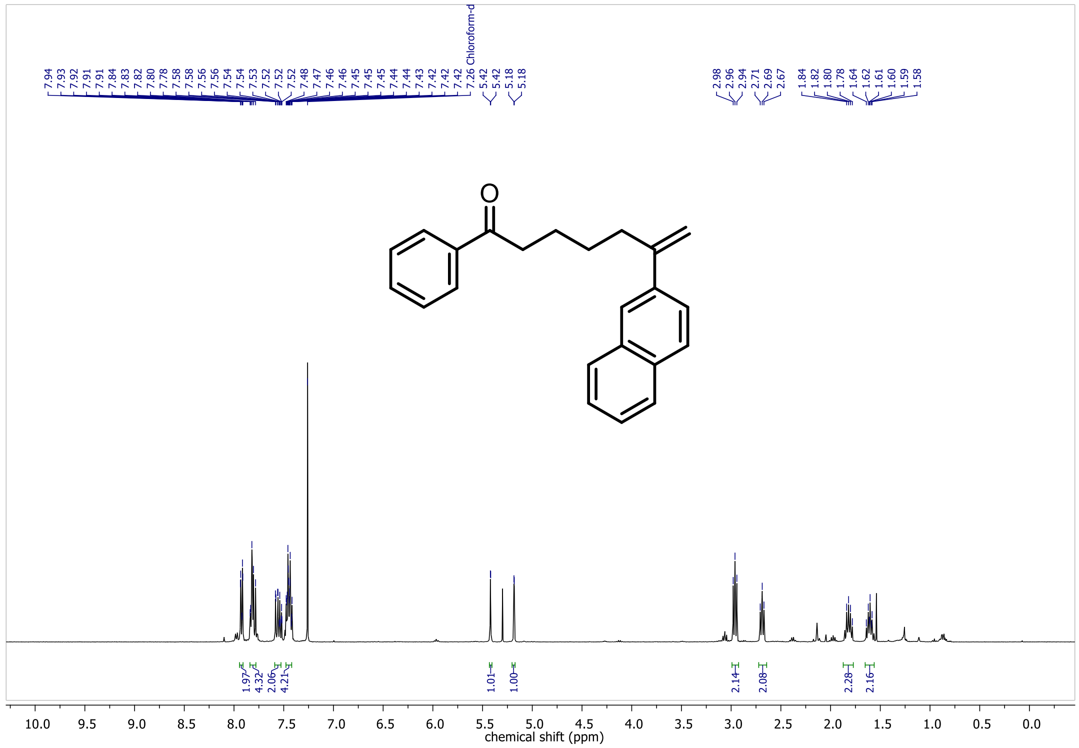

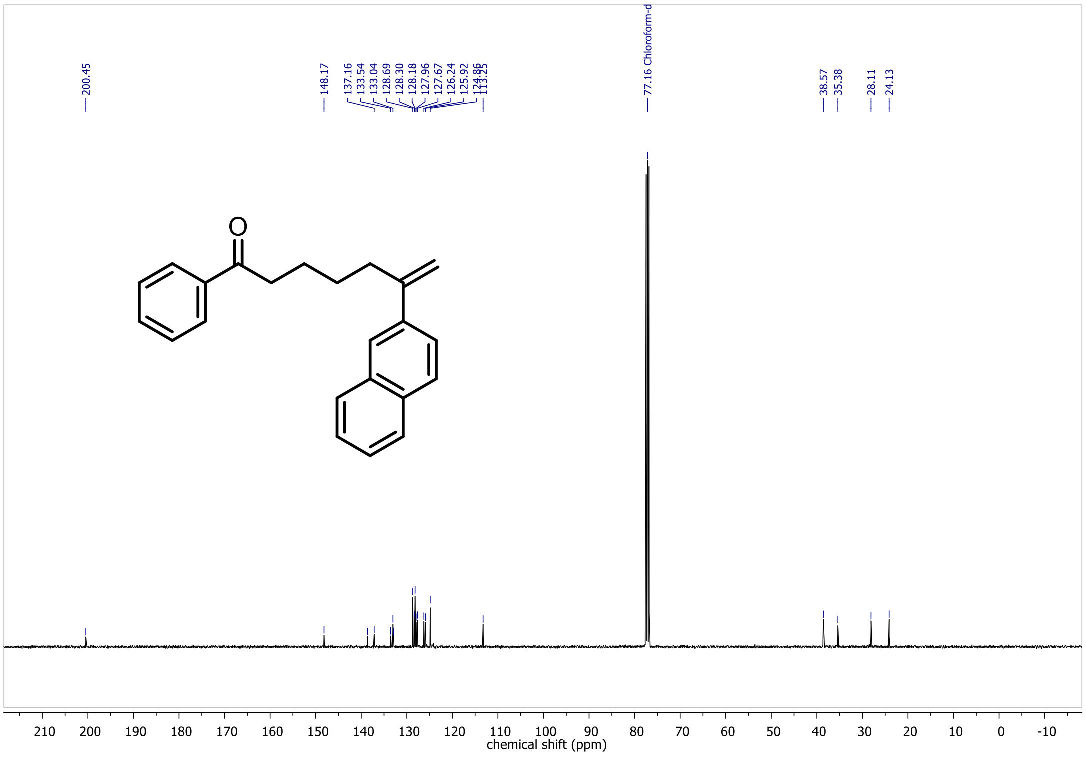

6-(Dibenzo[*b,d*]furan-1-yl)-1-phenylhept-6-en-1-one (4v)

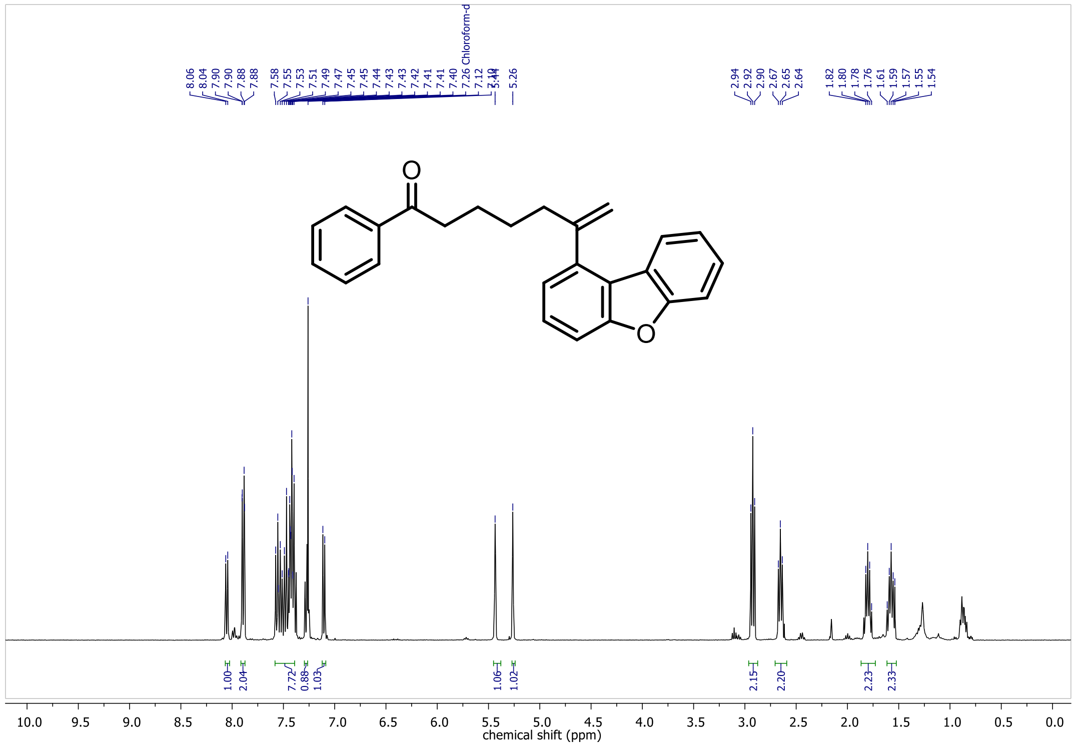

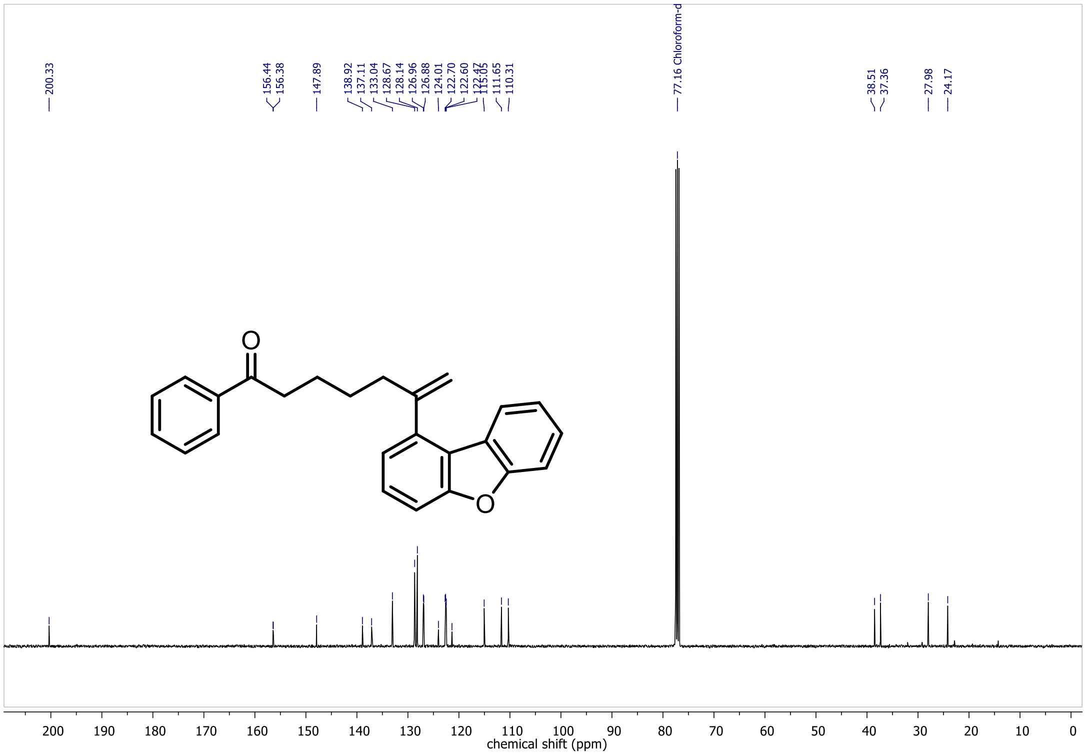

(*Z*)-Trimethyl((1-phenylhepta-1,6-dien-1-yl)oxy)silane (1a)

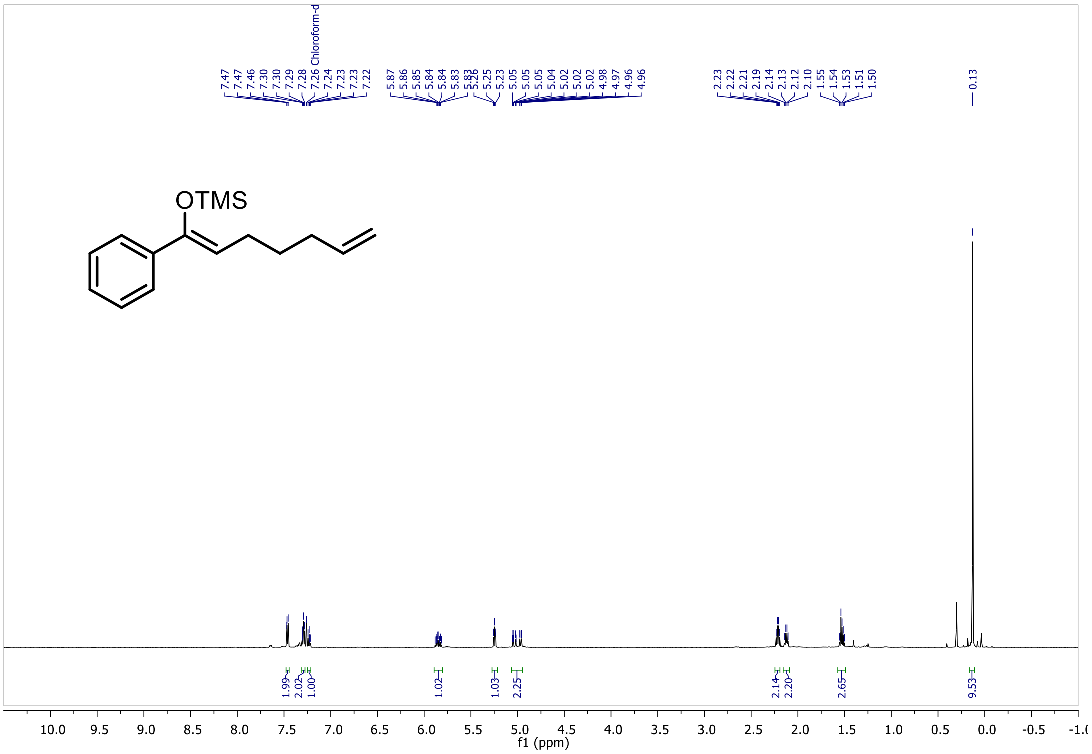

(*Z*)-((1-(3-Fluorophenyl)hepta-1,6-dien-1-yl)oxy)trimethylsilane (1b)

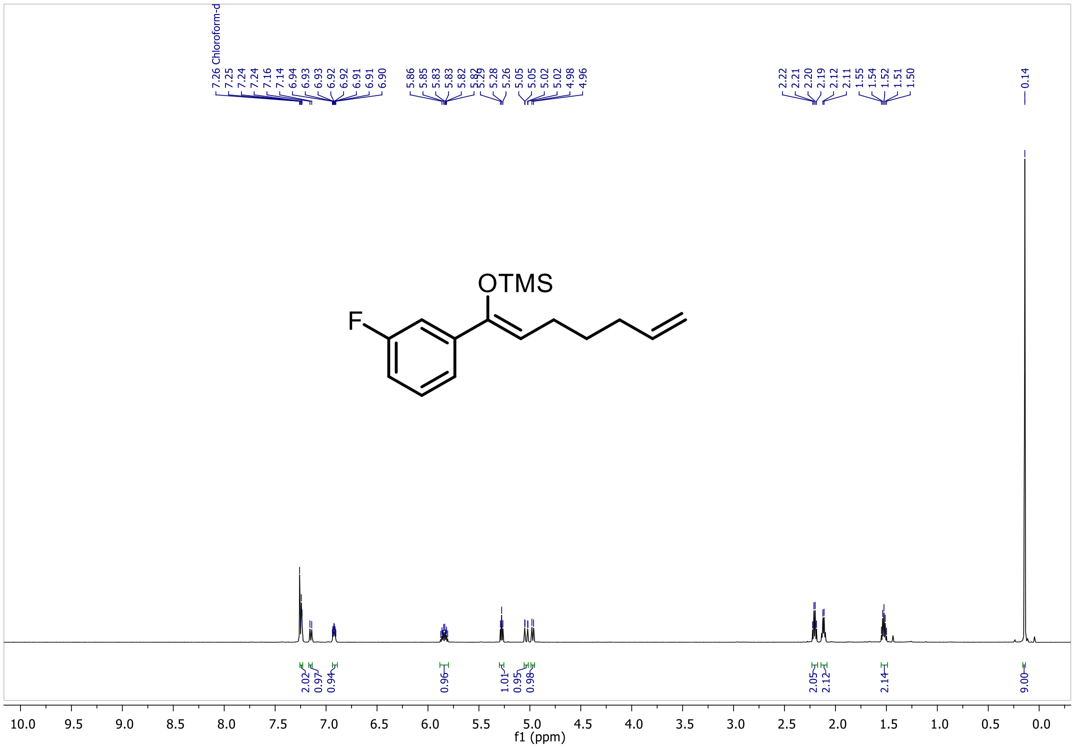

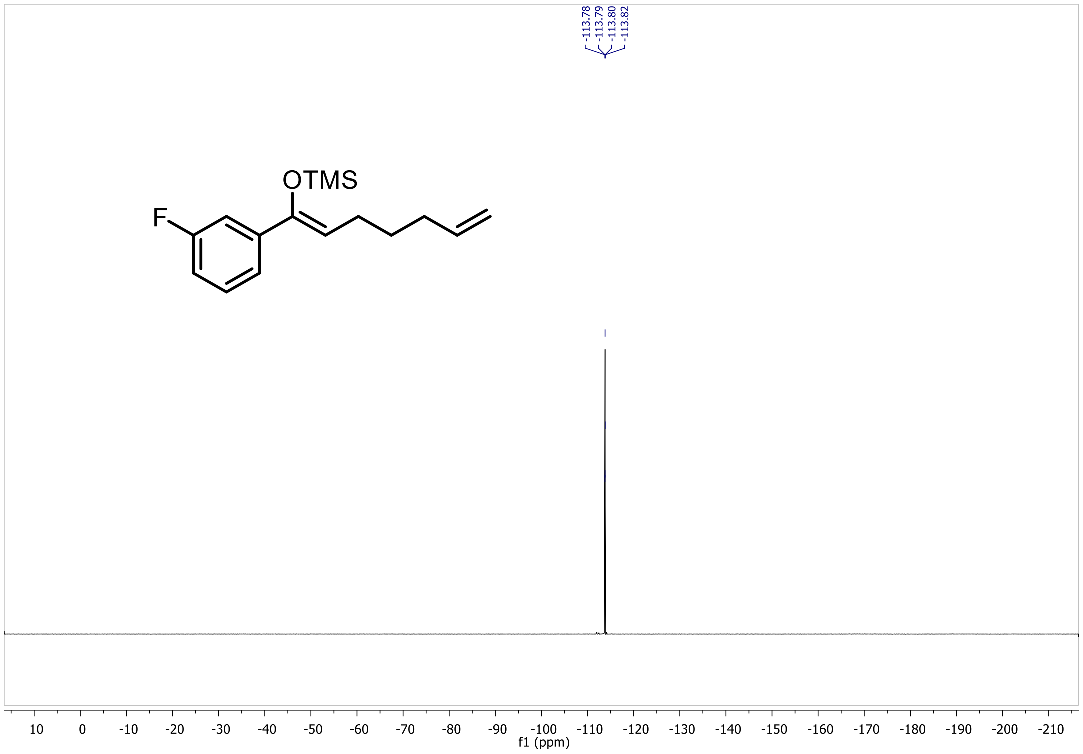

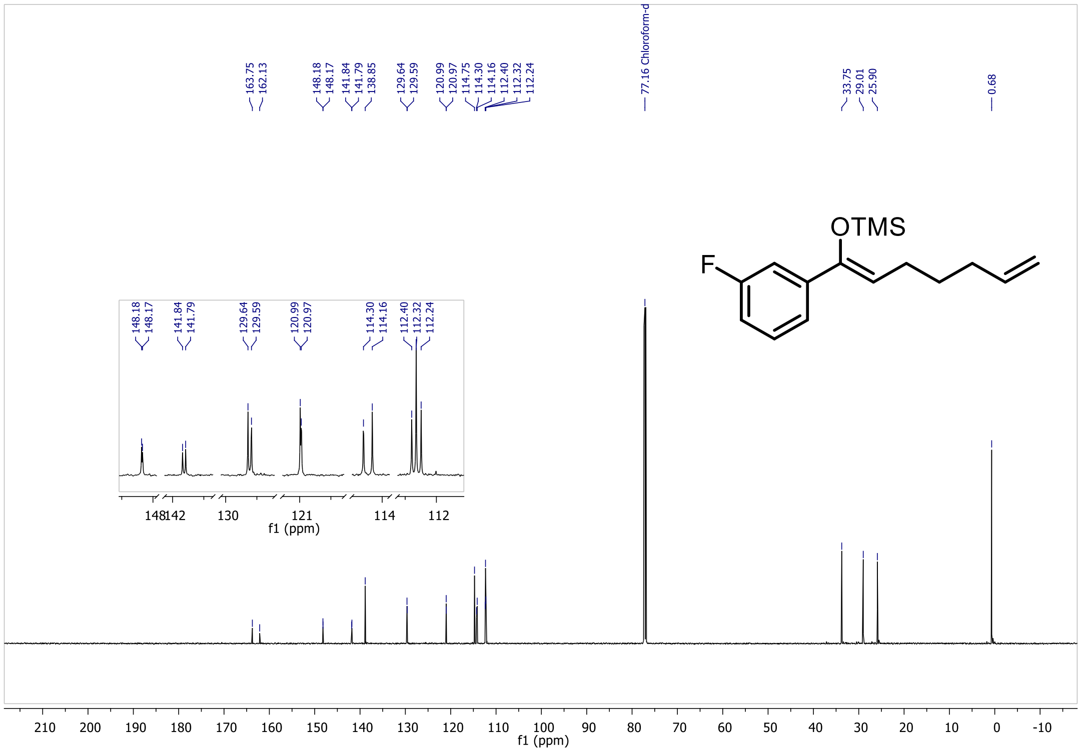

163.75
162.13
148.18
148.17
141.84
141.79
138.85
129.64
129.59
120.99
120.97
114.75
114.30
114.16
112.40
112.32
112.24
77.16 Chloroform-d
33.75
29.01
25.90
0.68
OTMS
F
f1 (ppm)

(*Z*)-((1-(4-Fluorophenyl)hepta-1,6-dien-1-yl)oxy)trimethylsilane (1c)

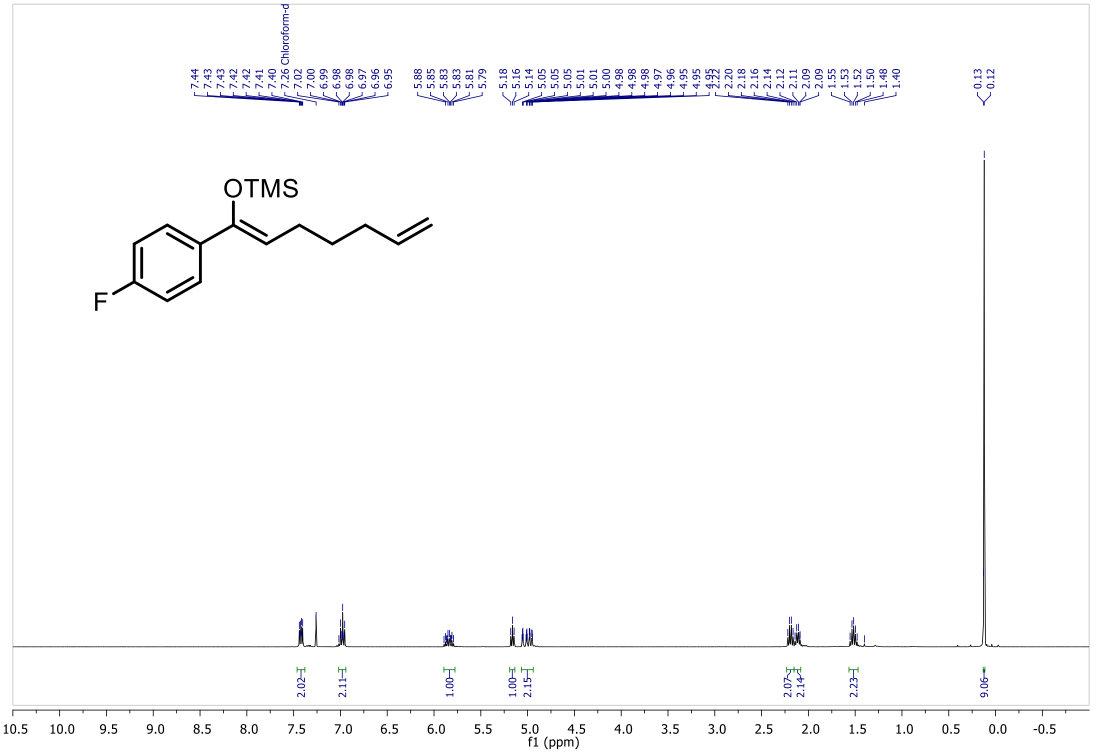

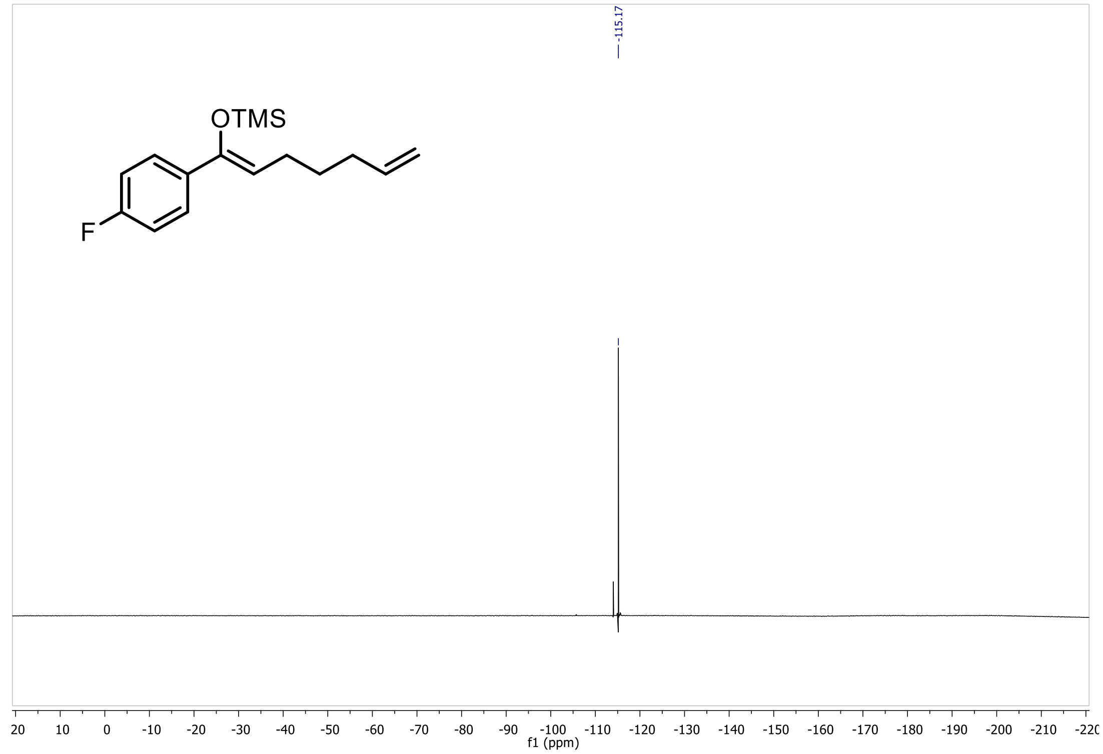

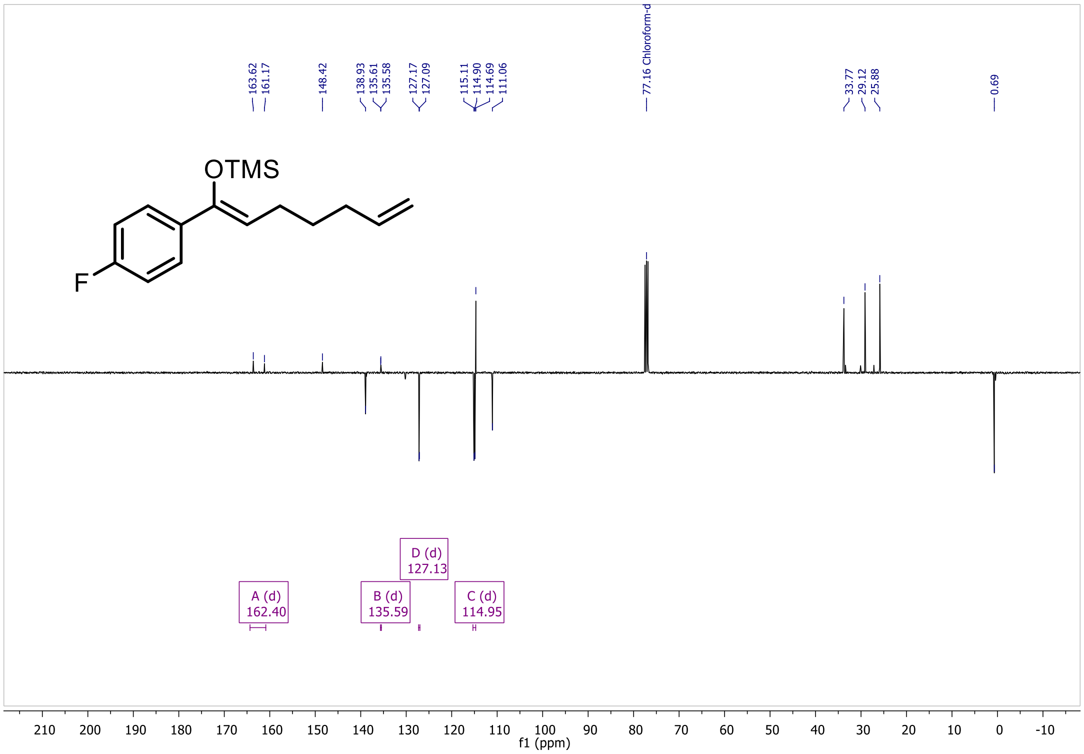

163.62
161.17
148.42
138.93
135.61
135.58
127.17
127.09
115.11
114.90
114.69
111.06
77.16 Chloroform-d
33.77
29.12
25.88
0.69
OTMS
F
A (d)
162.40
B (d)
135.59
D (d)
127.13
C (d)
114.95
210 200 190 180 170 160 150 140 130 120 110 100 90 80 70 60 50 40 30 20 10 0 -10
f1 (ppm)

(*Z*)-((1-(3,5-Bis(trifluoromethyl)phenyl)hepta-1,6-dien-1-yl)oxy)trimethylsilane (1d)

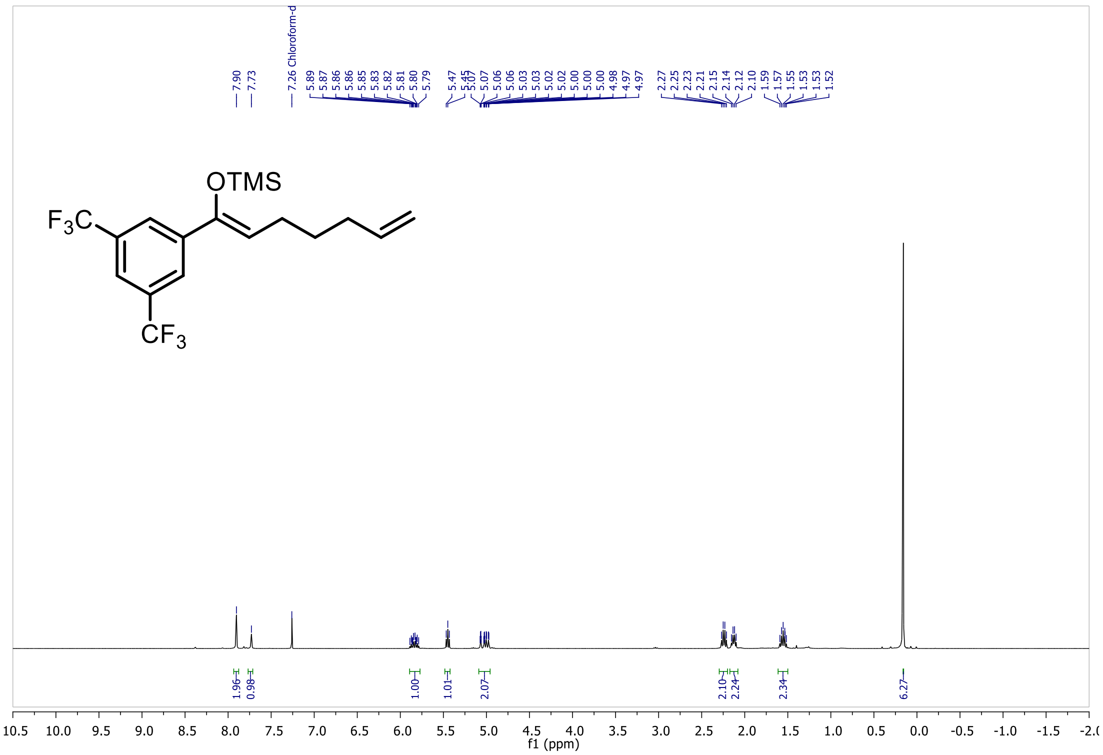

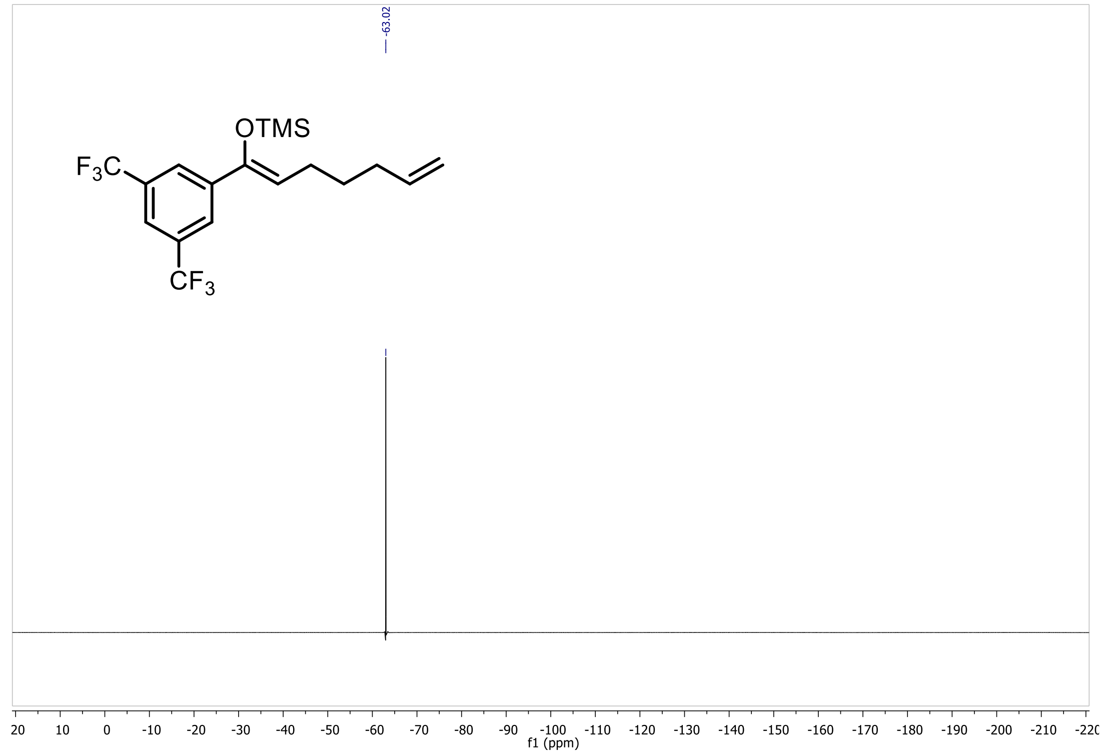

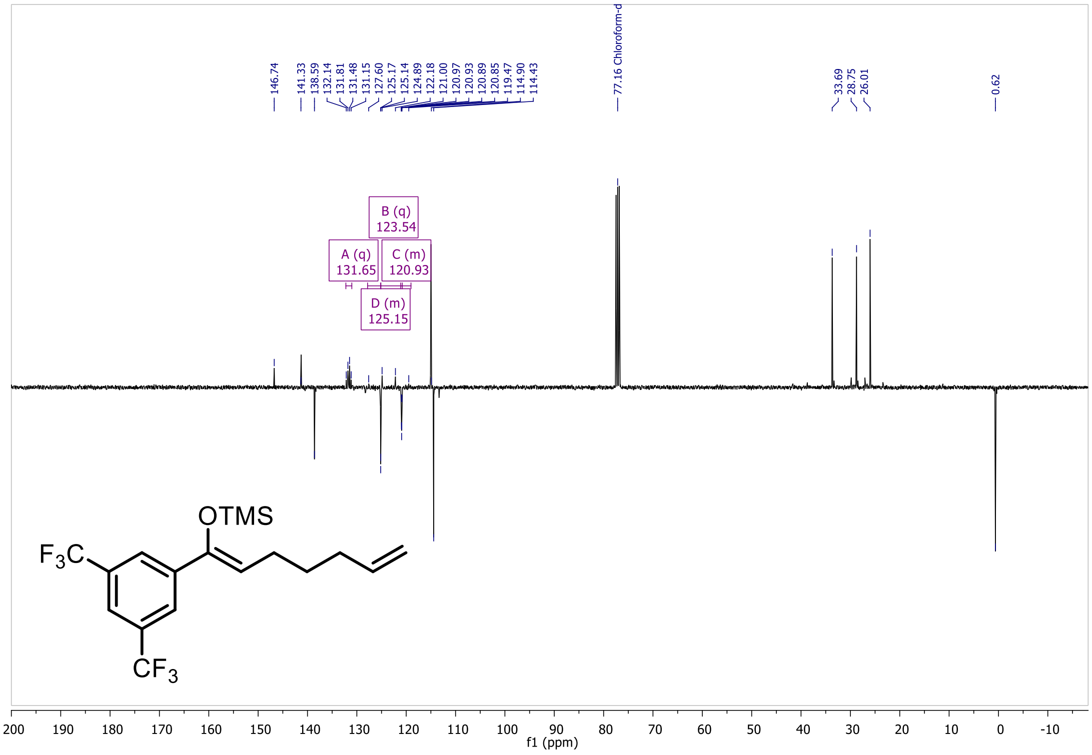
146.74
141.33
138.59
132.14
131.81
131.48
131.15
127.60
125.17
125.14
124.89
122.18
121.00
120.97
120.93
120.89
120.85
119.47
114.90
114.43
77.16 Chloroform-d
33.69
28.75
26.01
0.62
B (q)
123.54
A (q)
131.65
C (m)
120.93
D (m)
125.15
OTMS
F3C
CF3
200 190 180 170 160 150 140 130 120 110 100 90 80 70 60 50 40 30 20 10 0 -10
f1 (ppm)

(*Z*)-((1-(3,5-Difluorophenyl)hepta-1,6-dien-1-yl)oxy)trimethylsilane (1e)

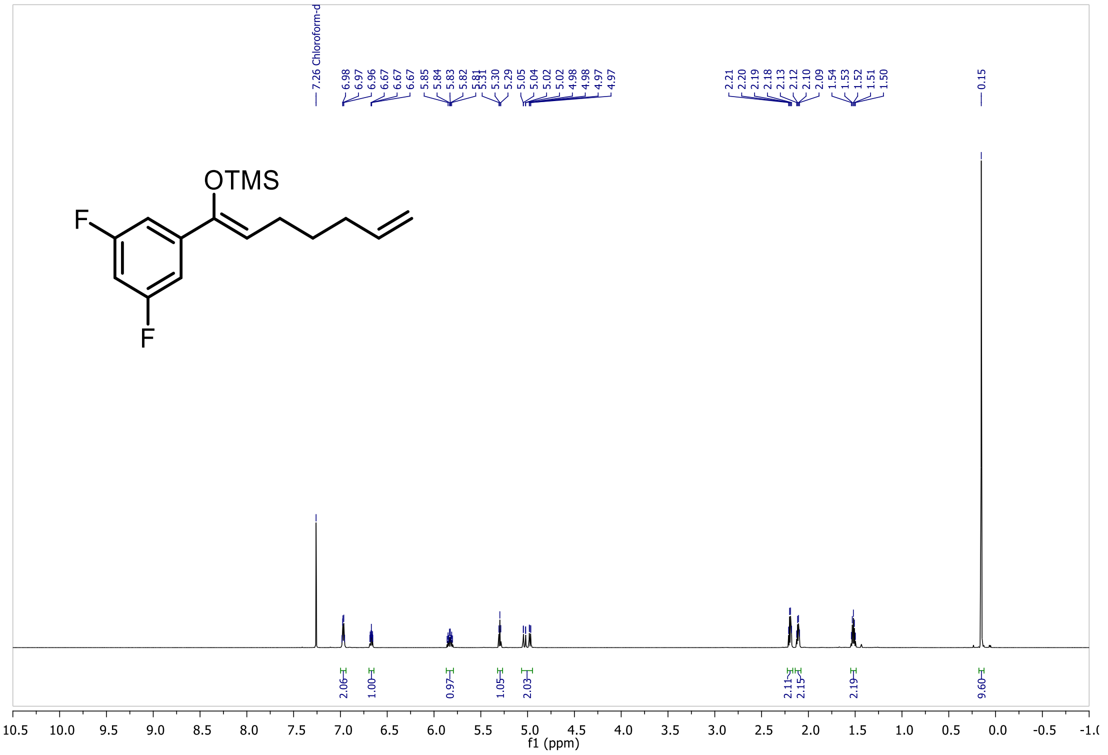

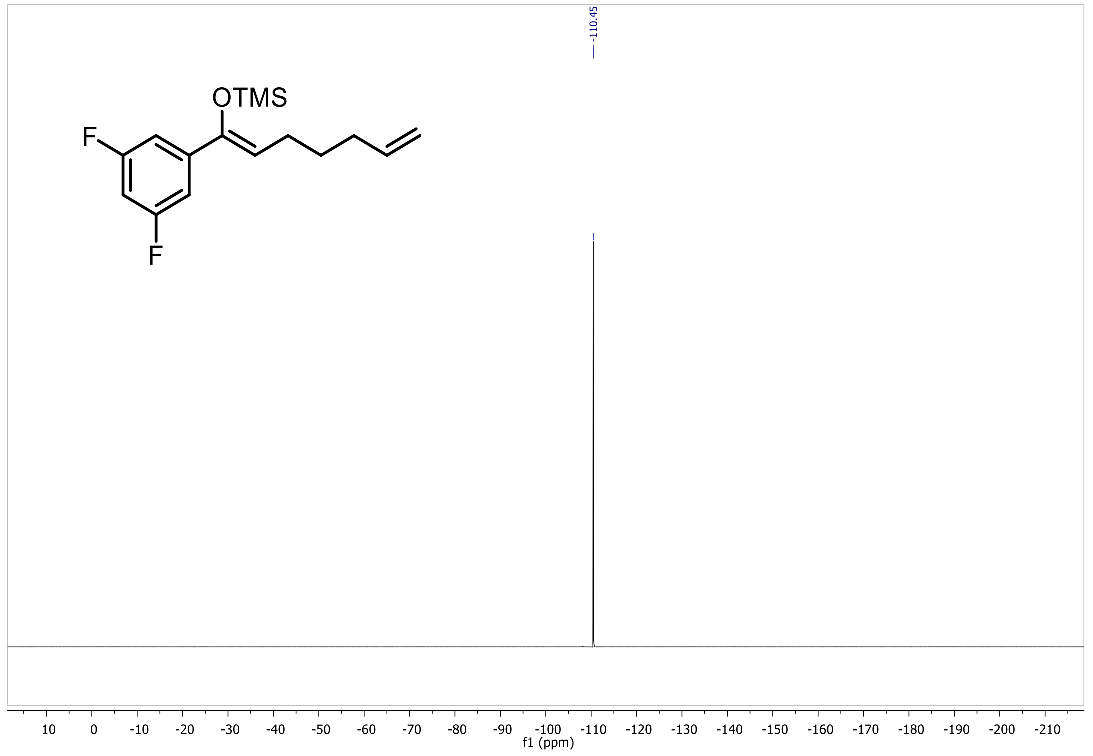

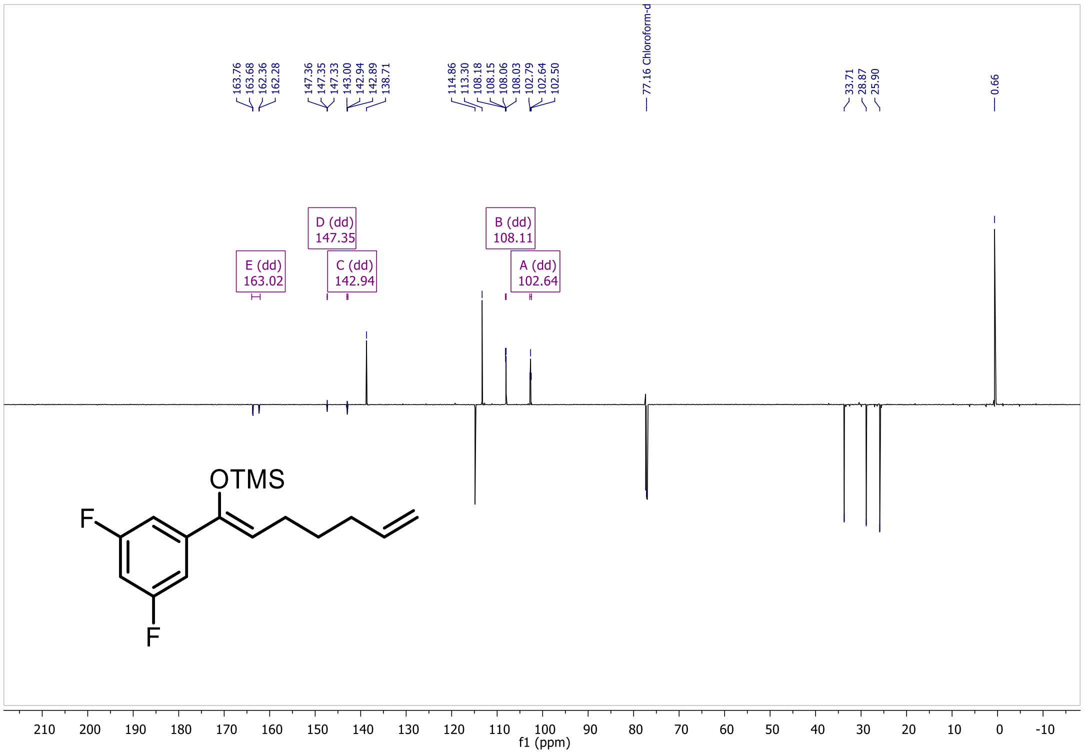
163.76
163.68
162.36
162.28
147.36
147.35
147.33
143.00
142.94
142.89
138.71
114.86
113.30
108.18
108.15
108.06
108.03
102.79
102.64
102.50
77.16 Chloroform-d
33.71
28.87
25.90
0.66
D (dd) 147.35
B (dd) 108.11
E (dd) 163.02
C (dd) 142.94
A (dd) 102.64
OTMS
F
F
210 200 190 180 170 160 150 140 130 120 110 100 90 80 70 60 50 40 30 20 10 0 -10
f1 (ppm)

(*Z*)-Trimethyl((1-(4-(trifluoromethyl)phenyl)hepta-1,6-dien-1-yl)oxy)silane (1f)

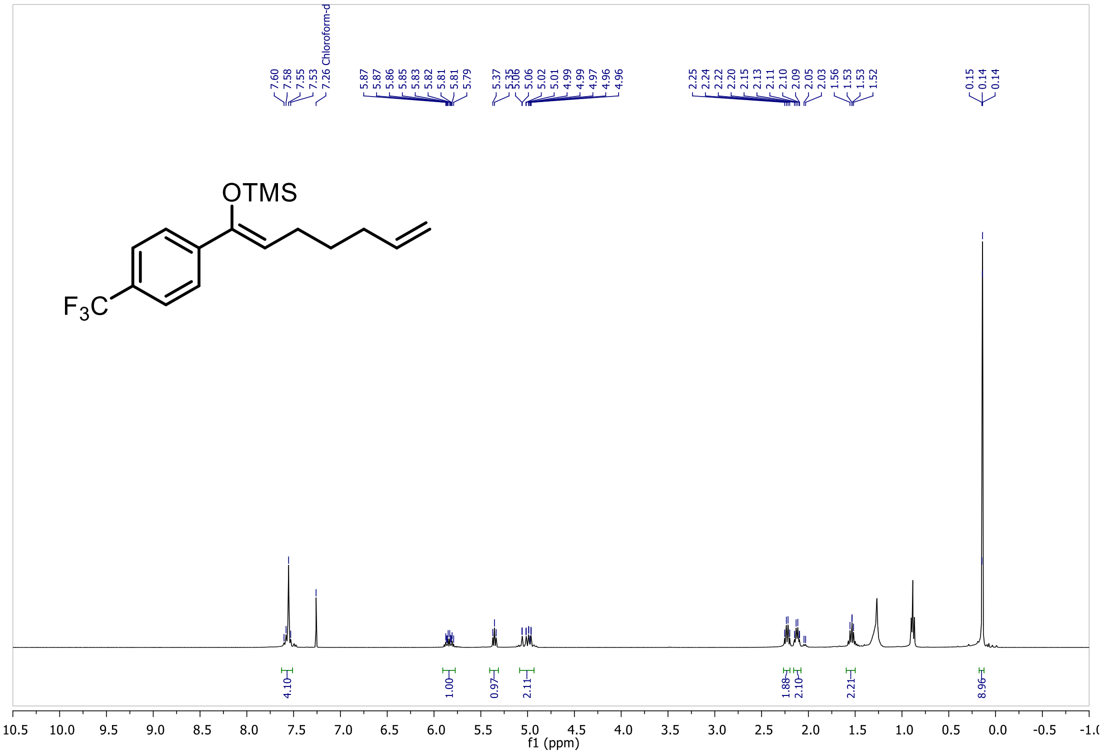

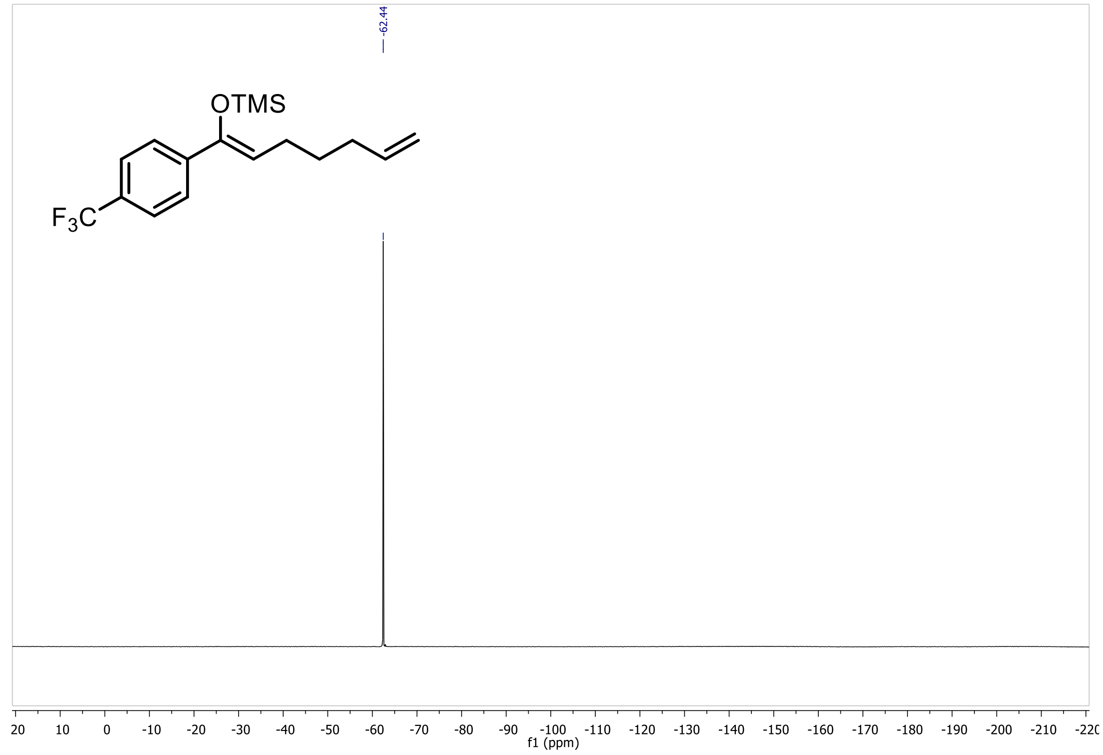

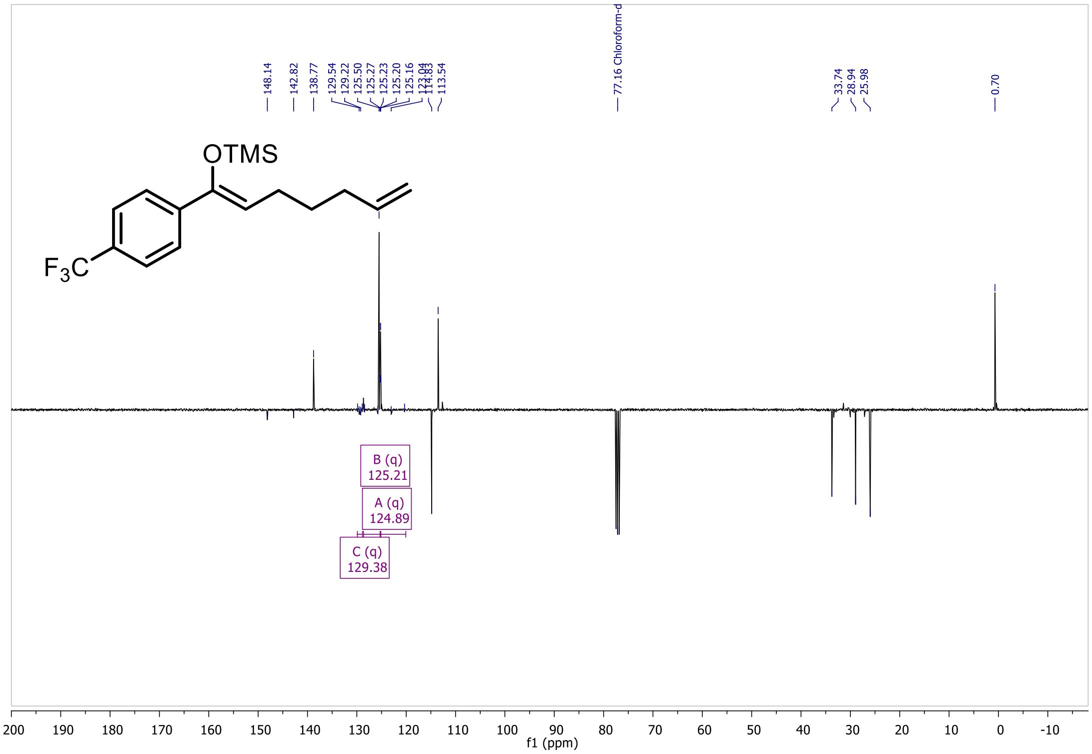

OTMS
F3C
148.14
142.82
138.77
129.54
129.22
125.50
125.27
125.23
125.20
125.16
123.04
114.83
113.54
77.16 Chloroform-d
33.74
28.94
25.98
0.70
B (q)
125.21
A (q)
124.89
C (q)
129.38
200 190 180 170 160 150 140 130 120 110 100 90 80 70 60 50 40 30 20 10 0 -10
f1 (ppm)

(*Z*)-((1-(3,4-Dichlorophenyl)hepta-1,6-dien-1-yl)oxy)trimethylsilane (1g)

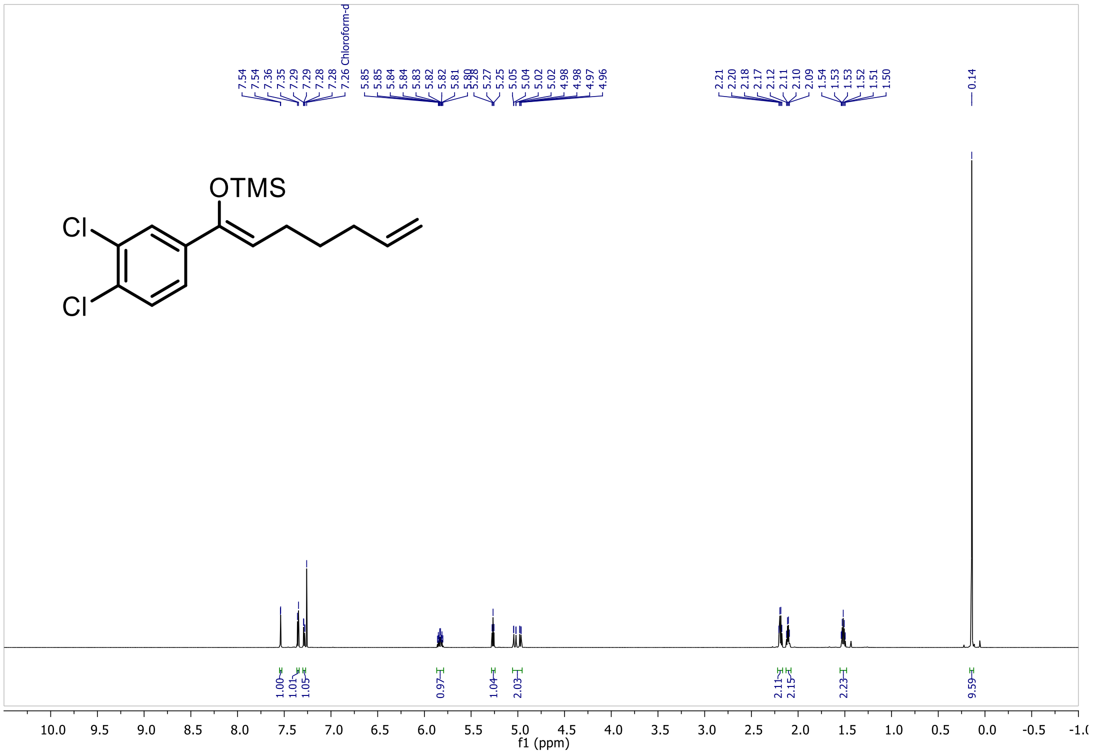

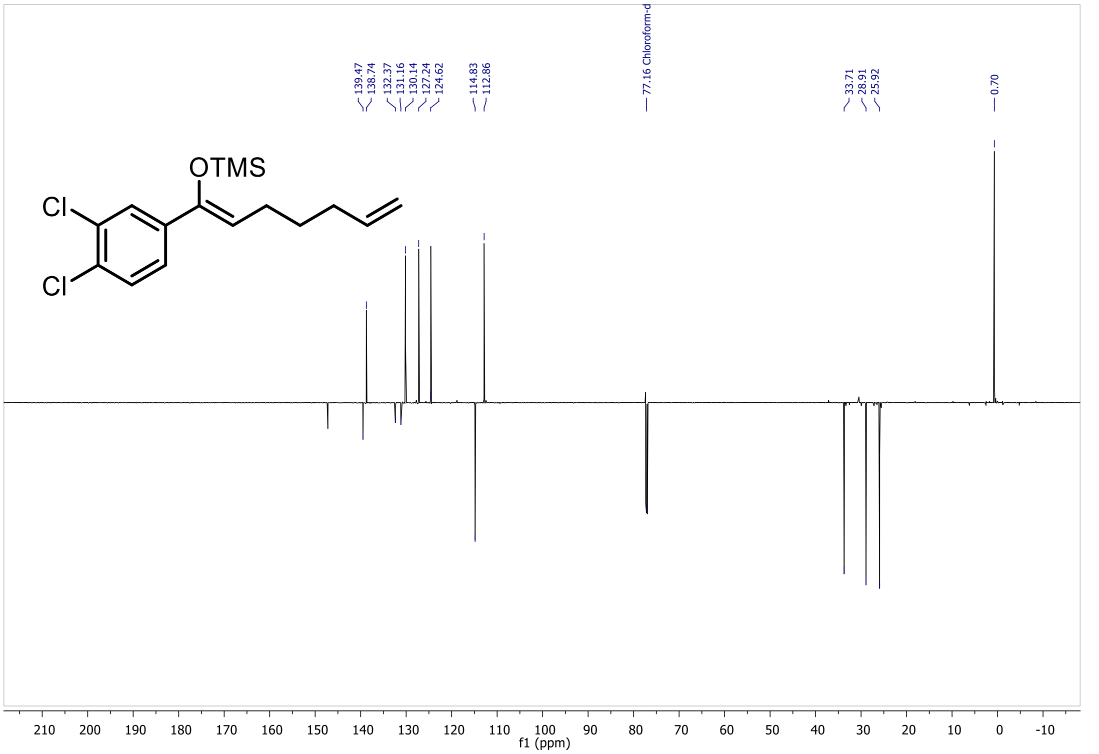

(*Z*)-((1-(3-Chlorophenyl)hepta-1,6-dien-1-yl)oxy)trimethylsilane (1h)

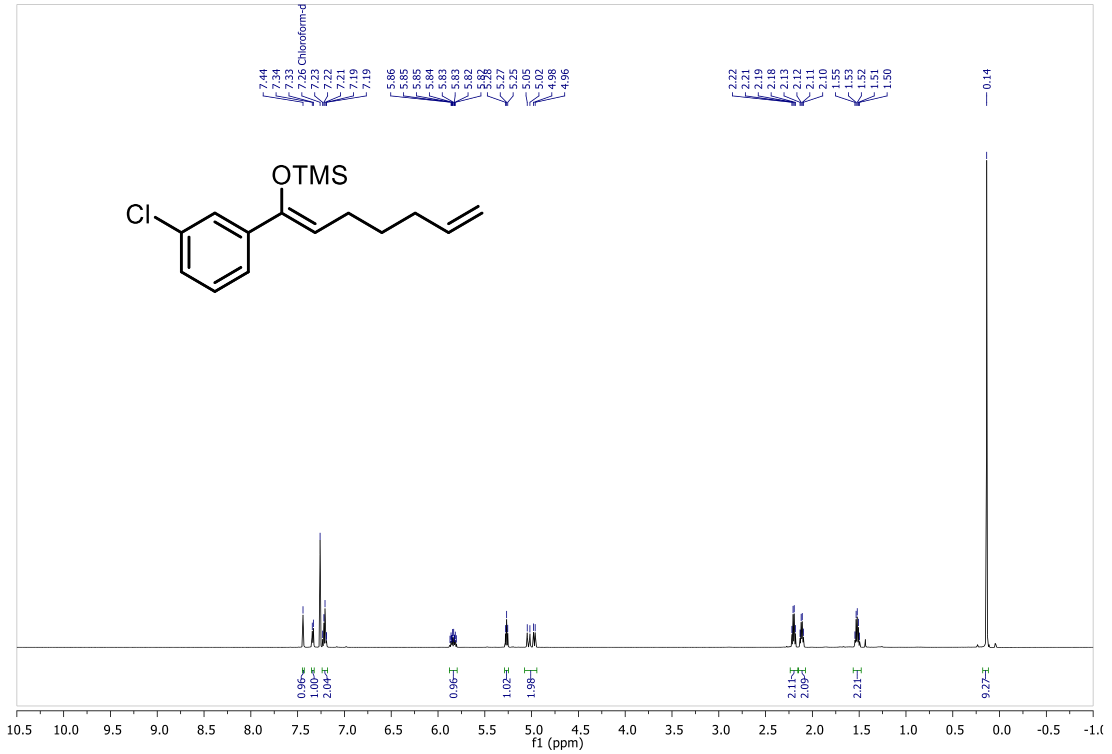

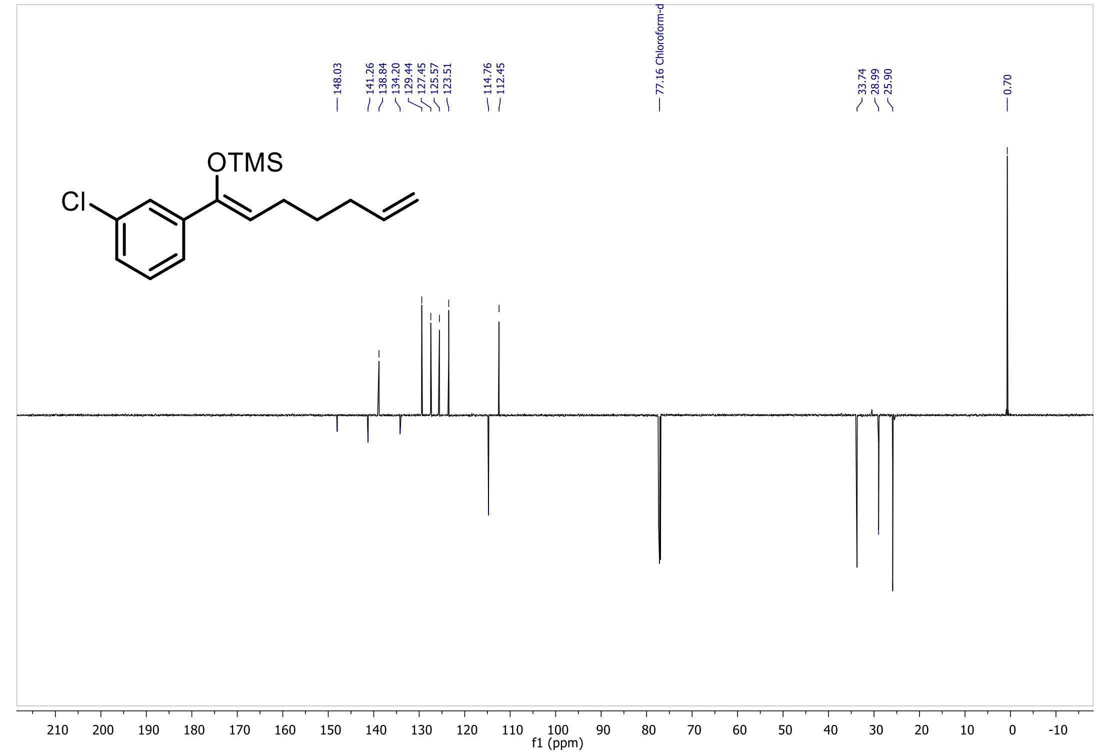

(*Z*)-((1-(2-Bromophenyl)hepta-1,6-dien-1-yl)oxy)trimethylsilane (1i)

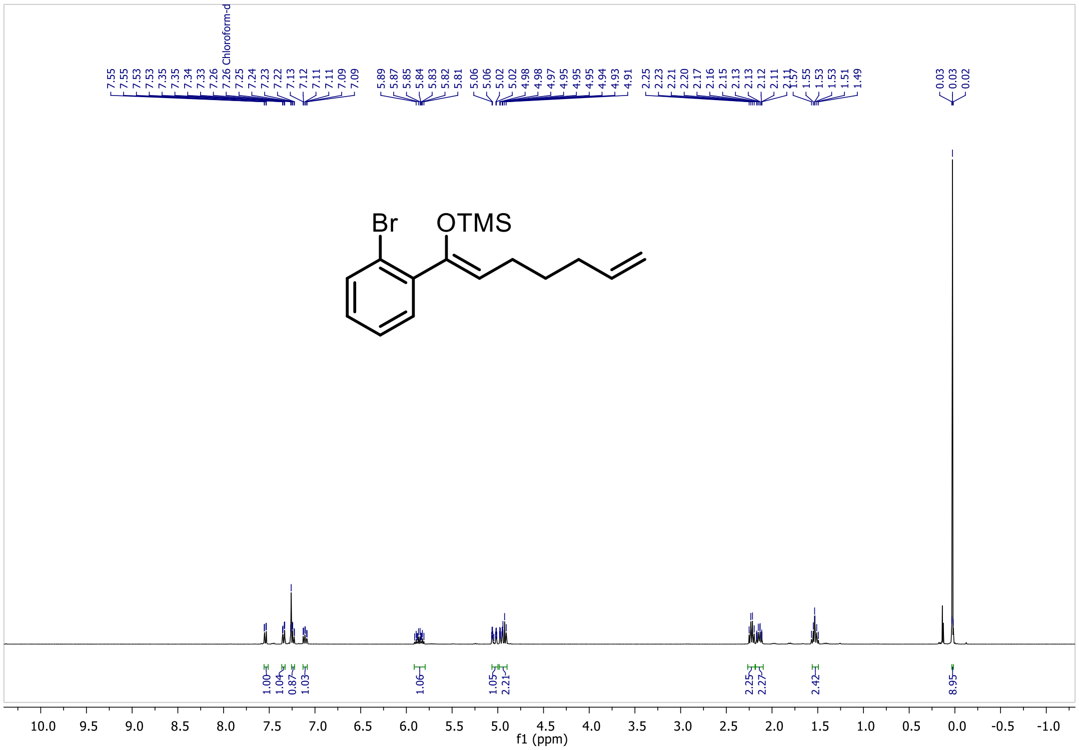

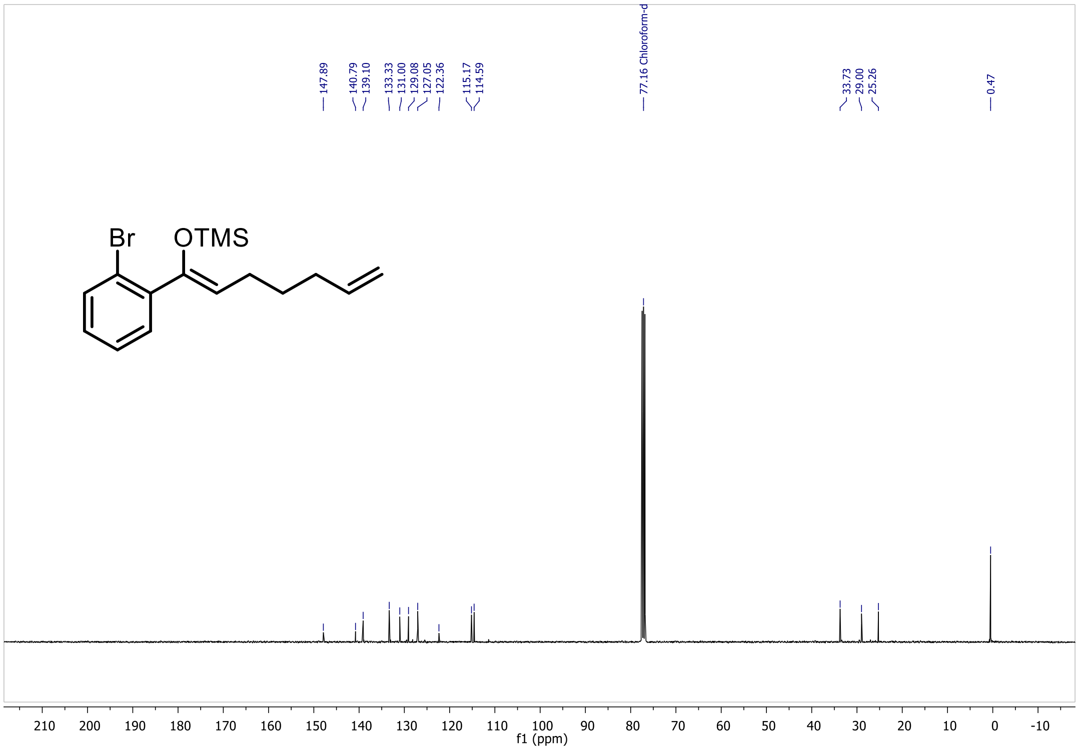

(*Z*)-((1-(4-(*tert*-Butyl)phenyl)hepta-1,6-dien-1-yl)oxy)trimethylsilane (1j)

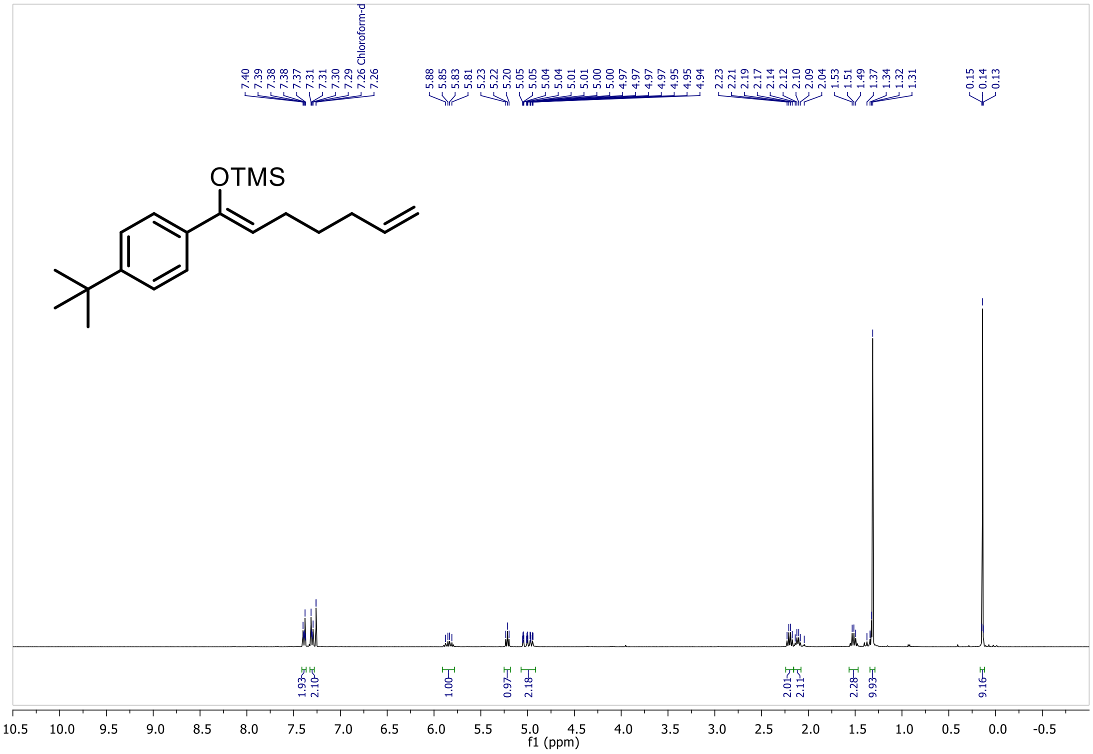

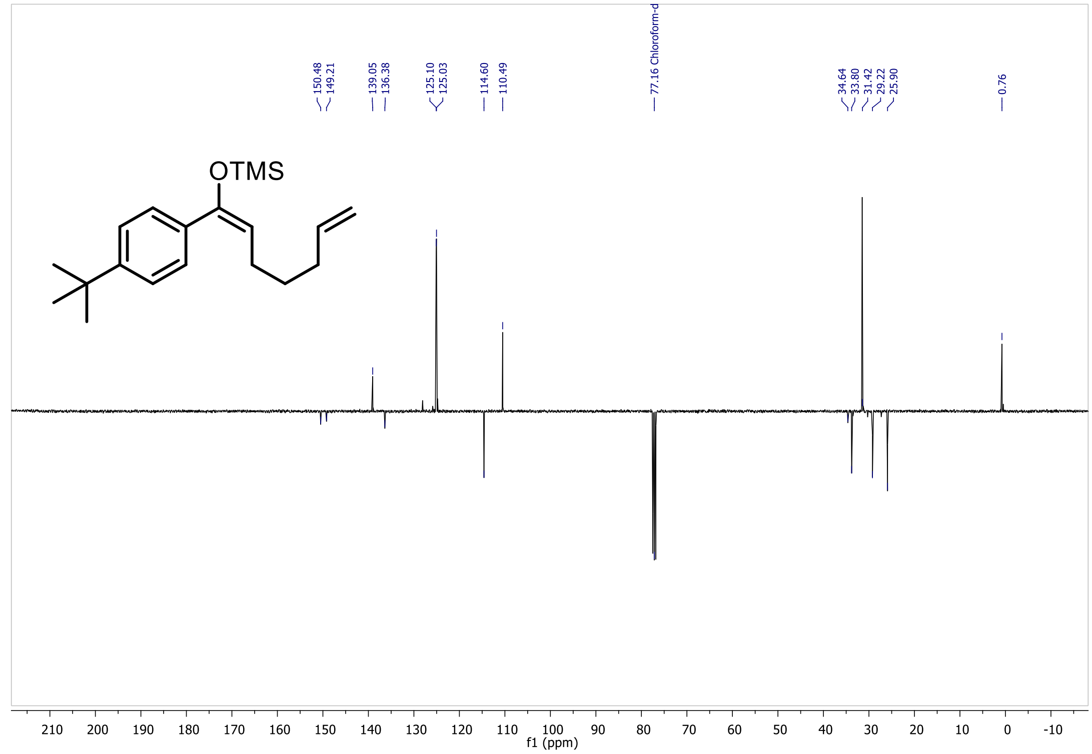

(*Z*)-4-(1-((Trimethylsilyl)oxy)hepta-1,6-dien-1-yl)benzonitrile (1k)

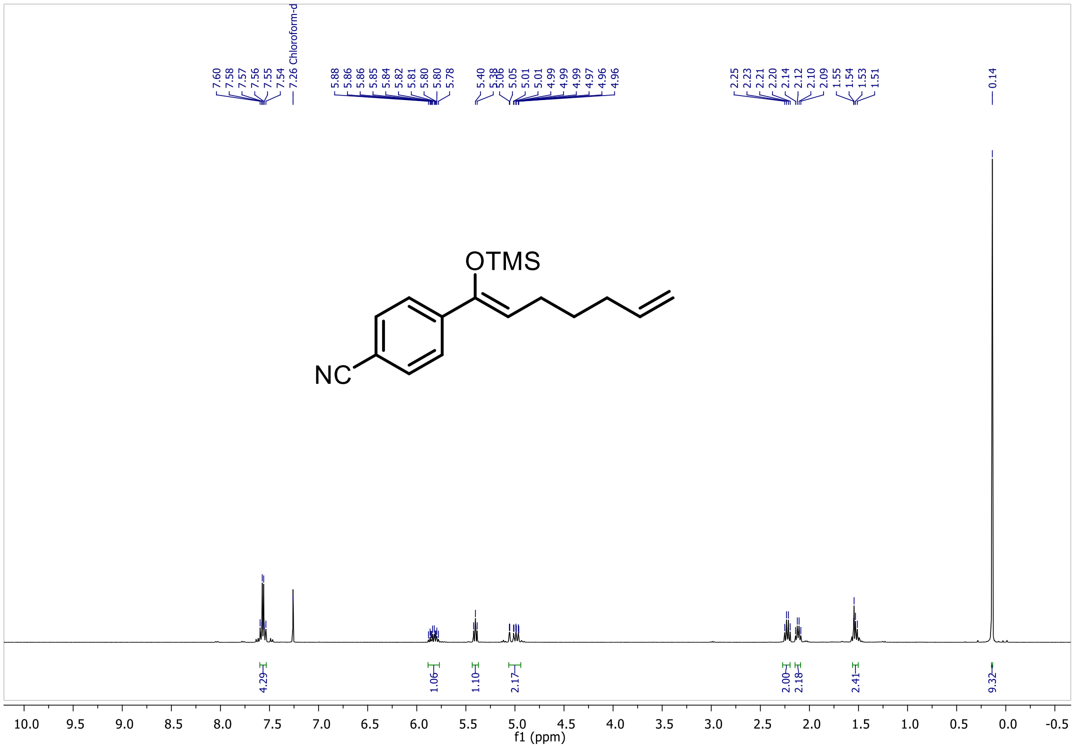

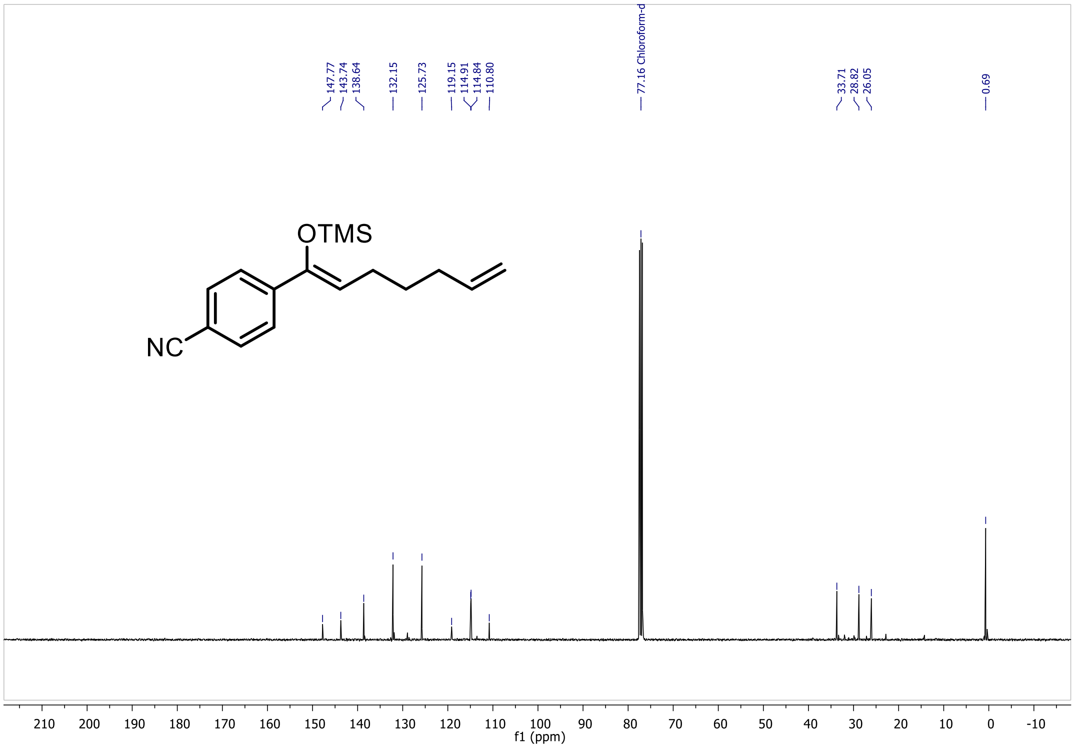

Methyl (*Z*)-4-(1-((trimethylsilyl)oxy)hepta-1,6-dien-1-yl)benzoate (1l)

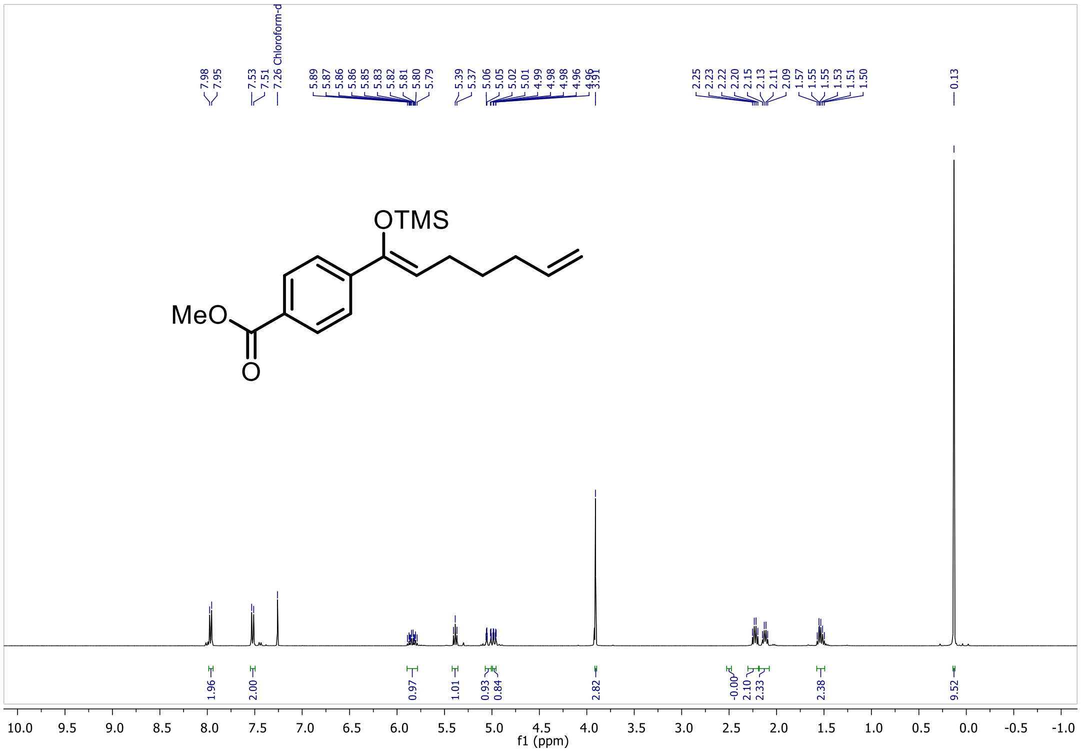

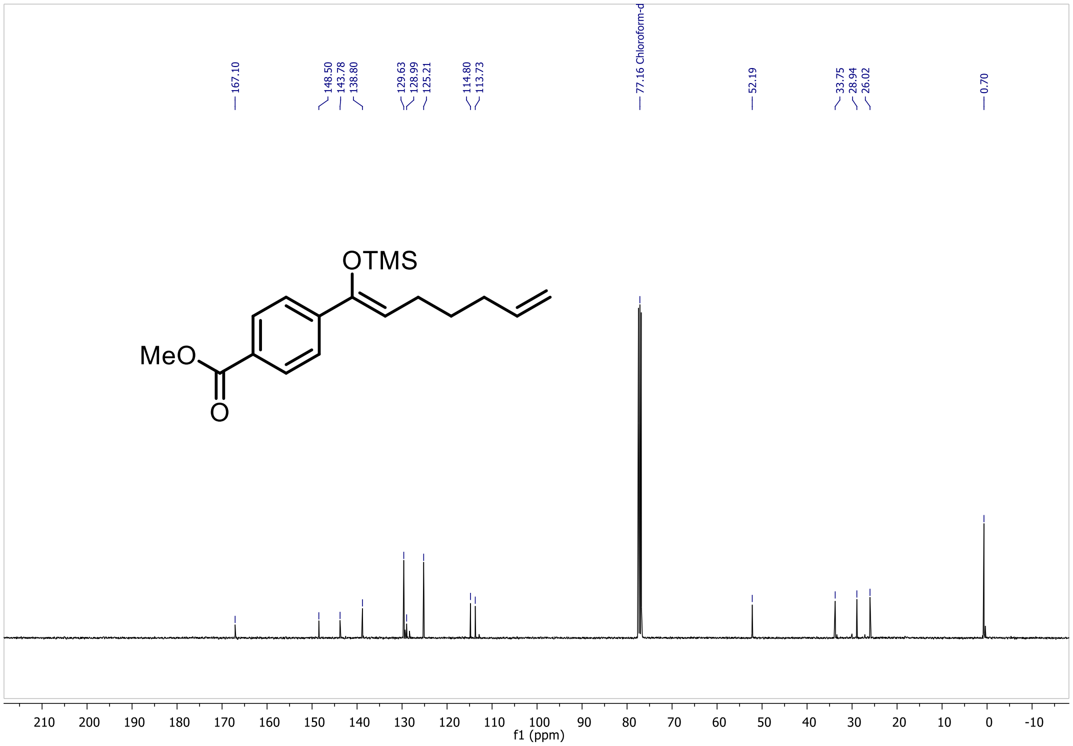

(Z)-Trimethyl((1-(4-(trifluoromethoxy)phenyl)hepta-1,6-dien-1-yl)oxy)silane (1m)

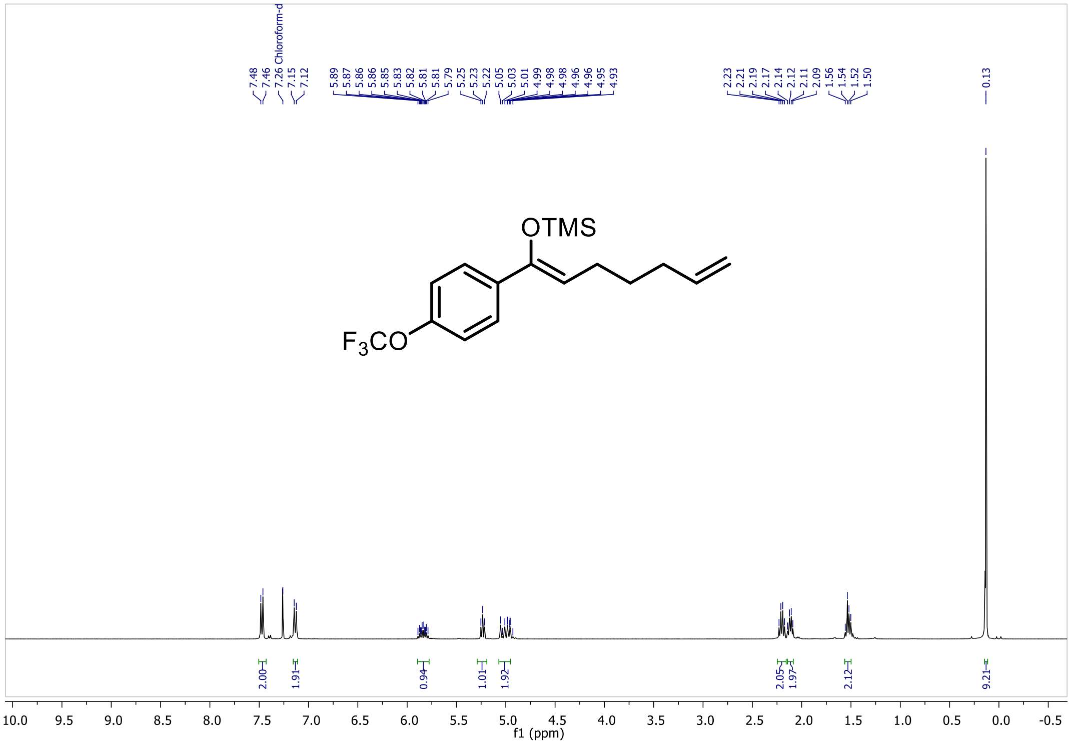

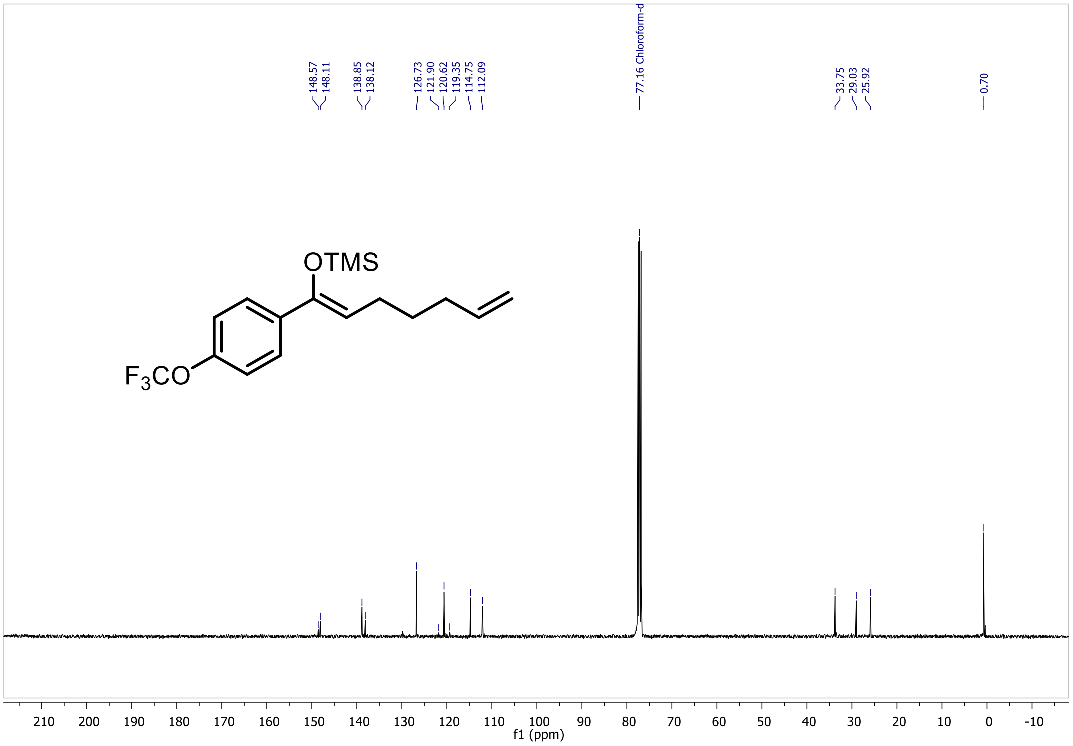

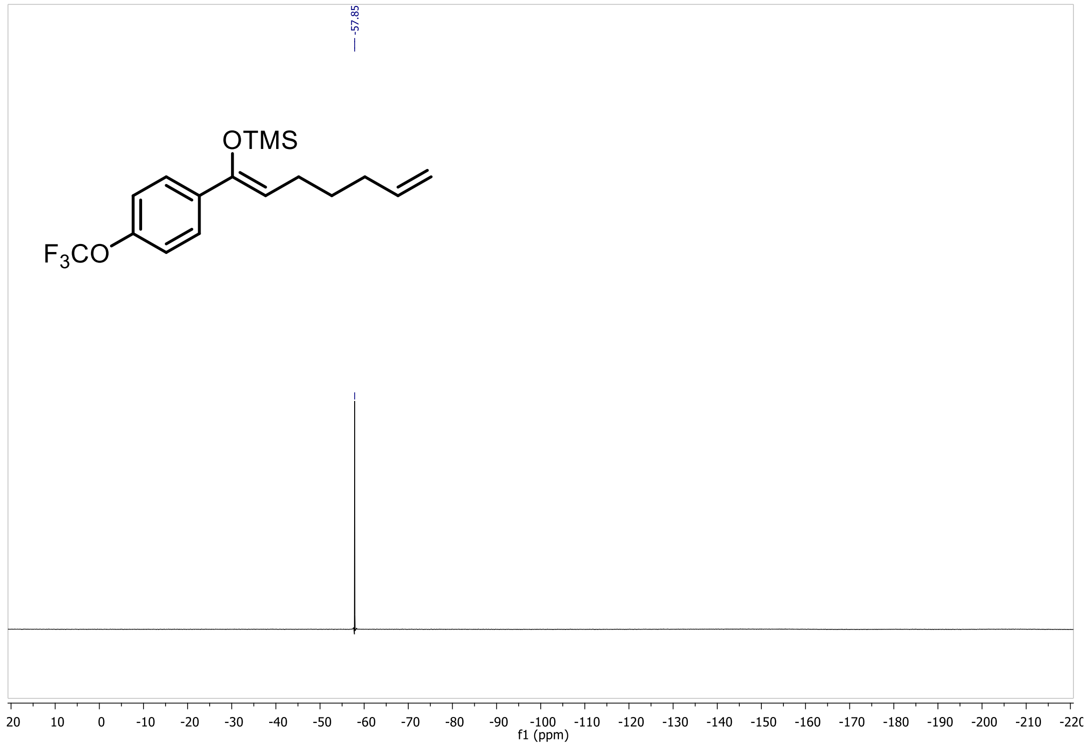

-57.85
OTMS
F3CO
20
10
0
-10
-20
-30
-40
-50
-60
-70
-80
-90
-100
-110
-120
-130
-140
-150
-160
-170
-180
-190
-200
-210
f1 (ppm)

(*Z*)-Trimethyl((1-(thiophen-2-yl)hepta-1,6-dien-1-yl)oxy)silane (1n)

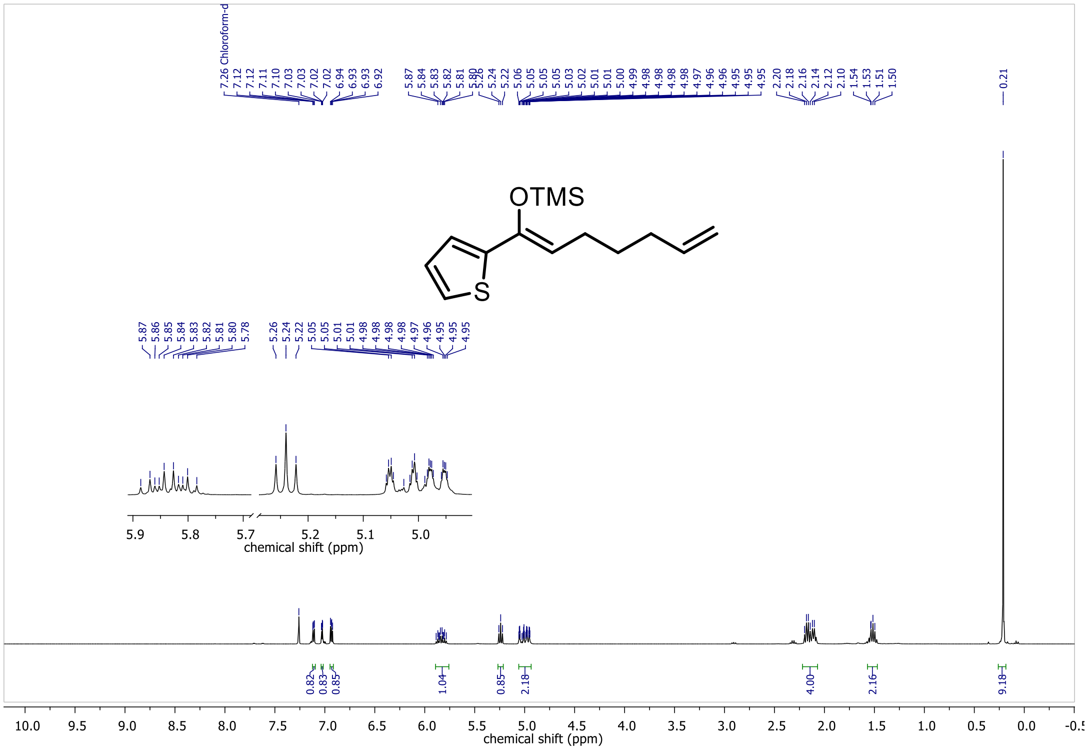

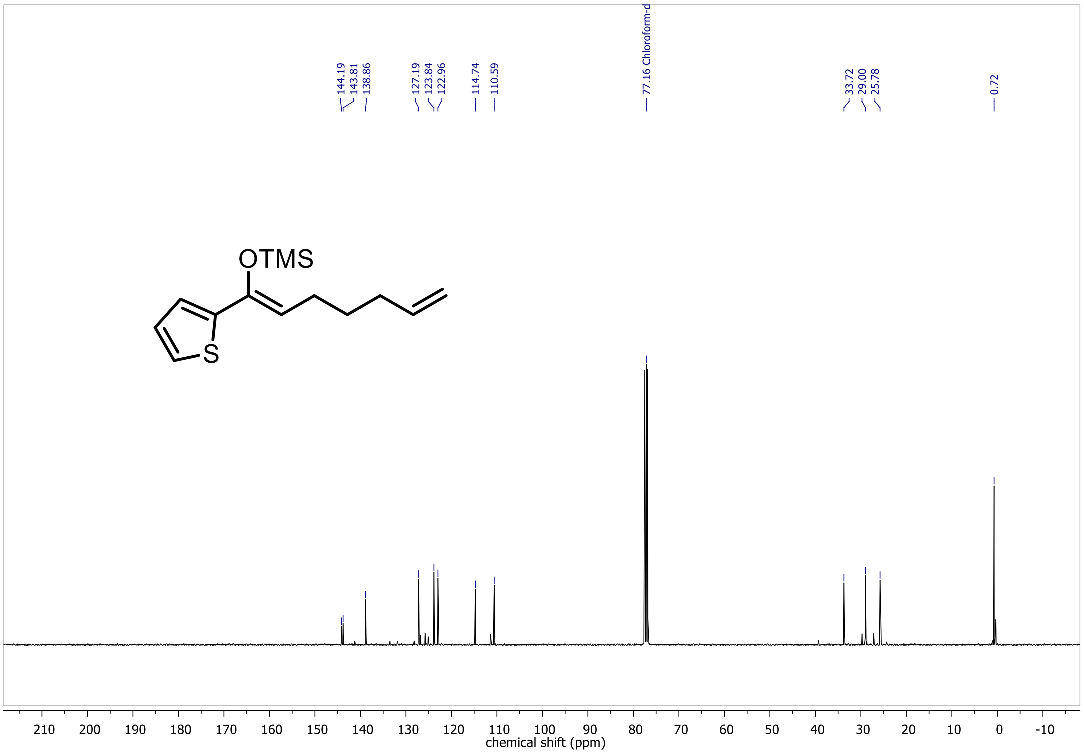

(*Z*)-((1-(Furan-2-yl)hepta-1,6-dien-1-yl)oxy)trimethylsilane (1o)

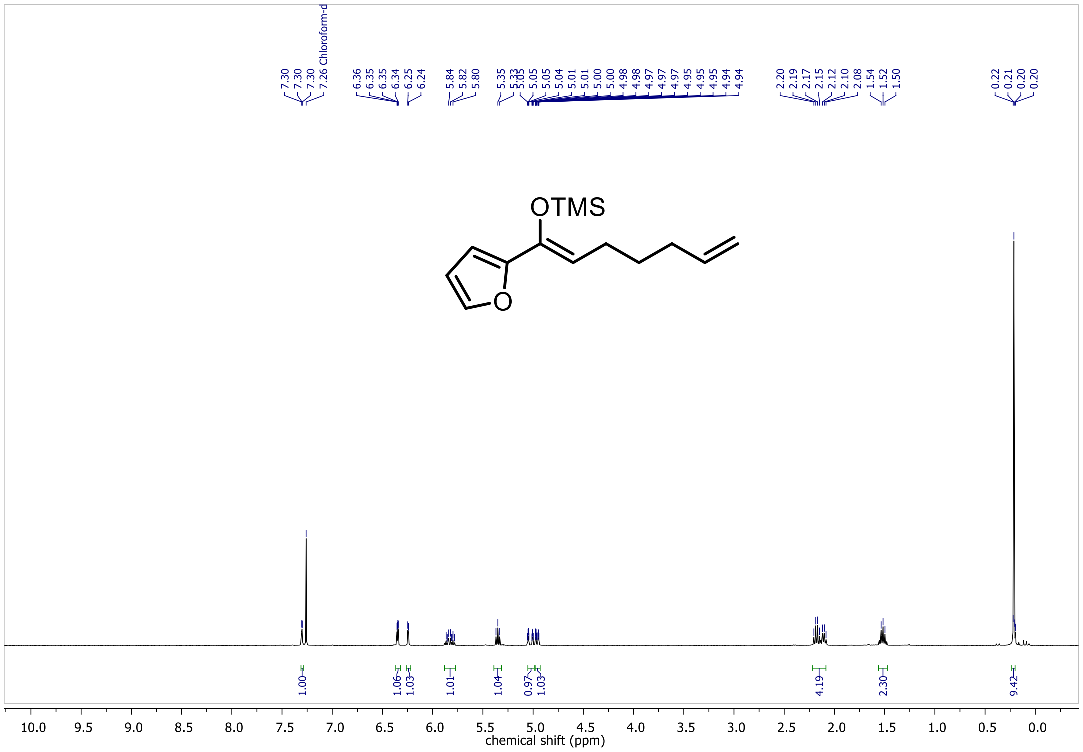

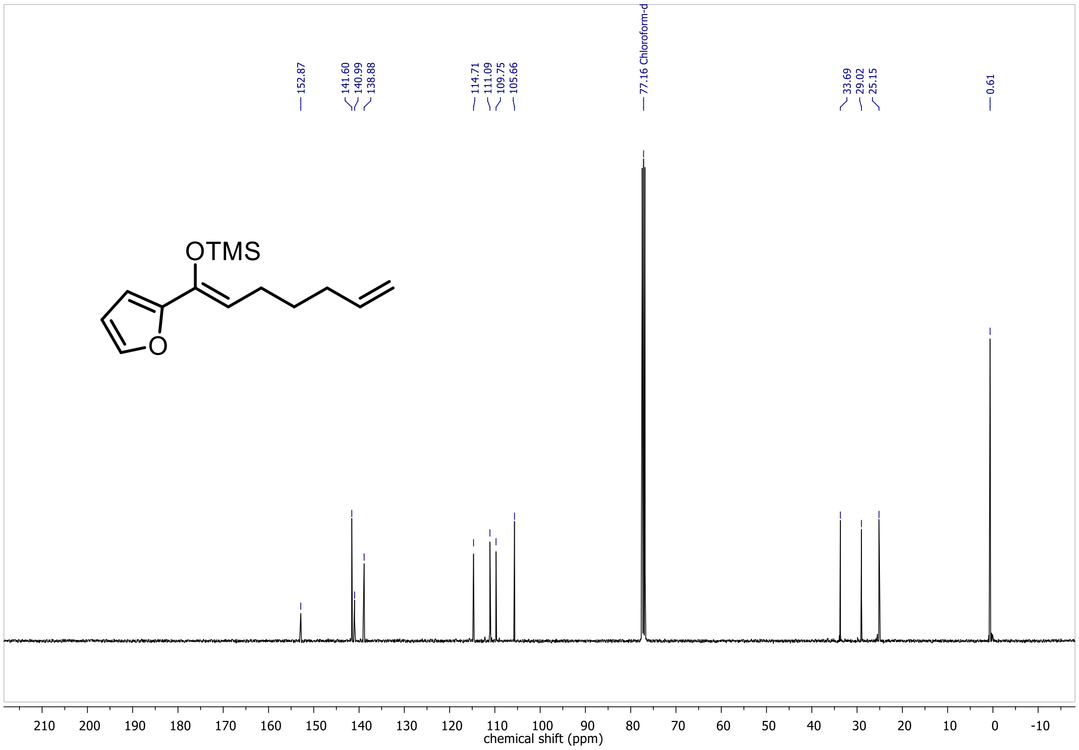

(*Z*)-((2,2-Dimethylnona-3,8-dien-3-yl)oxy)trimethylsilane (1p)

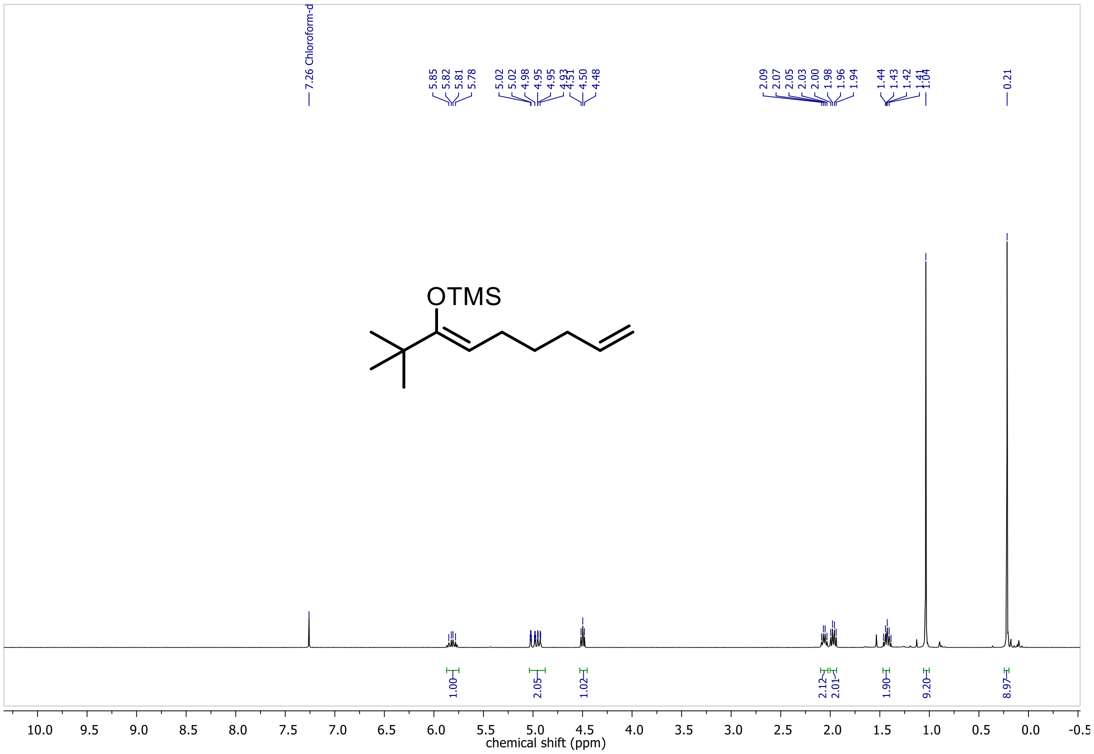

(((*Z*)-1-(Adamantan-1-yl)hepta-1,6-dien-1-yl)oxy)trimethylsilane (1q)

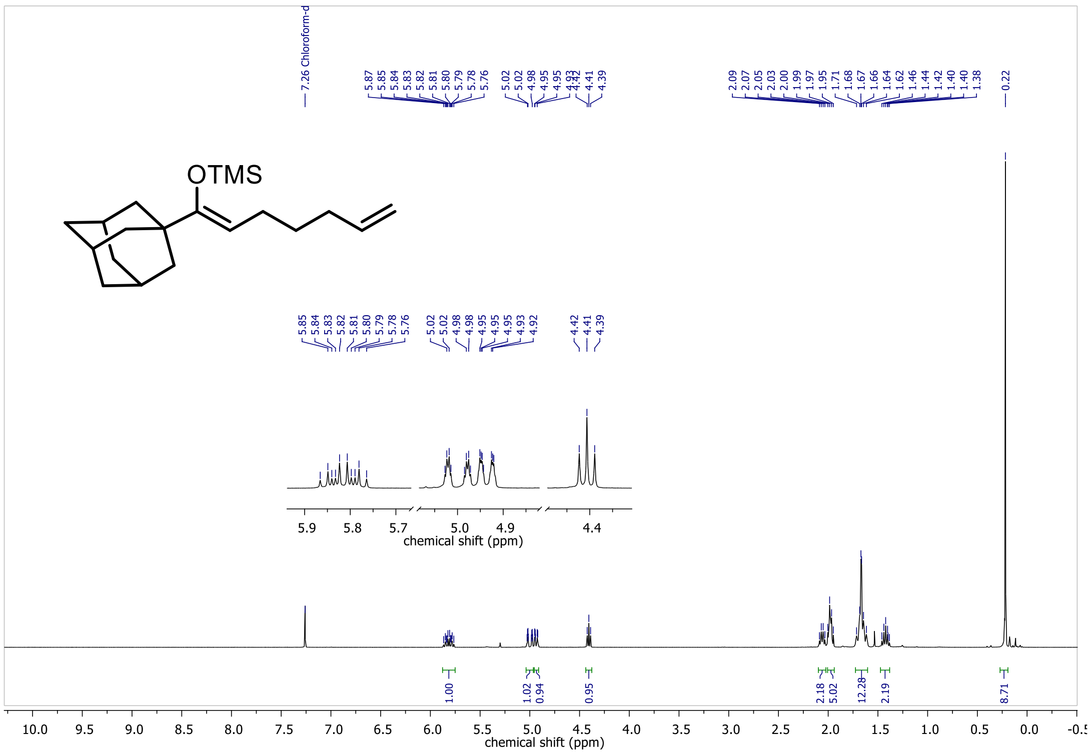

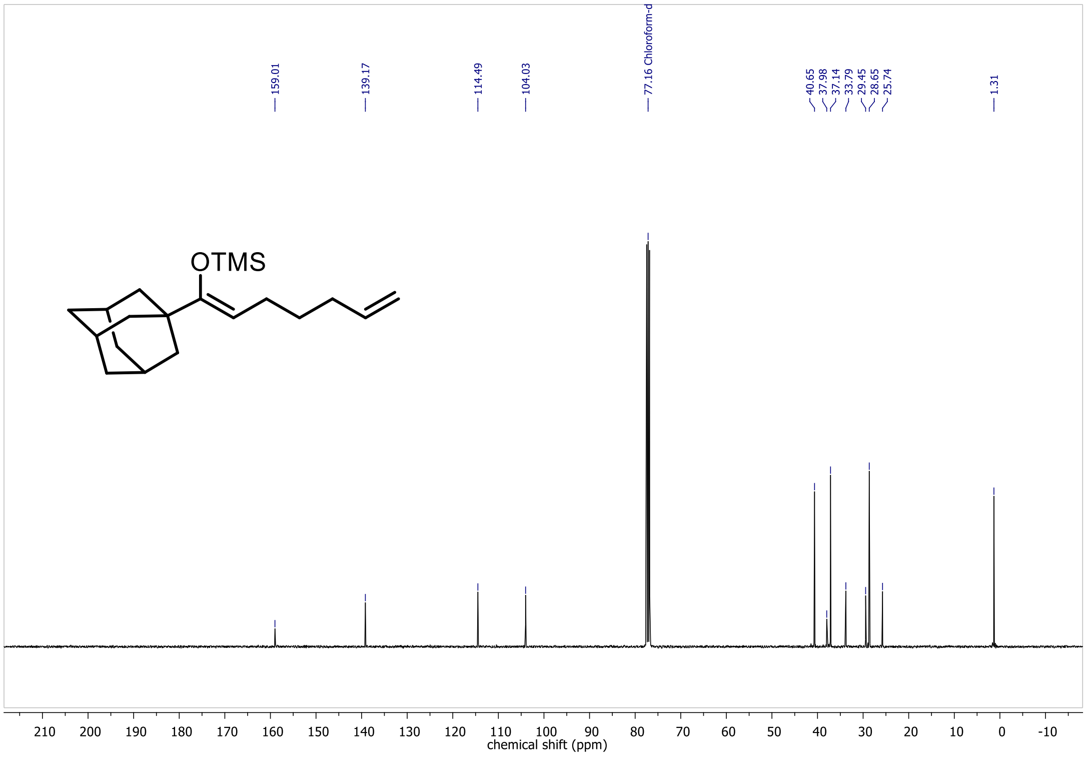

((1-cyclohexylhepta-1,6-dien-1-yl)oxy)trimethylsilane (1r)

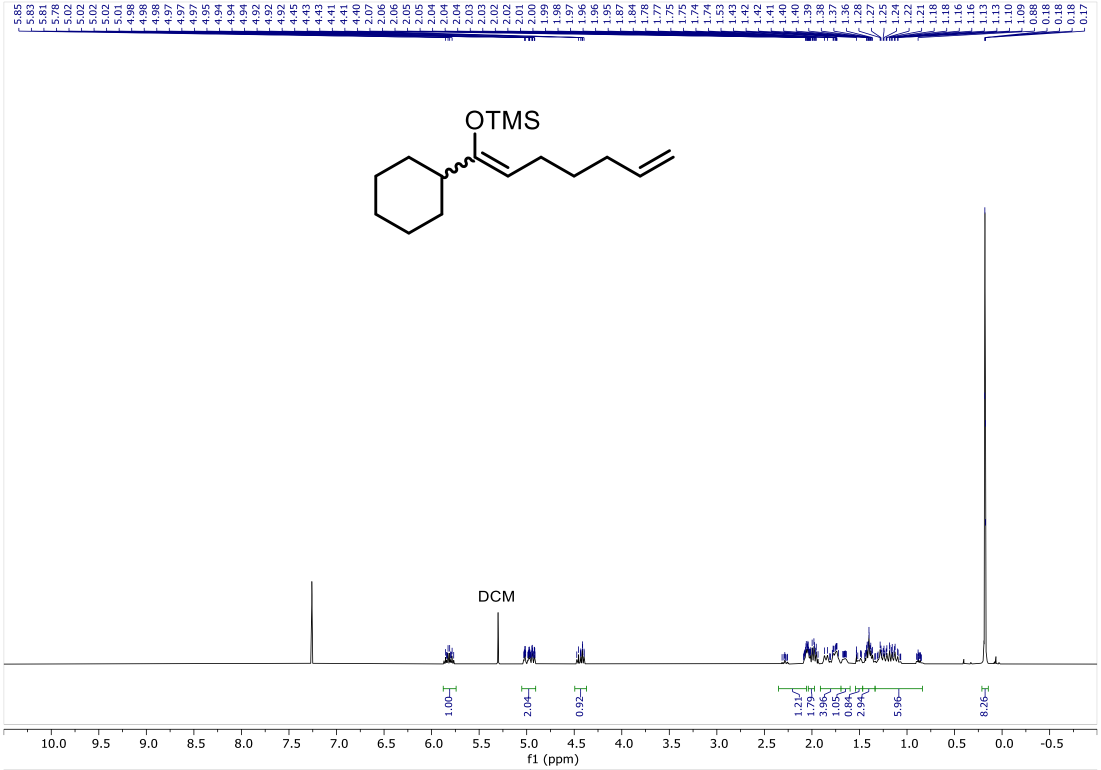

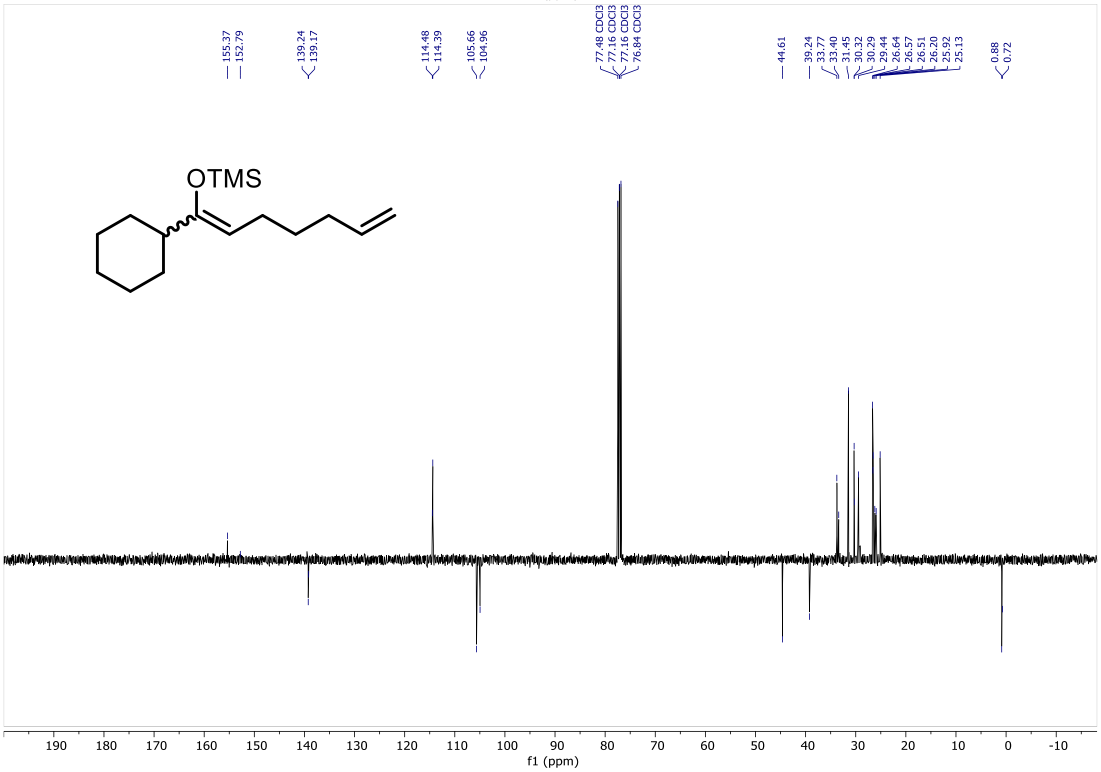

Trimethyl((5-methyl-1-phenylhepta-1,6-dien-1-yl)oxy)silane (1s)

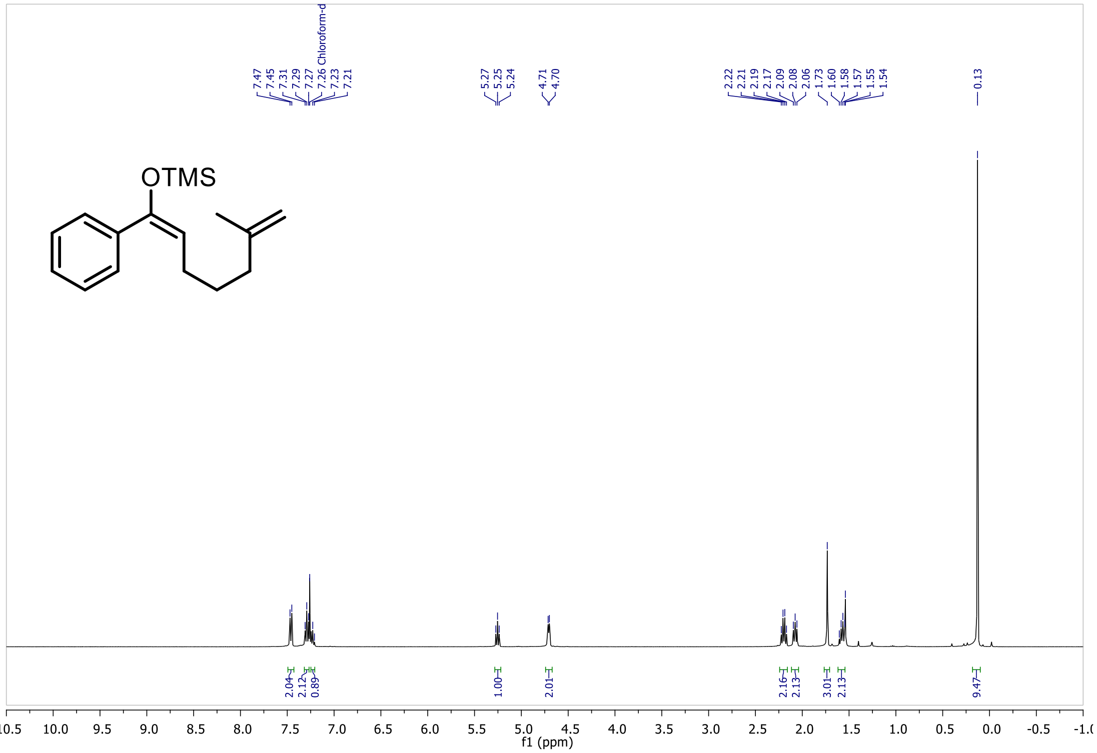

(*Z*)-((1,6-Diphenylhepta-1,6-dien-1-yl)oxy)trimethylsilane (1t)

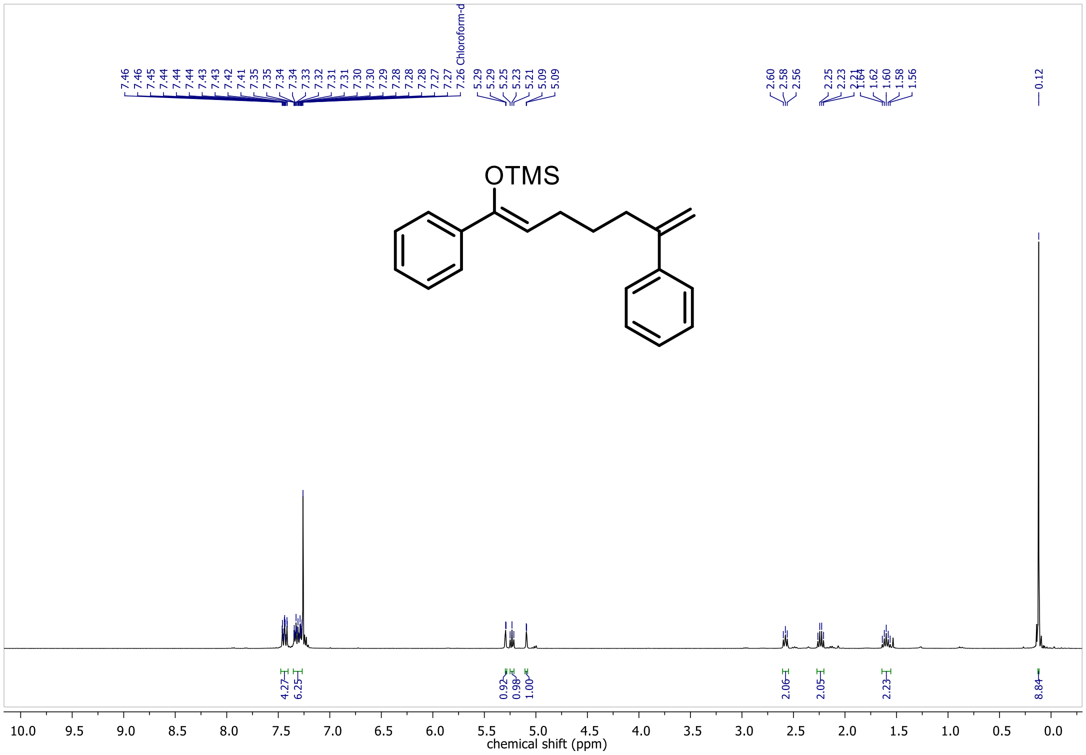

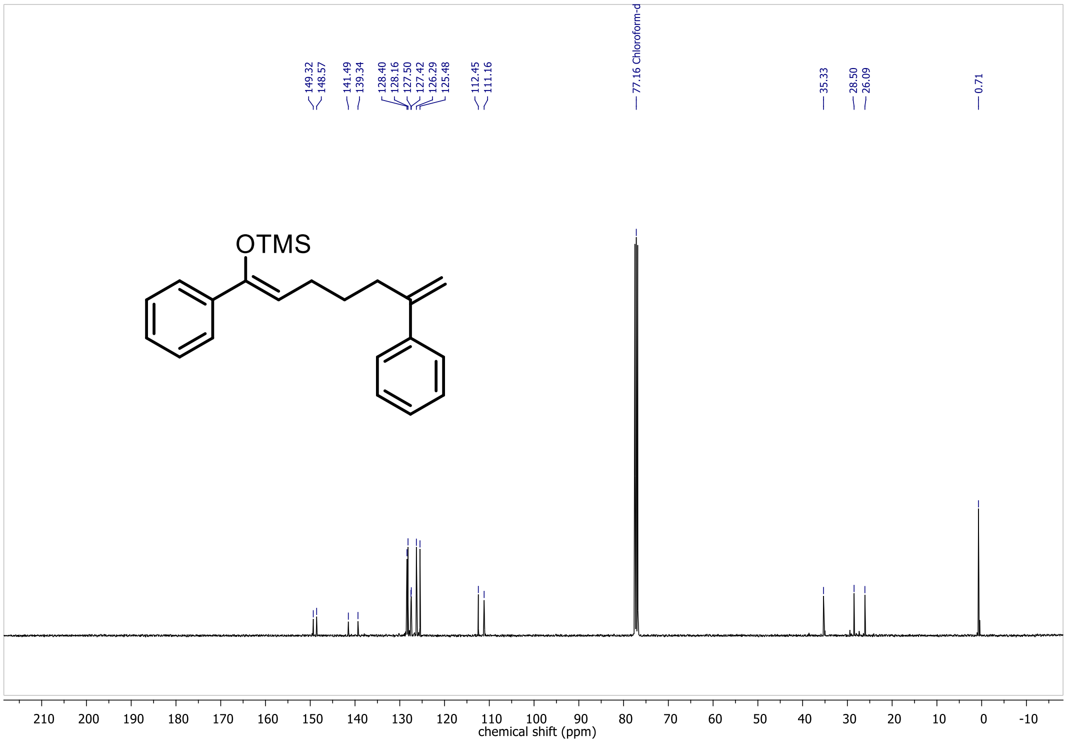

(*Z*)-Trimethyl((6-(naphthalen-2-yl)-1-phenylhepta-1,6-dien-1-yl)oxy)silane (1u)

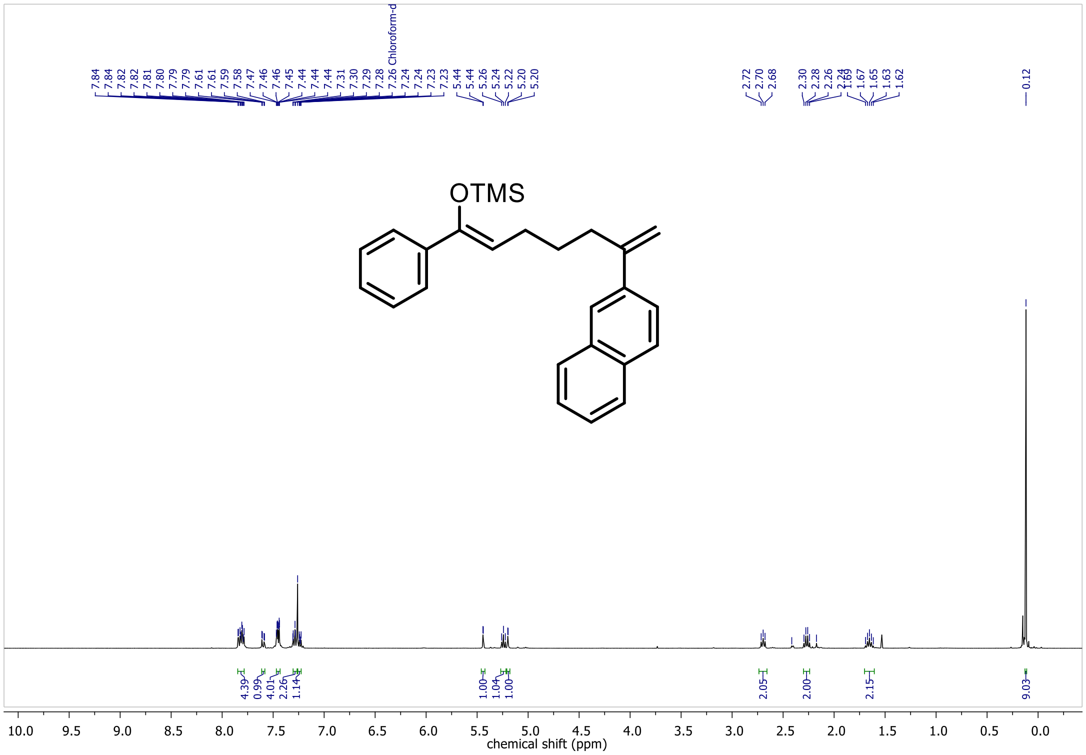

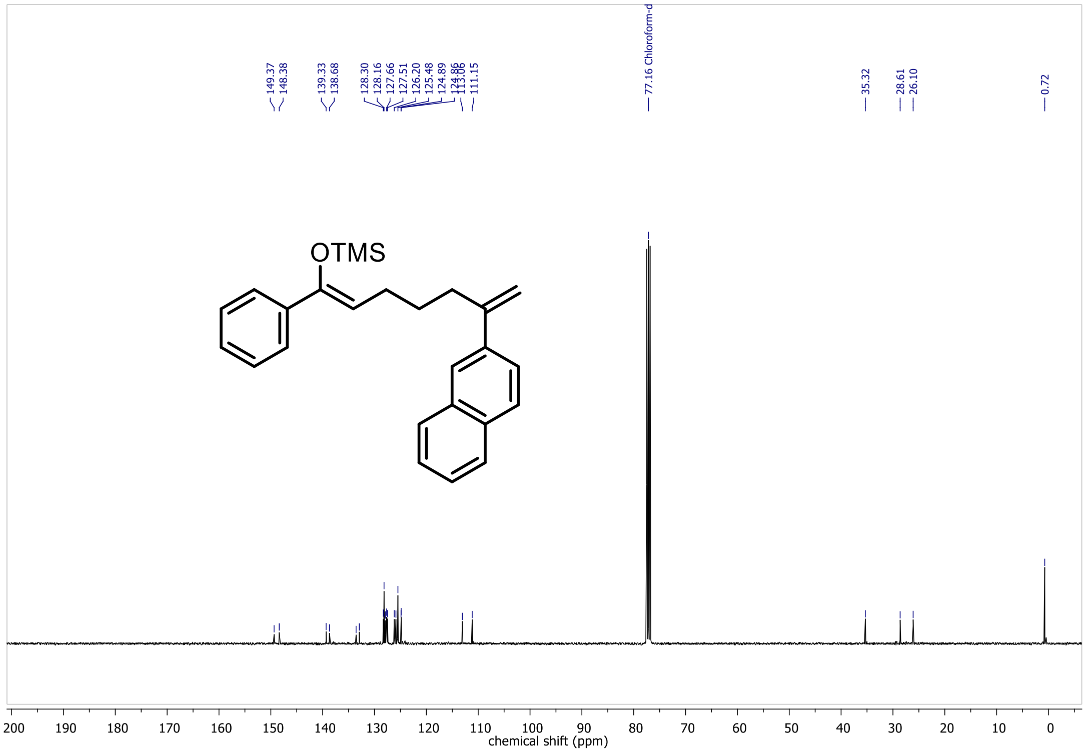

(*Z*)-((6-(Dibenzo[*b,d*]furan-1-yl)-1-phenylhepta-1,6-dien-1-yl)oxy)trimethylsilane (1v)

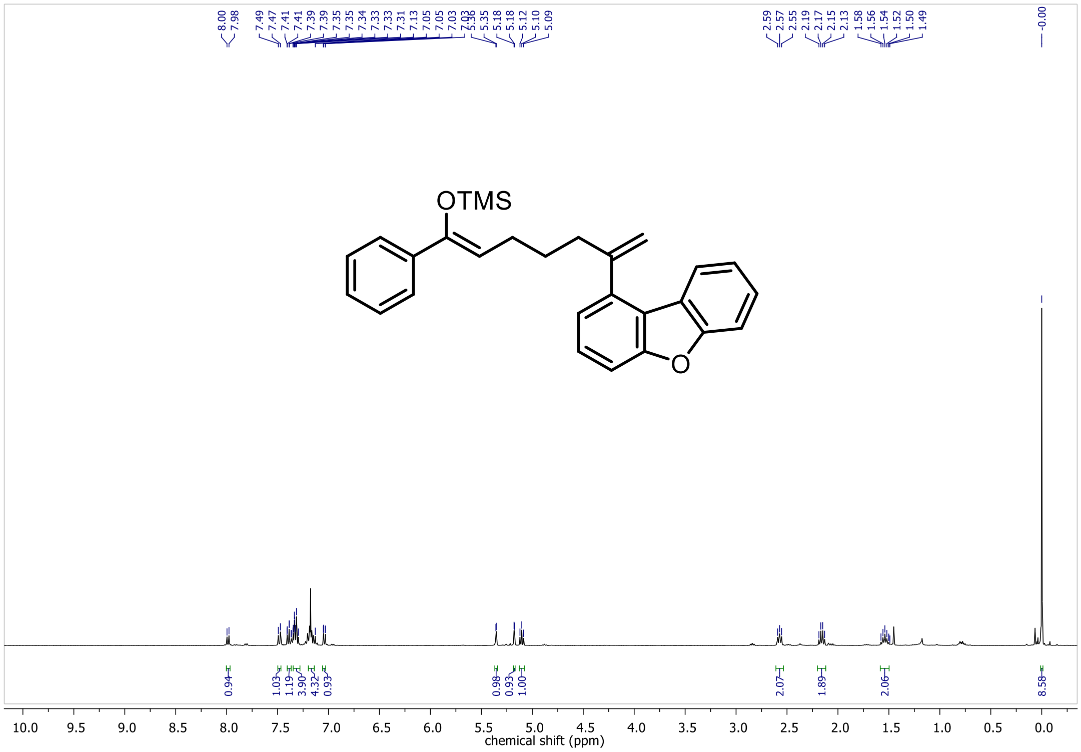

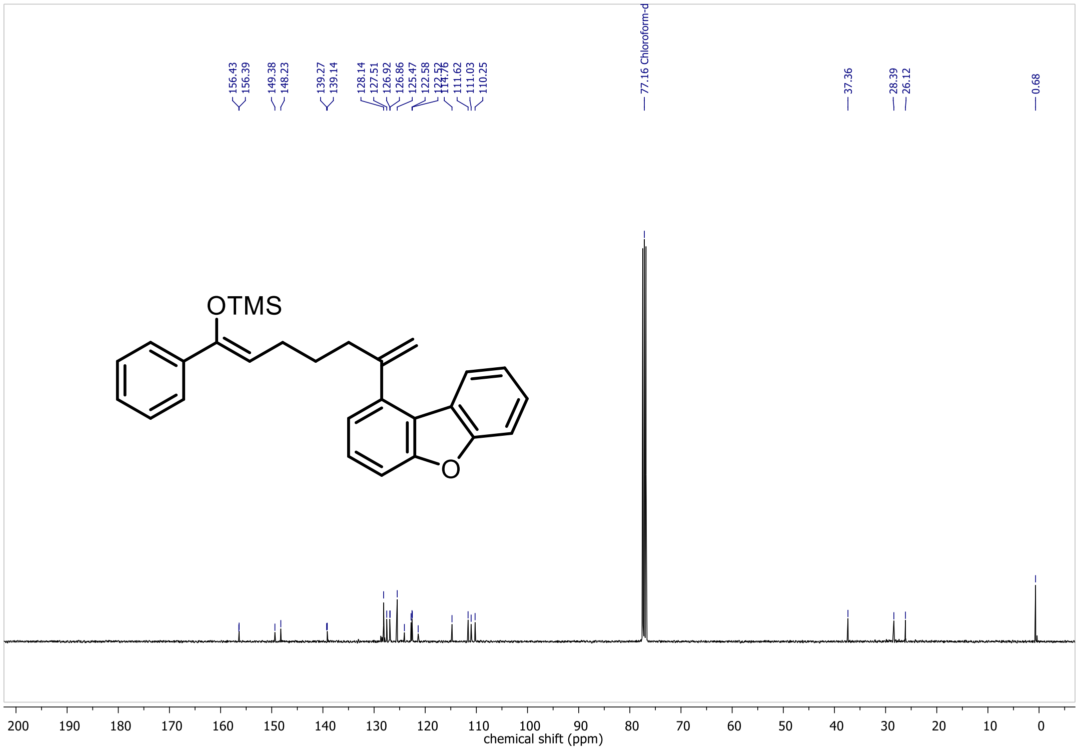

(Z)-Trimethyl((7-methyl-1-phenylocta-1,6-dien-1-yl)oxy)silane (1y)

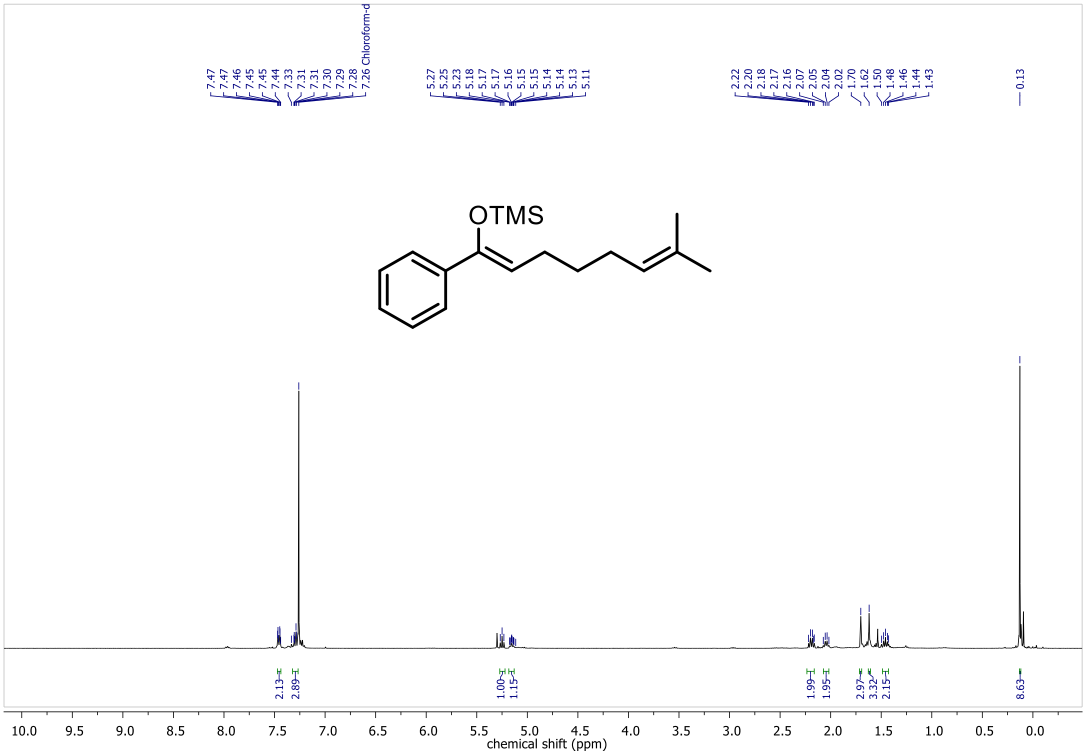

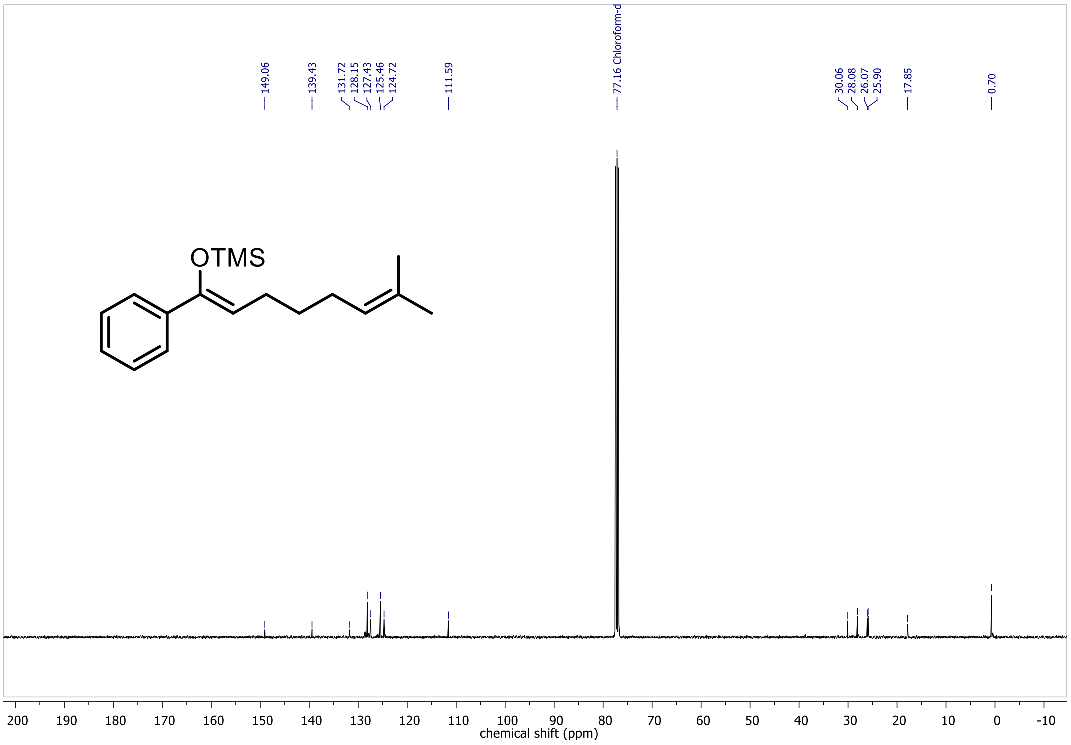

(*Z*)-Trimethyl((1-(4-(trifluoromethyl)phenyl)hepta-1,6-dien-1-yl-7,7-d2)oxy)silane (1f-d_2)

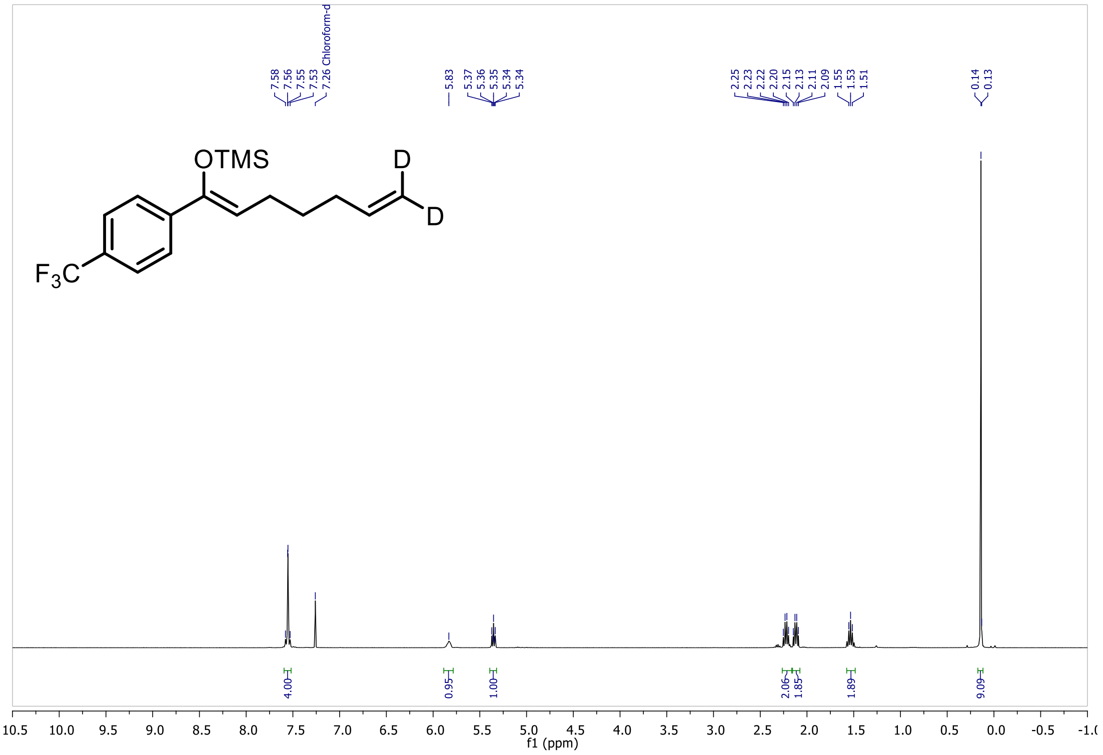

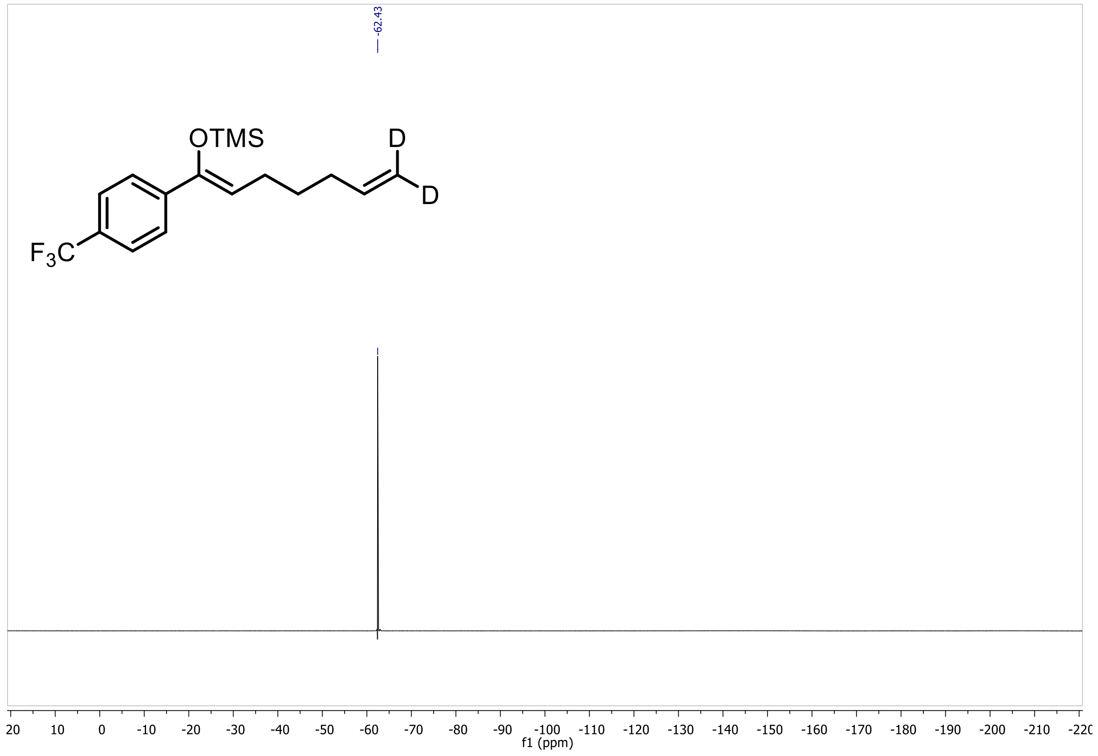

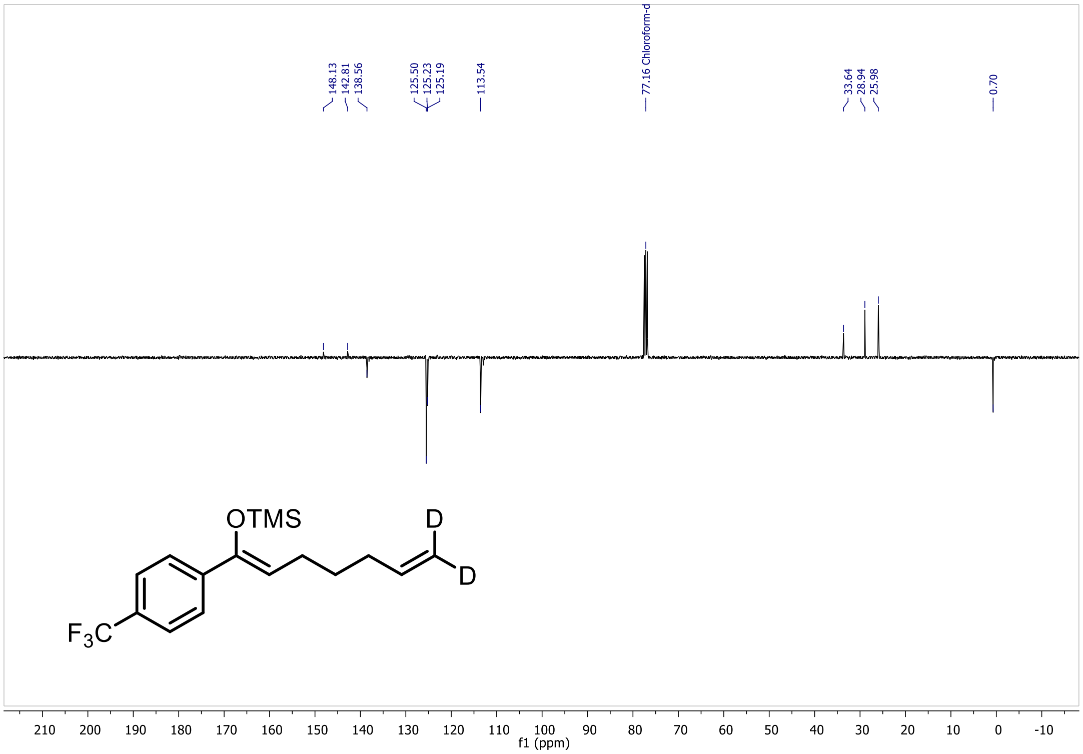
148.13
142.81
138.56
125.50
125.23
125.19
113.54
77.16 Chloroform-d
33.64
28.94
25.98
0.70
OTMS
D
D
F3C
210 200 190 180 170 160 150 140 130 120 110 100 90 80 70 60 50 40 30 20 10 0 -10
f1 (ppm)

***cis*-3-Hydroxycyclohexyl)(phenyl)methanone (2a)**

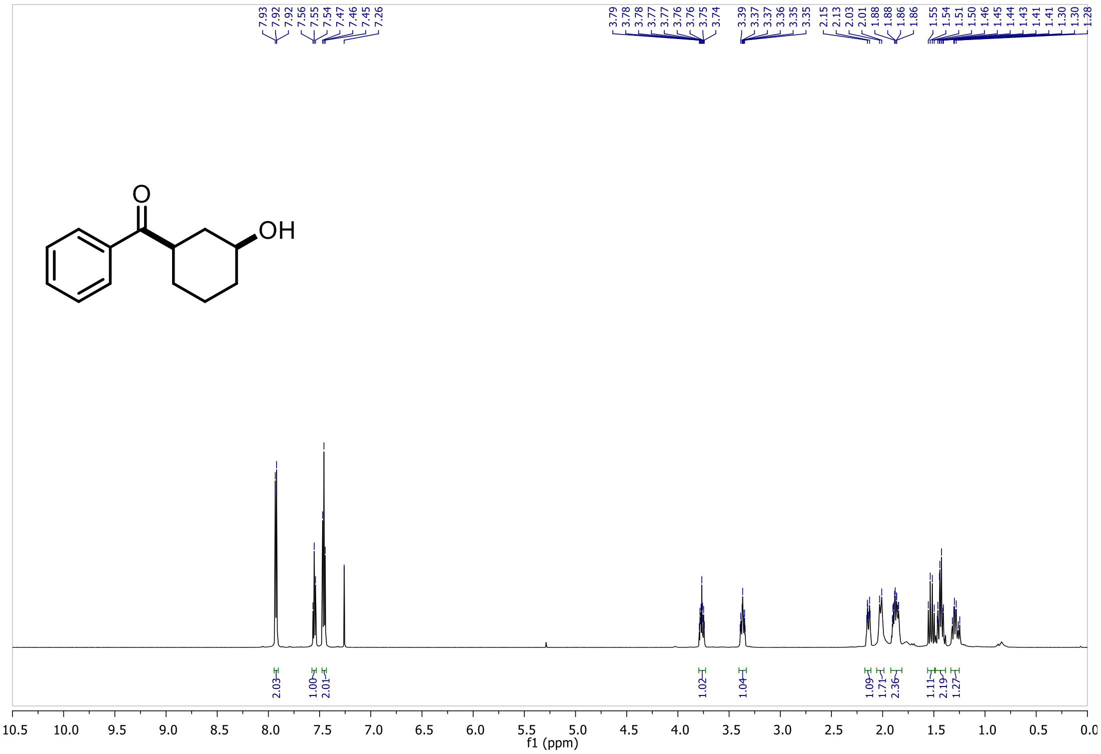

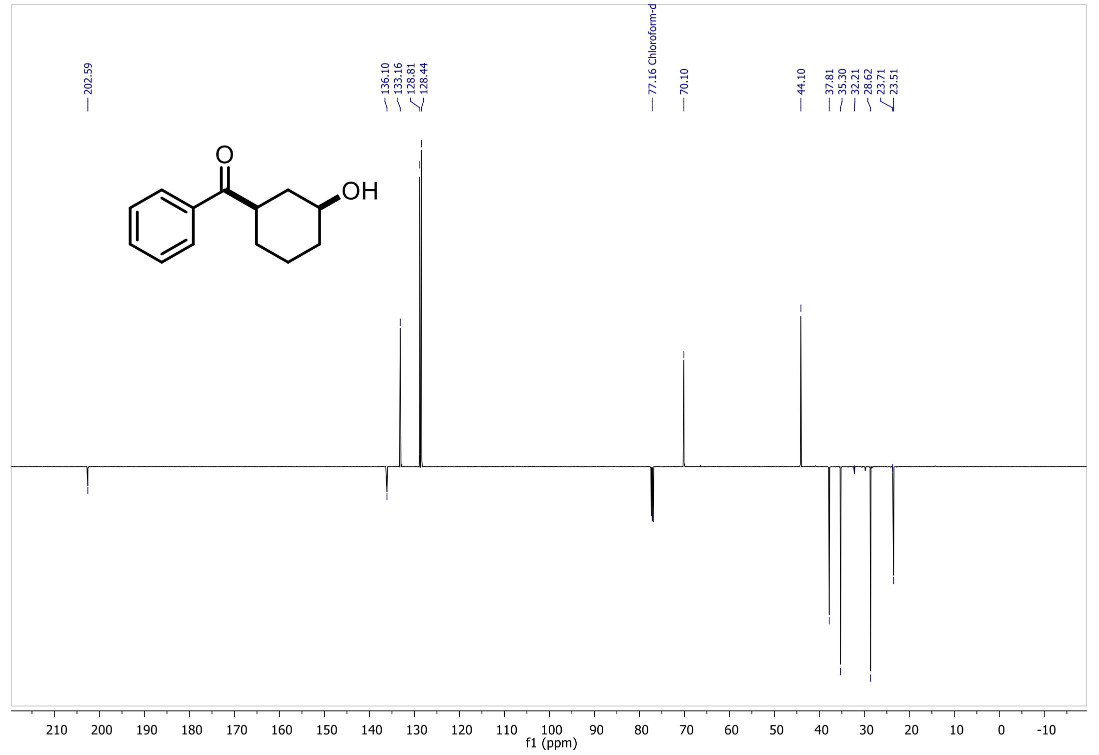

cis-(3-Fluorophenyl)(3-hydroxycyclohexyl)methanone (2b)

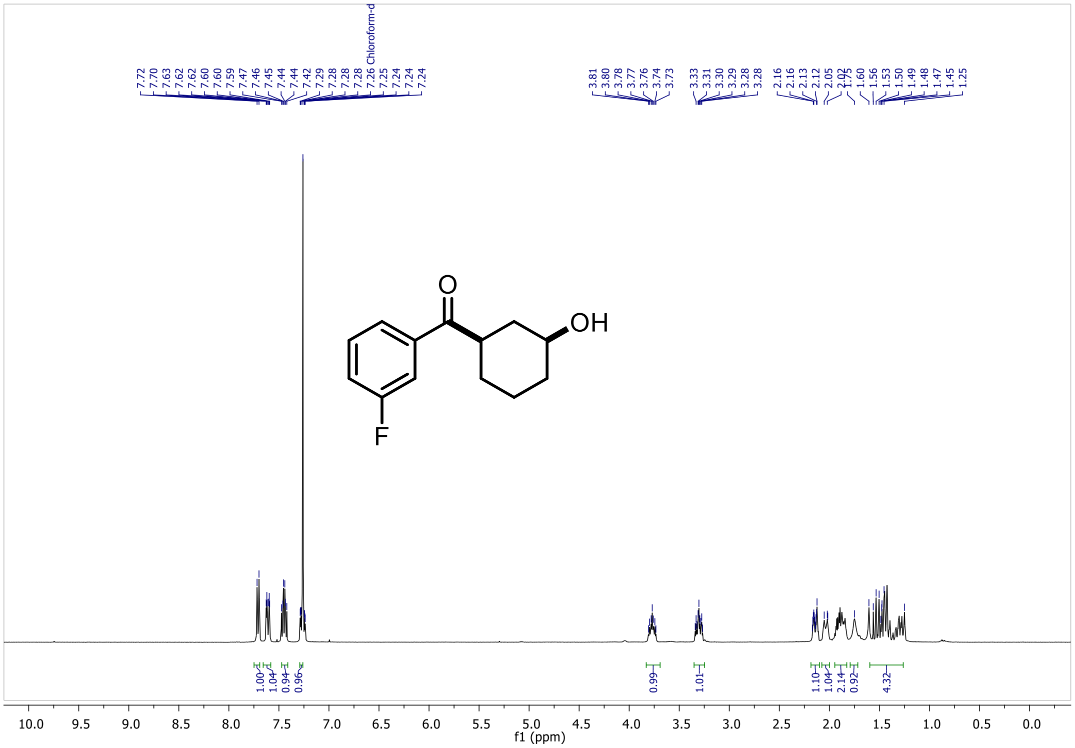

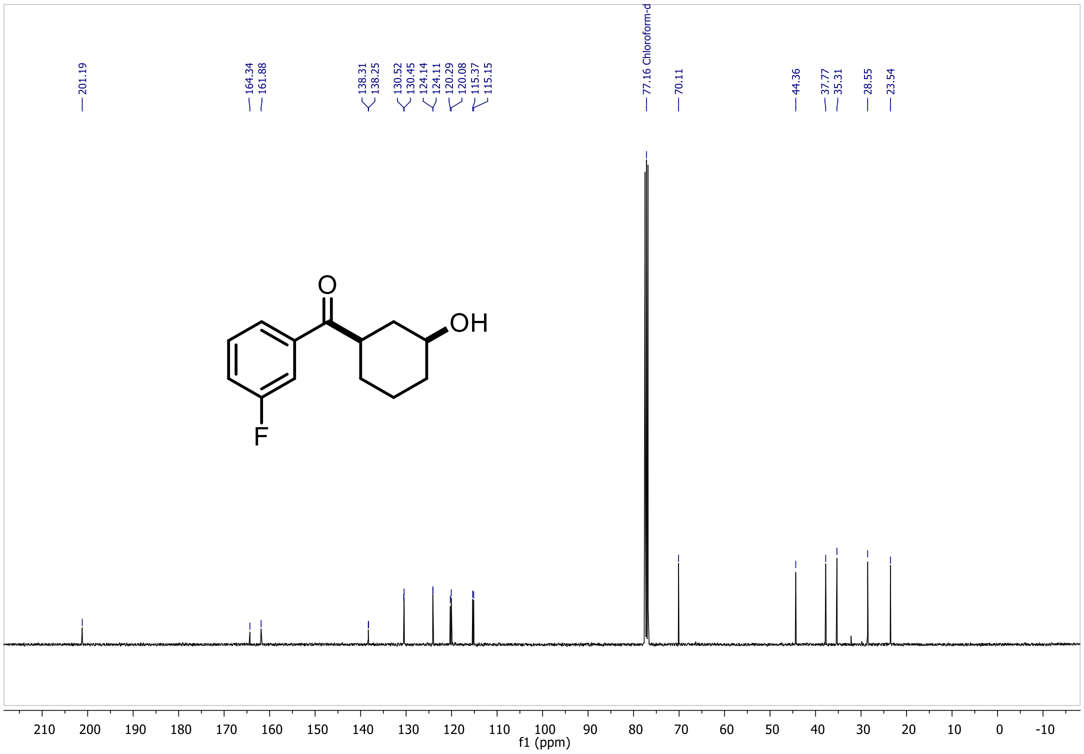

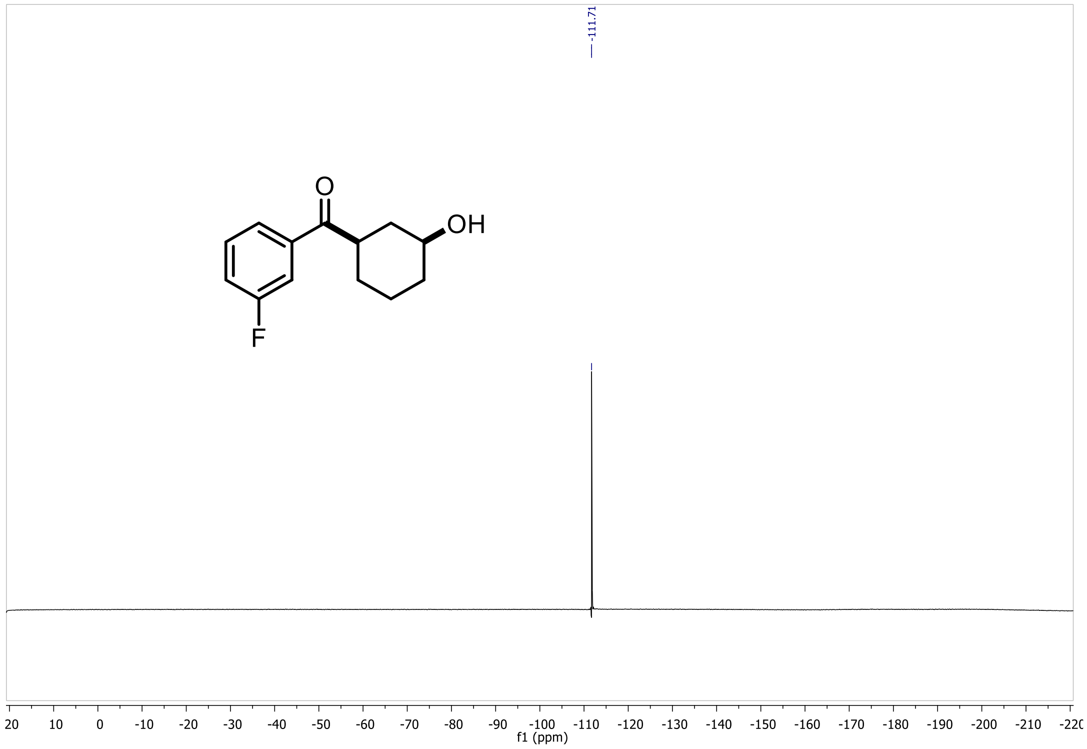

-111.71
O
OH
F
20 10 0 -10 -20 -30 -40 -50 -60 -70 -80 -90 -100 -110 -120 -130 -140 -150 -160 -170 -180 -190 -200 -210 -220
f1 (ppm)

***cis*-(4-Fluorophenyl)(3-hydroxycyclohexyl)methanone (2c)**

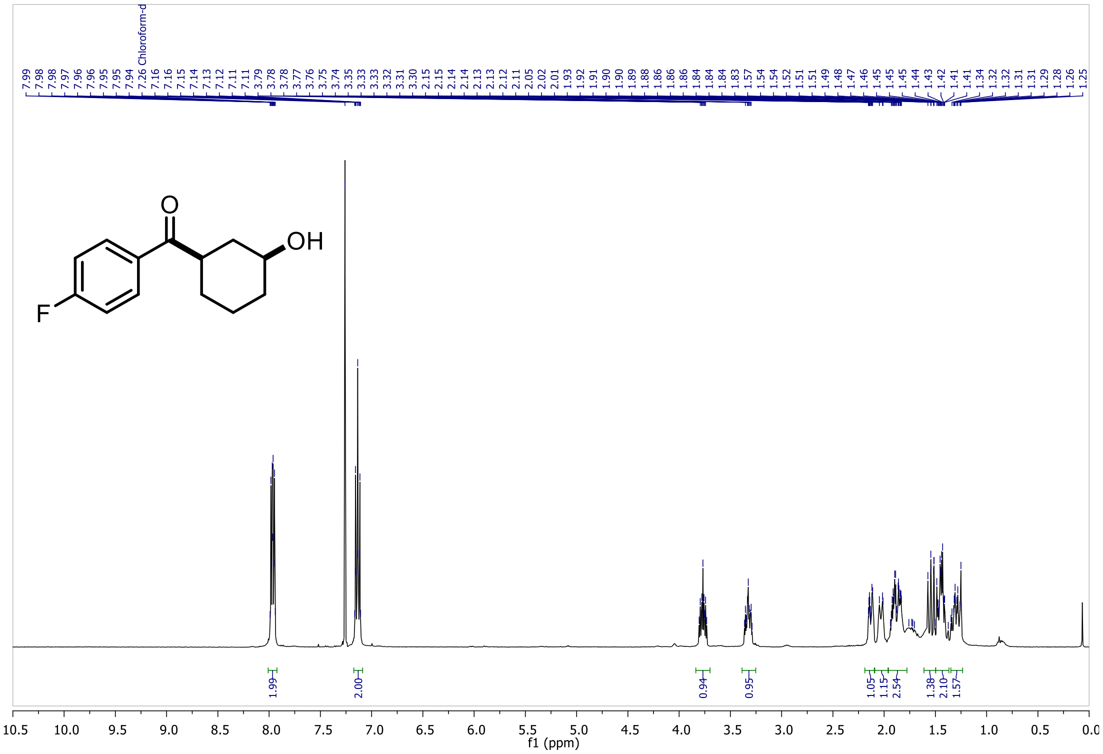

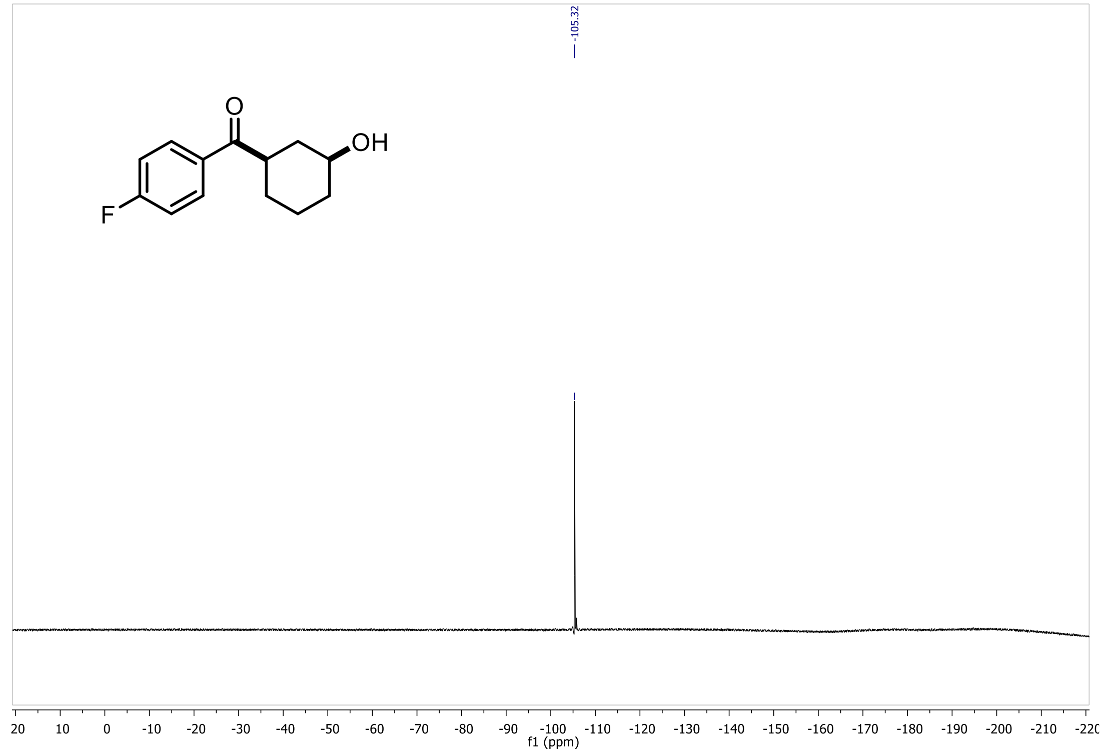

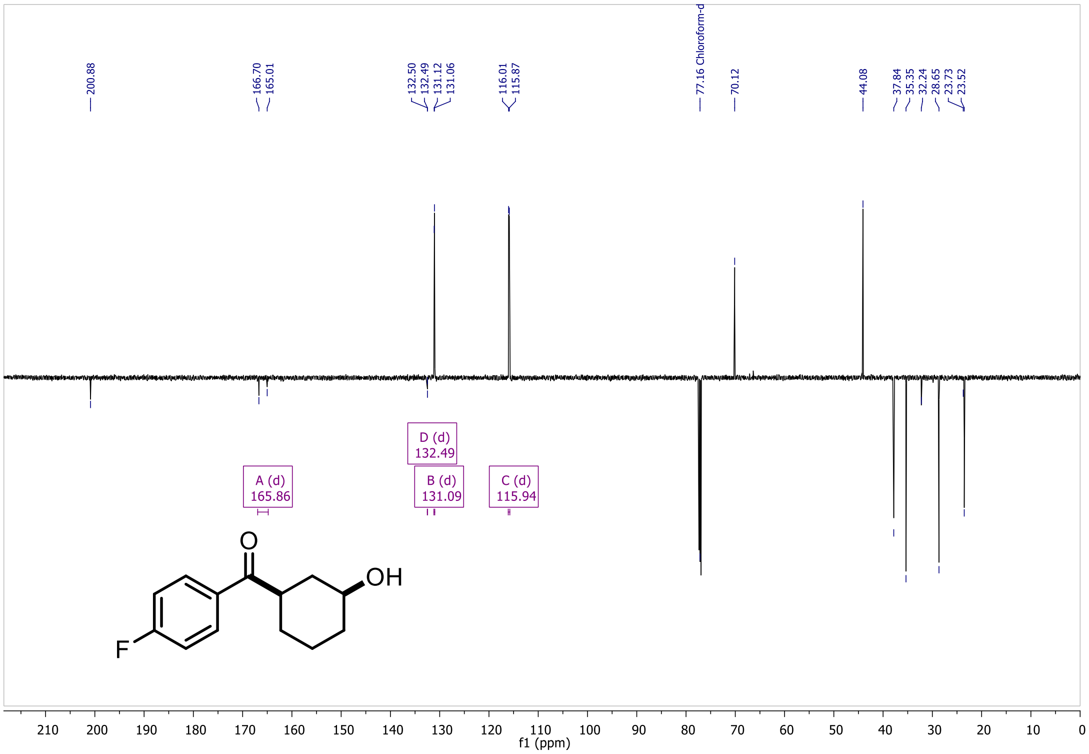

200.88
166.70
165.01
132.50
132.49
131.12
131.06
116.01
115.87
77.16 Chloroform-d
70.12
44.08
37.84
35.35
32.24
28.65
23.73
23.52
D (d)
132.49
A (d)
165.86
B (d)
131.09
C (d)
115.94
O
OH
F
210
200
190
180
170
160
150
140
130
120
110
100
90
80
70
60
50
40
30
20
10
0
f1 (ppm)

cis-(3,5-Bis(trifluoromethyl)phenyl)(3-hydroxycyclohexyl)methanone (2d)

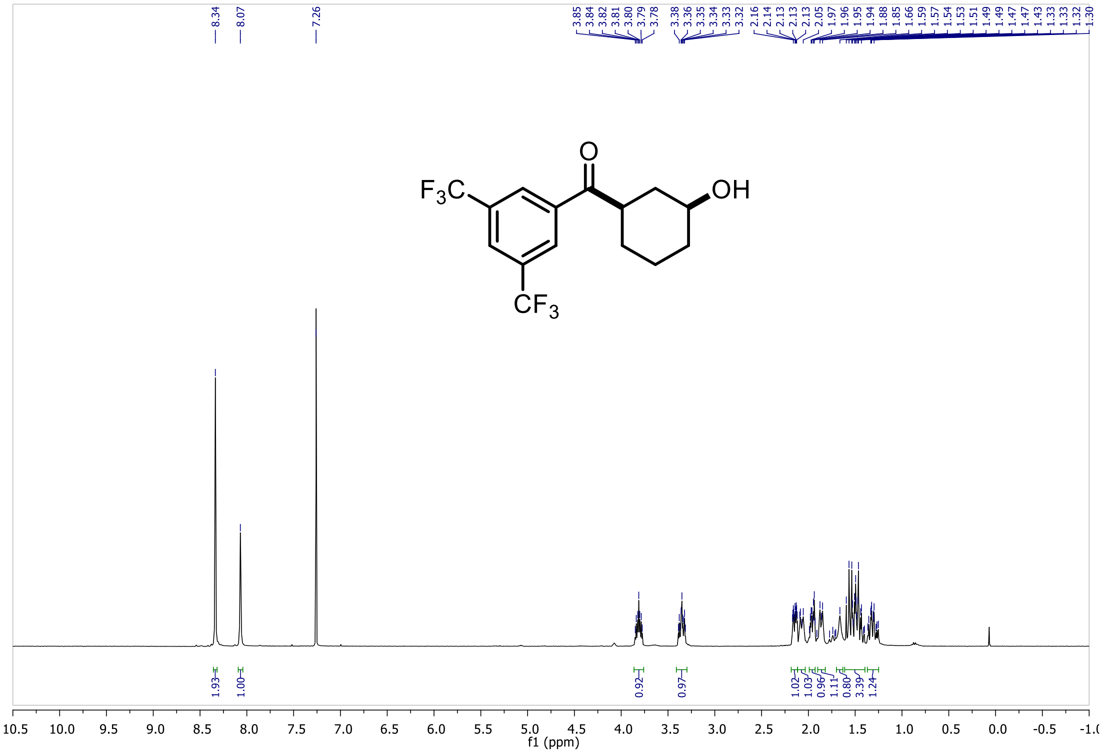

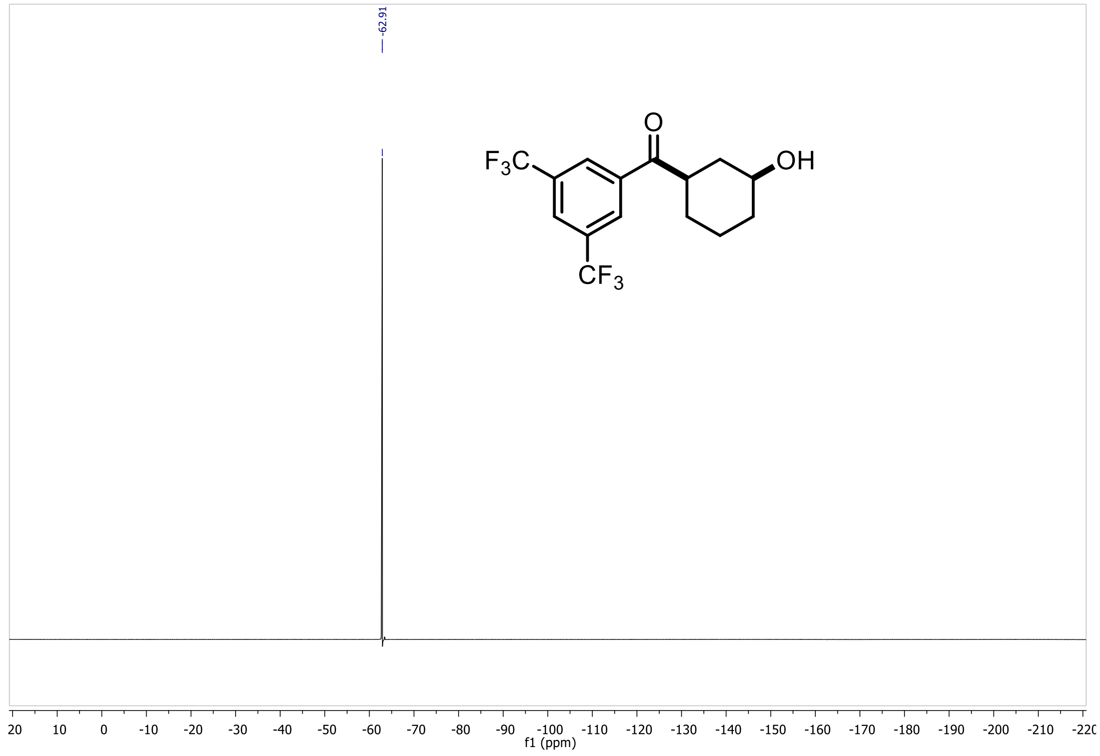

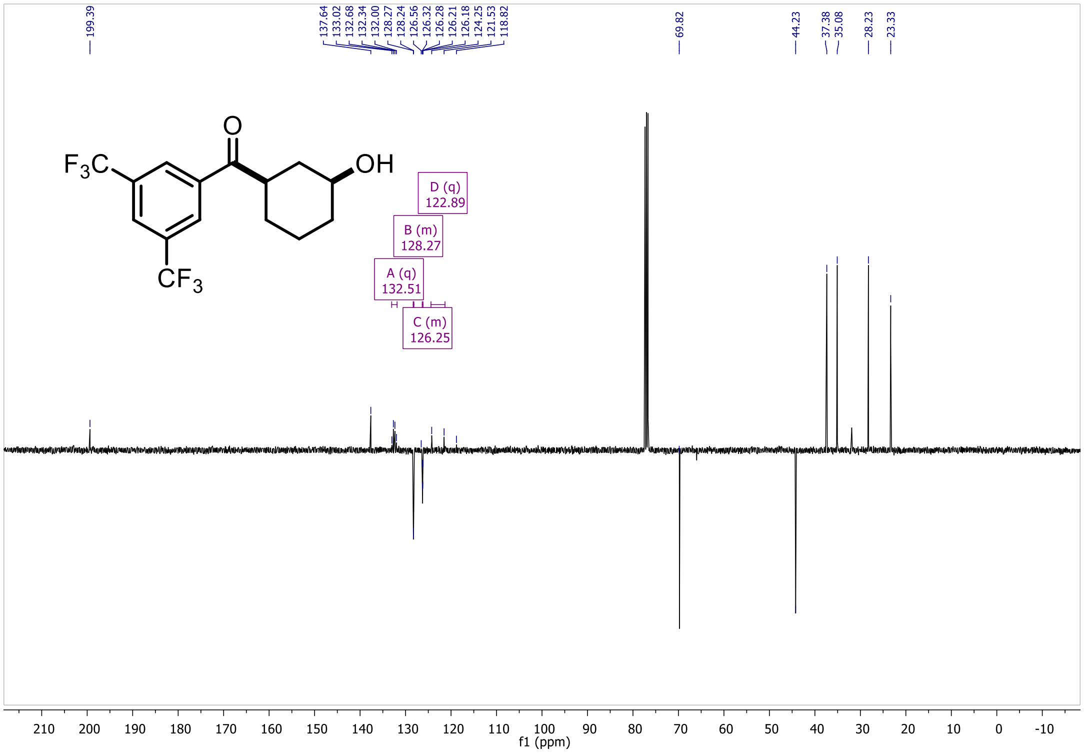
199.39
137.64
133.02
132.68
132.34
132.00
128.27
128.24
126.56
126.32
126.28
126.21
126.18
124.25
121.53
118.82
69.82
44.23
37.38
35.08
28.23
23.33
F3C
O
OH
CF3
D (q)
122.89
B (m)
128.27
A (q)
132.51
C (m)
126.25
210
200
190
180
170
160
150
140
130
120
110
100
90
80
70
60
50
40
30
20
10
0
-10
f1 (ppm)

***cis*-(3,5-Difluorophenyl)(3-hydroxycyclohexyl)methanone (2e)**

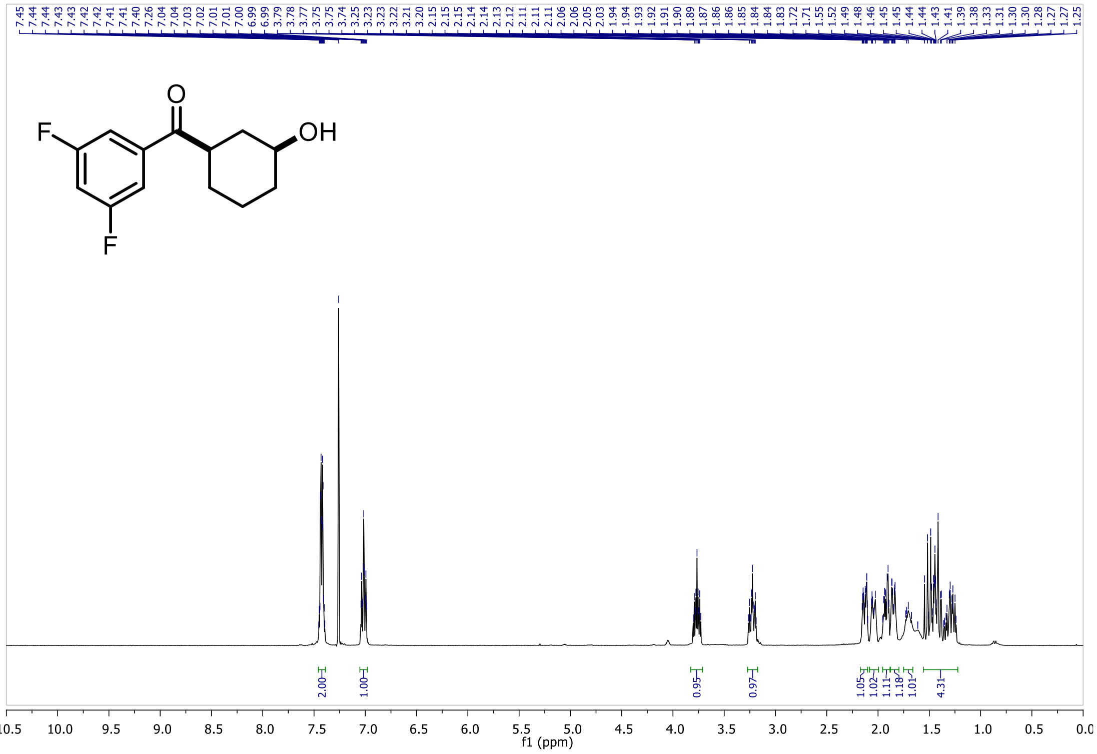

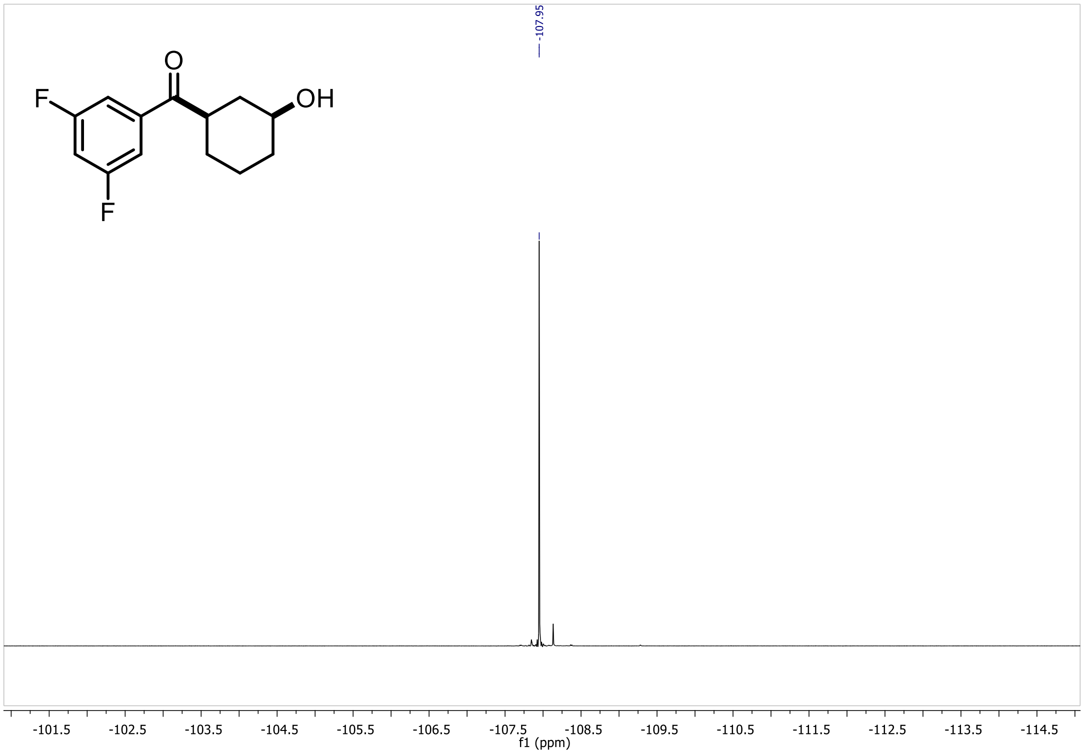

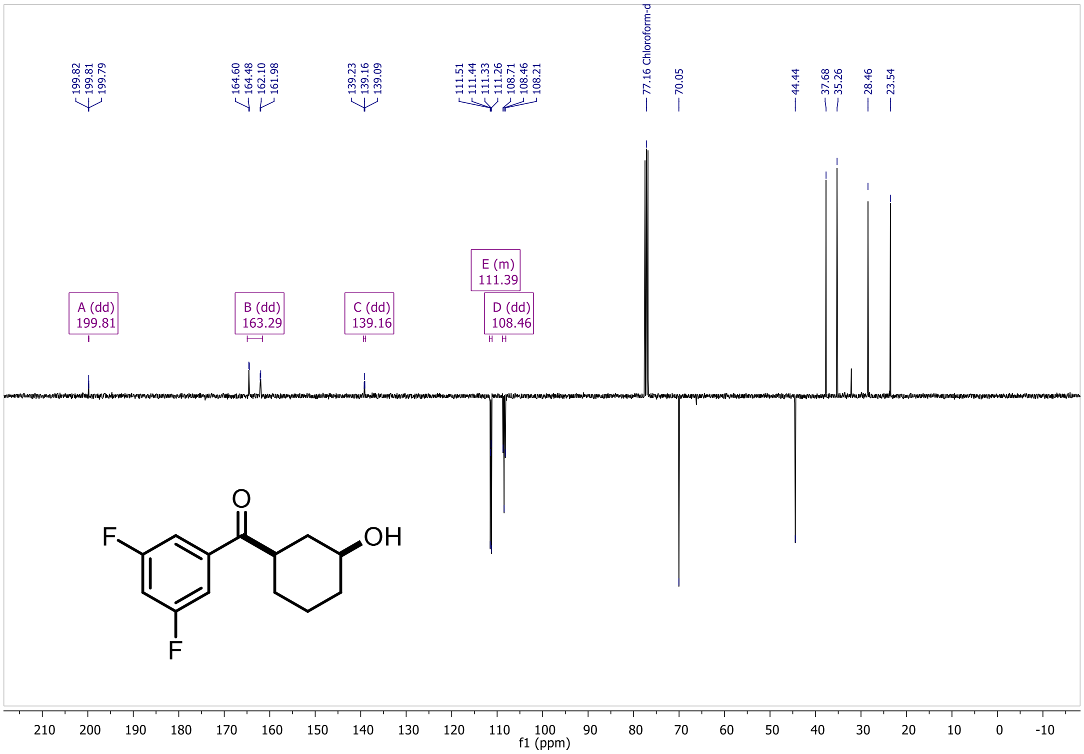
199.82
199.81
199.79
164.60
164.48
162.10
161.98
139.23
139.16
139.09
111.51
111.44
111.33
111.26
108.71
108.46
108.21
77.16 Chloroform-d
70.05
44.44
37.68
35.26
28.46
23.54
A (dd)
199.81
B (dd)
163.29
C (dd)
139.16
E (m)
111.39
D (dd)
108.46
O
F
OH
F
210 200 190 180 170 160 150 140 130 120 110 100 90 80 70 60 50 40 30 20 10 0 -10
f1 (ppm)

cis-(3-Hydroxycyclohexyl)(4-(trifluoromethyl)phenyl)methanone (2f)

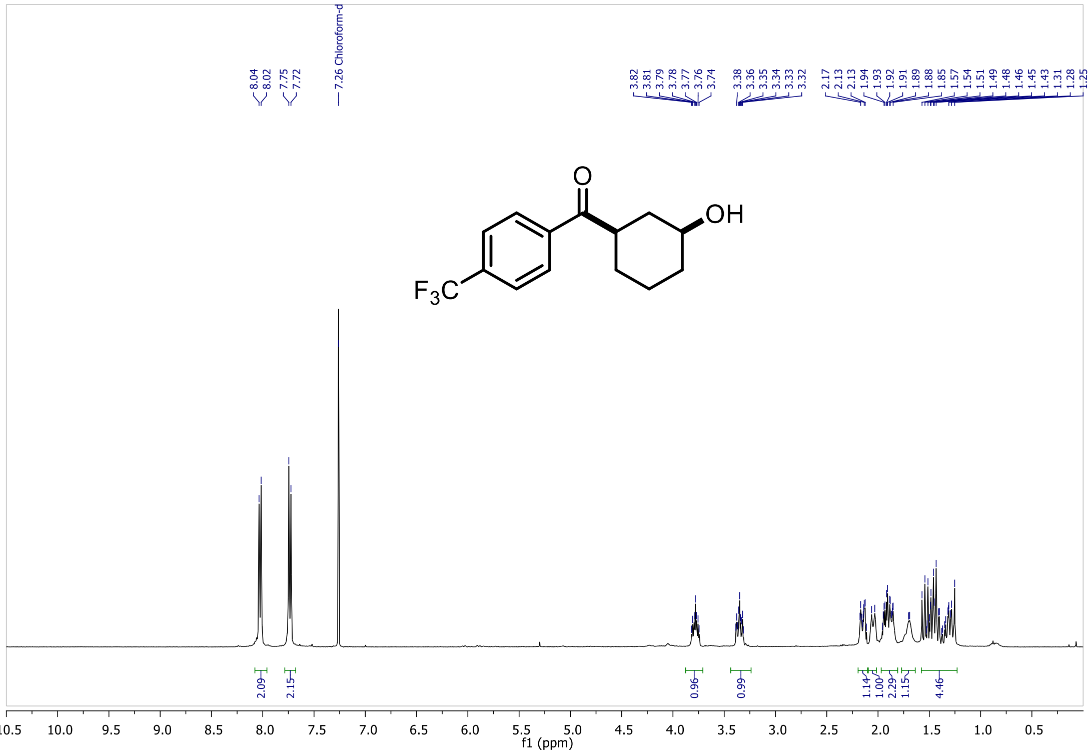

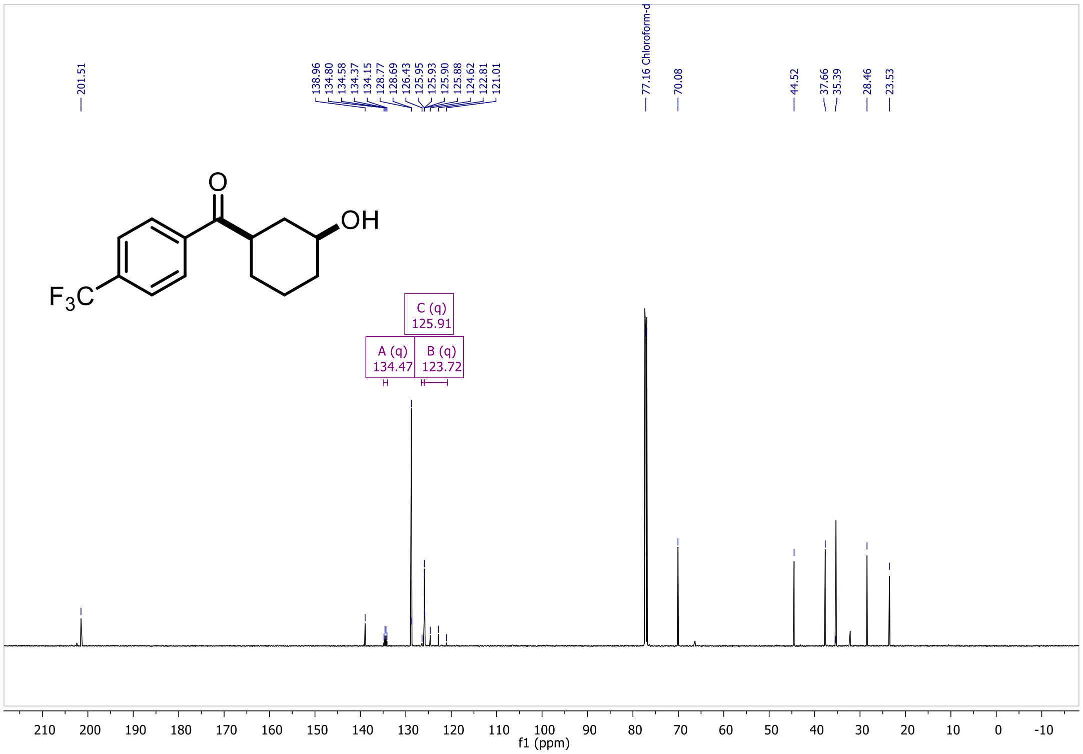

***cis*-(3,4-Dichlorophenyl)(3-hydroxycyclohexyl)methanone (2g)**

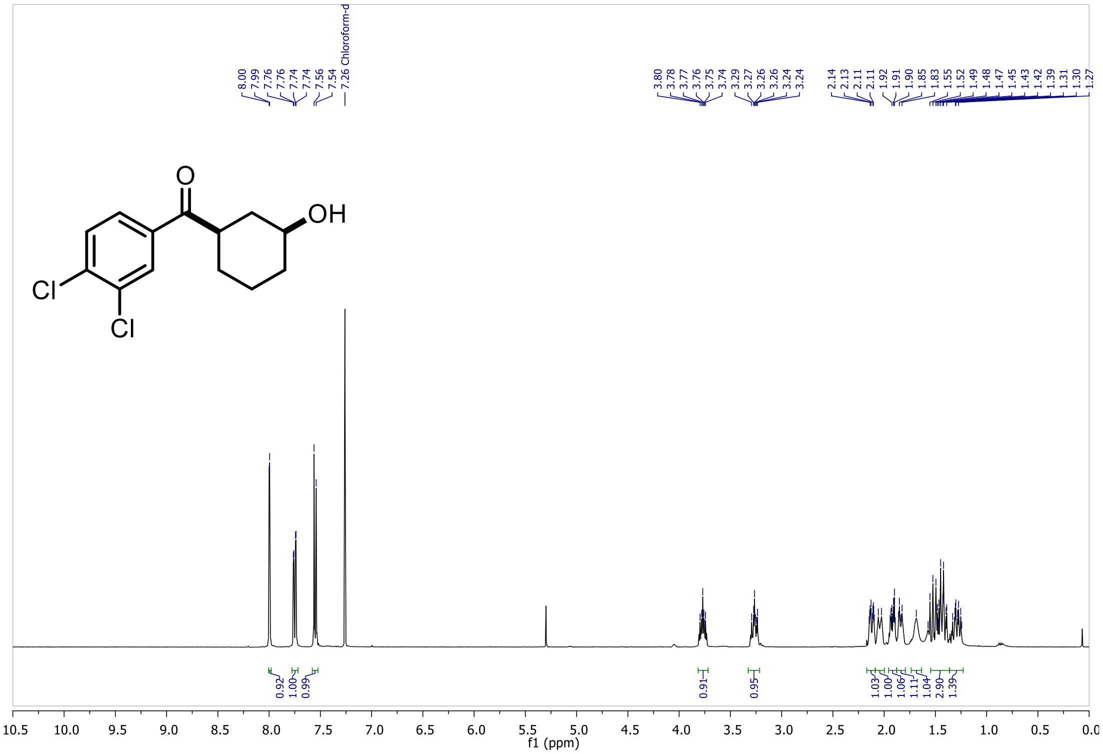

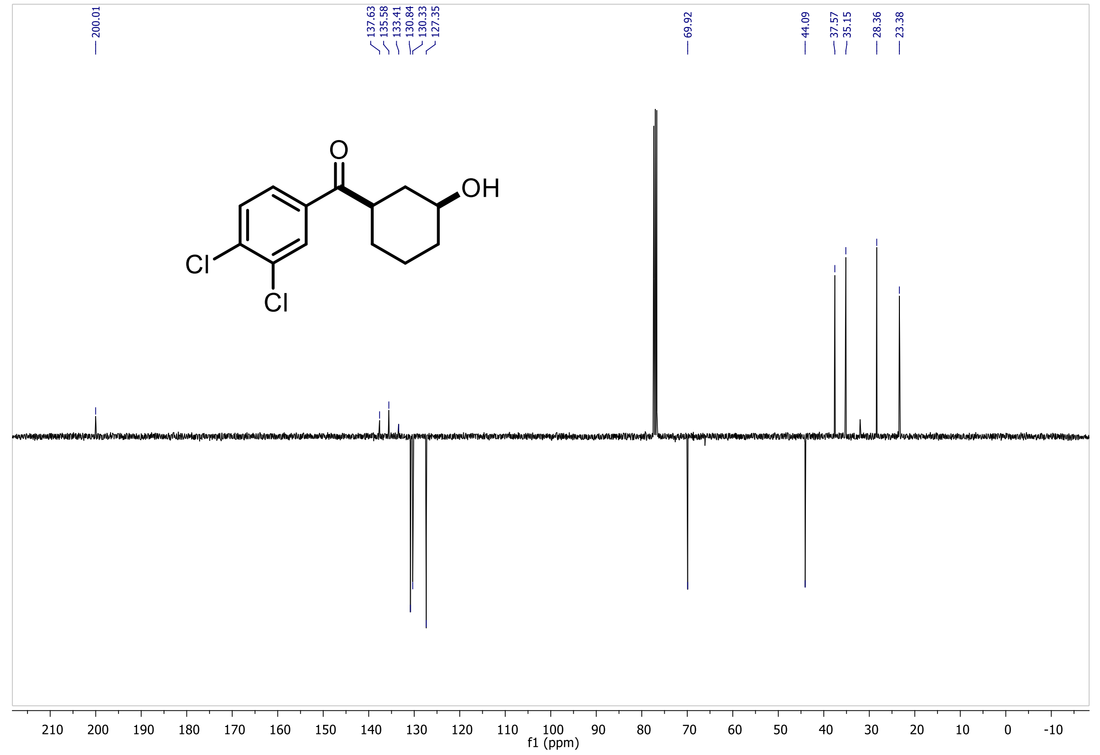

***cis*-(3-Chlorophenyl)(3-hydroxycyclohexyl)methanone (2h)**

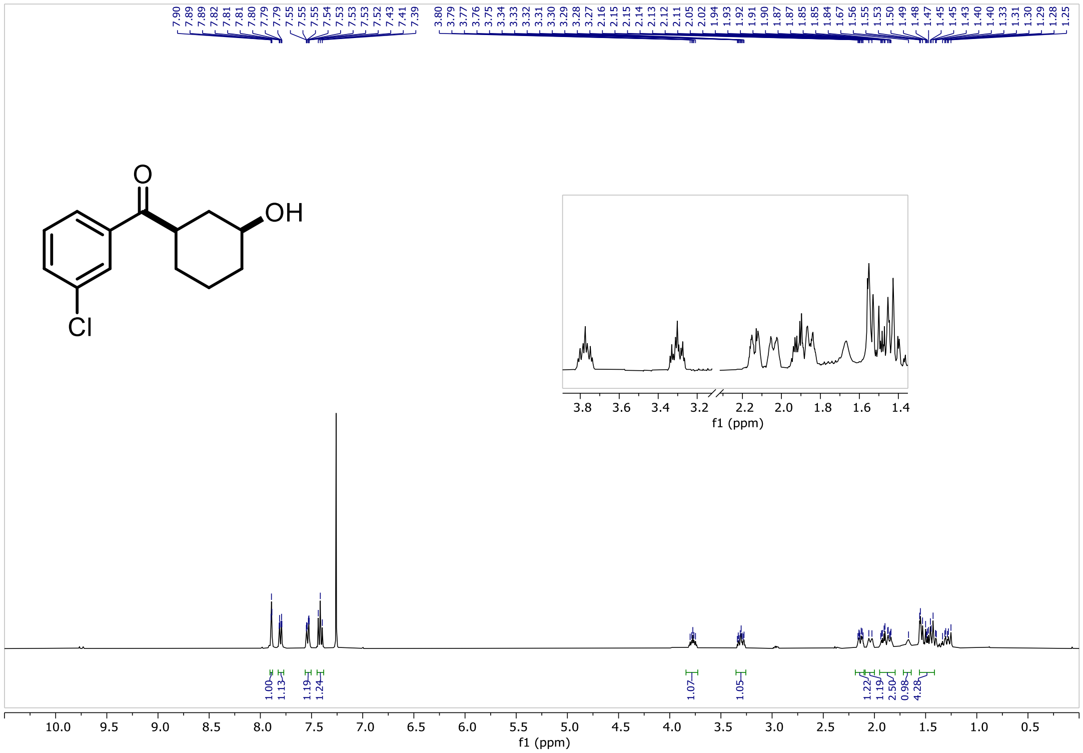

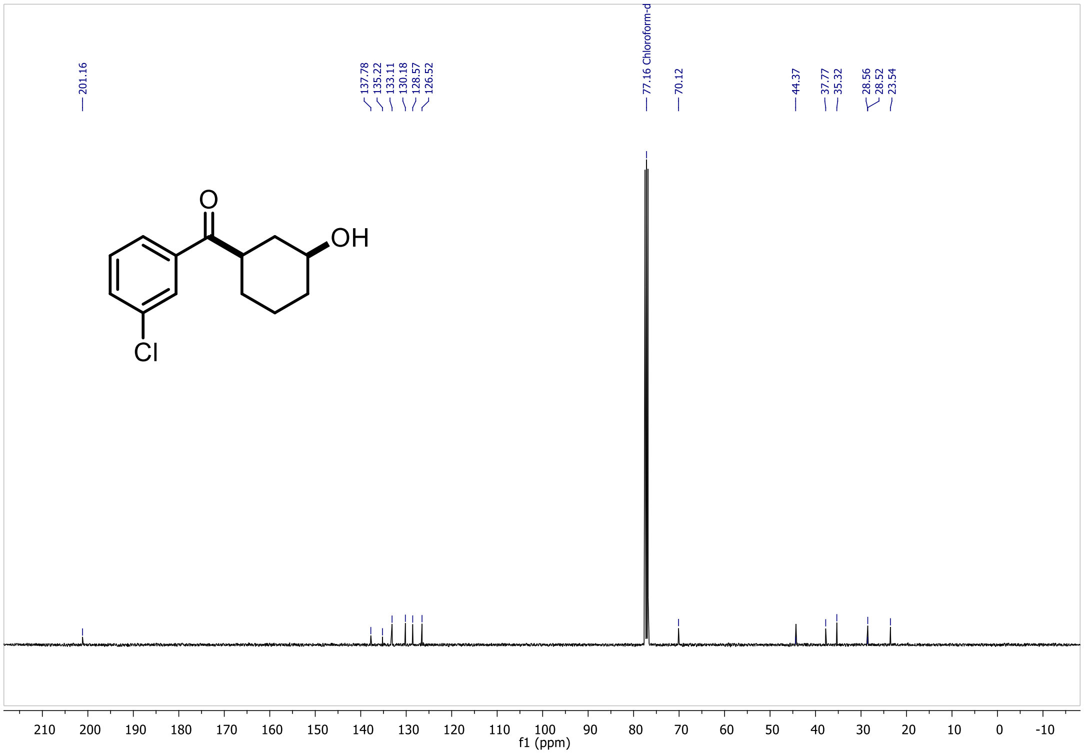

***cis*-(2-Bromophenyl)(3-hydroxycyclohexyl)methanone (2i)**

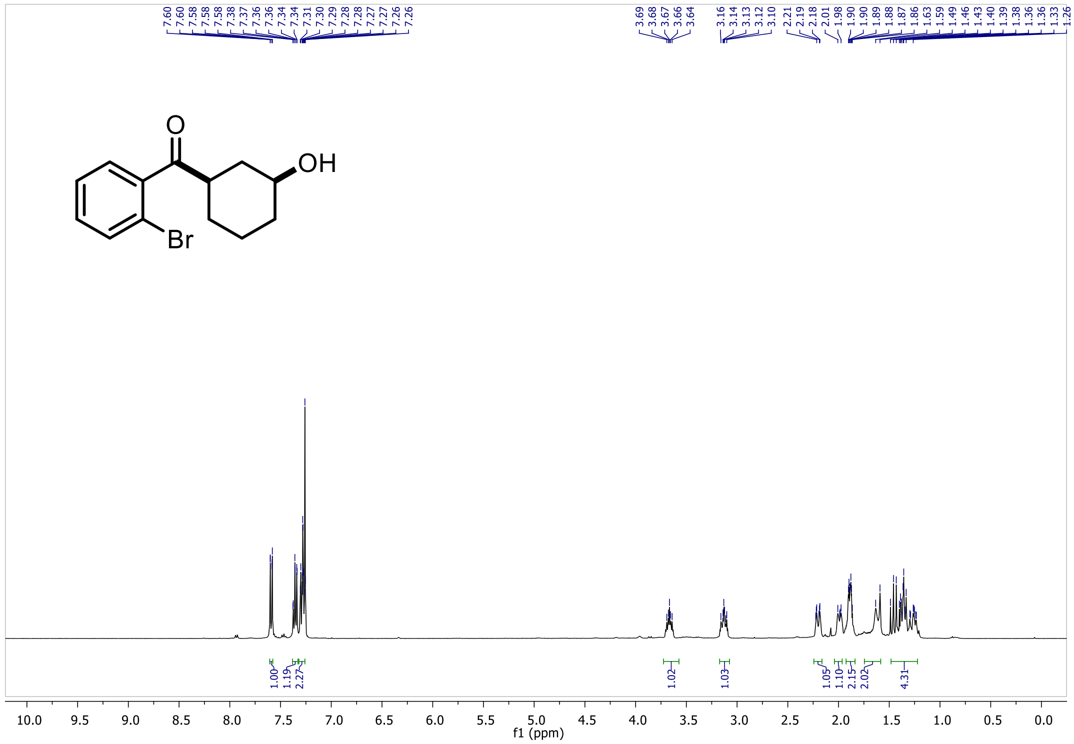

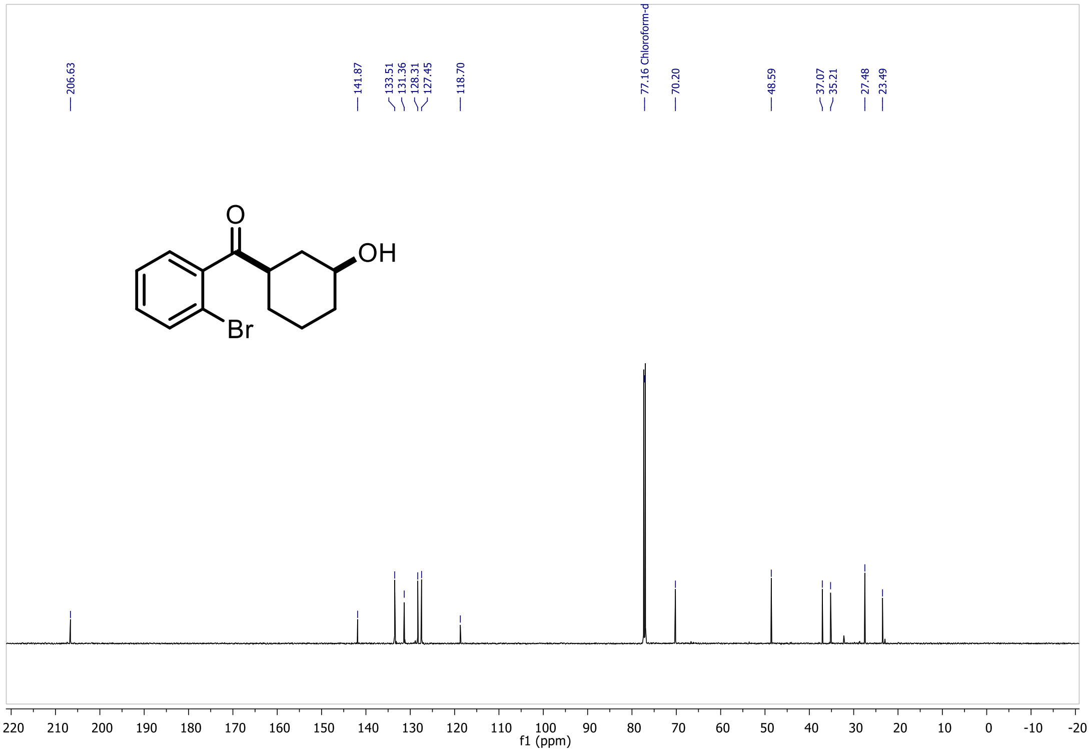

cis-(4-(_tert_-Butyl)phenyl)(3-hydroxycyclohexyl)methanone (2j)

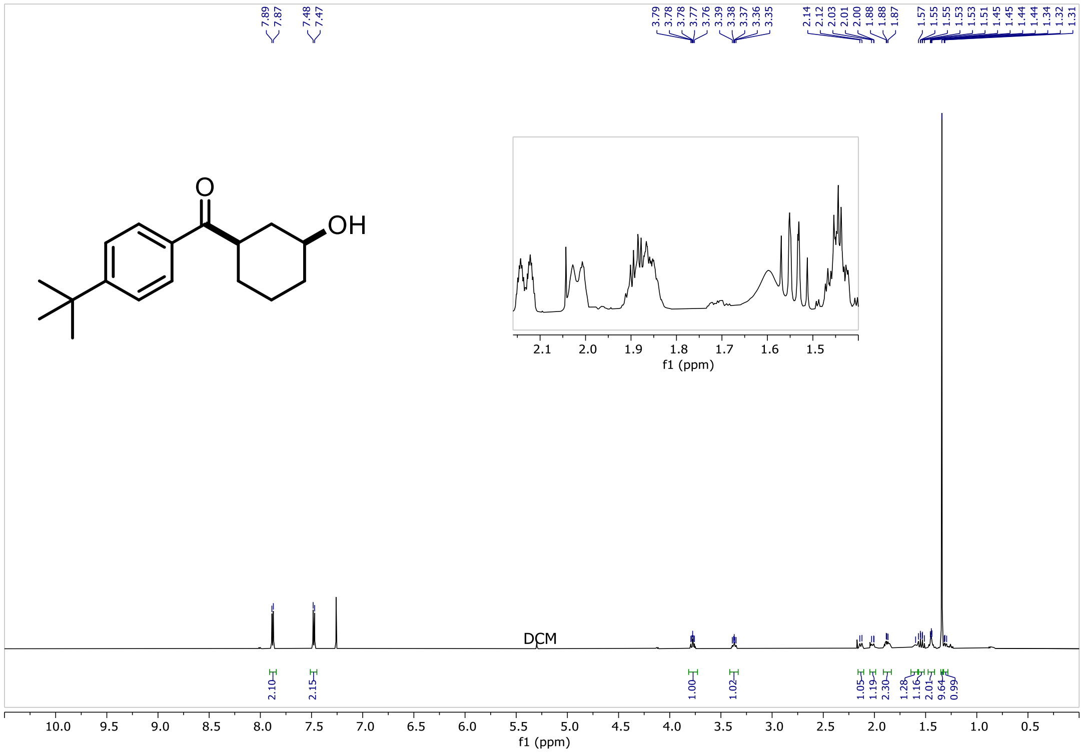

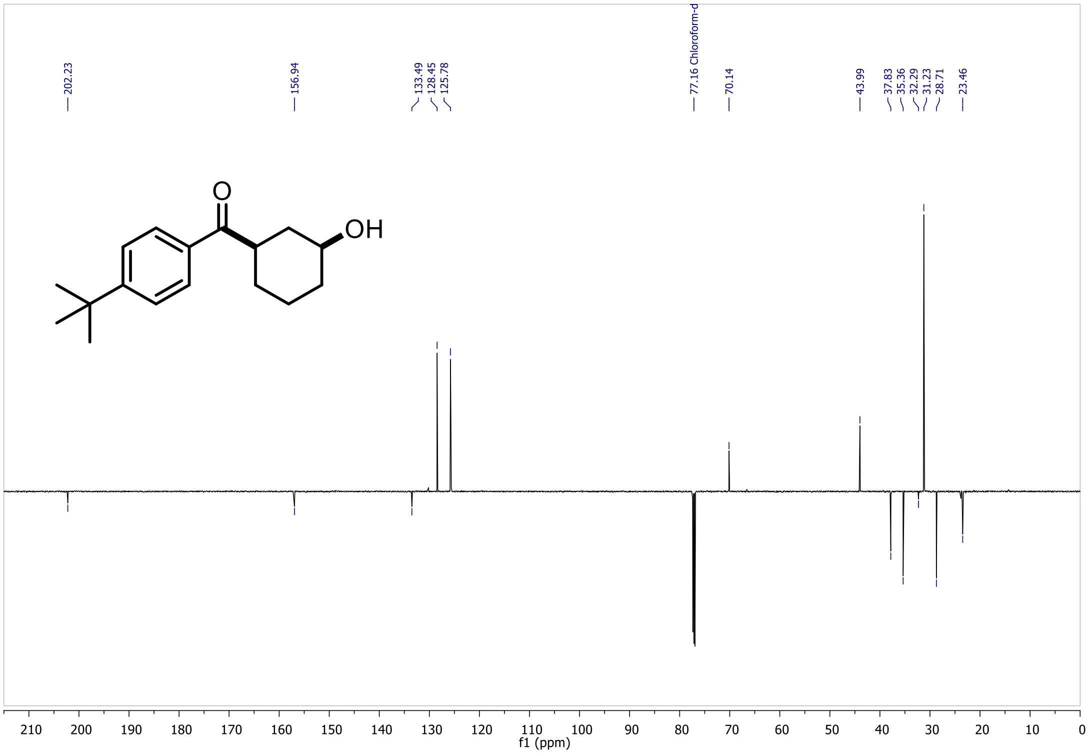

***cis*-4-(3-Hydroxycyclohexane-1-carbonyl)benzonitrile (2k)**

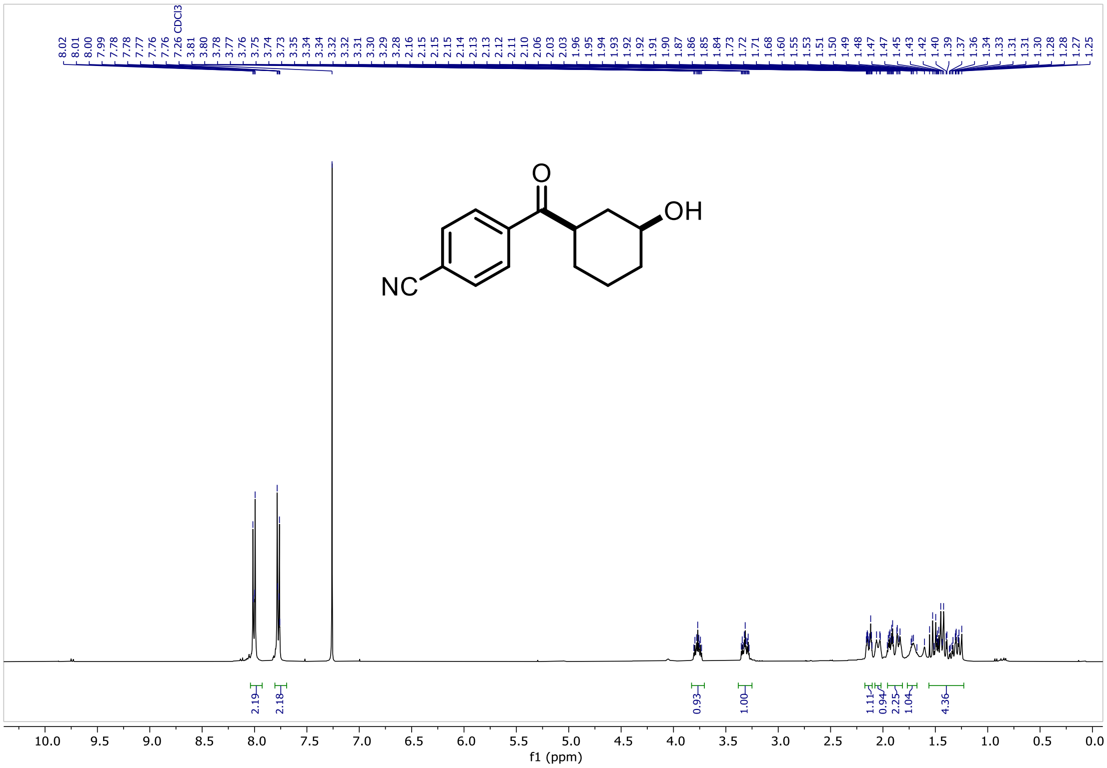

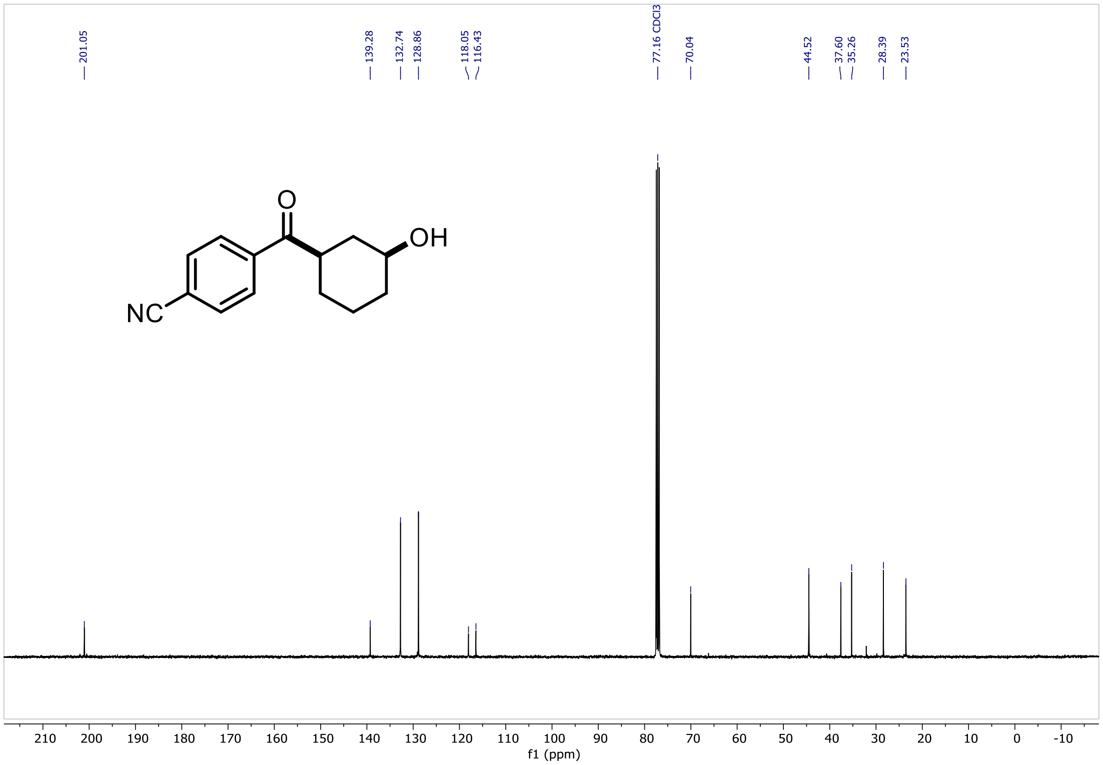

cis-Methyl 4-(3-hydroxycyclohexane-1-carbonyl)benzoate (2l)

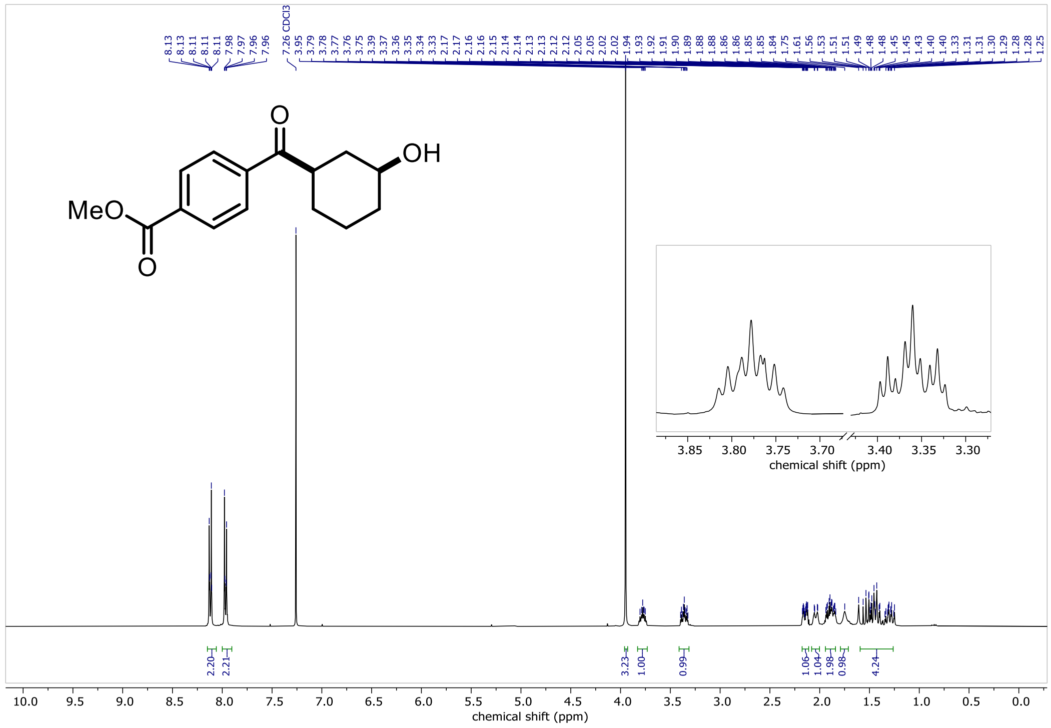

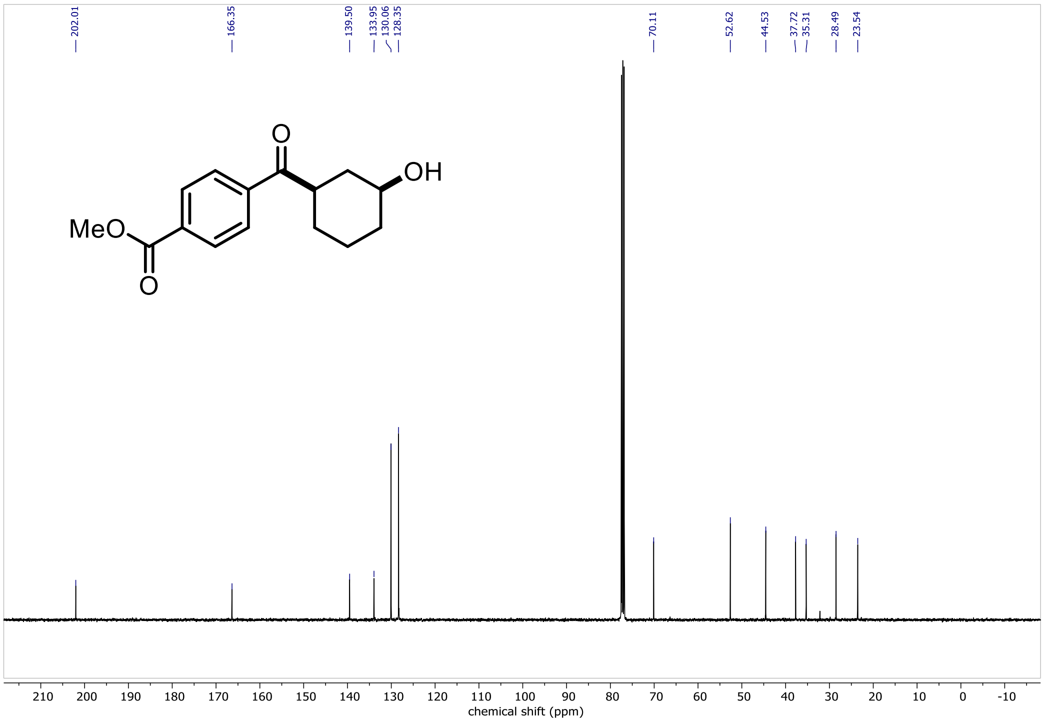

cis-(3-Hydroxycyclohexyl)(4-(trifluoromethoxy)phenyl)methanone (2m)

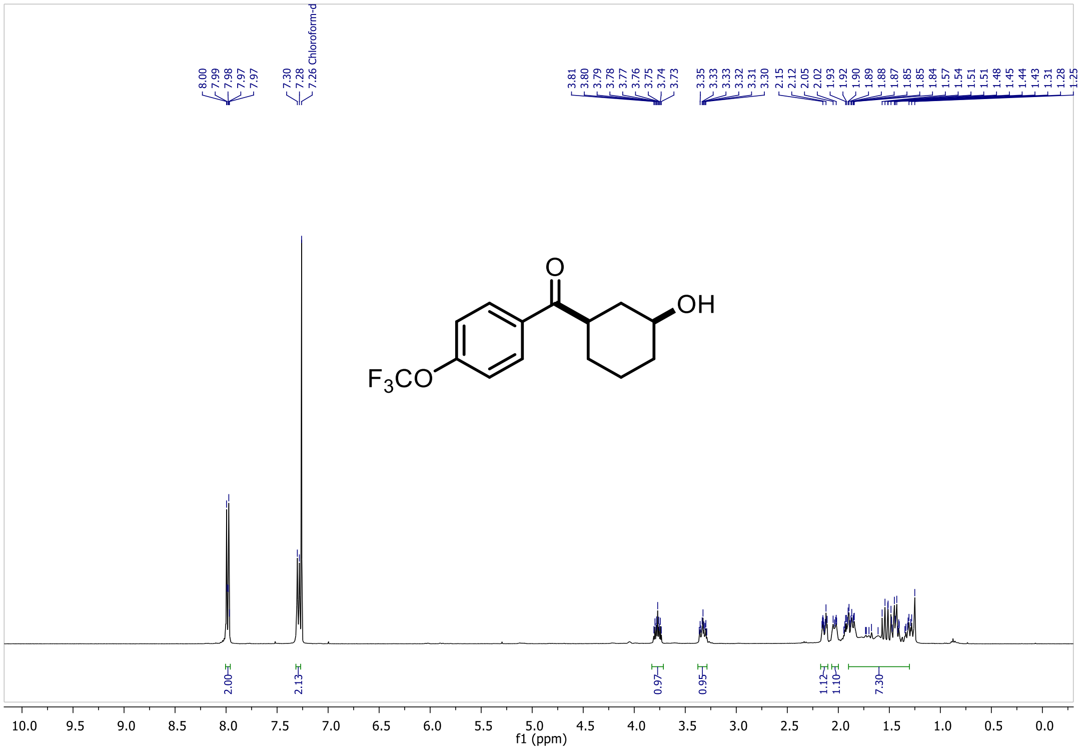

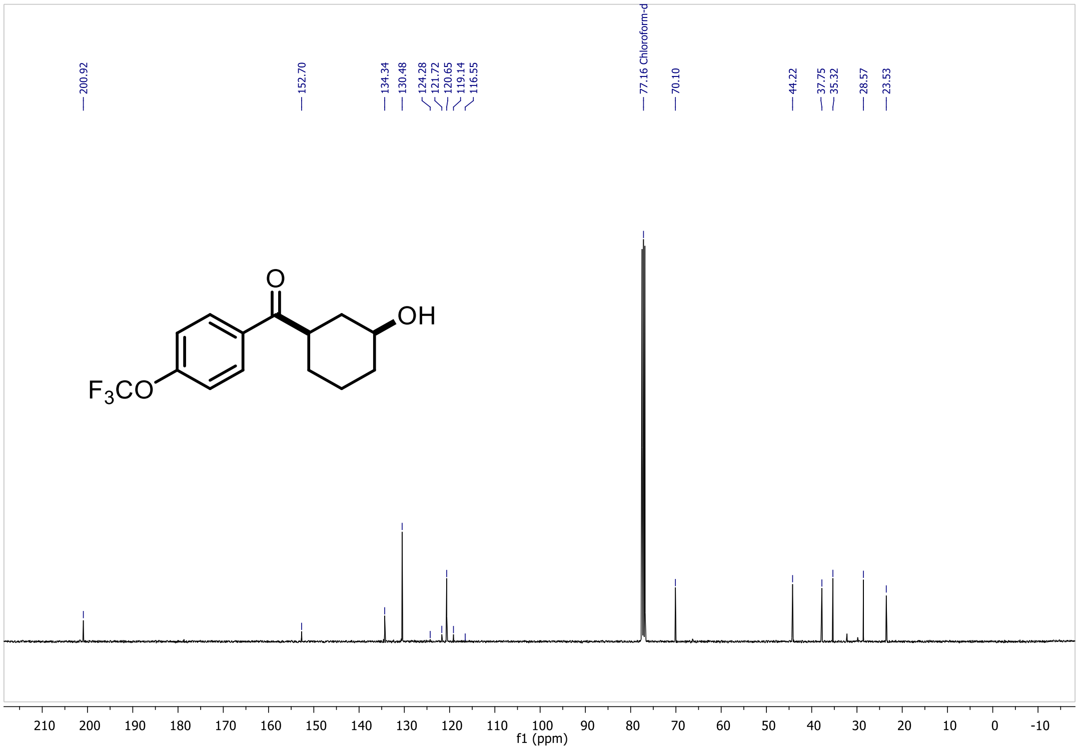

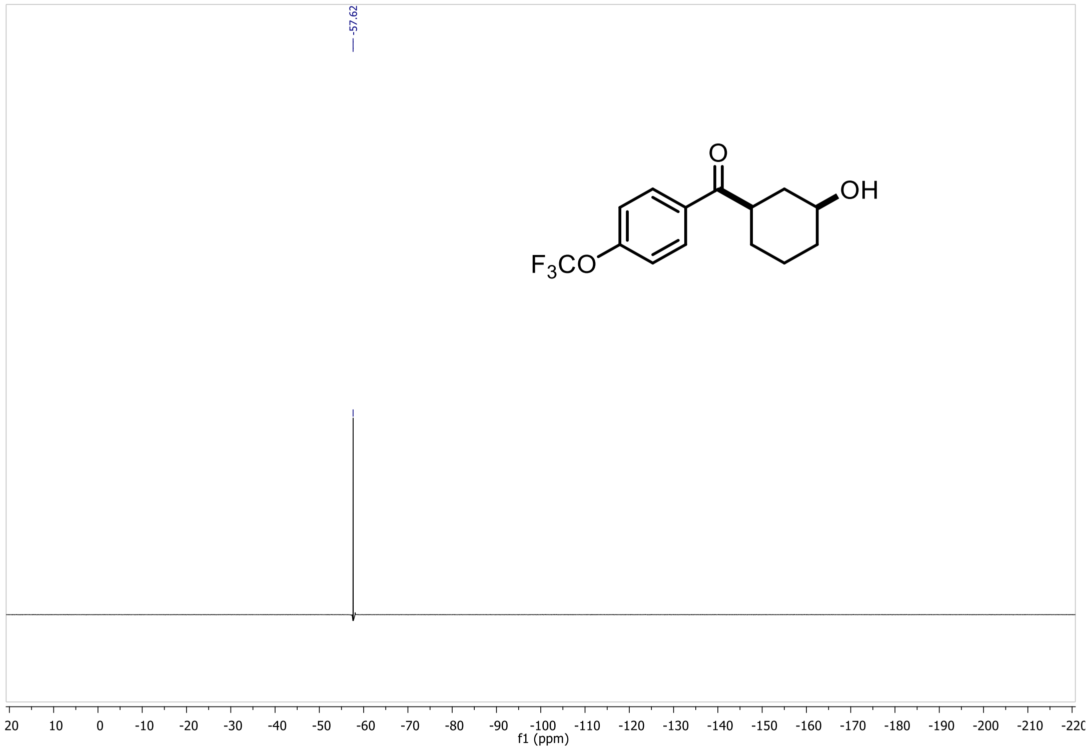

-57.62
F3CO
O
OH
20 10 0 -10 -20 -30 -40 -50 -60 -70 -80 -90 -100 -110 -120 -130 -140 -150 -160 -170 -180 -190 -200 -210 -220
f1 (ppm)

cis-(3-Hydroxycyclohexyl)(thiophen-2-yl)methanone (2n)

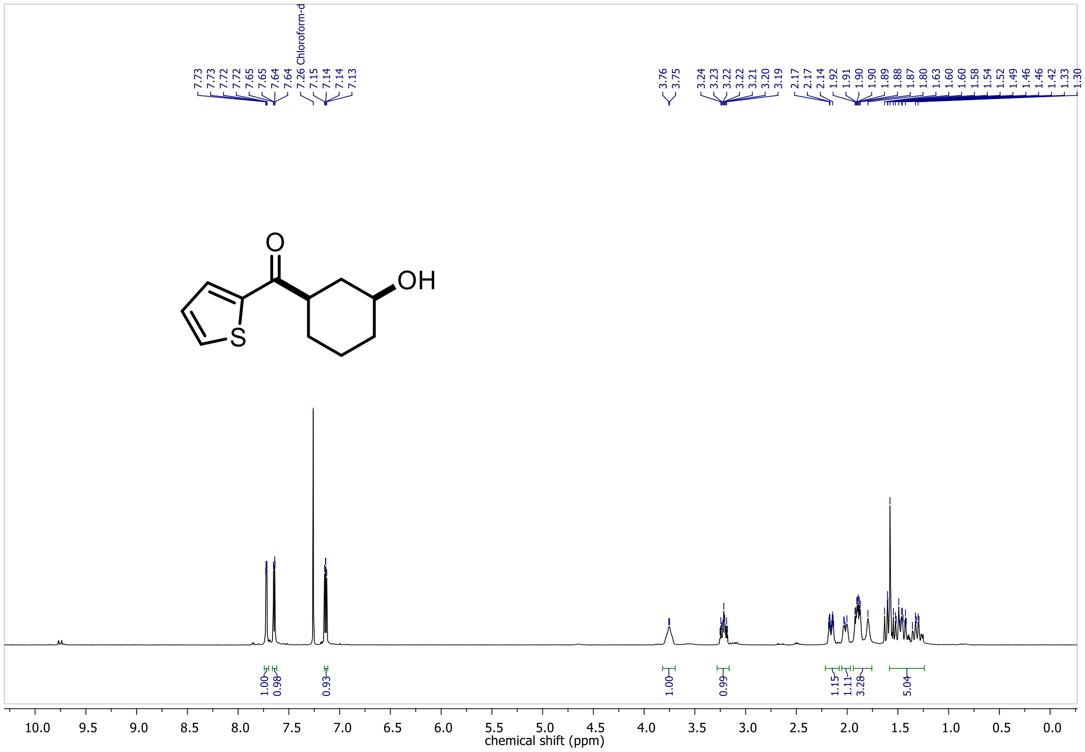

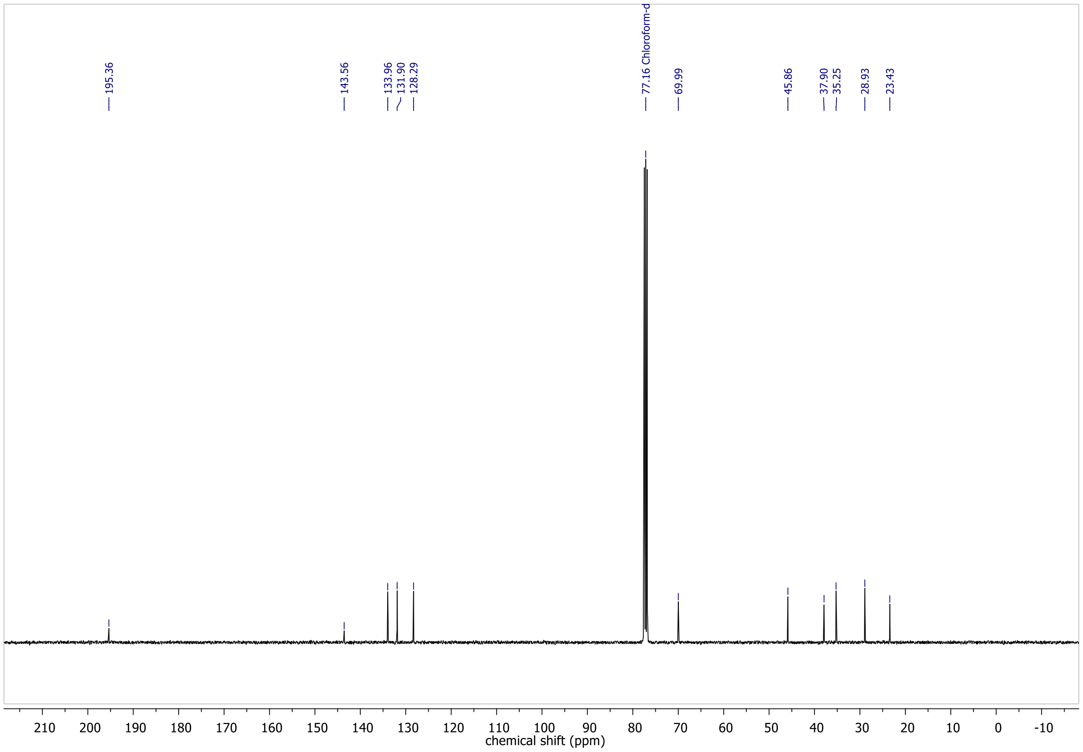

cis-Furan-2-yl(3-hydroxycyclohexyl)methanone (2o)

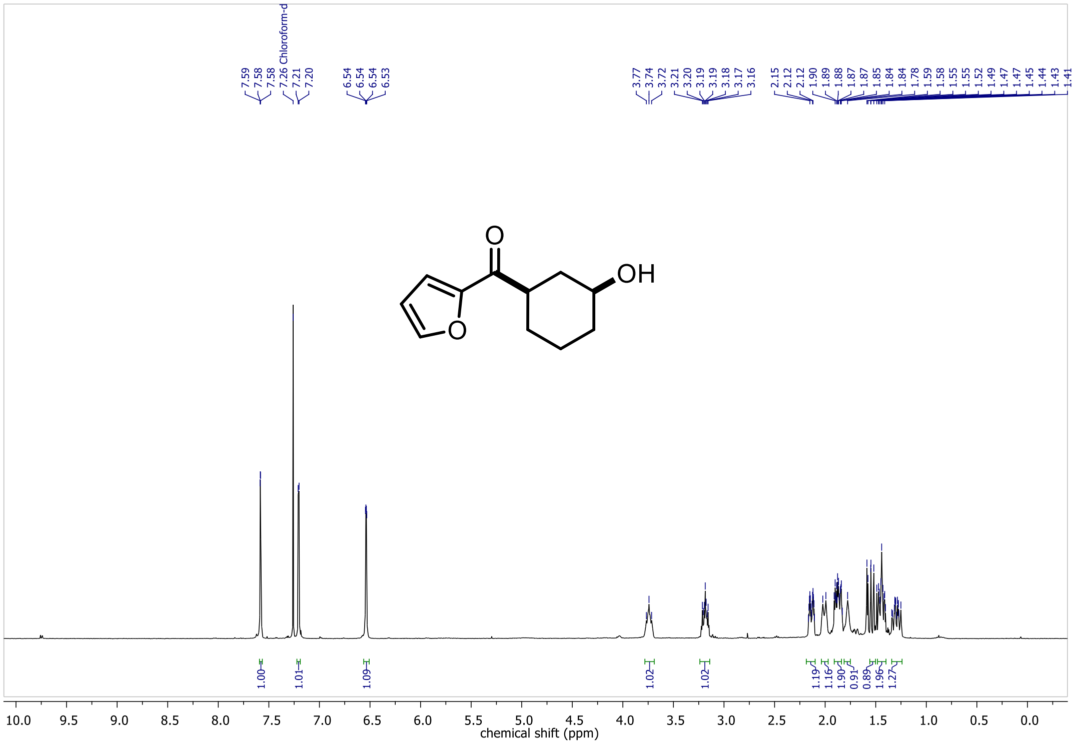

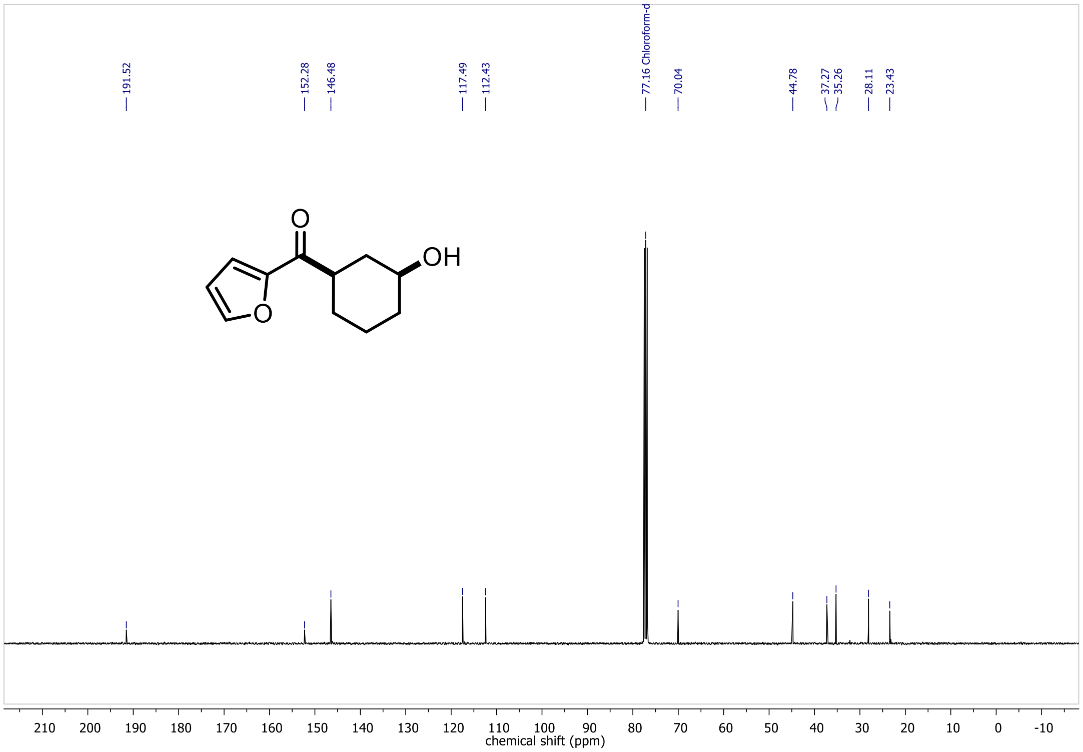

cis-1-(-3-Hydroxycyclohexyl)-2,2-dimethylpropan-1-one (2p)

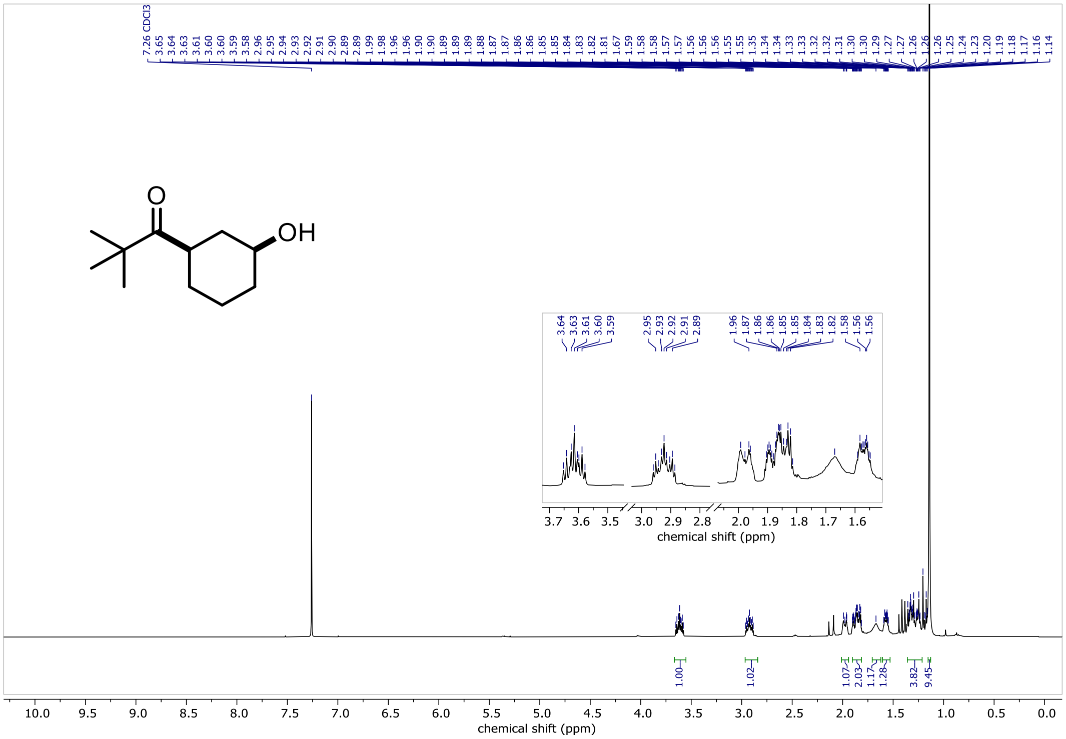

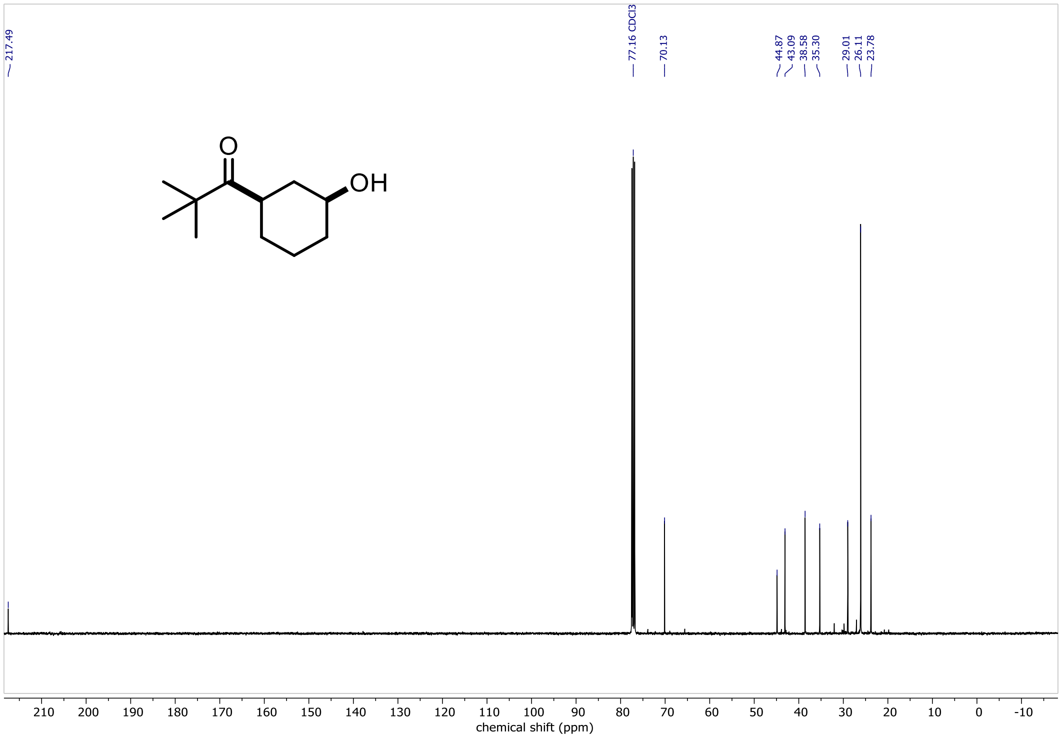

cis-Adamantan-1-yl-(3-hydroxycyclohexyl)methanone (2q)

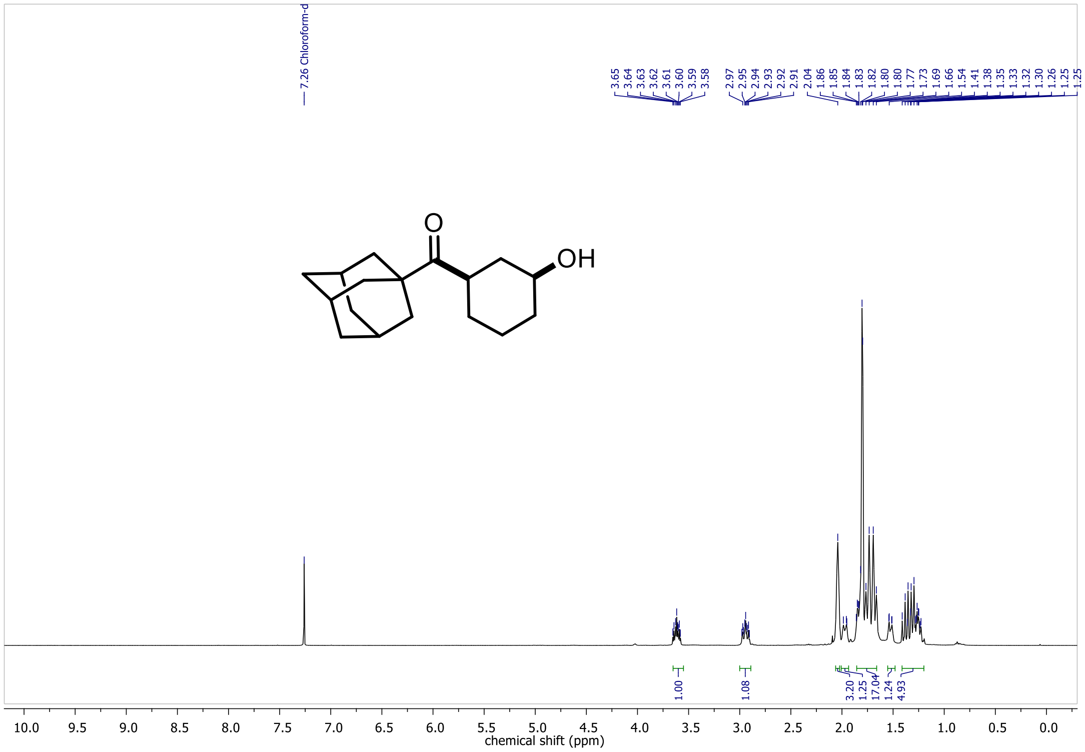

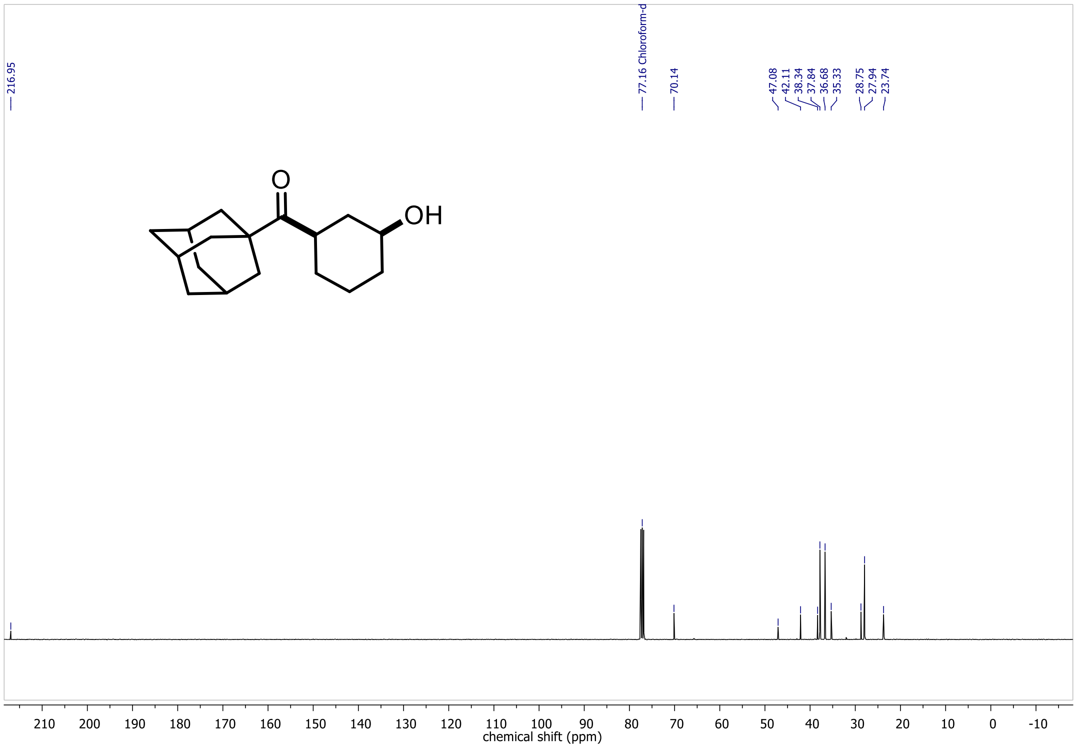

***cis*-cyclohexyl((1R,3S)-3-hydroxycyclohexyl)methanone** (2r)

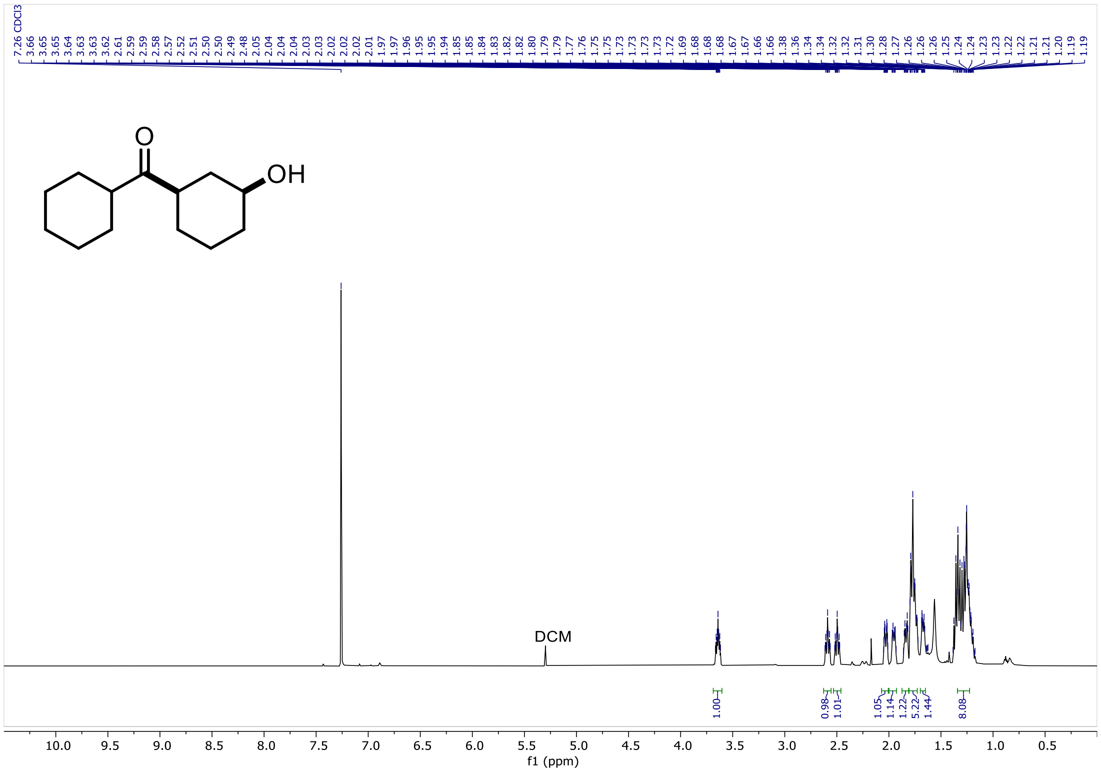

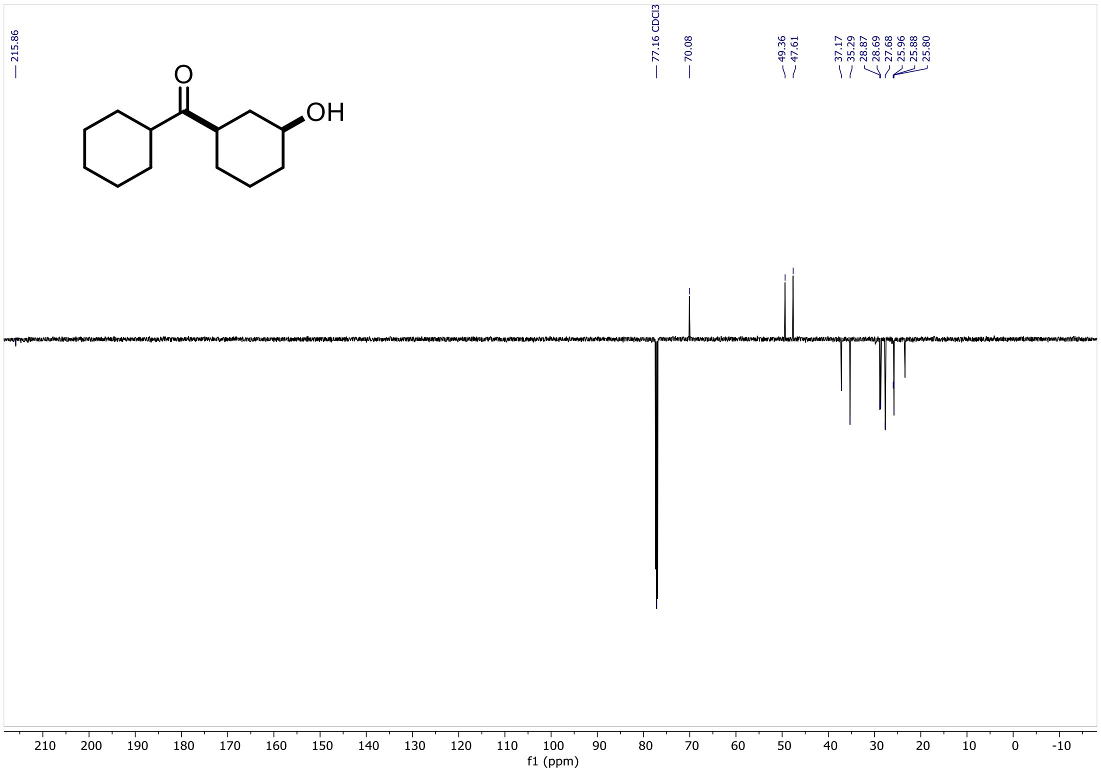

cis-3-Hydroxy-3-methylcyclohexyl)(phenyl)methanone (2s)

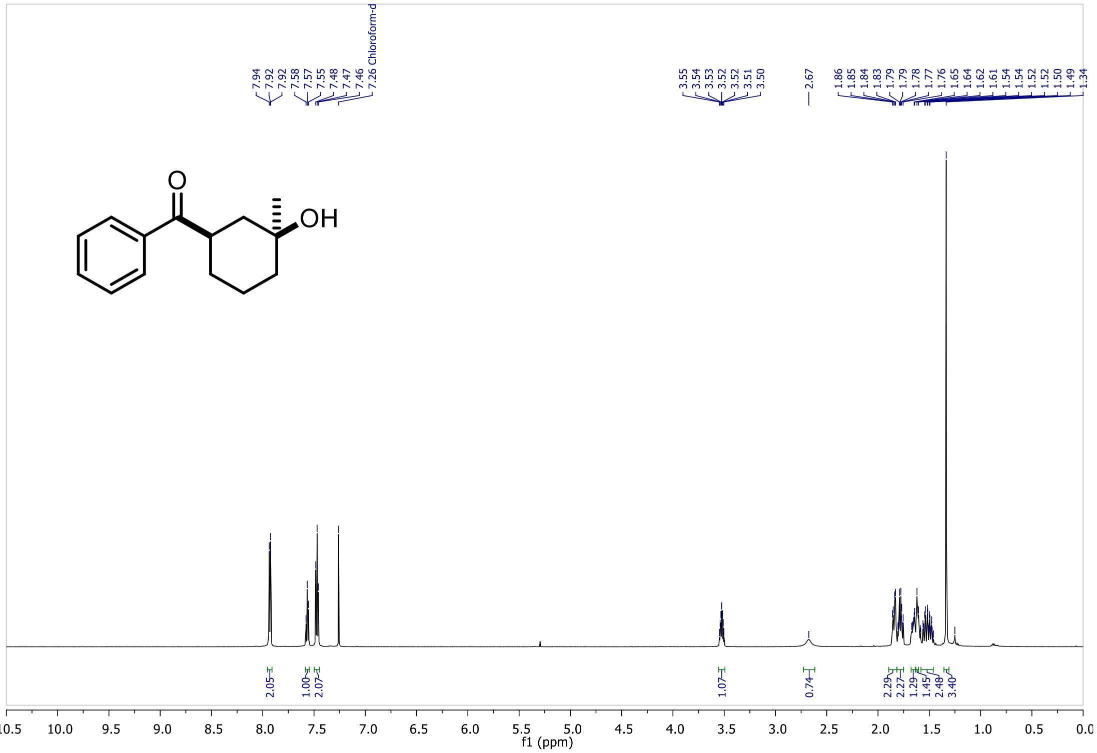

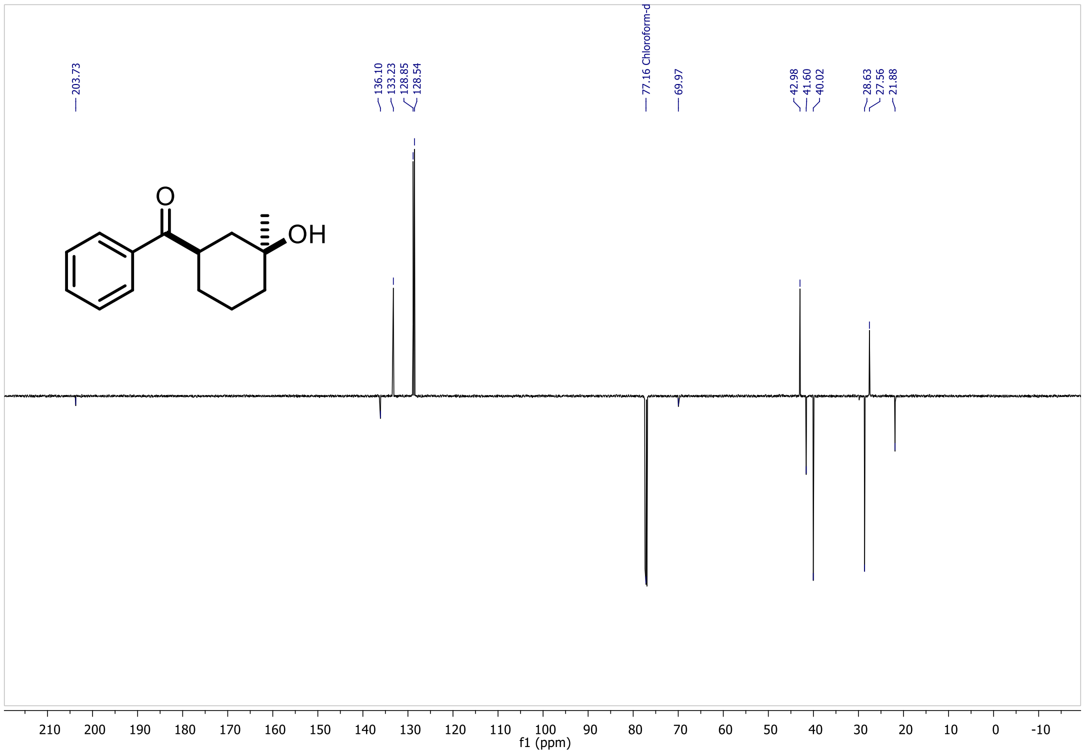

***cis*-3-Hydroxy-3-methylcyclohexyl)(phenyl)methanone (2s) – NOESY**

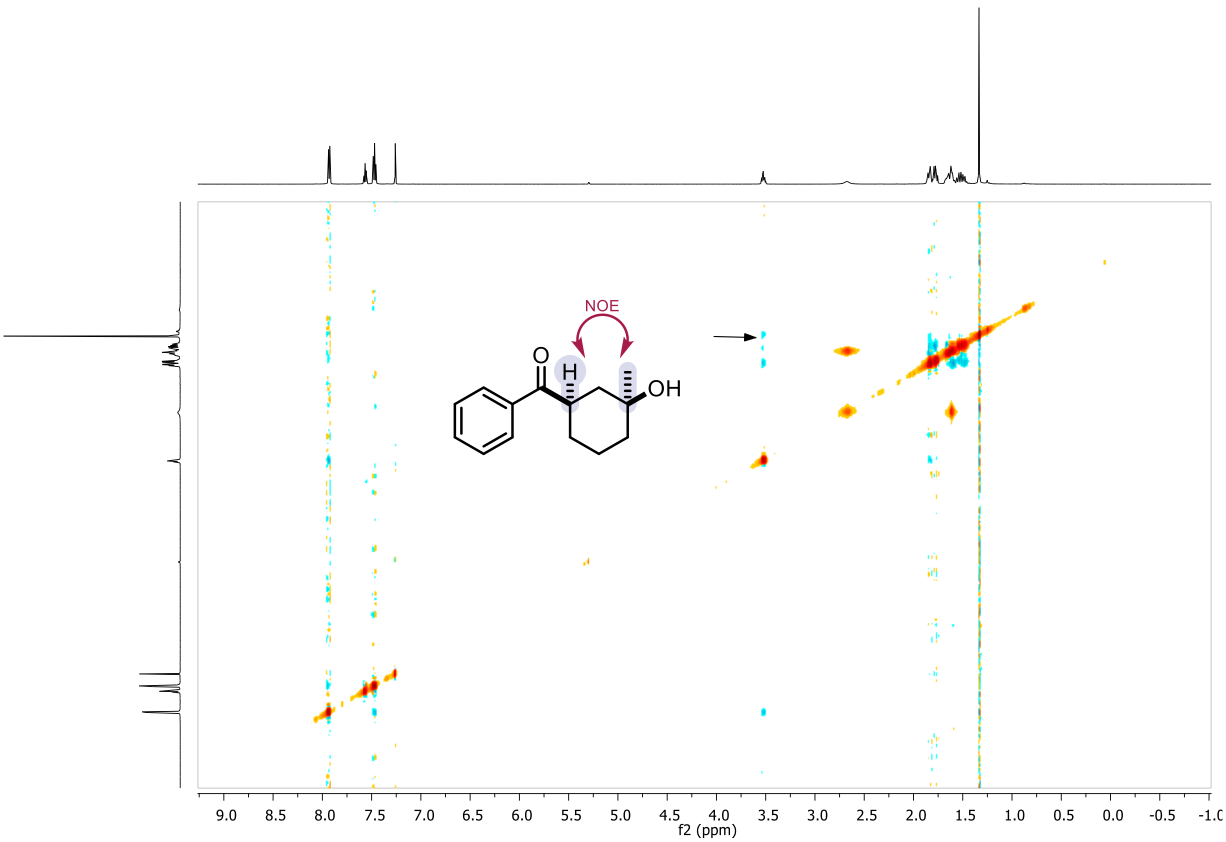

cis-(3-Hydroxy-3-phenylcyclohexyl)(phenyl)methanone (2t)

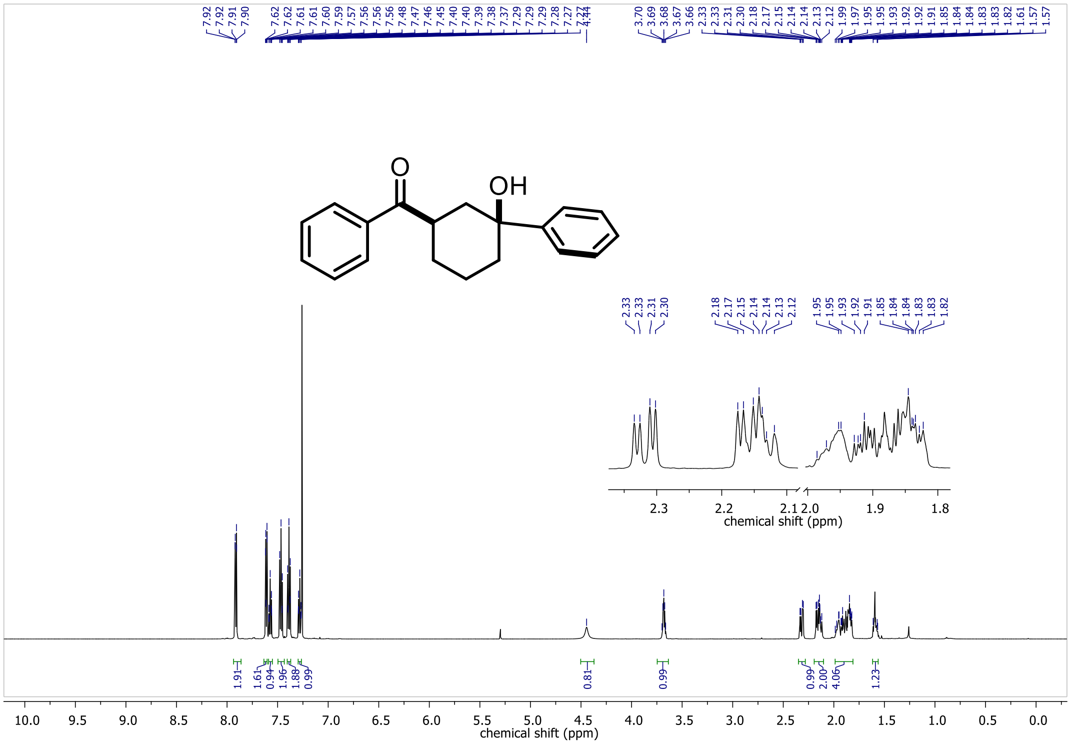

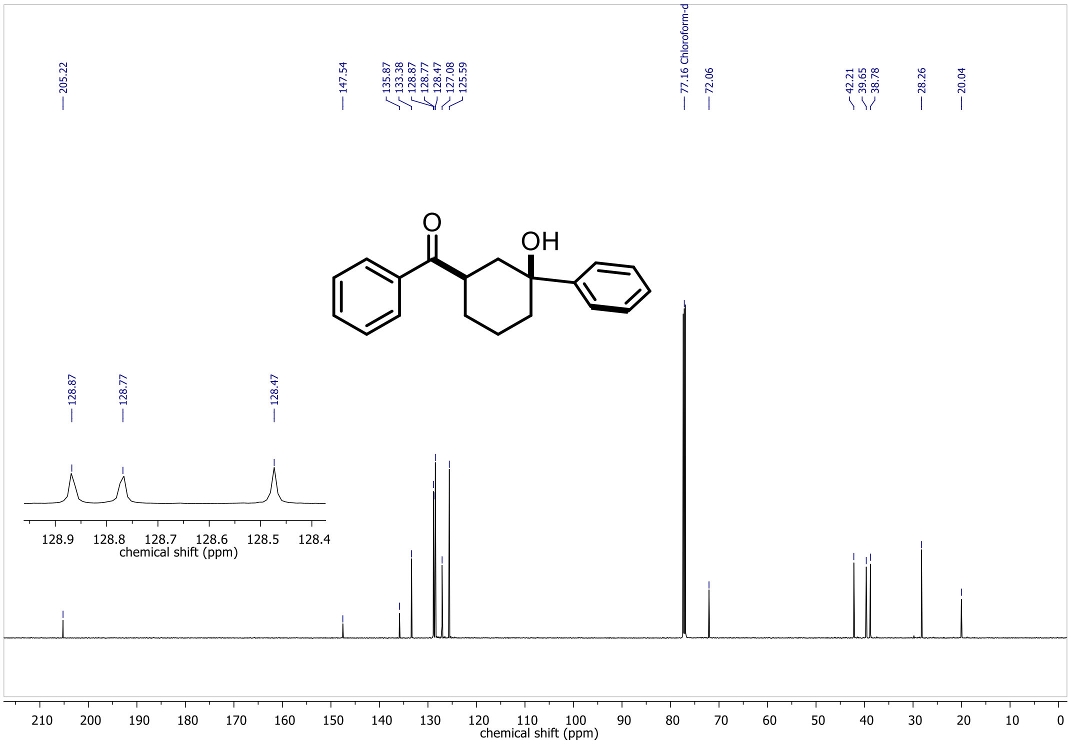

cis-3-Hydroxy-3-(naphthalen-2-yl)cyclohexyl)(phenyl)methanone (2u)

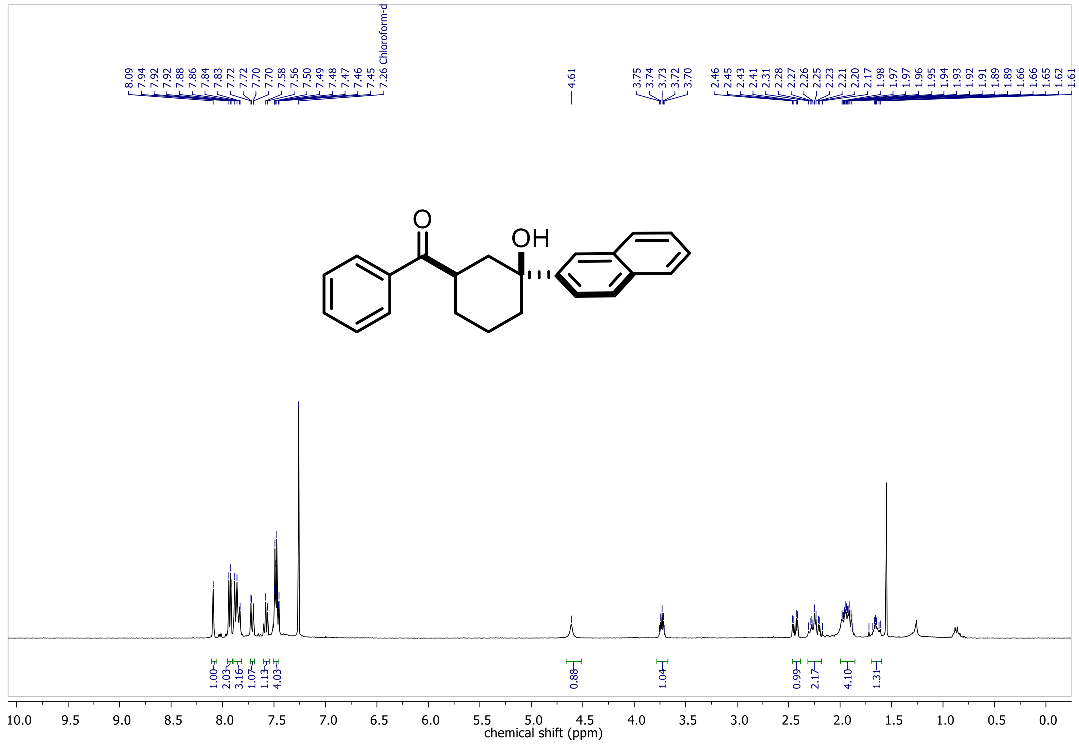

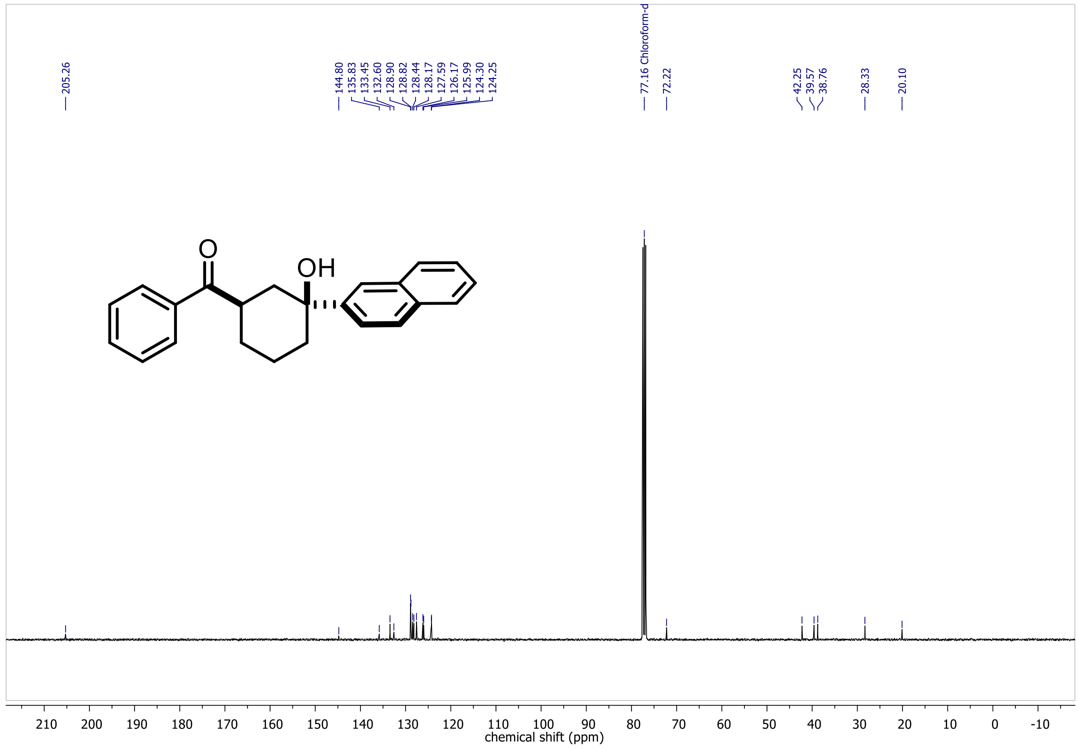

***cis*-3-(Dibenzo[*b,d*]furan-2-yl)-3-hydroxycyclohexyl)(phenyl)methanone (2v)**

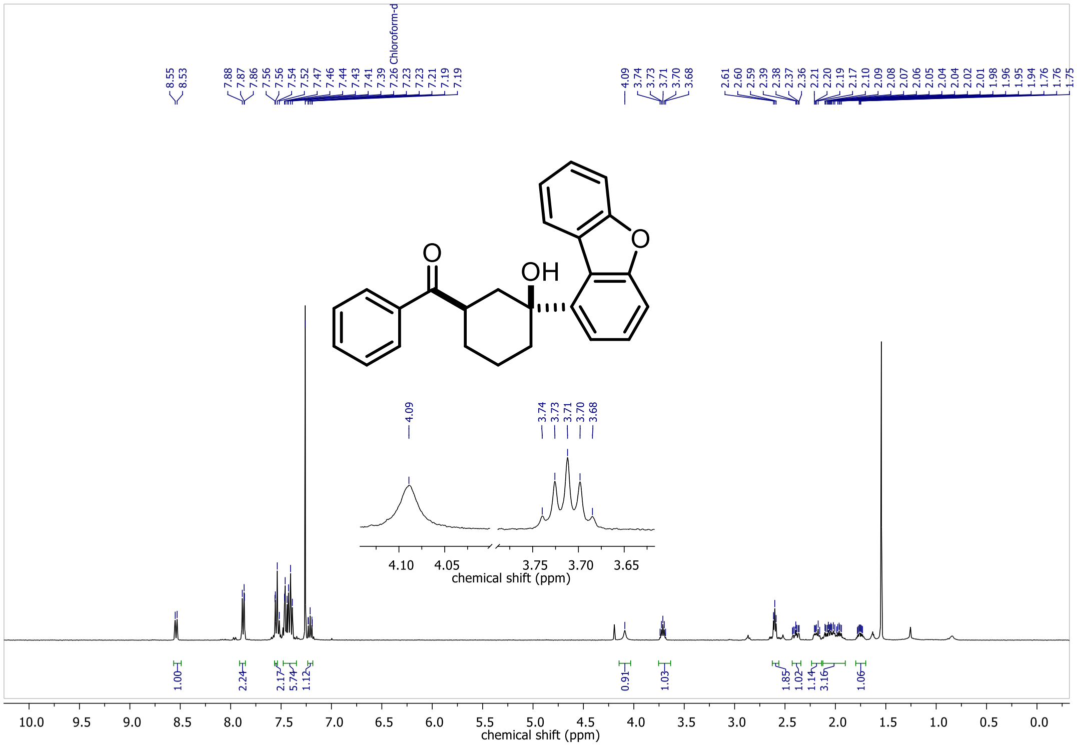

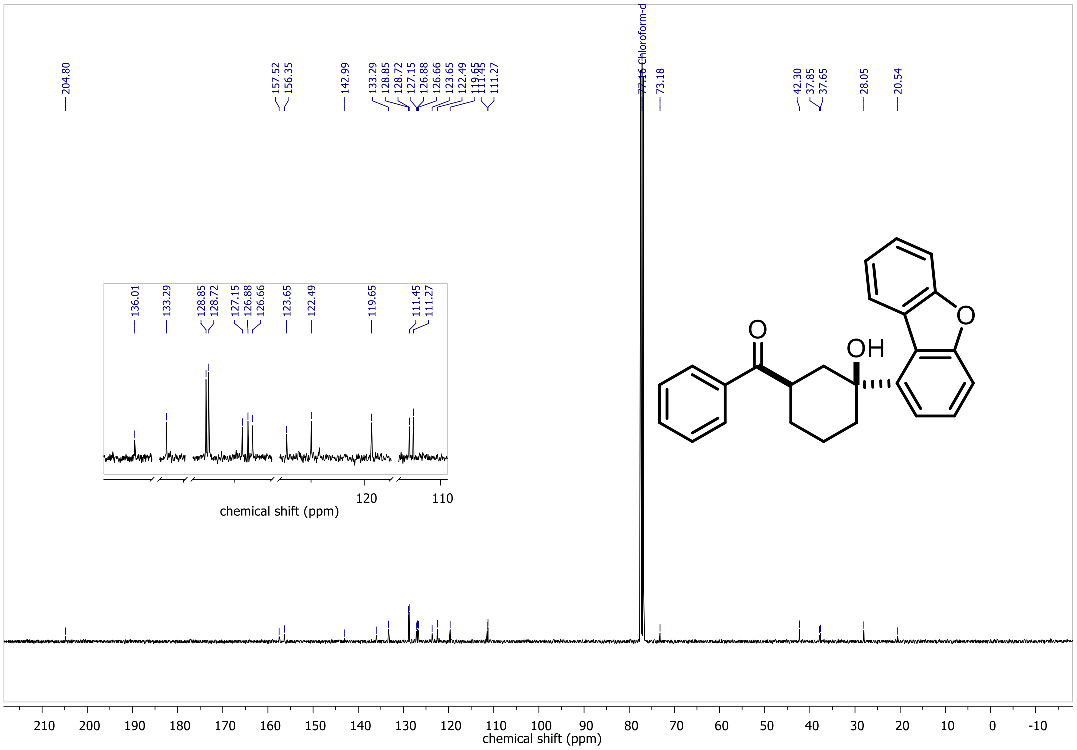

cis-1-(2-Benzoyl-1-methylcyclopentyl)ethan-1-one (2y)

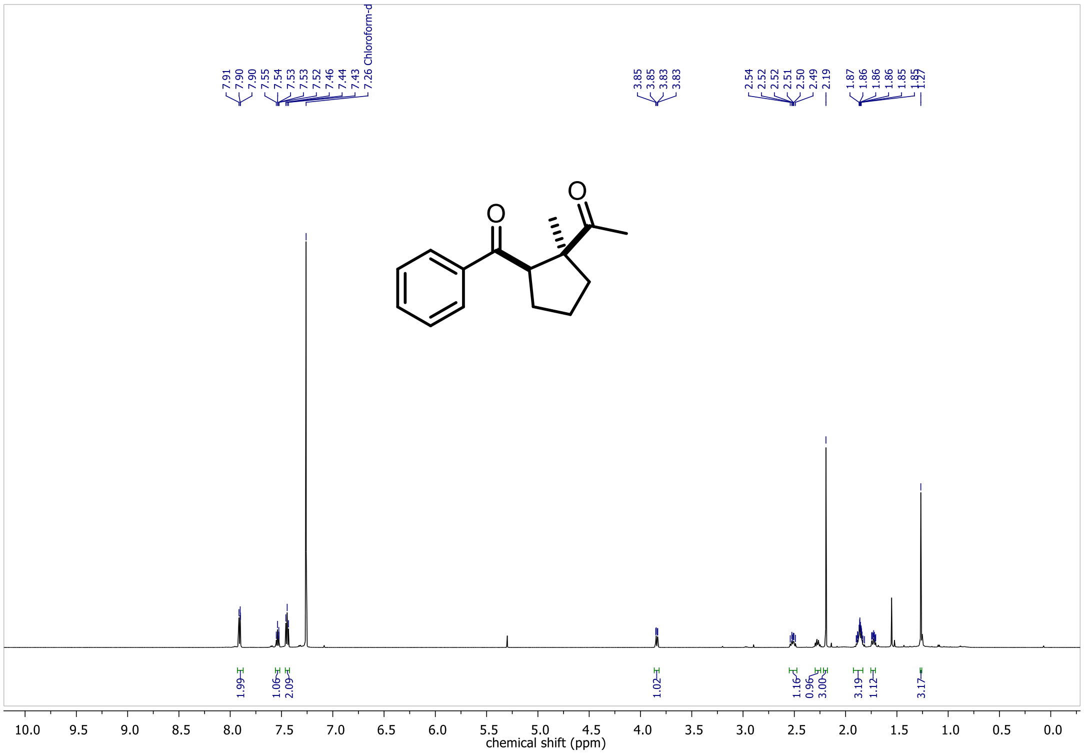

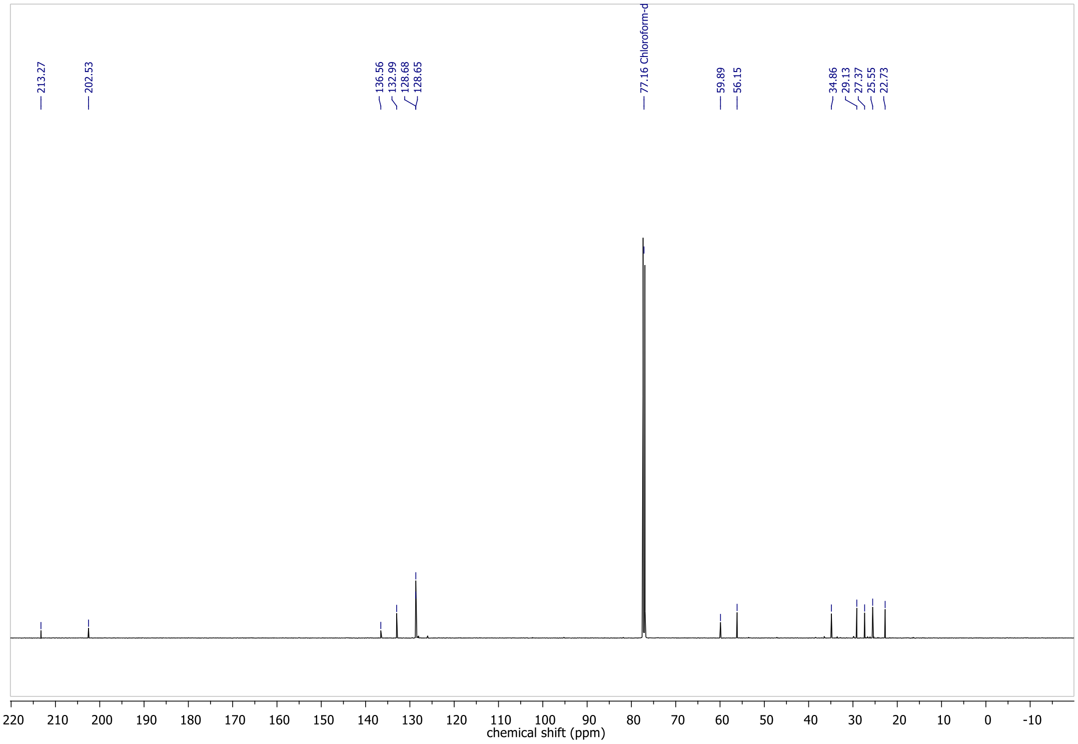

NOESY:

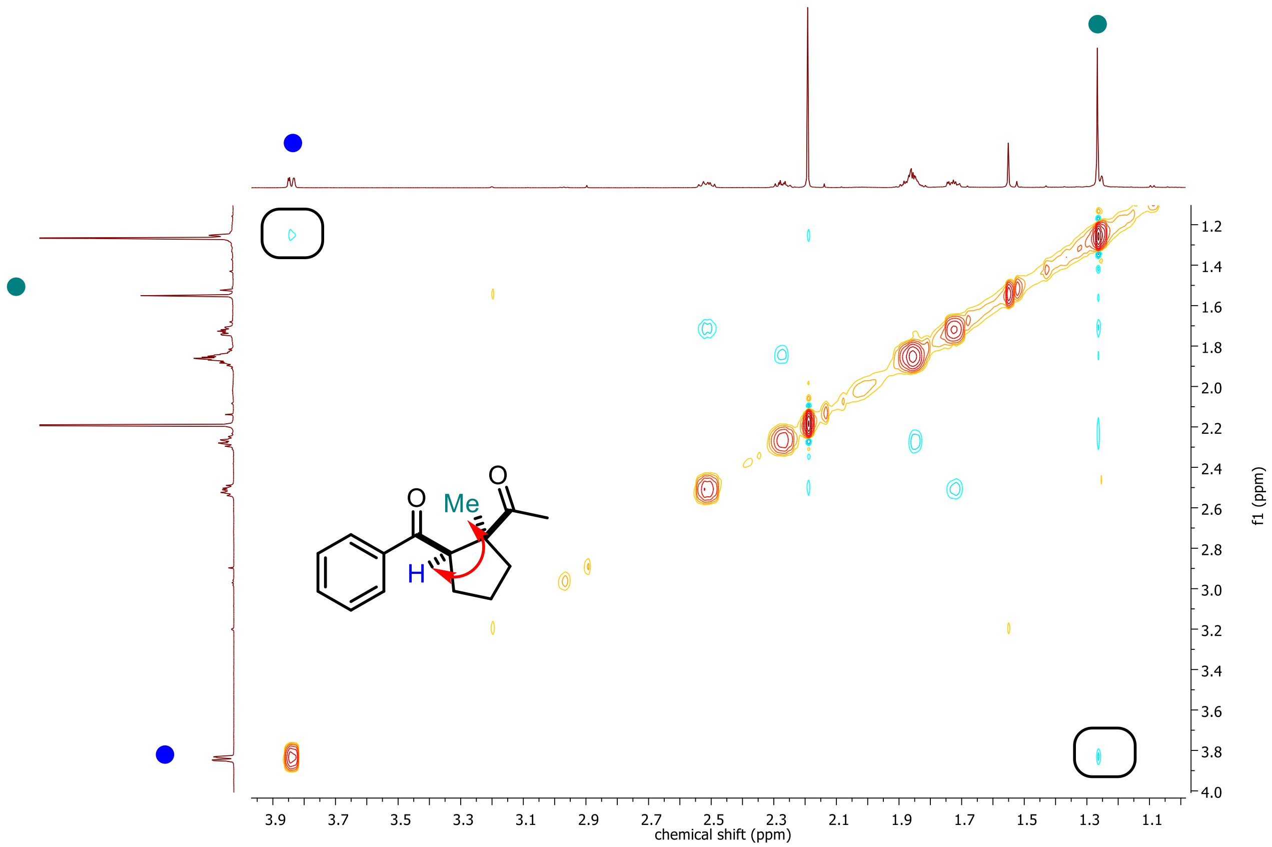

O
Me
O
H
1.2
1.4
1.6
1.8
2.0
2.2
2.4
2.6
2.8
3.0
3.2
3.4
3.6
3.8
4.0
f1 (ppm)
3.9
3.7
3.5
3.3
3.1
2.9
2.7
2.5
2.3
2.1
1.9
1.7
1.5
1.3
1.1
chemical shift (ppm)

2-(4-(Trifluoromethyl)benzoyl)cyclopentane-1-carbaldehyde (S2)

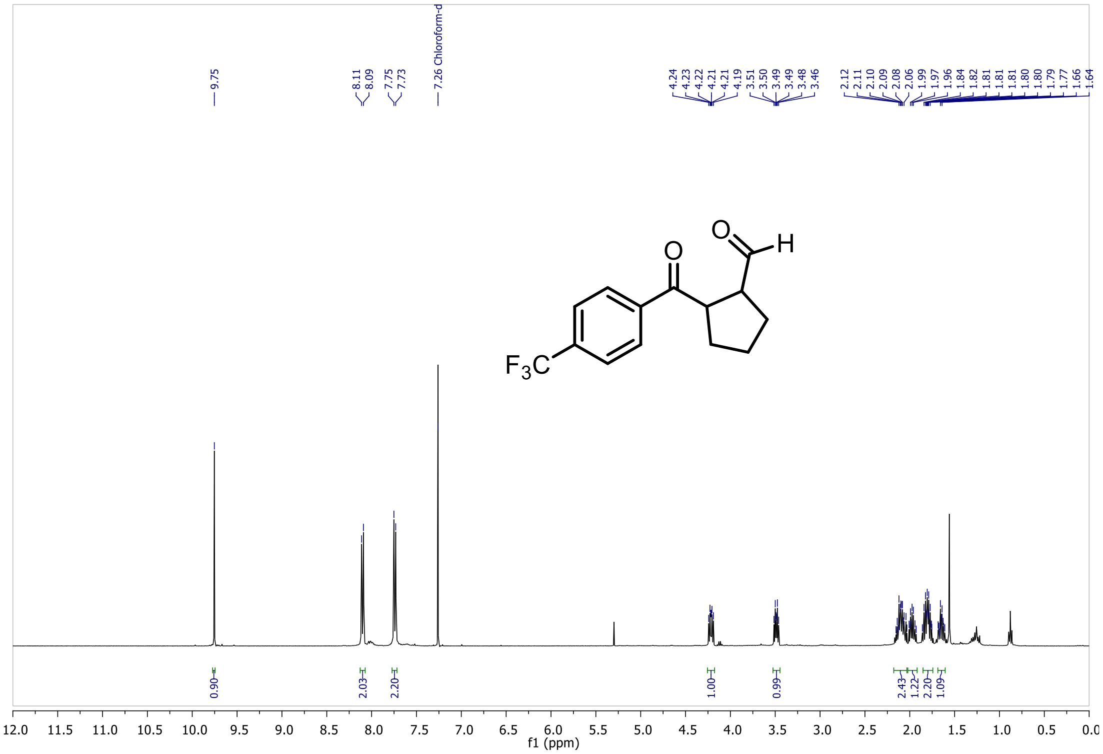

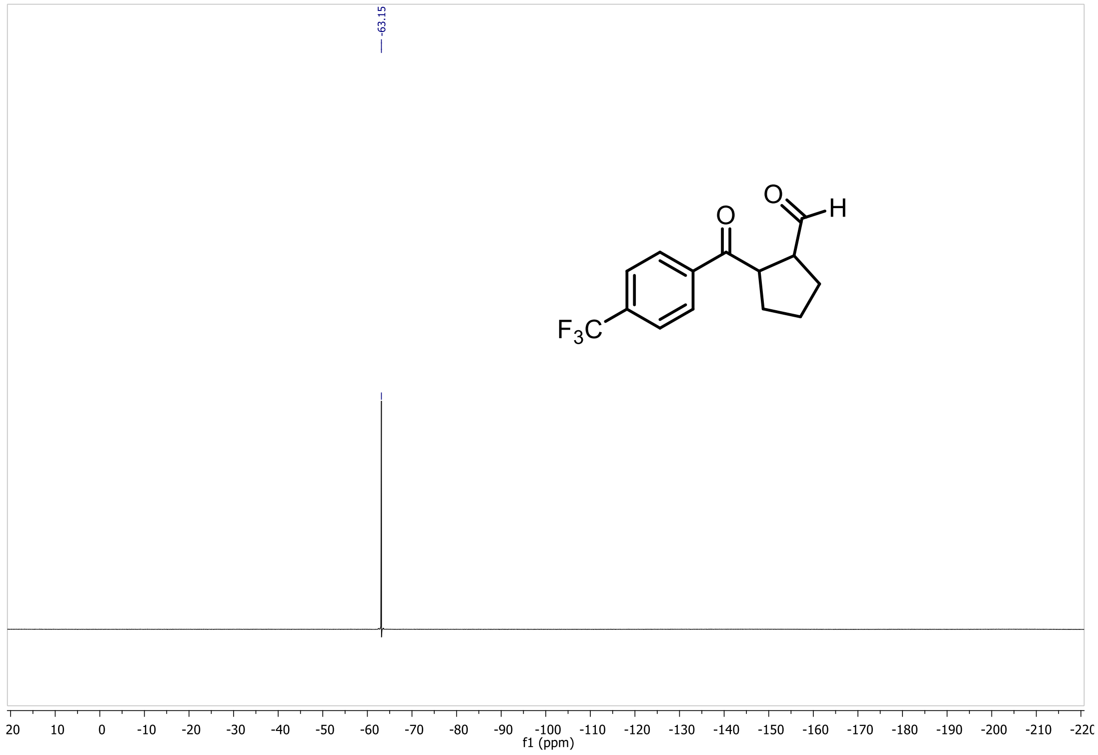

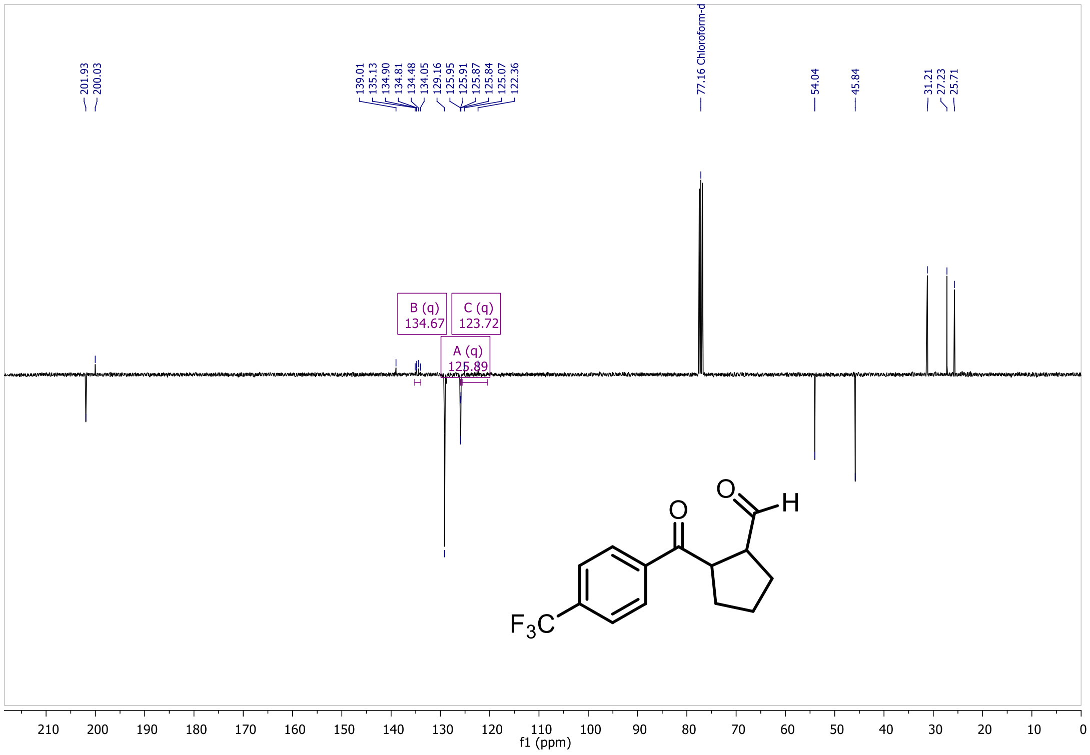
201.93
200.03
139.01
135.13
134.90
134.81
134.48
134.05
129.16
125.95
125.91
125.87
125.84
125.07
122.36
77.16 Chloroform-d
54.04
45.84
31.21
27.23
25.71
B (q)
134.67
C (q)
123.72
A (q)
125.89
O
H
O
F_3C
210
200
190
180
170
160
150
140
130
120
110
100
90
80
70
60
50
40
30
20
10
0
f1 (ppm)

7 References

1 V. V. Zhdankin, R. Tykwinski, B. Berglund, M. Mullikin, R. Caple, N. S. Zefirov and A. S. Koz'min, *J. Org. Chem.*, 1989, **54**, 2609–2612.

2 D. Karila, L. Leman and R. H. Dodd, *Org. Lett.*, 2011, **13**, 5830–5833.

3 W. J. Kerr, A. J. Morrison, M. Pazicky and T. Weber, *Org. Lett.*, 2012, **14**, 2250–2253.

4 T. G. Bolduc, C. Lee, W. P. Chappell and G. M. Sammis, *J. Org. Chem.*, 2022, **87**, 7308–7318.

5 N. Radhoff, C. G. Daniliuc and A. Studer, *Angew. Chem. Int. Ed.*, 2023, **62**, e202304771.

6 J. L. Krinsky, J. Arnold and R. G. Bergman, *Organometallics*, 2007, **26**, 897–909.

7 G. D. Kishore Kumar, G. E. Chavarria, A. K. Charlton-Sevcik, W. M. Arispe, M. T. MacDonough, T. E. Strecker, S.-E. Chen, B. G. Siim, D. J. Chaplin, M. L. Trawick and K. G. Pinney, *Bioorg. Med. Chem. Lett.*, 2010, **20**, 1415–1419.

8 J. B. Metternich, D. G. Artiukhin, M. C. Holland, M. von Bremen-Kühne, J. Neugebauer and R. Gilmour, *J. Org. Chem.*, 2017, **82**, 9955–9977.

9 M. Komatsuda, H. Ohki, H. Kondo Jr, A. Suto and J. Yamaguchi, *Org. Lett.*, 2022, **24**, 3270–3274.

10 B. Yang, X.-Y. Wang, X.-T. Huang, Z.-Y. Liu, X. Li, T. Huang, X.-S. Li, L.-Z. Wu, R. Fang and Q. Liu, *ACS Catal.*, 2023, **13**, 15331–15339.

11 D. N. Primer and G. A. Molander, *J. Am. Chem. Soc.*, 2017, **139**, 9847–9850.

12 A. Bourboula, O. G. Mountanea, G. Krasakis, C. Mantzourani, M. G. Kokotou, C. G. Kokotos and G. Kokotos, *Eur. J. Org. Chem.*, 2023, **26**, e202300008.

13 V. Vinayagam, T. V. Hajay Kumar, R. Nune, S. K. Karre and S. K. Sadhukhan, *J. Org. Chem.*, 2023, **88**, 2122–2131.

14 S. Lucas, R. Heim, M. Negri, I. Antes, C. Ries, K. E. Schewe, A. Bisi, S. Gobbi and R. W. Hartmann, *J. Med. Chem.*, 2008, **51**, 6138–6149.

15 M. Colella, A. Tota, Y. Takahashi, R. Higuma, S. Ishikawa, L. Degennaro, R. Luisi and A. Nagaki, *Angew. Chem. Int. Ed.*, 2020, **59**, 10924–10928.

16 Y. Yang, J. Liu, F. S. Kamounah, G. Ciancaleoni and J.-W. Lee, *J. Org. Chem.*, 2021, **86**, 16867–16881.

17 T. Niu, W. Zhang, D. Huang, C. Xu, H. Wang and Y. Hu, *Org. Lett.*, 2009, **11**, 4474–4477.

18 P. Knupe-Wolfgang, B. Mahn and G. Hilt, *Org. Lett.*, 2024, **26**, 6972–6976.

19 F. Huang and S. Zhang, *Org. Lett.*, 2019, **21**, 7430–7434.

20 X. Xu, C. Yang, S. Li, C. Meng, J. Yu, J. Yang and F. Li, *J. Catal.*, 2021, **402**, 335–343.

21 S. Guven, G. Kundu, A. Weßels, J. S. Ward, K. Rissanen and F. Schoenebeck, *J. Am. Chem. Soc.*, 2021, **143**, 8375–8380.

22 S. Chanthamath, S. Takaki, K. Shibatomi and S. Iwasa, *Angew. Chem. Int. Ed.*, 2013, **52**, 5818–5821.

23 L. Ackermann, A. Heidbreder, F. Wurche, F.-G. Klärner and J. Mattay, *J. Chem. Soc., Perkin Trans. 2*, 1999, 863–870.

24 J. B. Sperry, C. R. Whitehead, I. Ghiviriga, R. M. Walczak and D. L. Wright, *J. Org. Chem.*, 2004, **69**, 3726–3734.

25 B. R. Brutiu, G. Iannelli, M. Riomet, D. Kaiser and N. Maulide, *Nature*, 2024, **626**, 92–97.

26 A. Bauer, G. Di Mauro, J. Li and N. Maulide, *Angew. Chem. Int. Ed.*, 2020, **59**, 18208–18212.

27 S. Grimme, *J. Chem. Theory Comput.*, 2019, **15**, 2847–2862.

28 P. Pracht, F. Bohle and S. Grimme, *Phys. Chem. Chem. Phys.*, 2020, **22**, 7169–7192.

29 J. P. Perdew, K. Burke and M. Ernzerhof, *Phys. Rev. Lett.*, 1996, **77**, 3865–3868.

30 S. Grimme, J. Antony, S. Ehrlich and H. Krieg, *J. Chem. Phys.*, 2010, **132**, 154104.

31 S. Grimme, S. Ehrlich and L. Goerigk, *J. Comput. Chem.*, 2011, **32**, 1456–1465.

32 F. Weigend and R. Ahlrichs, *Phys. Chem. Chem. Phys.*, 2005, **7**, 3297–3305.

33 C. Adamo and V. Barone, *J. Chem. Phys.*, 1999, **110**, 6158–6170.

34 M. J. Frisch, G. W. Trucks, H. B. Schlegel, G. E. Scuseria, M. A. Robb, J. R. Cheeseman, G. Scalmani, V. Barone, G. A. Petersson, H. Nakatsuji, X. Li, M. Caricato, A. V. Marenich, J. Bloino, B. G. Janesko, R. Gomperts, B. Mennucci, H. P. Hratchian, J. V. Ortiz, A. F. Izmaylov, J. L. Sonnenberg, Williams, F. Ding, F. Lipparini, F. Egidi, J. Goings, B. Peng, A. Petrone, T. Henderson, D. Ranasinghe, V. G. Zakrzewski, J. Gao, N. Rega, G. Zheng, W. Liang, M. Hada, M. Ehara, K. Toyota, R. Fukuda, J. Hasegawa, M. Ishida, T. Nakajima, Y. Honda, O. Kitao, H. Nakai, T. Vreven, K. Throssell, J. A. Montgomery Jr, J. E. Peralta, F. Ogliaro, M. J. Bearpark, J. J. Heyd, E. N. Brothers, K. N. Kudin, V. N. Staroverov, T. A. Keith, R. Kobayashi, J. Normand, K. Raghavachari, A. P. Rendell, J. C. Burant, S. S. Iyengar, J. Tomasi, M. Cossi, J. M. Millam, M. Klene, C. Adamo, R. Cammi, J. W. Ochterski, R. L. Martin, K. Morokuma, O. Farkas, J. B. Foresman and D. J. Fox, *Gaussian 16 Rev. C.01*, Wallingford, CT, 2016.

35 E. Engelage, N. Schulz, F. Heinen, S. M. Huber, D. Truhlar and C. Cramer, *Chemistry*, 2018, **24**, 15983–15987.

36 E. Cancès, B. Mennucci and J. Tomasi, *J. Chem. Phys.*, 1997, **107**, 3032–3041.

37 J. Tomasi, B. Mennucci and R. Cammi, *Chem. Rev.*, 2005, **105**, 2999–3093.

38 A. V. Marenich, C. J. Cramer and D. G. Truhlar, *J. Phys. Chem. B*, 2009, **113**, 6378–6396.

39 S. Arava, J. N. Kumar, S. Maksymenko, M. A. Iron, K. N. Parida, P. Fristrup and A. M. Szpilman, *Angew. Chem. Int. Ed.*, 2017, **56**, 2599–2603.
